# Hepatic PPARα function and lipid metabolic pathways are dysregulated in polymicrobial sepsis

**DOI:** 10.15252/emmm.201911319

**Published:** 2020-01-09

**Authors:** Lise Van Wyngene, Tineke Vanderhaeghen, Steven Timmermans, Jolien Vandewalle, Kelly Van Looveren, Jolien Souffriau, Charlotte Wallaeys, Melanie Eggermont, Sam Ernst, Evelien Van Hamme, Amanda Gonçalves, Guy Eelen, Anneleen Remmerie, Charlotte L Scott, Caroline Rombouts, Lynn Vanhaecke, Liesbet De Bus, Johan Decruyenaere, Peter Carmeliet, Claude Libert

**Affiliations:** ^1^ Center for Inflammation Research VIB Ghent Belgium; ^2^ Department of Biomedical Molecular Biology Ghent University Ghent Belgium; ^3^ Bio Imaging Core VIB Center for Inflammation Research Ghent Belgium; ^4^ Laboratory of Angiogenesis and Vascular Biology VIB Center for Cancer Biology VIB Leuven Belgium; ^5^ Laboratory of Angiogenesis and Vascular Metabolism Department of Oncology and Leuven Cancer Institute (LKI) KU Leuven Leuven Belgium; ^6^ Faculty of Veterinary Medicine Department of Veterinary Public Health and Food Safety Laboratory of Chemical Analysis Ghent University Ghent Belgium; ^7^ Department of Critical Care Medicine Ghent University Hospital Ghent Belgium

**Keywords:** fibrates, lipid metabolism, lipotoxicity, liver, sepsis, Immunology, Metabolism

## Abstract

Despite intensive research and constant medical progress, sepsis remains one of the most urgent unmet medical needs of today. Most studies have been focused on the inflammatory component of the disease; however, recent advances support the notion that sepsis is accompanied by extensive metabolic perturbations. During times of limited caloric intake and high energy needs, the liver acts as the central metabolic hub in which PPARα is crucial to coordinate the breakdown of fatty acids. The role of hepatic PPARα in liver dysfunction during sepsis has hardly been explored. We demonstrate that sepsis leads to a starvation response that is hindered by the rapid decline of hepatic PPARα levels, causing excess free fatty acids, leading to lipotoxicity, and glycerol. In addition, treatment of mice with the PPARα agonist pemafibrate protects against bacterial sepsis by improving hepatic PPARα function, reducing lipotoxicity and tissue damage. Since lipolysis is also increased in sepsis patients and pemafibrate protects after the onset of sepsis, these findings may point toward new therapeutic leads in sepsis.

## Graphical abstract

Modulation of the immune response during sepsis remains insufficient for positive therapeutic outcome. This study highlights the contribution of lipid metabolic dysfunction to the pathology of sepsis, and downregulation of hepatic PPARα as a key factor in the metabolic dysregulation during sepsis.

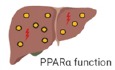

## Introduction

Sepsis is defined as a life‐threatening condition resulting from a dysregulated host response to infection and remains the major cause of deaths in intensive care units, with an overall mortality close to 25% (Singer *et al*, 2016). Sepsis hits some 19 million people yearly and is characterized by a major pro‐inflammatory status, intertwined with phases of immune suppression, which vary depending on time, tissue, and patient (Cavaillon & Giamarellos‐Bourboulis, 2019). These insights have encouraged the testing of a wide variety of immunomodulatory therapies in clinical trials over the past decades. Unfortunately, none of these treatments have demonstrated an actual survival benefit. As a consequence, the current management of sepsis is supportive rather than curative and focusses on the elimination of the pathogen, fluid resuscitation to preserve organ perfusion, maintaining adequate blood pressure, and mechanical support of failing organs (Evans, 2018). The lack of successful innovative therapeutics could be due to the prevailing notion that sepsis is a classical inflammatory condition, while more recent studies suggest that other pathways such as coagulation, circadian rhythm, and metabolism may play an important role (Cohen *et al*, 2015).

Classical features of sepsis, such as high fever, inflammation, immune activation, tachycardia, and the acute‐phase response, demand extra‐physiological energy that is supplied by the breakdown of carbohydrates, proteins, and lipids (Wolowczuk *et al*, 2008). Additionally, sepsis patients are often unable to eat, and optimal composition of parenteral and enteral feeding is still under discussion (Elke *et al*, 2015; Wischmeyer, 2018). Indeed, a strong decrease in ATP/ADP ratios in muscle, liver, and heart has been described in human patients and experimental animals (Brealey *et al*, 2002; Omachi *et al*, 2002; Correa *et al*, 2012). Moreover, extensive metabolic dysregulation has been reported in septic patients and several experimental animal models of systemic inflammation (Li *et al*, 2018).

The liver has an important function during inflammation since it contributes to the innate immune system by producing acute‐phase proteins and by phagocytosis of bacterial toxins by resident Kupffer cells (Sleyster & Knook, 1982; Ramadori & Christ, 1999). In addition, the liver is the central organ for carbohydrate, protein, and fat metabolism (Strnad *et al*, 2017). Liver dysfunction has been shown to occur during the early stages of sepsis and persists late into the immunosuppressive state (Wang *et al*, 1991). The initial dysregulation of hepatocellular function has been linked to problematic microcirculation and to the cytokine storm that is present early in the disease progression (Wang *et al*, 1997). However, the precise mechanism by which over‐activation of the immune system and liver dysfunction leads to liver failure remains unknown.

In times of energy deficit, lipids stored as triglycerides in adipose tissue belong to one of the largest endogenous energy supplies of the body (Cahill, 1970). The strong activation of the immune system during sepsis and the suboptimal feeding of many patients creates a state of energy deprivation that induces a starvation response (Rittig *et al*, 2016). First‐line energy‐supplying molecules such as glycogen and glucose are depleted within hours and are supplemented by lipids being released from the fat tissue, a process known as lipolysis (Nordenstrom *et al*, 1982). Several studies have confirmed that upon inflammation and infection, the release of free fatty acids (FFAs) from the white adipose tissue (WAT) into the bloodstream is increased (Forse *et al*, 1987; Wellhoener *et al*, 2011). During the starvation response, FFAs are mainly taken up by the liver to be oxidized in a process called β‐oxidation to provide energy and to produce ketone bodies, which are used as an energy source by the brain and other organs (Askanazi *et al*, 1980; Forse *et al*, 1987).

The oxidation of fatty acids is primarily controlled by the transcription factor peroxisome proliferator‐activated receptor alpha (PPARα, encoded by the *NR1C1* gene), which is highly expressed in liver and brown adipose tissue. PPARα is considered one of the major sensors of nutritional status that adapts metabolic homeostasis to energy deprivation (Polvani *et al*, 2016). The PPAR subfamily belongs to the family of nuclear receptors that share a conserved modular structure, consisting of an N‐terminal domain, important for transcriptional activation, a DNA‐binding domain that contains zinc fingers, a short hinge region, and the C‐terminal ligand‐binding domain. PPARα regulates transcription by forming a heterodimer with the retinoid X receptor (RXR) and recognizes specific DNA sequences referred to as PPAR response elements (PPREs; Dubois *et al*, 2017). Multiple studies have shown aberrant expression patterns of PPARα and its target genes in several tissues in the septic state (Wong *et al*, 2009; Standage *et al*, 2012). However, the mechanism behind this deficient signaling and the metabolic consequences during sepsis remain to be elucidated. Recently, Paumelle and colleagues have demonstrated that appropriate function of hepatic PPARα is crucial for survival of sepsis, induced by a bacterial infection in mice (Paumelle *et al*, 2019). We have confirmed this finding and found that hepatic PPARα function and signaling are altered at a genome‐wide level during sepsis, with deleterious effects on liver metabolic functions and health as a consequence. We also showed that modulating PPARα levels and activity with the agonist pemafibrate improves mortality in a CLP‐induced peritonitis sepsis mouse model by improving hepatic PPARα function and metabolic dysregulation.

## Results

### Hepatic PPARα action is hampered during sepsis

**Figure 1:**
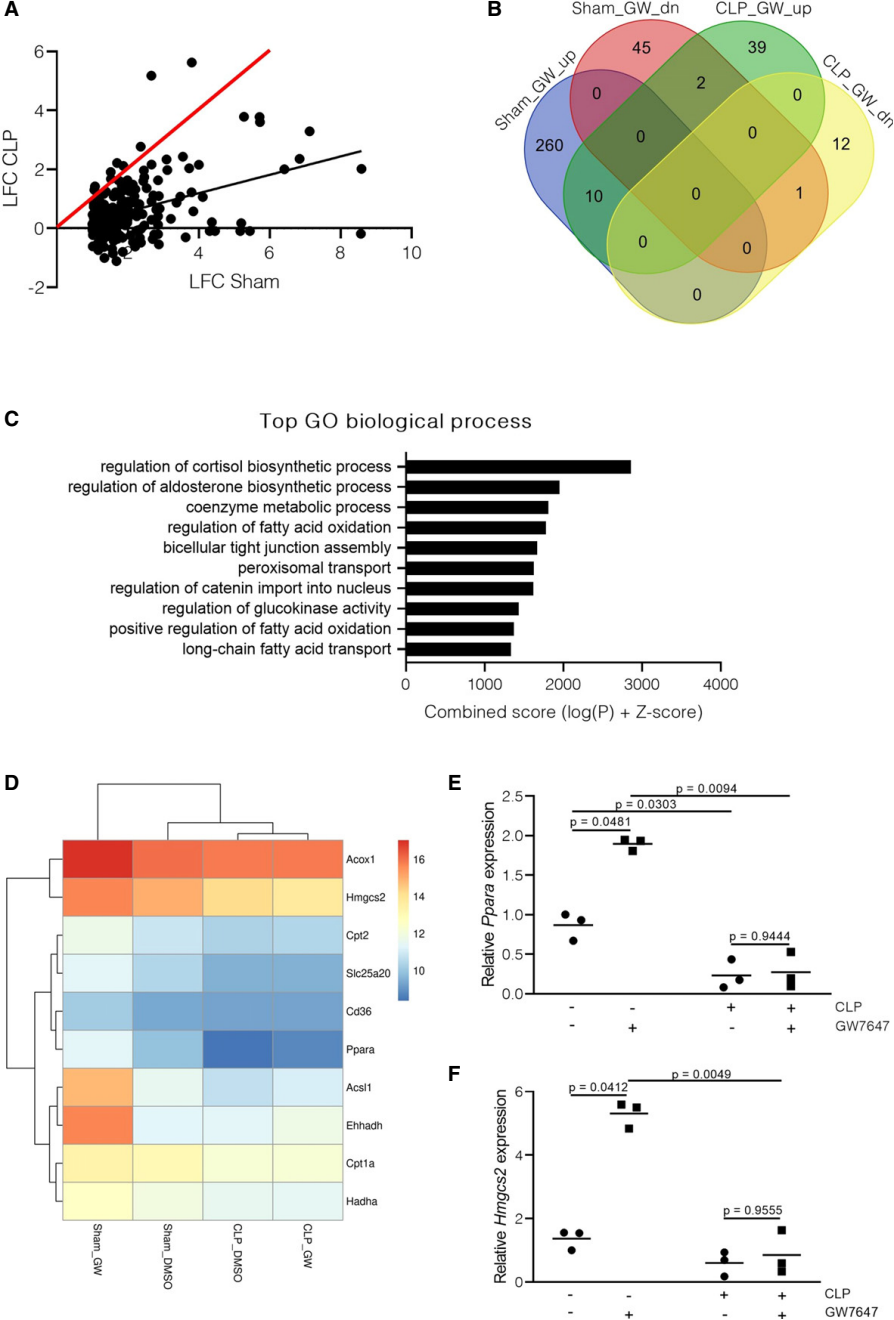
Hepatic PPARα signaling is disturbed at a genome‐wide level during sepsis A–DRNA‐seq of liver 10 h post‐sham or CLP. Mice (*n* = 3/group) underwent a sham or CLP operation and were injected with GW7647 (10 μg/g) 6 h post‐surgery, and after 4 h (total of 10 h), livers were isolated and RNA was prepared. (A) Scatter plot showing log fold change (LFC) of all GW7647‐upregulated genes (LFC > 0.8 and *P* < 0.05) in sham versus their LFC 10 h after CLP. The red line represents the diagonal, and the black line represents the real slope (0.3115) of the data. (B) Venn diagram depicting the amounts of genes upregulated (up) or downregulated (dn) by GW7647 in sham and CLP mice (LFC > 0.8 or < −0.8 and *P* < 0.05). (C) Top enriched gene ontology (GO) terms for genes that are downregulated in CLP mice without stimulation compared to unstimulated sham controls (LFC < −0.8 and *P* < 0.05). Composite of 3 datasets: CLP1 (6 h after CLP), CLP2 (8 h after CLP), and CLP3 (10 h after CLP). Analysis was performed with the Enrichr tool. (D) Heat map of differentially expressed genes in sham mice after GW7647 treatment, involved in β‐oxidation of fatty acids (unit scale bar = log_2_ of the normalized counts).E, FConfirmation of RNA‐seq data via qPCR on pure hepatocytes isolated via flow cytometry‐based sorting (*n* = 3/group). (E) *Ppara* and (F) *Hmgcs2* mRNA expression is shown as relative expression, normalized to housekeeping genes *Hprt* and *Rpl*. *P*‐values were calculated using 2‐way ANOVA analysis. Central lines represent mean. Source data are available online for this figure.

**Figure EV1:**
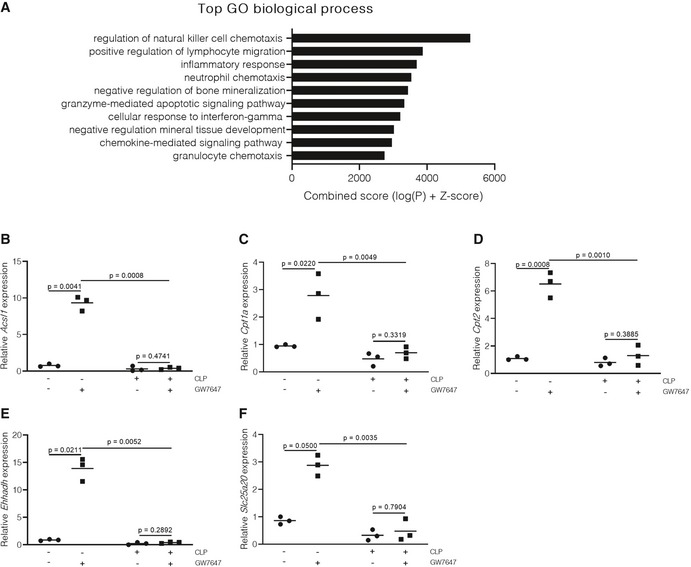
Hepatic PPARα signaling is disturbed at a genome‐wide level during sepsis ARNA‐seq of liver 10 h post‐sham or CLP. Mice (*n* = 3/group) underwent a sham or CLP operation and were injected with GW7647 (10 μg/g) 6 h post‐surgery, and after 4 h (total of 10 h), livers were isolated and RNA was prepared. Top enriched gene ontology (GO) terms for genes that are specifically upregulated by GW7647 in CLP mice. Analysis was performed with the Enrichr tool.B–FConfirmation of RNA‐seq data via qPCR on pure hepatocytes isolated via flow cytometry‐based sorting (*n* = 3/group). (B) *Acsl1*, (C) *Cpt1a*, (D) *Cpt2*, (E) *Ehhadh*, and (F) *Slc25a20* mRNA expression are shown as relative expression, normalized to housekeeping genes *Hprt* and *Rpl*. *P*‐values were calculated using 2‐way ANOVA analysis. Central lines represent mean.

Hepatic PPARα was previously shown to be crucial for survival during bacterial sepsis since mice lacking PPARα in the liver were sensitized to a lethal *E. coli* challenge and showed severe metabolic and inflammatory reprogramming (Paumelle *et al*, 2019). To investigate the functionality of liver PPARα during the CLP‐induced peritonitis sepsis mouse model, RNA sequencing was performed on livers of mice treated with CLP or sham operation, injected with either vehicle or the PPARα agonist GW7647 6h post‐operation. Gene expression was measured 4h after GW7647 administration. When plotting the log fold changes (LFCs) of all upregulated GW7647‐responsive genes (LFC > 0.8, *P* < 0.05) in sham mice versus their LFC after GW7647 in CLP, a clear shift toward a blunted PPARα activity was observed after CLP. (Fig 1A, black line (slope = 0.3115) versus red diagonal). Of the 270 genes that are significantly induced after GW7647 stimulation in sham mice, only 10 were still upregulated after CLP (Fig 1B). Similarly, of the 48 genes that are downregulated by GW7647 in the sham condition, 1 is still downregulated after CLP. After CLP, 39 genes were specifically induced by GW7647 in the sepsis condition, with most of these genes belonging to immune cell chemotaxis and pro‐inflammatory pathways, as shown by gene ontology (GO) term analysis (Fig EV1A and Dataset EV1). Of the 270 GW7647‐induced genes, 108 were found to be downregulated in livers of CLP mice without any stimulation, indicating that in addition to being non‐responsive to stimulation, PPARα downstream signaling is being downregulated after CLP. Moreover, GO term analysis of all the genes that are downregulated in liver after CLP compared with unstimulated sham controls revealed that these genes show enrichment for fat metabolic processes in which PPARα is involved such as fatty acid β‐oxidation and fatty acid transport (Fig 1C and Dataset EV2). As illustrated by the heat map, *Hmgcs2*,* Slc25a20*,* Cpt1a,* and other genes involved in β‐oxidation are indeed significantly upregulated by GW7647 in sham mice, unresponsive to GW7647 after CLP and downregulated by CLP (Fig 1D). Clustering analysis revealed that expression profiles of β‐oxidation genes are almost identical in CLP with or without GW7647 stimulation, while expression profiles of genes after GW7647 stimulation in sham mice were most distinctive. To confirm the contribution of hepatocytes to the GW7647 resistance, gene expression of *Ppara* and target genes was analyzed in a pure hepatocyte population, sorted by flow cytometry (Figs 1E–F and EV1B–F, Appendix Fig S1 for gating strategy). Together, these data demonstrate a reprogramming of PPARα signaling during sepsis in which pro‐inflammatory signaling is favored to activation of metabolic pathways. In addition, many GW7647‐responsive genes, including PPARα itself, are being downregulated during sepsis, which may cause severe disturbances in fatty acid metabolic pathways such as breakdown of fatty acids via β‐oxidation, energy generation, and ketone body formation.

### PPARα levels are downregulated in liver during sepsis

**Figure 2:**
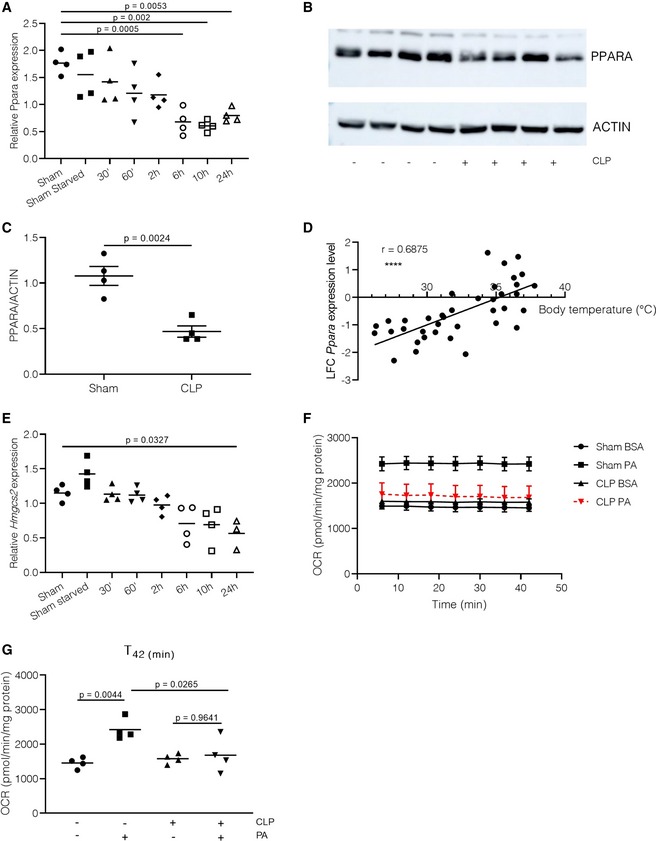
PPARα levels are decreased in the liver during sepsis and correlate with disease severity AMice (*n* = 4/group, data are representative of two experiments) underwent a sham (with or without starvation) or CLP operation, and liver was isolated on several timepoints post‐surgery for RNA preparation and qPCR. *Ppara* mRNA expression is shown as relative expression, normalized to housekeeping genes *Hprt* and *Rpl*. *P*‐values were calculated using 1‐way ANOVA analysis. Central lines represent mean.BPPARA protein levels were analyzed in livers 24h after sham or CLP by Western blot using actin as a loading control.CQuantification of PPARA Western blot. *P*‐value was calculated via 2‐way Student's *t*‐test (*n* = 4/group). Central line represents mean; error bars represent mean ± SEM.DPearson correlation between log fold change (LFC) *Ppara* expression levels and body temperature 24 h post‐sepsis (*n* = 38, *r* = 0.6875, combined data of four independent experiments).ELiver *Hmgcs2* mRNA expression at different timepoints post‐sepsis, expression is shown as relative expression, normalized to housekeeping genes *Hprt* and *Rpl*. *P*‐values were calculated using 1‐way ANOVA analysis (*n* = 4/group, data are representative of two experiments). Central lines represent mean.FOxygen consumption rates (OCRs) of liver tissue explants 24 h post‐sham or CLP. Liver tissue was isolated 24 h post‐surgery, and OCR was measured via Seahorse with BSA or palmitic acid (PA) as a substrate for 42 min. *n* = 4/group. Central line represents mean; error bars represent mean ± SEM.GVisualization of T_42(min)_ OCR, *P*‐values were calculated using 2‐way ANOVA analysis. Central lines represent mean. Source data are available online for this figure.

Downregulation of PPARα expression in whole blood of patients with septic shock was shown to be associated with decreased survival and increased bacterial load (Standage *et al*, 2012). In addition, one study has demonstrated a dose‐responsive downregulation of PPARα protein levels in hamster livers after LPS challenge (Beigneux *et al*, 2000). We hypothesized that the lack of transcriptional activity of PPARα during sepsis is due to decreased PPARα expression levels. By analyzing PPARα gene expression in liver at several timepoints after CLP, we found a time‐dependent gradual decrease in *Ppara* mRNA levels over time, with significant decreases in mRNA levels at 6, 10, and 24 h post‐sepsis initiation (Fig 2A). The decline in mRNA was reflected by significantly lower PPARA protein levels in liver 24 h after sepsis (Fig 2B and C). Reduced *Ppara* mRNA levels in liver during CLP‐induced sepsis were found to be a recurrent phenomenon, and a clear correlation was observed between body temperatures and PPARα expression levels, both measured 24 h after sepsis initiation (*r* = 0.6875, *P* < 0.0001; Fig 2D). This correlation implies that mice with higher PPARα expression levels have a higher body temperature, which may result in increased survival chances. As a consequence of the PPARα decline, some PPARα‐responsive genes such as *Hmgcs2* follow the gradual decline in mRNA levels in liver after sepsis (Fig 2E). Together, these data suggest a fast and strong downregulation of PPARα mRNA and protein levels in liver during sepsis. Since PPARα is the major transcription factor involved in β‐oxidation of fatty acids, we investigated the ability of liver explants to metabolize palmitic acid (PA) *ex vivo* via Seahorse technology. Liver explants of 24‐h‐starved sham mice showed an increase in oxygen consumption rate (OCR) when PA was added as a substrate instead of BSA, indicating increased activity of the β‐oxidative and oxidative phosphorylation pathway (Fig 2F–G, Appendix Fig S2 for all timepoints). This increase in metabolic activity was not observed in liver explants of CLP mice 24 h after sepsis initiation, suggesting that the decrease in PPARα levels and activity causes abnormalities in metabolic pathways such as the breakdown of fatty acids via β‐oxidation.

### Sepsis acutely activates lipolysis in fat tissue

**Figure 3:**
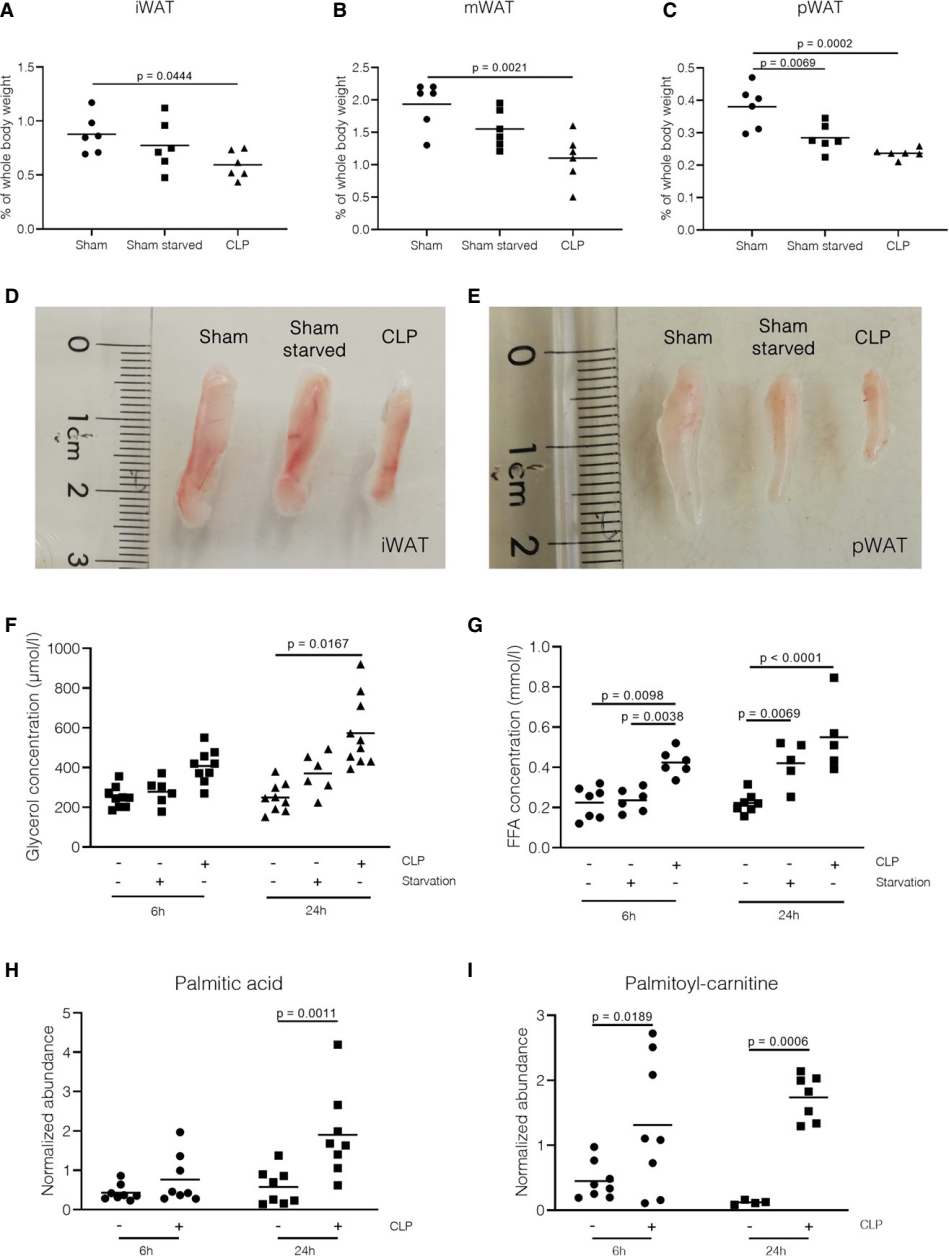
Lipolysis of subdermal and visceral fat pads is enhanced during sepsis A–CPercentage of body weight that is taken up by the inguinal fat pad (iWAT, A), the mesenteric fat pad (mWAT, B), and the perirenal fat pad (pWAT, C) 24 h post‐surgery in sham (with or without starvation) and CLP mice. *P*‐values were calculated with a 1‐way ANOVA test. Combined data of two independent experiments, *n* = 6/group. Central lines represent mean.D, EVisual representation of (D) iWAT and (E) pWAT sizes in sham, sham‐starved, and CLP mice 24 h post‐surgery. Data are representative of three independent experiments.F–IMice (*n* = 6–10/group) underwent a sham (with or without starvation) or CLP operation, and blood was collected 6 h and 24 h post‐surgery. Plasma was isolated, and (F) FFA concentration and (G) glycerol concentration were determined as described in the method section. *P*‐values were calculated with 2‐way ANOVA tests. Combined data of two independent experiments. (H–I) Normalized abundances of (H) palmitic acid and (I) palmitoyl‐carnitine determined via liquid chromatography‐mass spectrometric lipidomics. Values were normalized to IQ values, and *P*‐values were calculated with 2‐way ANOVA tests. *n* = 8/group. Central lines represent mean.

During a normal starvation response, fatty acids are being released from adipose tissue into the blood, by a process known as lipolysis, to provide energy by means of β‐oxidation (Cahill, 1970). Since sepsis reduces appetite and requires *supra*‐physiological energy supplies to fuel immune pathways, we believe sepsis mimics/exploits the starvation response. To monitor fat loss during sepsis, subcutaneous and visceral fat pads were weighed 24 h after the onset of sepsis. A significant decrease in the percentage of body weight taken up by the inguinal (iWAT), mesenteric (mWAT), and perirenal (pWAT) fat pads was observed 24 h after CLP compared with control sham mice, indicating a loss of fat tissue in septic mice (Fig 3A–C). Starvation of sham mice for 24 h led to significant decrease in weight of pWAT, however, not to the same extent as mice that underwent CLP. Visualization of iWAT (Fig 3D) and pWAT (Fig 3E) of sham, sham‐starved, and CLP mice 24 h post‐surgery confirmed the loss of fat mass after CLP. Activation of lipolysis is associated with an increase in free fatty acids (FFAs) and glycerol in the bloodstream. Both the concentrations of total FFAs and glycerol were increased in the blood of septic mice 6 h after the induction of sepsis, with a continued increase in FFA and glycerol plasma levels 24 h after sepsis (Fig 3F–G). Compared with 6‐h‐starved sham mice, CLP mice showed higher FFA levels, while glycerol levels showed a trend toward higher levels after CLP. Analysis of specific fatty acids in the blood 6 h and 24 h after sepsis initiation via liquid chromatography‐mass spectrometric lipidomics confirmed the increased levels of several FFAs and fatty acid (FA) carnitines, such as palmitic acid and palmitoyl‐carnitine, in plasma of septic mice (Fig 3H–I, Appendix Fig S3). Together, these data suggest an acute and accelerated increase in lipolysis in fat tissue during sepsis, leading to increased FFA and glycerol levels in the blood. Moreover, the increase in FA carnitines might indicate mitochondrial dysfunction with impaired import and β‐oxidation of fatty acids in the mitochondria.

### Sepsis causes ectopic lipid accumulation and lipotoxicity

**Figure 4:**
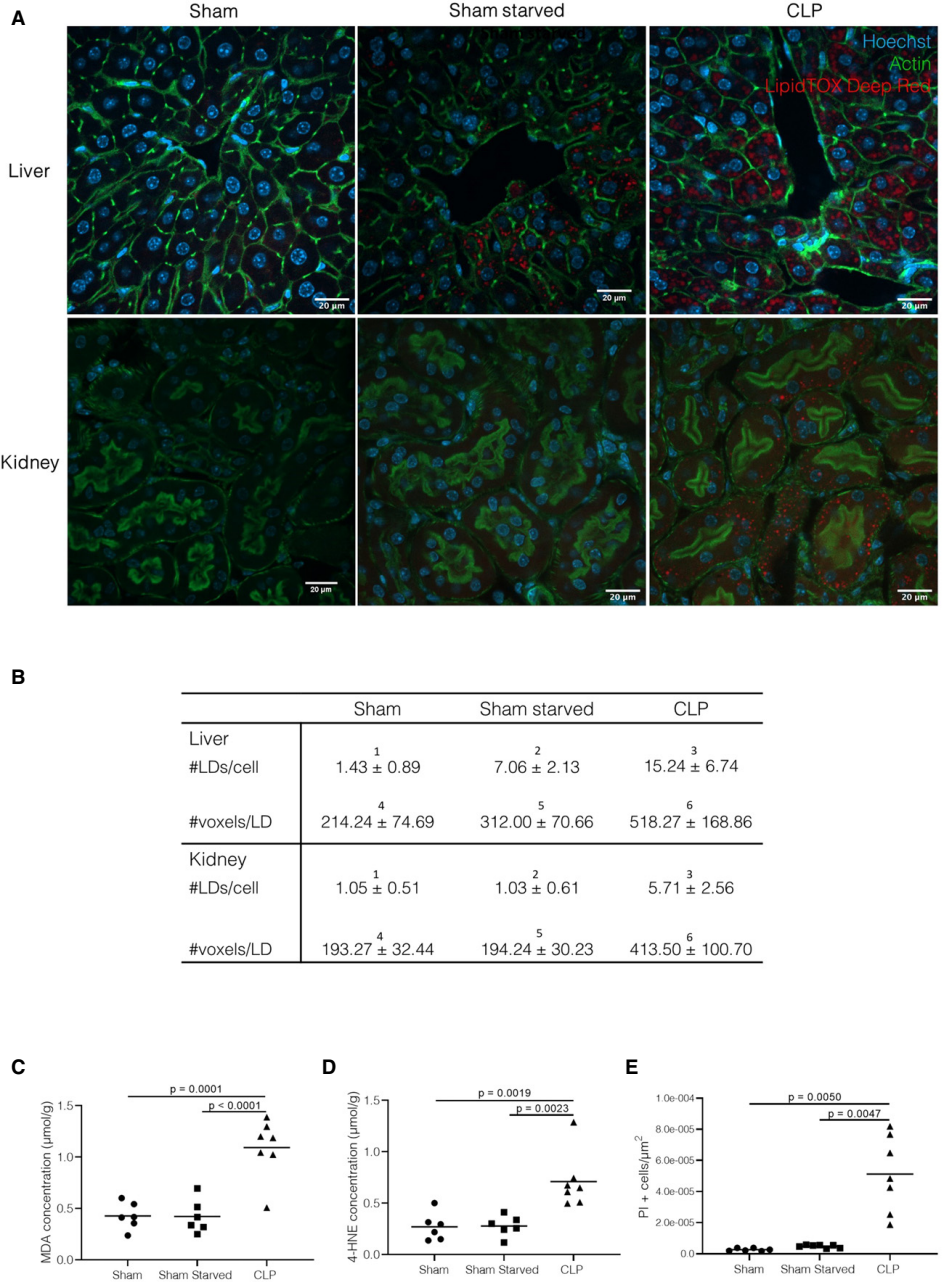
Lipids accumulate in liver and kidney during sepsis and cause lipotoxicity A, BImmunofluorescent images of liver and kidney 24 h after sham (with or without starvation) or CLP (*n* = 6–7/group, combined data of 2 independent experiments). White scale bar = 20 μm. (A) Cryosections were stained with Acti‐stain (green), Hoechst (blue), and LipidTOX (red). Z‐stacks were generated in 5–10 areas scattered across the entire tissue section. (B) The amount of lipid droplets (LDs)/cell and average size of LDs (represented by voxel counts) were calculated for each Z‐stack. Averages of the amount and size of LDs were converged for each mouse, and biological replicates are depicted in the table as mean ± SEM. *P*‐values were calculated using 1‐way ANOVA tests and can be found in Table EV1.C, DQuantification of lipid peroxidation by determination of (C) MDA and (D) 4‐HNE concentrations in liver homogenates 24 h post‐surgery in sham and CLP mice as described in methods (*n* = 6–7/group, combined data of two independent experiments). *P*‐values were calculated with 1‐way ANOVA tests. Central lines represent mean.EApoptosis in liver paraffin‐fixated sections 24 h after sepsis, measured by TUNEL staining, and presented as % PI‐positive cells/μm² tissue area. (*n* = 6/7 mice/group, combined data of two independent experiments). *P*‐values were calculated with 1‐way ANOVA tests. Central lines represent mean.

**Figure EV2:**
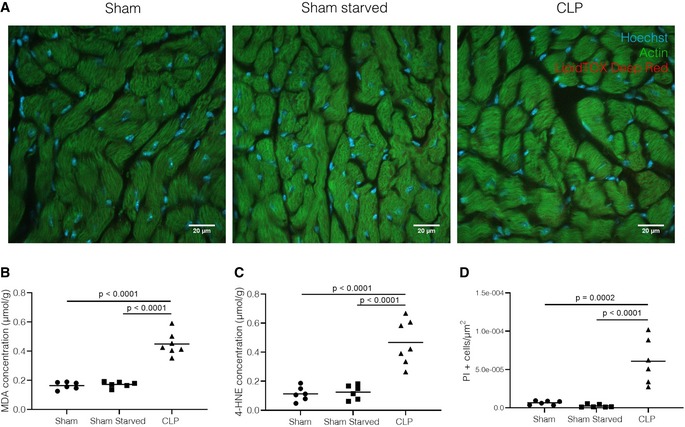
No lipid accumulation in heart after sepsis, lipotoxicity in kidney after sepsis AImmunofluorescent images of heart 24 h after sham (with or without starvation) or CLP (*n* = 6–7/group, data are representative of two experiments). Cryosections were stained with Acti‐stain (green), Hoechst (blue), and LipidTOX (red). Z‐stacks were generated in 5–10 areas scattered across the entire tissue section. The amount of lipid droplets/cell and average size of lipid droplets (represented by voxel counts) were calculated for each Z‐stack. White scale bar = 20 μmB, CQuantification of lipid peroxidation by determination of (B) MDA and (C) 4‐HNE concentrations in kidney homogenates 24 h post‐surgery in sham and CLP mice as described in methods (*n* = 6–7/group, combined data of two experiments). *P*‐value was calculated via one‐way ANOVA test. Central lines represent mean.DApoptosis in kidney paraffin‐fixated sections 24 h after sepsis, measured by TUNEL staining, and presented as % PI‐positive cells/μm² tissue area. *P*‐values were calculated with 1‐way ANOVA tests. Combined data of two experiments, *n* = 6–7/group. Central lines represent mean.

To protect tissues, lipid droplets are formed when fatty acid levels are increased in circulation, as is the case when lipolysis is increased (Plotz *et al*, 2016). Formation of lipid droplets and the release of lipids from the lipid droplet into the cytoplasm for further metabolic processing is tightly regulated by a coat of enzymes and proteins surrounding the lipid droplet (Barneda & Christian, 2017). Since lipolysis is increased during sepsis, we investigated the ectopic lipid accumulation in cryosections of liver, kidney, and heart by means of LipidTOX staining, a fluorescent dye with a high affinity for neutral lipids. Both liver and kidney showed extensive lipid droplet accumulation 24 h after sepsis initiation compared with healthy sham controls (Fig 4A). A 24‐h starvation of sham mice leads to a significant increase in lipid droplets in liver, but not to the same degree as seen in livers of septic mice. Lipid droplets were not observed in kidneys of sham mice after a 24‐h starvation, nor in heart tissue in any condition (Figs 4A and EV2A). Quantification of the amount of lipid droplets per cell and the average size of lipid droplets, expressed as voxel counts, confirmed the increased amount and size of lipid droplets in liver and kidney 24 h after sepsis (Fig 4B). Oxidative stress and the presence of reactive oxygen species have been described in liver and kidney in sepsis (Mantzarlis *et al*, 2017). Combined with leakage of lipids into the cytoplasm, the oxidative milieu could lead to lipotoxicity, a process characterized by the formation of toxic lipid radicals (Engin, 2017). As a measure of lipid peroxidation, we determined the presence of malondialdehyde (MDA) and 4‐hydroxynonenal (4‐HNE), which are the end products of lipid radical reactions. A significant increase in both MDA and 4‐HNE levels was observed in liver and kidney 24 h after CLP compared with healthy controls (Fig 4C and D, Kidney in Fig EV2B and C). In contrast, liver and kidney of 24‐h‐starved sham mice showed no increase in MDA and 4‐HNE levels. Lipid peroxides are highly reactive and have been shown to cause cell death and tissue damage (Ayala *et al*, 2014). In accordance with this, TUNEL staining in liver and kidney 24 h after CLP showed a significant increase in apoptotic cells compared with healthy controls, a phenomenon that was not observed in livers and kidneys of 24‐h‐starved mice (Fig 4E, Kidney in Fig EV2D). These data suggest that the increased presence of fatty acids in the blood may lead to ectopic deposition of lipid storages in liver and kidney after sepsis. Moreover, lipid peroxidation of excess lipids may cause lipotoxicity and could contribute to cell death in liver and kidney after sepsis.

### Pemafibrate boosts PPARα function and improves metabolic disease parameters during sepsis

**Figure 5:**
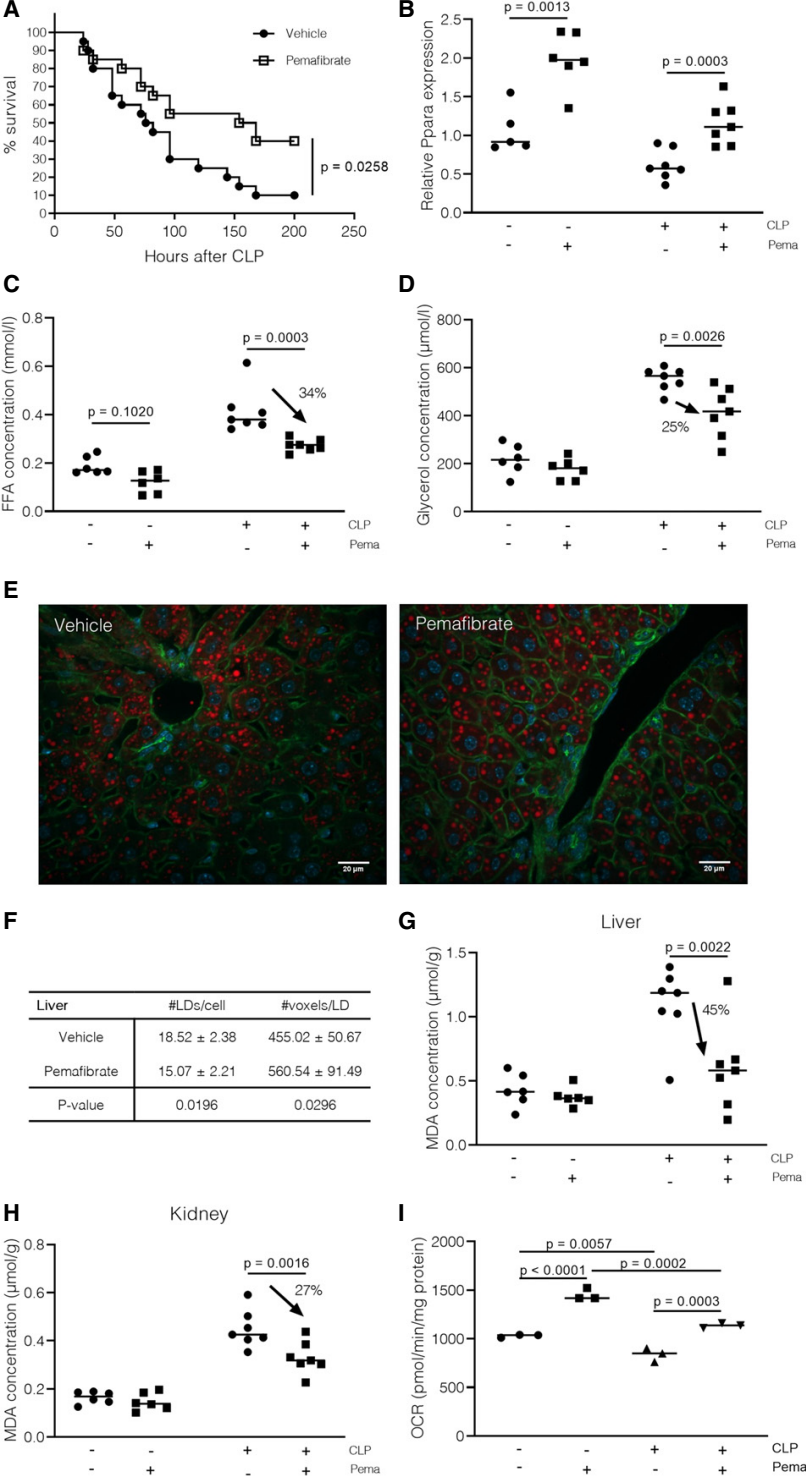
The PPARα agonist pemafibrate reduces mortality of sepsis by stimulating PPARα signaling and improving metabolic parameters. Mice were pretreated with pemafibrate (1 mg/kg) or vehicle (0,9% NaCl) for 1 week before being subjected to CLP ASurvival was monitored during 9 days, after which no further deaths occurred. Survival curve was analyzed via a log‐rank test. Combined data of three experiments, *n* = 20/group.BLiver samples were isolated 24 h after CLP (*n* = 5–7/group, combined data of two independent experiments), mRNA was prepared, and gene expression levels of *Ppara* were analyzed via qPCR. Gene expression values are shown as relative expression, normalized to housekeeping genes *Hprt* and *Rpl*. *P*‐values were calculated via 2‐way ANOVA test. Central lines represent mean.C, DPlasma was isolated 24 h after sepsis, and (C) FFA concentration and (D) glycerol concentration were determined as described in the method section. *P*‐values were calculated with 2‐way ANOVA tests. *n* = 5–7/group, combined data of two independent experiments. Arrows represent the % of decrease caused by pemafibrate treatment during sepsis. Central lines represent mean.EImmunofluorescent images of cryosections of liver 24h post‐surgery that were stained with Acti‐stain (green), Hoechst (blue), and LipidTOX (red). Z‐stacks were generated in 5–10 areas scattered across the entire tissue section. White scale bar = 20 μm.FThe amount of lipid droplets (LDs)/cell and average size of LDs (represented by voxel counts) were calculated for each Z‐stack. Averages of the amount and size of LDs were converged for each mouse, and biological replicates are depicted in the table as mean ± SEM. *P*‐values were calculated using two‐way Student's *t*‐tests. *n* = 6/group, combined data of two independent experiments.G, HQuantification of lipid peroxidation by determination of MDA concentration in (G) liver and (H) kidney homogenates 24 h post‐surgery in sham and CLP mice (*n* = 6–7/group, combined data of two independent experiments), as described in methods. *P*‐values were calculated with 2‐way ANOVA tests. Arrows represent the % of decrease caused by pemafibrate treatment during sepsis. Central lines represent mean.IOxygen consumption rates (OCRs) of liver tissue explants 24 h post‐sham or CLP (vehicle or pemafibrate‐treated) after supplementation of palmitic acid (PA), measured via Seahorse. Visualization of T_42(min)_ OCR. *P*‐values were calculates using 2‐way ANOVA analysis. One experiment, *n* = 3/group. Central lines represent mean.

**Figure EV3:**
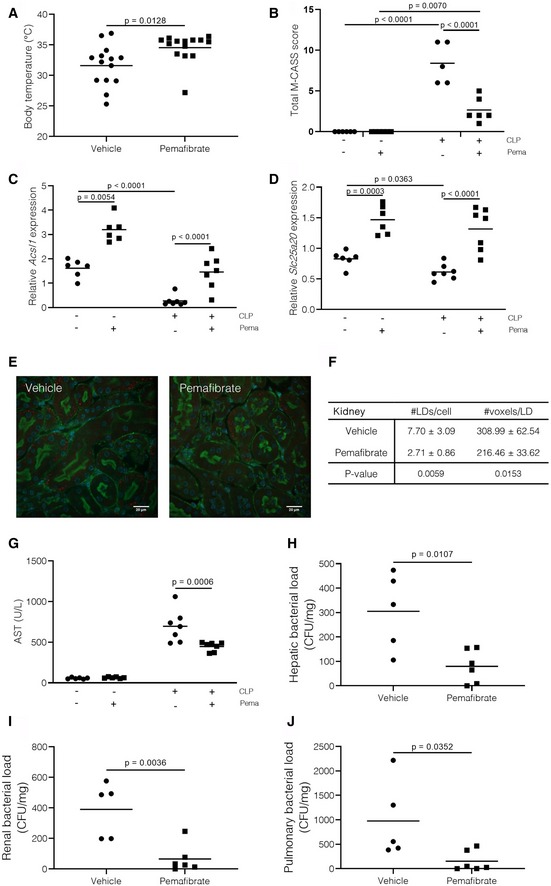
The PPARα agonist pemafibrate reduces mortality of sepsis by stimulating PPARα signaling and improving metabolic parameters Mice were pretreated with pemafibrate (1 mg/kg) or vehicle (0.9% NaCl) for 1 week before being subjected to CLP.
ABody temperature of mice 24 h post‐surgery. *P*‐values were calculated via 2‐way Student's *t*‐tests. Central lines represent mean.BMouse Clinical Assessment Score for Sepsis (M‐CASS) for sham septic mice 24 h post‐surgery. One experiment (*n* = 5–6/group). *P*‐values were calculated via 2‐way ANOVA. Central lines represent mean.C, DLiver samples were isolated 24 h after CLP (*n* = 5–7/group, data are representative of two experiments), mRNA was prepared, and gene expression levels of (C) *Acsl1 and* (D) *Slc25a20* were analyzed via qPCR. Gene expression values are shown relative expression, normalized to housekeeping genes *Hprt* and *Rpl*, and *P*‐values were calculated via 2‐way ANOVA. Central lines represent mean.EImmunofluorescent images of cryosections of kidney 24 h post‐surgery that were stained with Acti‐stain (green), Hoechst (blue), and LipidTOX (red). Z‐stacks were generated in 5–10 areas scattered across the entire tissue section. White scale bar = 20μm.FThe amount of lipid droplets (LDs)/cell and average size of LDs (represented by voxel counts) were calculated for each Z‐stack. Averages of the amount and size of lipid droplets were converged for each mouse, and biological replicates are depicted in the table as mean ± SEM. *P*‐values were calculated using unpaired *t*‐tests. *n* = 6/group, combined data of two experiments.GBlood was isolated 24 h post‐surgery, and plasma aspartate aminotransferase (AST) levels were determined as described in Materials and Methods. *P*‐values were calculated with 2‐way ANOVA tests. *n* = 6–7/group, combined data of two independent experiments. Central lines represent mean.H–JBacterial load was determined in (H) liver, (I) kidney, and (J) lung tissue homogenates 24 h post‐sepsis. Values are shown as CFU/mg tissue. *P*‐values were calculated using 2‐way Student's *t*‐tests. One experiment, *n* = 5–6 mice/group. Central lines represent mean.

We have shown that hepatic PPARα transcriptional function is severely dampened during sepsis, potentially due to a drastic decrease in hepatic PPARα mRNA and protein levels. Since the PPARα coding gene *PPARα* is a PPARα‐responsive gene itself, we hypothesized that pretreatment of mice with the PPARα agonist pemafibrate might increase PPARα gene expression, improve PPARα function, and protect mice during the CLP‐induced peritonitis sepsis model. A 1‐week pretreatment of mice with pemafibrate significantly reduced mortality from 90% to 50% compared with vehicle‐treated controls (Fig 5A), and this protection was associated with higher body temperatures in the pemafibrate‐treated group. (Fig EV3A). In addition, disease severity, assessed by usage of the Mouse Clinical Assessment Score for Sepsis (M‐CASS), was reduced in the pemafibrate group compared with vehicle‐treated control mice (Fig EV3B). Treatment with pemafibrate increased expression of *Ppara* in livers of sham mice (Fig 5B). Importantly, gene expression of PPARα and downstream genes was also increased after pemafibrate in livers of CLP mice 24 h post‐sepsis initiation, with many genes reaching expression levels close to those seen in vehicle‐treated sham mice (only *Acsl1* and *Slc25a20* are shown, Fig EV3C and D). Pemafibrate treatment decreased plasma FFA levels by 34% and glycerol levels by 25% compared with vehicle‐treated controls (Fig 5C and D). Although PPARα agonists have been shown to reduce circulatory lipids, clinical trials on non‐alcoholic fatty liver disease (NAFLD) have not reported any improvement in hepatic steatosis (Bajaj *et al*, 2007; Fernandez‐Miranda *et al*, 2008). Mice that were pretreated with pemafibrate for 1 week displayed less lipid droplets in liver 24 h after CLP, while the average size of the lipid droplets was increased in the pemafibrate‐treated group compared with vehicle‐treated septic mice (Fig 5E and F). In contrast, kidneys of pemafibrate‐treated mice had less lipid accumulation and smaller lipid droplets in comparison with untreated control mice 24 h after CLP (Fig EV3E and F). Lipotoxicity, assessed via MDA concentration measurement, was lower in livers and kidneys of pemafibrate‐pretreated mice 24 h after sepsis initiation (Fig 5G and H). Since pemafibrate treatment partly restored PPARα gene expression and reduced metabolic disease parameters, we investigated the functionality of the β‐oxidation pathway via Seahorse. Liver explants of CLP mice that were pretreated with pemafibrate displayed increased OCR upon PA addition compared with vehicle‐treated CLP mice, indicating that increased PPARα levels improved the flux through the hepatic β‐oxidation pathway, which may contribute to the decreased levels of systemic FFAs after sepsis (Fig 5I, all timepoints in Appendix Fig S4).

### Pemafibrate protects against sepsis by reducing tissue damage

**Figure 6:**
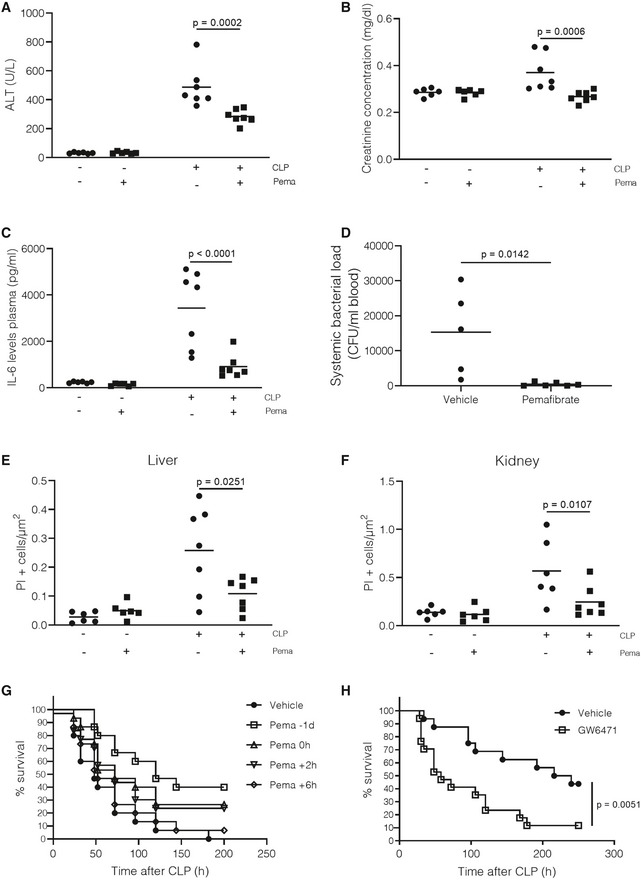
Pemafibrate reduces tissue damage during sepsis A–FMice were pretreated with pemafibrate (1 mg/kg) or vehicle (0.9% NaCl) for 1 week before being subjected to sham or CLP. (A–C) Plasma was collected 24 h post‐surgery, and (A) alanine aminotransferase (ALT), (B) creatinine concentrations, and (C) IL‐6 levels were measured as described in methods. *P*‐values were calculated with 2‐way ANOVA tests. *n* = 6‐7/group, combined data of two independent experiments. (D) Systemic bacterial load (CFU/ml blood) 24 h post‐sepsis in vehicle or pemafibrate‐treated mice. *P*‐values were calculated with 2‐way Student's *t*‐test. (E–F) Apoptosis in (E) liver and (F) kidney paraffin‐fixated sections 24 h after sepsis, measured with TUNEL staining, and depicted as % of PI‐positive cells/μm² tissue area. *P*‐values were calculated with 2‐way ANOVA tests. *n* = 6–7/group, combined data of two independent experiments. Central lines represent mean.GPemafibrate (1 mg/kg) or vehicle (0.9% NaCl) was administered at different timepoints before and after the induction of sepsis and survival was monitored during 9 days, after which no further deaths occurred. Survival curve was analyzed via log‐rank tests, and *P*‐values can be found in Table EV1. Combined data of 3 independent experiments, *n* = 15/group.HMice were injected with the PPARα antagonist GW6471 (10 μg/g) or vehicle (DMSO) 3 h pre‐CLP and 3h post‐CLP. Survival was monitored during 9 days, after which no further deaths occurred. Survival curve was analyzed via a log‐rank test. Combined data of two independent experiments, *n* = 16/group.

**Figure EV4:**
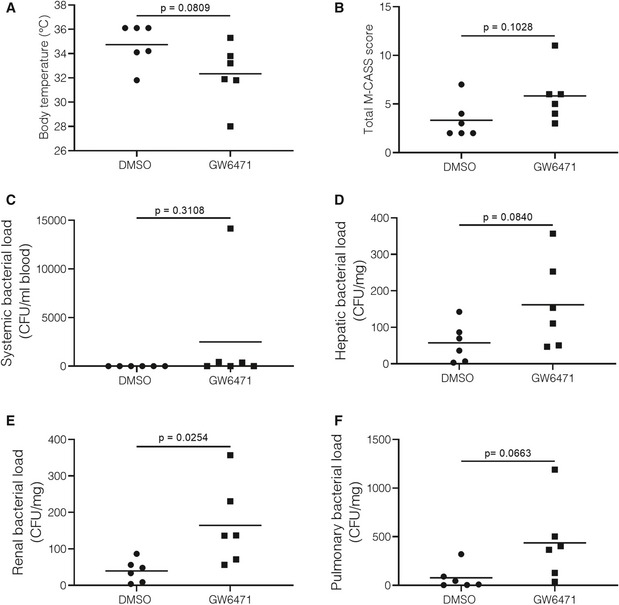
GW6471 treatment worsens septic disease parameters and bacterial load in blood and organs Mice were injected with the PPARα antagonist GW6471 (10 μg/g) or vehicle (DMSO) 3 h pre‐CLP and 3 h post‐CLP, 24 h post‐CLP blood, and organs were isolated (*n* = 5‐6/group). One experiment.
ABody temperature of mice 24 h post‐surgery. Central lines represent mean.BMouse Clinical Assessment Score for Sepsis (M‐CASS) for septic mice 24 h post‐surgery. Central lines represent mean.C–FBacterial load in (C) blood and (D) liver, (E) kidney, and (F) lung tissue homogenates of septic mice. Values are shown as CFU/mg tissue. Central lines represent mean.Data information: *P*‐values were calculated using two‐way Student's *t*‐tests.

Organ dysfunction is a well‐described phenomenon during sepsis and has been included into the most recent sepsis (Sepsis‐3) definition (Singer *et al*, 2016). Abnormal liver and kidney functions are frequently observed in septic patients and are reflected by increased alanine aminotransferase (ALT)/aspartate aminotransferase (AST) and creatinine blood levels, respectively. Pemafibrate treatment decreased ALT‐AST and creatinine plasma levels compared with vehicle‐treated controls 24 h after sepsis, indicating improved liver and kidney function (Fig 6A and B, AST levels in Fig EV3G). In addition, a decline in apoptotic cell death was observed in livers and kidneys, 24 h after sepsis initiation, of mice that were pretreated for 1 week with pemafibrate (Fig 6D and E). In addition to its well‐known metabolic function, PPARα has also been described to have limited anti‐inflammatory potential through the induction of anti‐inflammatory genes and direct inhibition of AP‐1 and NFKB (Delerive *et al*, 1999). Moreover, fibrates have been demonstrated to reduce systemic and organ bacterial loads through increased recruitment of neutrophils to the site of infection (Tancevski *et al*, 2014). In accordance, IL‐6 plasma levels were lower after pemafibrate treatment compared with vehicle‐treated controls 24 h after CLP, indicating a more controlled inflammatory environment during pemafibrate‐pretreated sepsis (Fig 6C). Also, mice that were treated with pemafibrate showed lower systemic, hepatic, renal, and pulmonary bacterial levels 24 h after sepsis initiation (Figs 6D and EV3H–J). These mechanisms could explain the reduction in sepsis‐induced apoptotic cell death we observed in liver and kidney after pemafibrate treatment (Fig 6E and F). To evaluate the therapeutic potential of pemafibrate, mice were supplemented with pemafibrate at different timepoints before and after the induction of sepsis (Fig 6G). A 1‐day pretreatment with pemafibrate protected mice to the same extent as the 1‐week pretreatment, while administration of pemafibrate at the time or 2 h after sepsis induction showed a lower but still significant protection. Pemafibrate demonstrated no protective effect when administered later in the disease progression. To determine whether inhibition of PPARα, by means of an antagonist, would lead to increased sensitivity to sepsis, mice were injected with the PPARα antagonist GW6471 3 h before and 3 h after sepsis was induced. Mice that were treated with GW6471 displayed a significant increase in mortality during CLP from ~44% to ~88%, and worsened disease and metabolic parameters, showing that inhibition of PPARα function is detrimental for survival during sepsis (Figs 6H and EV4). Together, these data demonstrate that by manipulating PPARα expression levels and function, mice can be protected or sensitized for CLP‐induced peritonitis. Moreover, pemafibrate pretreatment protects mice against sepsis through enhanced hepatic PPARα function, which in turn improves metabolic and inflammatory parameters, and reduces organ dysfunction.

### Lipolysis is increased in critically ill patients

**Figure 7:**
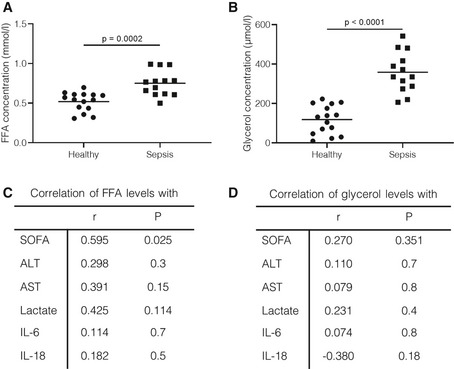
Lipolysis is increased in septic patients. Blood samples were collected from healthy volunteers and septic patients (*n* = 13 septic patients, =15 healthy controls) A, BPlasma was prepared, and (A) FFA and (B) glycerol concentrations were determined as described in the methods. *P*‐values were calculated with two‐way Student's *t*‐tests. Central lines represent mean.C, DCorrelation of (C) FFA and (D) glycerol concentrations with disease severity scores, lactate, or inflammatory cytokine levels from septic patients was calculated. Values are shown as Pearson correlation values (*r*) with associated *P*‐values.

Lipolysis of adipose tissue was shown to be increased in patients with septic shock, who had higher glycerol and FFA blood levels compared to patients without shock (Ilias *et al*, 2014). In confirmation of this observation, we found FFA and glycerol concentrations to be elevated in plasma of sepsis patients at day 1 of ICU admittance (Fig 7A and B). Higher FFA levels were correlated with patient SOFA scores and showed a trend toward correlation with AST and lactate plasma levels in septic patients (Fig 7C). However, plasma FFA levels were not correlated with IL‐6 or IL‐18 levels in these patients. Glycerol plasma levels were not correlated with disease severity or inflammatory status in sepsis patients (Fig 7D). These data suggest that lipolysis is involved in the metabolic dysregulation of tissue function but is not directly correlated with the inflammatory status of septic patients.

## Discussion

With 30 million cases per year and an overall mortality up to 25%, sepsis remains a highly unmet medical need despite many years of intensive research. Sepsis is associated with inflammation, but based on numerous clinical trials with inflammatory inhibitors, and since inflammation and metabolism are tightly intertwined, the focus of more recent research has shifted toward the metabolic perturbations during sepsis. Sepsis is characterized by a profound metabolic dysregulation in many metabolic pathways such as carbohydrate metabolism, amino acid metabolism, and fat metabolism. For a more detailed description on the metabolic changes during sepsis, we refer the reader to a recent extensive review (Van Wyngene *et al*, 2018).

One of the major causes of mortality of sepsis is the development of multiple organ dysfunction, followed by organ failure (Wang *et al*, 2014). Liver dysfunction is not very common in septic patients; however, when it develops into liver failure, it becomes a life‐threatening condition. Importantly, liver dysfunction was shown to have a remarkable prognostic relevance for the course of sepsis and a strong predictive capacity for mortality (Koch *et al*, 2011; Dizier *et al*, 2015). The liver has clear immunological defensive roles during sepsis, such as scavenging of bacteria and production of inflammatory cytokines, which are described in detail elsewhere (Wang *et al*, 2014; Strnad *et al*, 2017). In contrast, the metabolic changes and perturbations in liver during sepsis, with focus on fat metabolism in particular, remain largely unexplored.

Peroxisome proliferator‐activated receptor (PPAR)α is of major interest since it is the main transcription factor regulating fat catabolic pathways in the liver, and the knowledge of its role in sepsis is currently incomplete. Hepatic PPARα was recently shown to be crucial for survival during sepsis, induced by bacterial infection in mice, by controlling the metabolic response in hepatocytes (Paumelle *et al*, 2019). Opposed to the strong and rapid decline of PPARα expression levels we observed during sepsis, this study reported an increase in PPARα levels in liver after *E. coli* infection. A potential explanation for this discrepancy is the use of different animal models to induce sepsis. Bacterial injection models generally do not mimic the human septic condition completely, since there is often a steady stream of bacteria present in human sepsis, while injection of bacteria causes a sudden increase in bacterial load (Lewis *et al*, 2016). The widely used cecal ligation and puncture (CLP) is regarded as the golden standard as it mimics the progression and characteristics of human sepsis, especially peritonitis, better (Dejager *et al*, 2011). The use of different sepsis mouse models could elicit diverse temporal profiles of immunological and metabolic responses, ultimately leading to divergent causes of lethality. In support of our data, Paumelle *et al* (2019) reported lowered PPARΑ expression levels in livers of non‐surviving critically ill patients, a finding that should however be interpreted with caution since the livers were dissected post‐mortem.

Several studies have reported decreased PPARα levels in whole blood, liver, kidney, and heart during systemic inflammation (Beigneux *et al*, 2000; Feingold *et al*, 2008; Drosatos *et al*, 2011; Standage *et al*, 2012). However, the mechanisms behind the PPARα regulation during sepsis remain largely unknown and are possibly varying between tissues. One study reported that inhibition of the JNK pathways increased cardiac PPARα expression and prevented LPS‐induced cardiac dysfunction (Drosatos *et al*, 2011). Other studies have shown a link between increased levels of certain cytokines, such as IL‐6 and IL‐1β, and decreased PPARα expression (Chew *et al*, 2014; Chung *et al*, 2015). Moreover, miRNA‐dependent regulation of PPARs has been described in metabolic diseases and could play a role in PPARα downregulation during sepsis (Portius *et al*, 2017). PPAR expression has been demonstrated to be influenced by physical activity and cardiorespiratory fitness (Luquet *et al*, 2003; Queiroga *et al*, 2015); therefore, reduced locomotion should also be considered as a potential cause of decreased PPARα expression in the liver. Indeed, septic mice showed a reduction in fitness and activity, demonstrated by the increase in M‐CASS score (Fig EV3B), while treatment with pemafibrate improved the overall health of septic mice and thus improved locomotion and activity. Additionally, future experiments should also consider the effect of body and environmental temperature on PPARα expression, as PPAR levels have been linked to body temperature and the experiments in this study were not performed at rodent thermoneutral temperatures (30°C; Kersten *et al*, 1999; Cannon & Nedergaard, 2009; Chen & Yang, 2014). Next to decreased PPARα expression, we demonstrated lower levels of various PPARα‐responsive genes in livers of septic mice. Since many of these genes are involved in β‐oxidation of fatty acids, our findings are in line with the decreased respiratory activity of livers of septic mice in the presence of palmitic acid as a substrate for energy production.

In times of limited caloric intake, a starvation response is initiated in which lipids become the main source of energy to prevent muscle wasting (Cahill, 1970). In a process referred to as lipolysis, free fatty acids (FFAs) are being released from adipose tissue into the bloodstream, and subsequently taken up by peripheral organs to produce energy via β‐oxidation. Several studies have shown that the increased energy demands in the initial phase of sepsis are provided by lipid mobilization through activation of lipolysis in adipose tissue of septic patients (Askanazi *et al*, 1980; Nordenstrom *et al*, 1982; Rittig *et al*, 2016). Our analysis of FFA and glycerol levels in plasma of septic patients on day 1 of ICU admittance supports these findings, and the positive correlation between FFA levels and SOFA scores in these patients suggests that activation of lipolysis contributes to the metabolic dysregulation and poor prognosis during sepsis. Moreover, we have shown that compared to a normal starvation response, in which no acute inflammation is present, lipolysis is faster and stronger activated during sepsis. This acute activation of lipolysis in sepsis is necessary to provide substrates for β‐oxidation to produce energy and ketone bodies in liver and other lipid‐metabolizing organs such as kidney and heart (Fritz *et al*, 1962; Rossi *et al*, 1968). However, due to the downregulation of PPARα in liver, uptake and oxidation of fatty acids is slowed, and lipids accumulate in circulation, liver, and kidney as shown by lipidomics and LipidTOX analysis.

Hepatic lipid accumulation occurs as a transient metabolic adaption to starvation in which excess lipids are safely stored in lipid droplets to avoid toxic effects (Ohama *et al*, 1994). Build‐up of lipid droplets in liver was indeed observed during CLP; nonetheless, two important differences could be noted in comparison with a normal starvation response. First, livers accumulate more and bigger lipid droplets during sepsis and second, kidneys showed lipid droplet accumulation, a phenomenon that is never observed during a normal starvation response. Excessive lipid accumulation in circulation and organs, in particular in combination with the presence of reactive oxygen species (ROS) during sepsis, can lead to the production of toxic lipid by‐products, a process which is called lipotoxicity and often occurs through lipid peroxidation (Engin, 2017; Gai *et al*, 2019). Two well‐described lipid peroxidation by‐products, malondialdehyde (MDA) and 4‐hydroxynonenal (4‐HNE), were significantly increased in liver and kidney during sepsis and are highly reactive, causing toxicity by physical interaction with amino acid and nucleosides (Ayala *et al*, 2014). In addition, the fatty acid palmitate was upregulated in circulation during sepsis and has been demonstrated to have lipotoxic effects through various mechanisms (Park *et al*, 2014). Together, accumulation of certain fatty acids and toxic lipid peroxidation by‐products could contribute to the increased induction of apoptotic cell death in liver and kidney during sepsis. These results demonstrate that sepsis acutely activates lipolysis which, in combination with the failing β‐oxidation due to PPARα downregulation, leads to the accumulation of lipids in systemic circulation and peripheral organs. This accumulation may in turn cause lipotoxicity, could contribute to organ dysfunction, and ultimately lead to organ failure.

Fibrates are synthetic ligands of PPARα and are commonly used for treatment of metabolic disorders that display dyslipidemia, such as non‐alcoholic fatty liver disease (NAFLD), cardiovascular disease, and type 2 diabetes (Kondo *et al*, 2010; Elam *et al*, 2011). In addition to the lipid‐lowering properties of fibrates, potent anti‐inflammatory effects have been associated with fibrates as they reduce the expression of inflammatory genes, inhibit the release of pro‐inflammatory cytokines, and regulate the activation and function of inflammatory cells (Michalik & Wahli, 2006). Gemfibrozil and fenofibrate were shown to improve the course of bacterial sepsis by attenuating the inflammatory response, independent of the metabolic effects of fibrates (Tancevski *et al*, 2014; Camara‐Lemarroy *et al*, 2015). We found that administration of pemafibrate, a novel selective PPARα modulator (SPPARMα) with improved selectivity, potency, and safety profile (Ishibashi *et al*, 2016), improved PPARα function and reduced the metabolic dysregulation during sepsis. Although pemafibrate does not improve steatosis in the liver after sepsis, it did reduce the accumulation of toxic lipid peroxidation products and cell death in liver and kidney. Comparable to fenofibrate, pemafibrate reduced bacterial loads in circulation and target organs (Tancevski *et al*, 2014), indicating that through modulation of PPARα expression and metabolic pathways, pemafibrate may assist in the control of tissue infection and damage. Indeed, several studies have shown that metabolism is clearly linked to immunity and that the metabolic status of inflammatory cells changes their response to infection (Ganeshan & Chawla, 2014; Pearce & Pearce, 2018). However, as fibrates have many mechanisms of action and the liver is not the only organ affected during sepsis (Morel & Singer, 2014; Tancevski *et al*, 2014; Camara‐Lemarroy *et al*, 2015), it is essential to study the role of the liver in the protection of pemafibrate during sepsis, for example via the use of PPARα hepatocyte‐specific knock‐out animal models. A preliminary experiment suggests that the liver is crucial for the protective effect of pemafibrate in sepsis, since depletion of PPARα in the liver prevented pemafibrate‐mediated protection (Appendix Fig S5). Importantly, pemafibrate showed potential in a more therapeutic setting since it is able to protect mice against sepsis when administered early in the disease progression. These results demonstrate that the metabolic effects of fibrates through activation of PPARα contribute to the protective mechanism of fibrates in sepsis.

In conclusion, our results demonstrate that sepsis leads to an abnormal starvation response and major metabolic aberrations through acute activation of lipolysis in fat tissue and downregulation of PPARα in the liver. We suggest the use of fibrates as adjunct therapy for the treatment of sepsis as they hold considerable therapeutic potential by improving hepatic PPARα function and metabolic function during sepsis. However, the knowledge on dysregulation of PPARα signaling and metabolic pathways during sepsis is far from complete and warrants further study in both animal models of sepsis and septic patients.

## Materials and Methods

### Mice

Male mice (C57BL/6J) were ordered from Janvier (Le Genest‐St.Isle, France) and were housed in light‐controlled (14‐h light; 10‐h dark), air‐conditioned, specific pathogen‐free conditions with food and water *ad libitum*. All experiments were approved by the institutional ethics committee for animal welfare of the Faculty of Sciences, Ghent University, Belgium. The methods were carried out in accordance with the relevant guidelines and regulations. Mice were 8–10 weeks old at the time of the experiments.

#### Cecal ligation and puncture

Mice were subjected to CLP in order to induce polymicrobial septic shock, as described by a published standard operating procedure (Rittirsch *et al*, 2009). Briefly, mice were anesthetized by isoflurane inhalation and a one‐centimeter incision was made in the abdomen after which the cecum was exposed and 75% ligated. This was followed by making two punctures in the cecum with a 21‐gauge needle. During the procedure, some cecal content is pushed out using sterile forceps. The abdominal musculature and skin were closed with simple running sutures and metallic clips, respectively. The mice were resuscitated by intraperitoneal injection of an antibiotic cocktail containing ceftriaxone (25 mg/kg; Sigma‐Aldrich NV) and metronidazole (12.5 mg/kg; Sigma‐Aldrich NV) in 100 μl phosphate‐buffered saline (PBS) 10 h and 24 h after CLP onset. Disease severity was scored according to the M‐CASS scoring system (Mai *et al*, 2018). For experiments aimed to isolate blood and organ samples, sham‐operated mice of which the cecum was exposed but not ligated or punctured were used and are indicated as sham, or sham‐starved when food was taken away at the time of the surgery. For biochemical characterization, mice were euthanized via cervical dislocation at different timepoints post‐sepsis initiation and plasma and organs were collected.

### Reagents

#### Gw7647

GW7647 (Tocris Bioscience) was prepared as a solution of 2.5 mg/ml in DMSO. Mice underwent a sham or CLP procedure and received 7.5 mg/kg GW7647 6 h after sham or CLP surgery via intraperitoneal injection.

#### Pemafibrate

Pemafibrate (K‐877, Chemscene) was prepared as a solution of 200 μg/ml in 0.1% ethanol in 0.9% NaCl. Mice received 1 mg/kg of pemafibrate or 0.1% ethanol/0.9% NaCl (vehicle) via oral gavage at different timepoints before and after sepsis initiation. During the 1‐week challenge, mice were gavaged on alternating days, with 4 gavages in total. On day 7, the mice underwent a sham or CLP procedure. During lethality experiments, mice received an additional daily gavage of pemafibrate or vehicle. For biochemical characterization, mice were euthanized by cervical dislocation 24 h post‐surgery and plasma and organs were collected.

#### Gw6471

GW6471 (Tocris Bioscience) was prepared as a solution of 2.5 mg/ml in DMSO. Mice were injected intraperitoneally 3 h before and 3 h after sham or CLP surgery.

### Liver transcriptomic analysis

#### RNA sequencing

Total RNA was isolated with the RNeasy Mini Kit (Qiagen) according to the manufacturer's instructions. Biological triplicates were used for every condition. RNA concentration was measured, and RNA quality was checked with the Agilent RNA 6000 Pico Kit (Agilent Technologies) and sequenced on a Illumina Genome Analyzer IIx. Data were mapped to the mouse (mm10) reference genome transcriptome with tophat2 (Kim *et al*, 2013). Only uniquely mapped reads were retained. Gene‐level read counts were obtained with the HTSeq python package. Differential gene expression was assessed with the DESeq2 package, and the FDR was set at the 1% level. Gene ontology (GO) term enrichment on selected gene groups was performed via the Enrichr tool (Chen *et al*, 2013).

#### Real‐time qPCR

Liver was isolated and stored in RNA later (Life Technologies Europe) before RNA was isolated with the Aurum Total RNA Mini Kit (Bio‐Rad) according to the manufacturer's protocol. SensiFAST cDNA Synthesis Kit (GC Biotech BV) was used to reverse‐transcribe 1000 ng of RNA into cDNA. cDNA was diluted 20 times in ultrapure water for use in RT–PCRs. RT–PCR primers for used targets are listed in supplementals. RT–PCR was performed with SensiFAST SYBR No‐ROX Mix (Bioline) and was performed in duplicate in a Roche LCII 480. Relative expression of targets was calculated by comparison with HPRT and RPL expression in qBase^+^ software (Biogazelle, Gent, Belgium). Primer sequences for qPCR can be found in Appendix Table S4.

### Biochemical analysis

Analysis of mouse plasma aspartate aminotransferase (AST), alanine aminotransferase (ALT), and creatinine levels was kindly provided to us by the University Hospital of Ghent. Free fatty acid (FFA) (Abnova) and glycerol (Cayman Chemical) were determined via colorimetric assays. Plasma IL‐6 levels were measured by ELISA (eBioscience). Organ MDA and 4‐HNE levels were determined via the principle of the lipid peroxidation assay (Eagle Biosciences).

### FAO metabolic assay

An XF‐24 Extracellular Flux Analyzer (Seahorse Bioscience) was used for fatty acid oxidation (FAO) measurement of liver explants. Liver tissue (~10 mg) was incubated in FAO buffer (111 mM NaCl, 4.7 mM KCl, 1.25 mM CaCl_2_, 2.0 mM MgSO_4_, 1.2 mM NaH_2_PO_4_, 2.5 mM glucose, 0.5 mM carnitine, 5 mM HEPES) immediately after isolation. OCR was measured at basal level after addition of BSA or palmitic acid (PA) (Seahorse XF Palmitate‐BSA FAO Substrate, Agilent). Protein concentration of tissue extracts was measured via the Bradford protocol and was used to normalize OCR values.

### Bacterial load quantification

Systemic bacterial load was determined through plating of 100 μl of blood on tryptic soy agar (TSA) plates. 24 h post‐inoculation at 37°C, colony‐forming units (CFUs) were counted and CFU/ml was determined. Organ bacterial loads were analyzed as followed: 100 mg of liver and kidney, and 30 mg of lung tissue were isolated 24 h post‐sepsis and homogenized in 1ml of sterile PBS. Bacterial load was determined via plating of 100 μl of tissue homogenates on lysogeny broth (LB) agar plates. 24 h post‐inoculation at 37°C, CFUs were counted and CFU/mg of tissue was calculated.

### Flow cytometry

#### Liver digestion and hepatocyte purification

Liver perfusion and hepatocyte isolation were performed as described by Bonnardel *et al* (2019).

#### Cell sorting and RT–qPCR

Single‐cell hepatocyte suspensions (0.5–5 × 10^6^ cells) were stained with appropriate antibodies (Appendix Table S1) at 4°C for 20 min in the dark. FCBlock 2.4G2 antibody was used to minimize non‐specific binding of antibodies to FcR‐bearing cells. Hepatocytes were sorted as live‐gated CD45^−^ Lyve‐1^−^ cells using an ARIA III (BD, Biosciences). The gating strategy can be found in Appendix Fig S1. Final analysis and graphical output were performed using FlowJo software (Tree Star, Inc.). For each sample, 20,000 hepatocytes were sorted into RLT (1% β‐mercaptoethanol) lysis buffer and RNA was prepared using the RNeasy Plus Micro Kit (Qiagen Benelux B.V.) as described by the manufacturer's protocol. All RNA was used to synthesize cDNA with the SensiFAST cDNA Synthesis Kit (GC Biotech BV) and diluted 10× in ultrapure water before RT–PCR was performed with SensiFAST SYBR No‐ROX Mix (Bioline) in duplicate in a Roche LCII 480. Relative expression of targets was calculated by comparison with β‐actin expression.

### Western blot analysis

For the detection of PPARα, protein was isolated out of snap‐frozen liver tissue with RIPA lysis buffer, supplemented with protease inhibitor cocktail (Roche). Protein samples containing 30 μg of protein were separated by electrophoresis in a 10% gradient SDS polyacrylamide gel and transferred to nitrocellulose membranes (pore size, 045 μm). After blocking the membranes with a &frac12; dilution of Starting Block/PBST 0.1% (Thermo Fisher Scientific), membranes were incubated overnight at 4°C with a primary antibody against PPARα (1:1,000, catalog sc‐398394, Santa Cruz Biotechnology). Blots were washed with PBST 0.1% and then incubated for 1 h at room temperature with anti‐mouse HRP antibody (1:10,000, catalog GENA931, Sigma‐Aldrich NV.). Immunoreactive bands were visualized detected and quantified using an Amersham Imager 600 (GE Healthcare Life Sciences). After visualization of PPARα, the process was repeated with a primary antibody against Actin (1:5,000, catalog MA5‐15739, Life Technologies Europe).

### Histological analysis

#### TUNEL

TUNEL staining for detection and quantification of apoptosis in mouse liver and kidney paraffin‐embedded sections was performed using the “*In Situ* Cell Death Detection Kit, TMR Red” (Sigma‐Aldrich N.V.), according to a standard protocol. DNA strand breaks were labeled with fluorescein (TMR Red) and imaged by fluorescence microscopy.

#### LipidTOX

Cryostat sections 20 μm in thickness were rehydrated in PBS for 5 min after which the sections were blocked in blocking buffer (2% BSA, 1% fetal calf serum, 1% goat serum, in 0.5% saponin) for 30 min at RT. The antibody mix (LipidTOX Deep Red (1:400, Life Technologies Europe B.V.); Acti‐stain 488 Phalloidin (1:150, Cytoskeleton Inc.)) was added and incubated for 2 h at RT. After washing with PBS for 5 min, nuclear staining (Hoechst (1:1,000, Sigma‐Aldrich N.V.)) was added for 5 min at RT. Slides were washed in PBS for 5 min, quickly rinsed in water to remove residual salt, and mounted. For each cryosection, Z‐stacks of 5–10 areas were imaged with a spinning disk confocal microscope (Zeiss), using a 40× Plan‐Apochromat objective lens (1.4 Oil DIC (UV) VIS‐IR M27)) at a pixel size of 0.167 μm and at optimal Z‐resolution (240 mm). Z‐stacks were processed in Volocity (PerkinElmer), and the amount of lipid droplets and average size of lipid droplets (depicted as voxels) was calculated.

### Lipidomics

#### Extraction

In short, a total volume of 3 ml of methyl tert‐butyl ether with 0.01% BHT (w/v) was added to 40 μl of mouse plasma. This mixture was vortexed for 30 s, and the sample was shaken for 20 min at 200 rpm at 20°C in an incubator (New Brunswick Innova 42, Eppendorf). Next, 1 ml of ultrapure water with 5% trichloroacetic acid (w/v) was added to induce phase separation, which was enforced by centrifugation for 5 min at 960 *g* at 20°C. Subsequently, 500 μl of the upper layer, consisting of methyl tert‐butyl ether, was collected and 50 μl of methanol internal standard mixture (25 ng/μl) was added before being evaporated to dryness at 30°C under a gentle stream of nitrogen. The residue was dissolved in 100 μl of chloroform and 250 μl of methanol, after which 100 μl of the solution was transferred to an amber glass vial. An aliquot (5 μl) of sample was injected into the chromatographic system. Liquid chromatography was achieved on a Dionex UltiMate 3000 XRS UHPLC system (Thermo Fisher Scientific), and MS analysis was carried out by high‐resolution hybrid quadrupole Q‐Exactive Orbitrap MS (Thermo Fisher Scientific) as previously published (Van Meulebroek *et al*, 2017).

#### Targeted analysis

Analytical reference standards were purchased from Sigma‐Aldrich. A selection of the reference standards can be found in Appendix Table S3, and for the full standard list, we refer the reader to Van Meulebroek *et al* (2017). To correct for instrumental drift, biological samples, i.e., quality control (QC) samples, were used. These QC samples are considered as representative bulk control samples and were prepared by pooling all samples. QC samples were dispersed evenly across the sample batch, and duplicate QC injections were performed after every ten samples. The average signal of those two injections was used for normalizing the ten preceding samples. Samples were injected in a randomized order. XCalibur 3.0 software (Thermo Fisher) was used for targeted processing of full‐scan data, including identification and quantification of lipid target compounds. Identification of a compound was done by use of the m/z value of the molecular ion (mass deviation ≤ 3 ppm) and the retention time relative to that of an internal standard (deviation ≤ 2.5%), all being determined from the corresponding analytical standard.

### Human study

The clinical study protocol was approved by the ethics committee of the University Hospital of Ghent. Patient selection and sample collection on day 1 of the ICU stay were conducted as described in the below. Patients’ characteristics such as their initial Sequential Organ Failure Assessment (SOFA) score, site of infection, the presence of septic shock, and treatments are summarized in Appendix Table S2.

#### Patient selection

After admission to the ICU, thirteen patients were enrolled within 24 h after meeting the criteria for severe sepsis or septic shock defined at the consensus conference of 2001 (Levy *et al*, 2003) and after a signed informed consent was obtained from the patient itself or a legal representative. The experiments conformed to the principles set out in the WMA Declaration of Helsinki and the Department of Health and Human Services Belmont Report. Recently, a new consensus definition has been published, but they were published after the initiation of the study so we applied the old definition (Singer *et al*, 2016). Patients’ characteristics such as initial SOFA score, site of infection, the presence of septic shock, and treatments are summarized in Appendix Table S2.

#### Inclusion and exclusion criteria of patients with severe sepsis and clinical sample preparation

The criteria determined in the consensus conference of 2001 were followed to select the patients (Levy *et al*, 2003). Male or female patients ≥ 18 years of age were included after meeting two of following criteria of severe sepsis: hyper‐ or hypothermia (> 38°C or < 36°C); heart rhythm > 90/min; respiratory rate > 20/min; and leukocytosis or leukopenia (> 12,000/mm³ or < 4,000/mm³), in addition to a suspected or present source of infection and elevated lactate levels (> 12 mg/dl) OR urinary output < 0.5 ml/kg/h during > 2 h despite adequate fluid resuscitation OR acute lung injury with PaO_2_/FiO_2_ < 250 in the absence of pneumonia as an infection source OR acute lung injury with PaO_2_/FiO_2_ < 200 in the presence of pneumonia as an infection source OR thrombocytopenia (< 100,000/μl) OR coagulopathy (INR > 1.5). Patients with septic shock were included when they fulfilled the criteria of severe sepsis in addition to persistent hypotension despite adequate fluid resuscitation (systolic pressure < 90 mmHg or reduction of > 40 mmHg compared with baseline) OR the need for vasopressors despite adequate fluid resuscitation.

Patients were excluded after meeting the following criteria: age < 18 years, the use of immunosuppressive medication and patients with HIV, hematological malignancies, liver cirrhosis, or chronic kidney insufficiency.

From patients meeting the inclusion criteria, blood was withdrawn within 24 h of diagnosis (day 1) and the sequential organ failure (SOFA) score was determined. Blood was collected in heparin‐coated vials for biochemical analysis. Control patients had blood withdrawn at ambulatory centers. Thirteen patients and fifteen healthy controls were included in this study.

### Statistical analysis

Groups were compared with two‐way unpaired Student's *t*‐test, multiple‐group comparisons were performed using one‐ or two‐way analysis of variance (ANOVA), and the log‐rank test was used for survival tests using the Prism Software (GraphPad Software). Significance of correlation between parameters was determined via calculation of the Pearson (*r*) correlation coefficient using the GraphPad Software. Samples were assumed to be normally distributed with similar variance between groups. No randomization was used to determine experimental groups, and no blinding of the investigator was performed. Group sizes were determined on the basis of previous experience. No data were excluded from the analyses.

### Study approval

All experiments in this paper were approved by the ethical committee of the Faculty of Sciences, Ghent University.

## Author contributions

LVW conceived and performed experiments and co‐wrote the manuscript. TV, JV, KVL, JS, CW, ME, and SE performed experiments. ST analyzed RNA sequencing data. EVH and AG conceived and performed microscopy experiments. GE conceived and performed Seahorse experiments. CLS and AR conceived and performed flow cytometry experiments. CR and LV conceived and performed lipidomic experiments. LDB and JD kindly provided septic patient blood samples. PC conceived Seahorse experiments. CL conceived experiments and co‐wrote the manuscript.

## Conflict of interest

The authors declare that they have no conflict of interest.

## For more information

Author website: https://www.irc.ugent.be/index.php?id=claudeliberthome.

Surviving sepsis campaign: http://www.survivingsepsis.org/Pages/default.aspx.

**The paper explained**

Hepatic PPARα function and lipid metabolic pathways are dysregulated in polymicrobial sepsis.

**Problem**

Sepsis hits 19 million people yearly, 3 million of whom are children, with a mortality rate of approximately 25%. Due to the lack of innovative insights and therapeutics, sepsis remains a high unmet medical need. In our opinion, this lack of treatment is largely due to the fact that inflammation has been considered as the main driving and killing mechanism in sepsis. However, recent evidence points toward profound metabolic dysregulations as an alternative lethal aspect of sepsis.

**Results**

In our paper, we use the cecal ligation and puncture mouse sepsis model to investigate the metabolic alterations during sepsis. We have found that septic animals enter a starvation response, in which fat reserves are addressed and high‐energy free fatty acids, together with glycerol, are released from the white adipose tissue. In order to be useful, the energy‐rich free fatty acids have to be taken up by hepatocytes and oxidized to yield energy and ketone bodies. This metabolic oxidation (β‐oxidation) is organized in the liver mainly by one transcription factor, i.e., PPARα. In our sepsis model, we observed that PPARα loses its biological activity and that PPARα quickly declines in mRNA and protein concentration. As a result, free fatty acids (and glycerol) accumulate, leading to toxicity and cell death. Activation and inactivation of PPARα before the onset of sepsis confirm the importance of this transcription factor, and more importantly, activation of residual PPARα after the onset of sepsis can lead to a significant protection and survival. Lastly, we confirmed the acute activation of lipolysis in sepsis in humans, as free fatty acid and glycerol levels were increased in blood of septic patients compared with healthy controls.

**Impact**

These data show that liver PPARα, in addition to influencing inflammation, plays a crucial role in the control of free fatty acid oxidation during sepsis. Moreover, we have clearly demonstrated that PPARα activity drops dramatically during sepsis, causing severe metabolic alterations. We believe our findings may have a major impact on the sepsis research field and could pave the way to new therapeutic interventions in sepsis as our data using a novel PPARα agonist are promising.

## Supporting information



Appendix

Expanded View Figures PDF

Table EV1

Dataset EV1

Dataset EV2

Source Data Appendix

Review Process File

Source Data for Figure 2

## Data Availability

RNA‐seq data: Gene expression. Deposited at the National Center for Biotechnology Information Gene Expression Omnibus public database (http://www.ncbi.nlm.nih.gov/geo/) under accession number GSE139484. Lipidomics: Deposited at the MetaboLights database (https://www.ebi.ac.uk/metabolights/) under accession number MTBLS1386.
