# Supplementary material for: Hepatic PPARα function and lipid metabolic pathways are dysregulated in polymicrobial sepsis
Source: EMBO Mol Med. 2020 Jan 9;12(2):e11319. doi: 10.15252/emmm.201911319 (PMC7005534; doi:10.15252/emmm.201911319)
Supplement: Supplementary file 1 — Appendix [file EMMM-12-e11319-s001.pdf]

Appendix

Table of content

Appendix materials and methods

PPARa fl/fl mice p2

Adenoviral vectors p2

Appendix Figures

Figure S1: Gating strategy for liver hepatocytes. p3

Figure S2: Sepsis reduces activity of the hepatic β-oxidation pathway (Seahorse, all timepoints). p4

Figure S3: Plasma free fatty acids are increased after CLP (extra lipids). p5

Figure S4: Pemafibrate improves functionality of the hepatic β-oxidation pathway (all timepoints). p6

Figure S5: The liver is crucial for the pemafibrate protective effect during sepsis (preliminary data). P7

Appendix Tables

Table S1: Antibodies used for sorting of hepatocytes. P8

Table S2: Clinical characteristics of patients including in the study. P8

Table S3: Analytical reference standards that were included in the lipidomics methodology p9

Table S4: Primer sequences for genes analyzed via qPCR. p10

Appendix materials and methods

PPARa fl/fl mice

PPARa fl/fl mice were kindly provided by the lab of Karolien De Bosscher (VIB, Ghent University, Belgium) and were housed in a conventional BL2 animal facility during the experiment. During 1 week, mice were gavaged with 1 mg/kg pemafibrate on alternating days, with 4 gavages in total. On day 7, the mice underwent a sham or CLP procedure and received additional daily gavages of pemafibrate or vehicle during the lethality experiment. Mice were injected intravenously with the adenoviral vectors 72h before the cecal ligation and puncture (CLP) operation. The mice were resuscitated by intraperitoneal injection of an antibiotic cocktail containing ceftriaxone (25 mg/kg; Sigma-Aldrich NV) and metronidazole (12.5 mg/kg; Sigma-Aldrich NV) in 100 µl phosphate buffered saline (PBS) 10h and 24h after CLP onset. For organ isolation, mice were euthanized via cervical dislocation 72h after viral vector injection.

Adenoviral vectors

Control (GFP tagged) and Cre-expressing adenoviruses (pAV[Exp]-CMV>Cre) (type 5) were purchased from VectorBuilder (https://en.vectorbuilder.com/). Vectors were handled and diluted as described by the manufacturer. Mice were intravenously injected with $2x10^{10}$ viral particles.

Appendix Figures

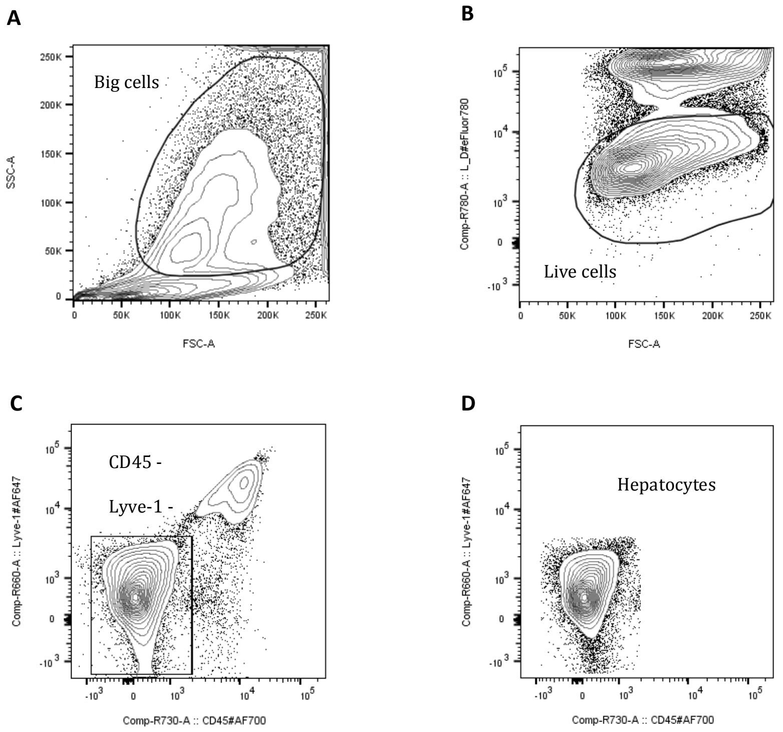

Figure S1. Gating strategy for liver hepatocytes. (A-D) Representative FACS plot showing identification of $CD45^-$ $Lyve\text{-}1^-$ hepatocytes in the liver from total single cells.

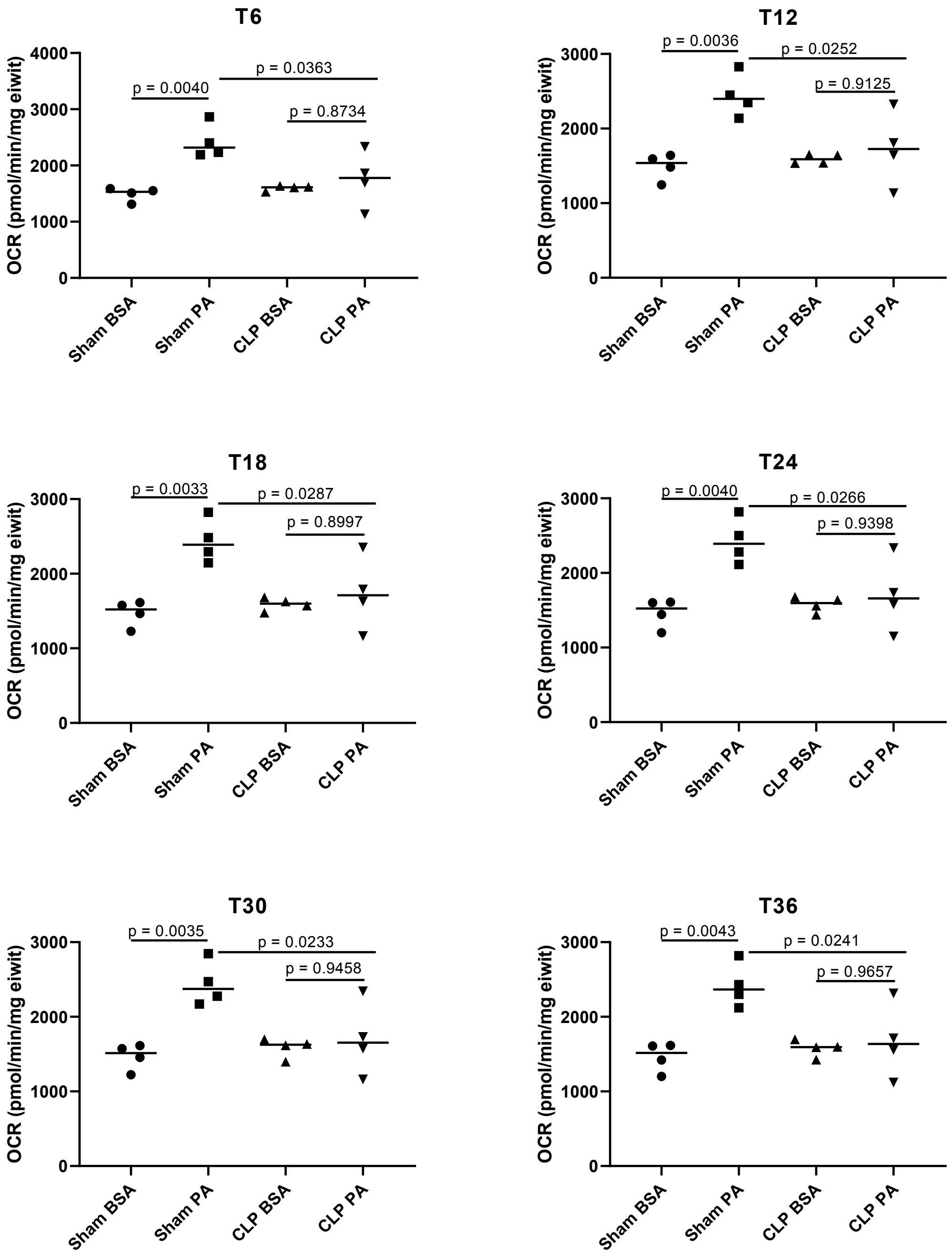

Figure S2. Sepsis reduces activity of the hepatic β-oxidation pathway (Seahorse, all timepoints). Oxygen consumption rates (OCR) of liver tissue explants 24h post sham or CLP. Liver tissue was isolated 24h post-surgery and OCR was measured via Seahorse with BSA or palmitic acid (PA) as a substrate for 42 min. n = 4/group. P-values were calculates using 2-way ANOVA analysis.

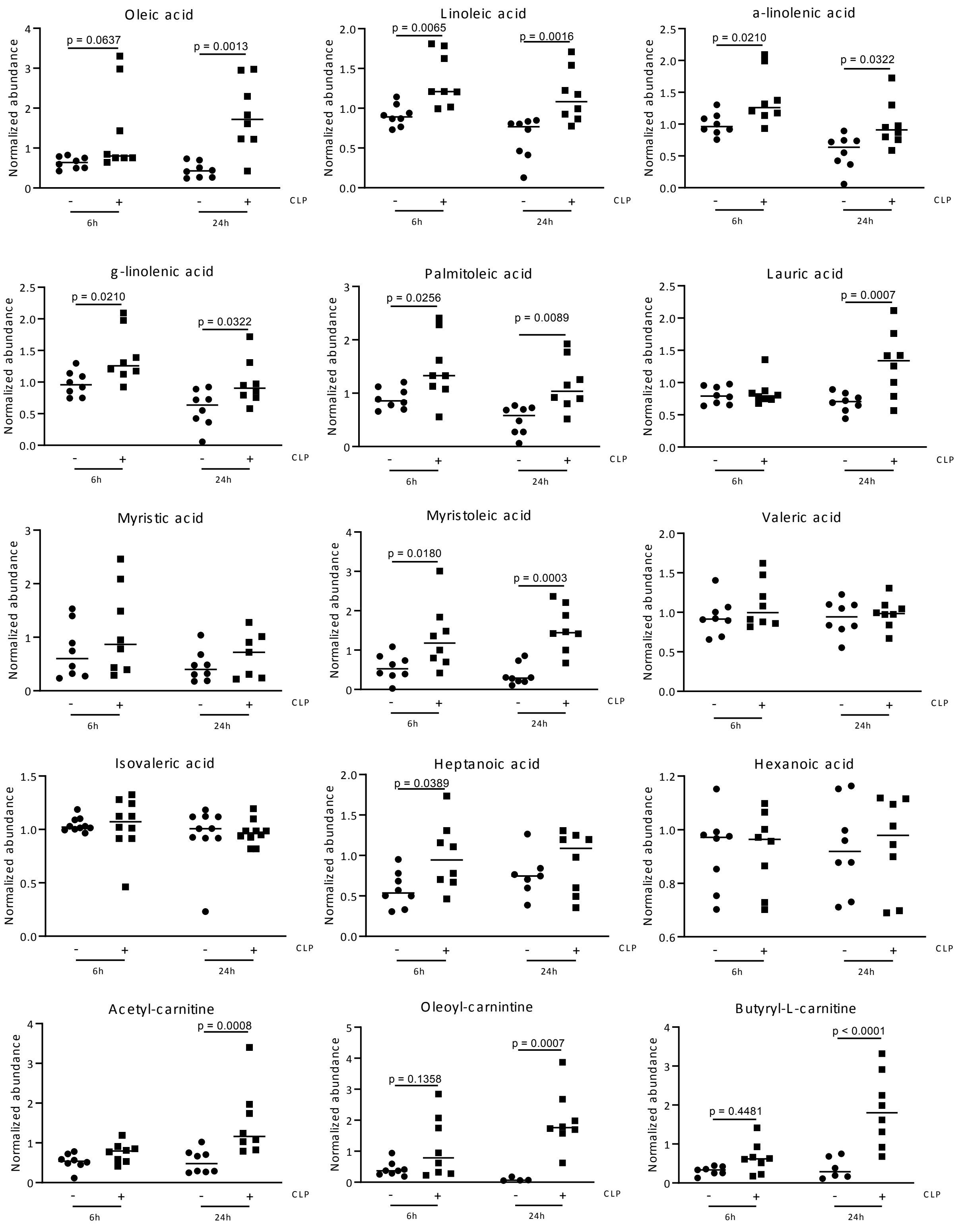

Figure S3. Plasma free fatty acids are increased after CLP (extra lipids). Mice (n= 5-7/group) underwent a sham (with or without starvation) or CLP operation and blood was collected 6h and 24h post-surgery. Plasma was isolated normalized abundances of specific lipids and fatty acids were determined via liquid chromotography-mass spectrometric lipidomics. Values were normalized to IQ values .P-values were calculated with 2-way ANOVA tests. n = 8/group.

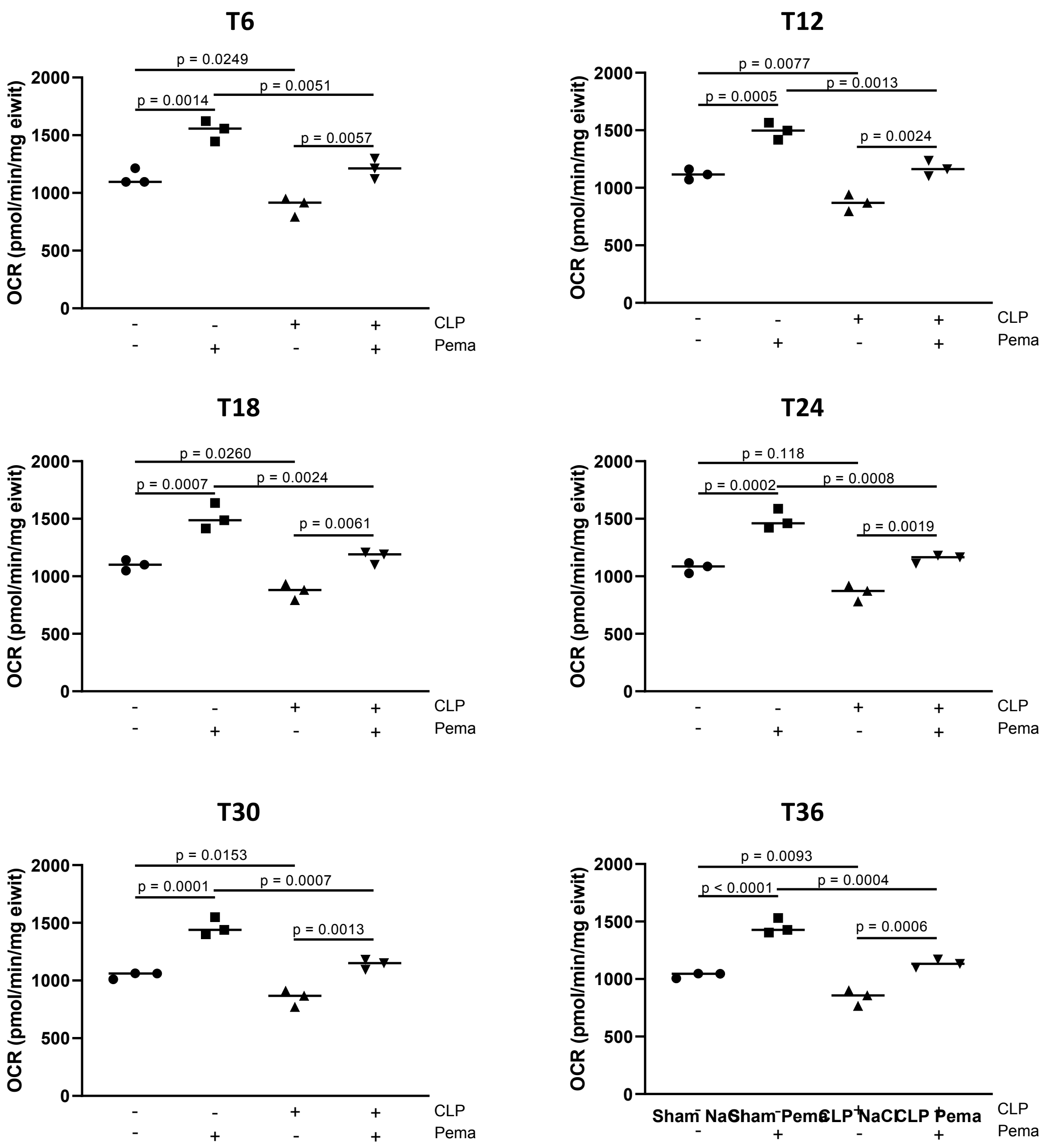

Figure S4. Pemafibrate improves functionality of the hepatic β-oxidation pathway (all timepoints). Mice were pre-treated with Pemafibrate (1mg/kg) or vehicle (0,9% NaCl) for 1 week before being subjected to CLP. Oxygen consumption rates (OCR) of liver tissue explants 24h post sham or CLP (vehicle or pemafibrate treated) after supplementation of palmitic acid (PA), measured via Seahorse. Visualization of OCR, p-values were calculates using 2-way ANOVA analysis. One experiment, n = 3/group.

A

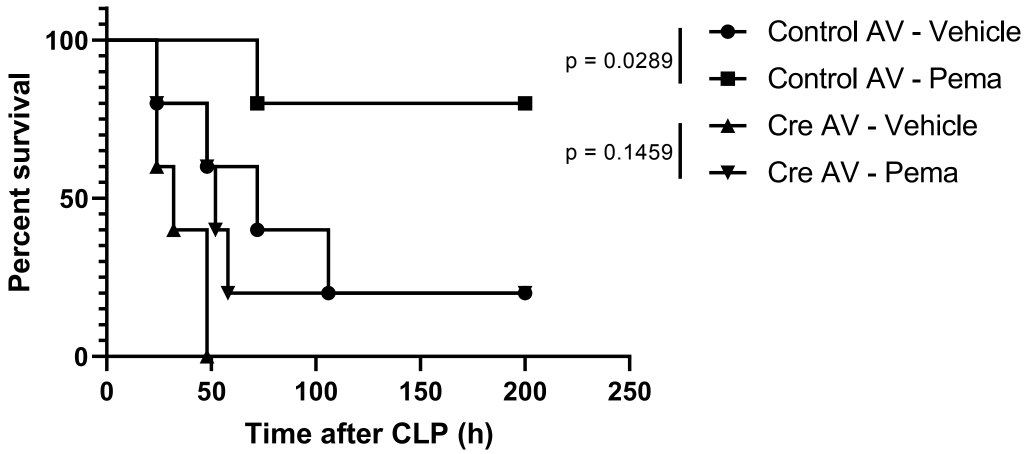

B

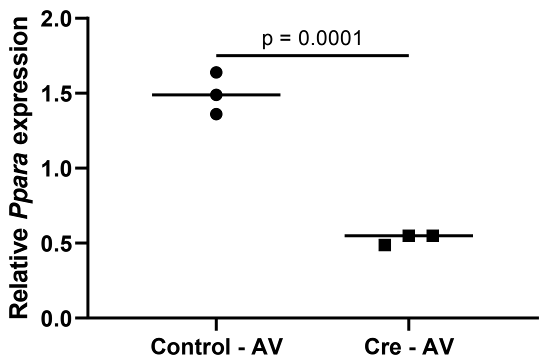

Fig S5. The liver is crucial for the pemafibrate protective effect during sepsis (preliminary data). PPARα fl/fl mice were assigned to a pemafibrate-treated group or a vehicle-treated (NaCl) group for 1 week and received an intravenous injection with either an adenoviral vector (AV) expressing Cre or a control empty viral vector 72h before sepsis (CLP) induction. **(A)** Survival of all groups was followed over-time, until no further deaths occurred. Survival curve was analyzed via Chi square tests. One experiment, n = 5/group. **(B)** In parallel, mice were sacrificed 72h post-virus injection, liver was isolated and knock-down of PPARα was assessed via qPCR expression analysis. *Ppara* expression is shown as relative expression, normalized to housekeeping genes *Hprt* and *Rpl*. P-value was calculated with a two-way Student's t-test (n = 3/group).

Appendix Tables

Table S1: Antibodies used for sorting of hepatocytes.

Antibody	Clone	Dilution	Source	Fluorochrome
Lyve-1	ALY7	1:400	eBioscience	eFluor660
CD45	30-F11	1:200	eBioscience	AF700
L/D	/	1:400	eBioscience	APC-eFluor780
FcBlock 2.4G2	2.4G2	1:200	eBioscience	/

Table S2: Clinical characteristics of patients including in the study.

Patients number	SOFA score @ time of ICU admission	Site of infection	Septic shock?	Treatment
1	12	Perforation bowel, complication after surgery (draining liver abscess)	Yes	Vancocin/Flagyl/Meronem/Ciproxine/Zyvoxid
2	8	Pancreatitis	SIRS	Tazocin
3	11	Small bowel perforation (no fecal peritonitis), 4 quadrant peritonitis (whole peritoneum infected, not local), complication after surgery	Yes	NA
4	14	Endocarditis	Yes	Meronem, Zyvoxid, Tavanic, Pentrexyl
5	10	Steatohepatitis + colon perforation	Yes	Meronem, Tazocin, Vancocin, Erythrocine, Tavanic, Eusaprim
6	6	Bilateral pneumonia	Yes	Augmentin, Biclar, Tazocin, Augmentin
7	6	Trauma by surgery, pancreatitis, necrosectomy	Yes	Meronem, Vancocin, Ciproxine
8	9	Urinary tract infection	Yes	Zyvoxid, Augmentin
9	10	Pneumonia	Severe sepsis	Tazocin, Eusaprim
10	11	Pneumonia	Yes	Cefazoline, Zinacef IV
11	7	Pneumonia	NA	Augmentin, Biclar, Solumedrol
12	13	Pneumonia	Yes	Augmentin, Biclar, Meronem, Negaban
13	6	Infection with unknown focus	Yes	Tazocin, Flagyl, Tavanic
NA: Not available				

Table S3: Analytical reference standards that were included in the lipidomics methodology, presenting compound specific information about the identity, chromatographic and mass spectrometric features.

Lipid compound	Chemical formula	m/z-value	Rt (min)
Palmitic acid	$C_{16}H_{32}O_2$	255.23295	4.53
Palmitoyl-carnitine	$C_{23}H_{45}NO_4$	400.34214	4.87
Oleic acid	$C_{18}H_{34}O_2$	281.24860	4.88
Linoleic acid	$C_{18}H_{32}O_2$	279.23295	4.43
a-Linoleic acid	$C_{18}H_{30}O_2$	277.21730	3.86
g-Linoleic acid	$C_{18}H_{30}O_2$	277.21730	3.86
Palmitoleic acid	$C_{16}H_{30}O_2$	253.21730	3.76
Lauric acid	$C_{12}H_{24}O_2$	199.17035	2.20
Myristic acid	$C_{14}H_{28}O_2$	227.20165	3.13
Myristoleic acid	$C_{14}H_{26}O_2$	225.18600	2.41
Valeric acid	$C_5H_{10}O_2$	101.05933	1.26
Isovaleric acid	$C_5H_{10}O_2$	101.06080	1.23
Heptanoic acid	$C_7H_{14}O_2$	129.09075	1.36
Hexanoic acid	$C_6H_{12}O_2$	115.07542	1.30
Acetyl-carnitine	$C_9H_{17}NO_4$	204.12303	1.27
Oleoyl-carnitine	$C_{25}H_{47}NO_4$	426.35779	5.16
Butyryl-L-carnitine	$C_{11}H_{21}NO_4$	232.15433	1.31

Table S4: Primer sequences for genes analyzed via qPCR.

Gene Name	Forward primer (5’ – 3’)	Reverse primer (3’ – 5’)
PPARa	AGAGCCCCATCTGTCCTCTC	ACTGGTAGTCTGCAAAACCAAA
Hmgcs2	GAAGAGAGCGATGCAGGAAAC	GTCCACATATTGGGCTGGAAA
Acsl1	TGCCAGAGCTGATTGACATTC	GGCATACCAGAAGGTGGTGAG
Cpt1a	CTCCGCCTGAGCCATGAAG	CACCAGTGATGATGCCATTCT
Cpt2	CAGCACAGCATCGTACCCA	TCCCAATGCCGTTCTCAAAT
Ehhadh	ATGGCTGAGTATCTGAGGCTG	GGTCCAAACTAGCTTTCTGGAG
Slc25a20	GACGAGCCGAAACCCATCAG	AGTCGGACCTTGACCGTGT
Hadha	TGCATTTGCCGCAGCTTTAC	GTTGGCCCAGATTTCGTTCA
Acox1	TAACTTCCTCACTCGAAGCCA	AGTTCCATGACCCATCTCTGTC
Hprt	AGTGTTGGATACAGGCCAGAC	CGTGATTCAAATCCCTGAAGT
Rpl	CCTGCTGCTCTCAAGGTT	TGGTTGTCACTGCCTCGTACTT
Actin	GCTTCTAGGCGGACTGTAACTGA	GCCATGCCAATGTTGTCTCTTAT
