# Supplementary material for: Hepatic PPARα function and lipid metabolic pathways are dysregulated in polymicrobial sepsis
Source: EMBO Mol Med. 2020 Jan 9;12(2):e11319. doi: 10.15252/emmm.201911319 (PMC7005534; doi:10.15252/emmm.201911319)
Supplement: Supplementary file 2 — Expanded View Figures PDF [file EMMM-12-e11319-s002.pdf]

Expanded View Figures

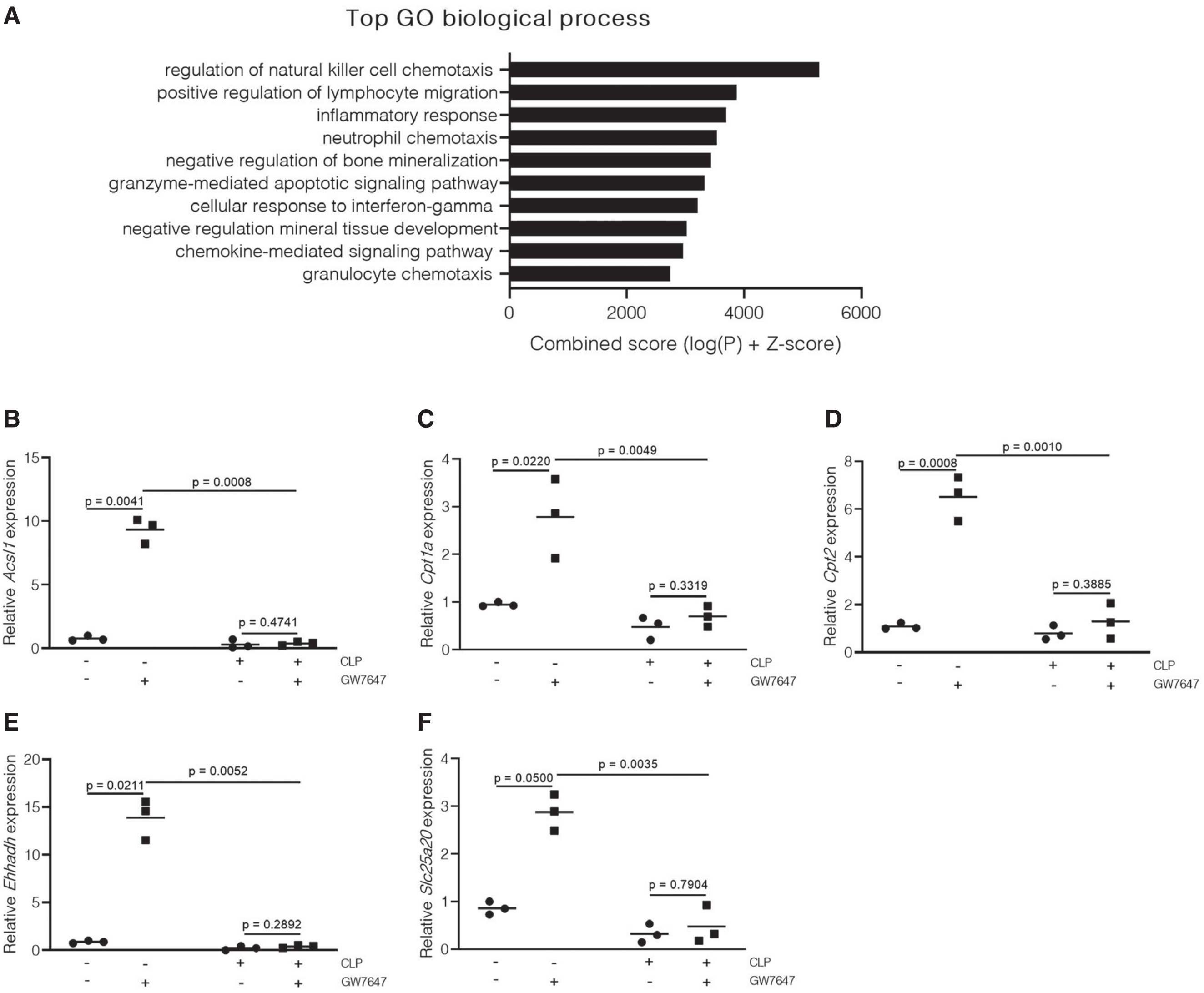

Figure EV1. Hepatic PPARα signaling is disturbed at a genome-wide level during sepsis.

A RNA-seq of liver 10 h post-sham or CLP. Mice (n = 3/group) underwent a sham or CLP operation and were injected with GW7647 (10 μg/g) 6 h post-surgery, and after 4 h (total of 10 h), livers were isolated and RNA was prepared. Top enriched gene ontology (GO) terms for genes that are specifically upregulated by GW7647 in CLP mice. Analysis was performed with the Enrichr tool.

B–F Confirmation of RNA-seq data via qPCR on pure hepatocytes isolated via flow cytometry-based sorting (n = 3/group). (B) *Acsl1*, (C) *Cpt1a*, (D) *Cpt2*, (E) *Ehhadh*, and (F) *Slc25a20* mRNA expression are shown as relative expression, normalized to housekeeping genes *Hprt* and *Rpl*. *P*-values were calculated using 2-way ANOVA analysis. Central lines represent mean.

© 2020 The Authors

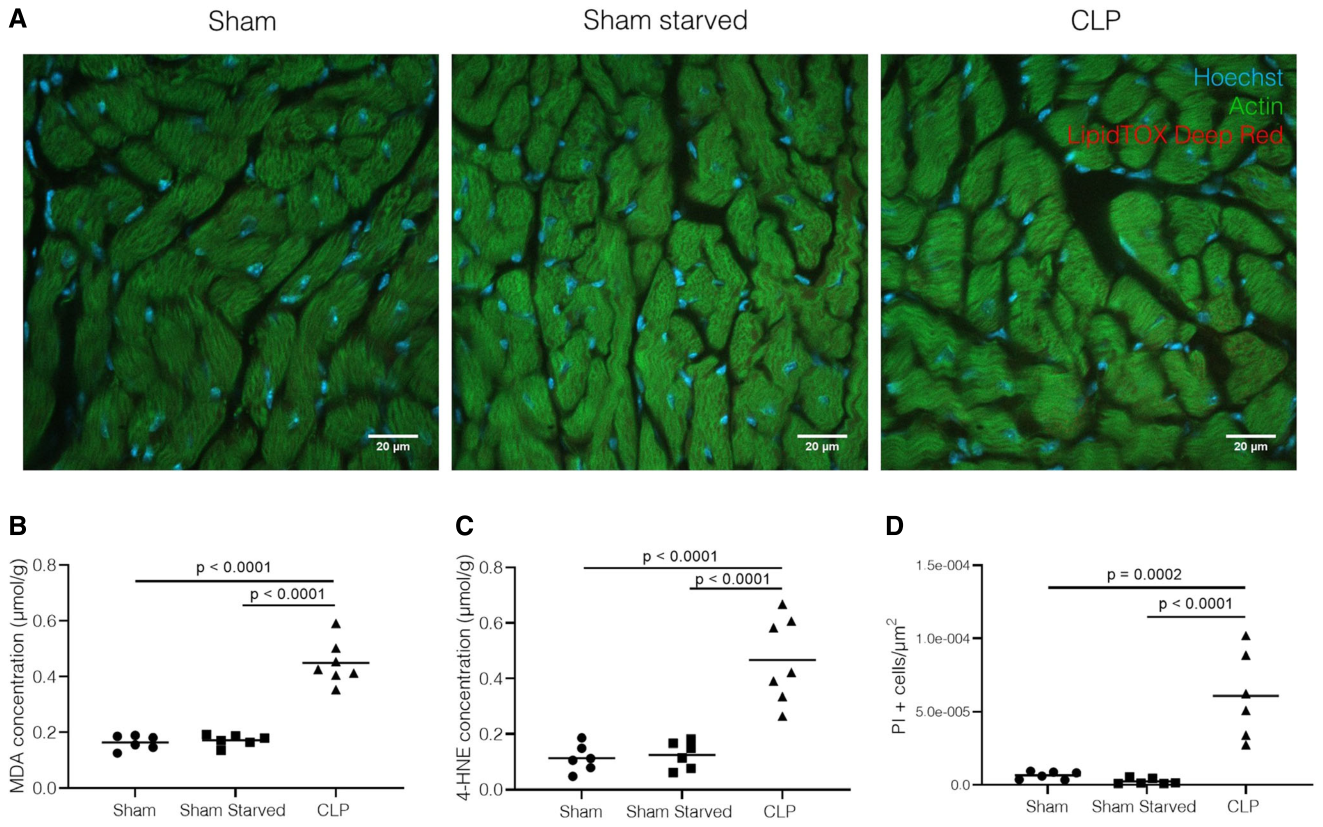

Figure EV2. No lipid accumulation in heart after sepsis, lipotoxicity in kidney after sepsis.

A Immunofluorescent images of heart 24 h after sham (with or without starvation) or CLP (n = 6–7/group, data are representative of two experiments). Cryosections were stained with Acti-stain (green), Hoechst (blue), and LipidTOX (red). Z-stacks were generated in 5–10 areas scattered across the entire tissue section. The amount of lipid droplets/cell and average size of lipid droplets (represented by voxel counts) were calculated for each Z-stack. White scale bar = 20 µm

B, C Quantification of lipid peroxidation by determination of (B) MDA and (C) 4-HNE concentrations in kidney homogenates 24 h post-surgery in sham and CLP mice as described in methods (n = 6–7/group, combined data of two experiments). *P*-value was calculated via one-way ANOVA test. Central lines represent mean.

D Apoptosis in kidney paraffin-fixated sections 24 h after sepsis, measured by TUNEL staining, and presented as % PI-positive cells/µm² tissue area. *P*-values were calculated with 1-way ANOVA tests. Combined data of two experiments, n = 6–7/group. Central lines represent mean.

Figure EV3. The PPARα agonist pemafibrate reduces mortality of sepsis by stimulating PPARα signaling and improving metabolic parameters.

Mice were pretreated with pemafibrate (1 mg/kg) or vehicle (0.9% NaCl) for 1 week before being subjected to CLP.

A Body temperature of mice 24 h post-surgery. *P*-values were calculated via 2-way Student's *t*-tests. Central lines represent mean.

B Mouse Clinical Assessment Score for Sepsis (M-CASS) for sham septic mice 24 h post-surgery. One experiment (n = 5–6/group). *P*-values were calculated via 2-way ANOVA. Central lines represent mean.

C, D Liver samples were isolated 24 h after CLP (n = 5–7/group, data are representative of two experiments), mRNA was prepared, and gene expression levels of (C) *Acsl1 and* (D) *Slc25a20* were analyzed via qPCR. Gene expression values are shown relative expression, normalized to housekeeping genes *Hprt* and *Rpl*, and *P*-values were calculated via 2-way ANOVA. Central lines represent mean.

E Immunofluorescent images of cryosections of kidney 24 h post-surgery that were stained with Acti-stain (green), Hoechst (blue), and LipidTOX (red). Z-stacks were generated in 5–10 areas scattered across the entire tissue section. White scale bar = 20µm.

F The amount of lipid droplets (LDs)/cell and average size of LDs (represented by voxel counts) were calculated for each Z-stack. Averages of the amount and size of lipid droplets were converged for each mouse, and biological replicates are depicted in the table as mean ± SEM. *P*-values were calculated using unpaired *t*-tests. n = 6/group, combined data of two experiments.

G Blood was isolated 24 h post-surgery, and plasma aspartate aminotransferase (AST) levels were determined as described in Materials and Methods. *P*-values were calculated with 2-way ANOVA tests. n = 6–7/group, combined data of two independent experiments. Central lines represent mean.

H–J Bacterial load was determined in (H) liver, (I) kidney, and (J) lung tissue homogenates 24 h post-sepsis. Values are shown as CFU/mg tissue. *P*-values were calculated using 2-way Student's *t*-tests. One experiment, n = 5–6 mice/group. Central lines represent mean.

 © 2020 The Authors

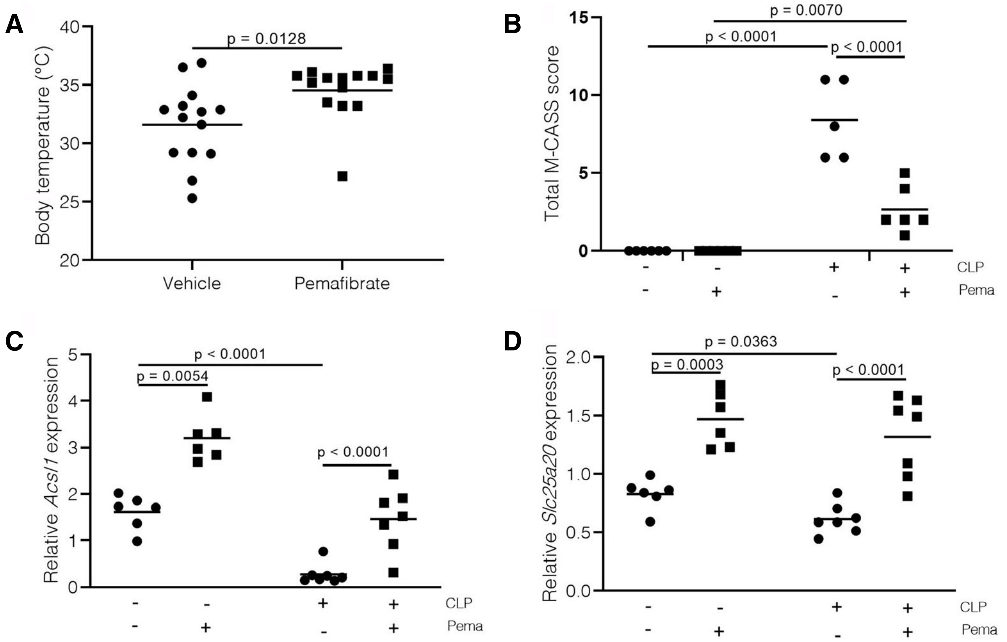

E

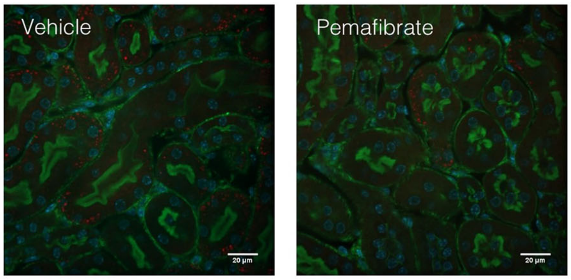

F

Kidney	#LDs/cell	#voxels/LD
Vehicle	7.70 ± 3.09	308.99 ± 62.54
Pemafibrate	2.71 ± 0.86	216.46 ± 33.62
P-value	0.0059	0.0153

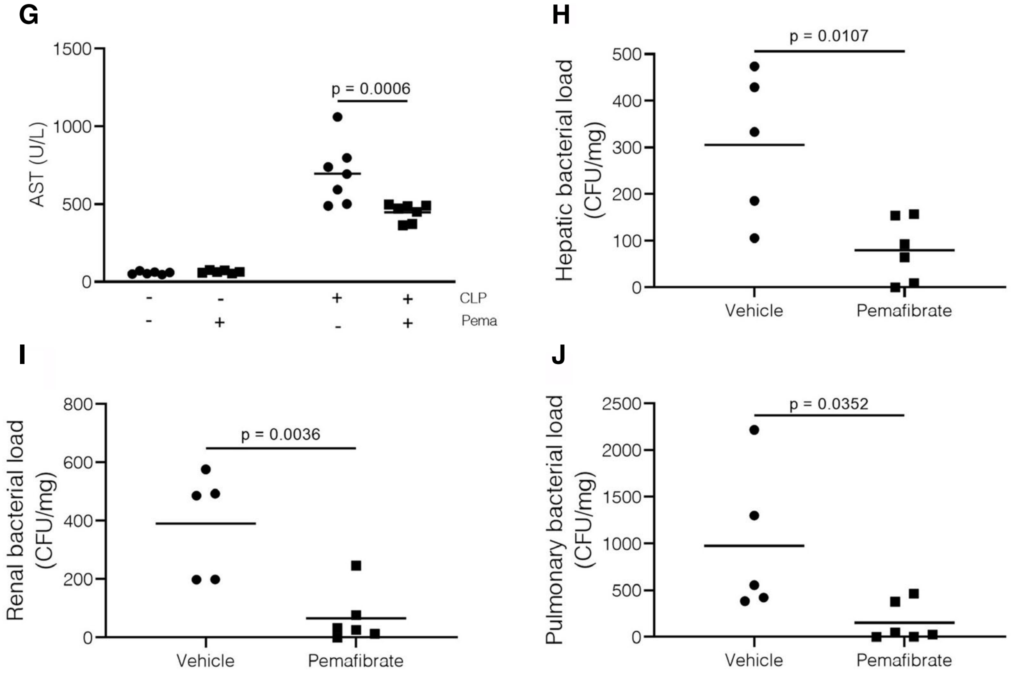

Figure EV3.

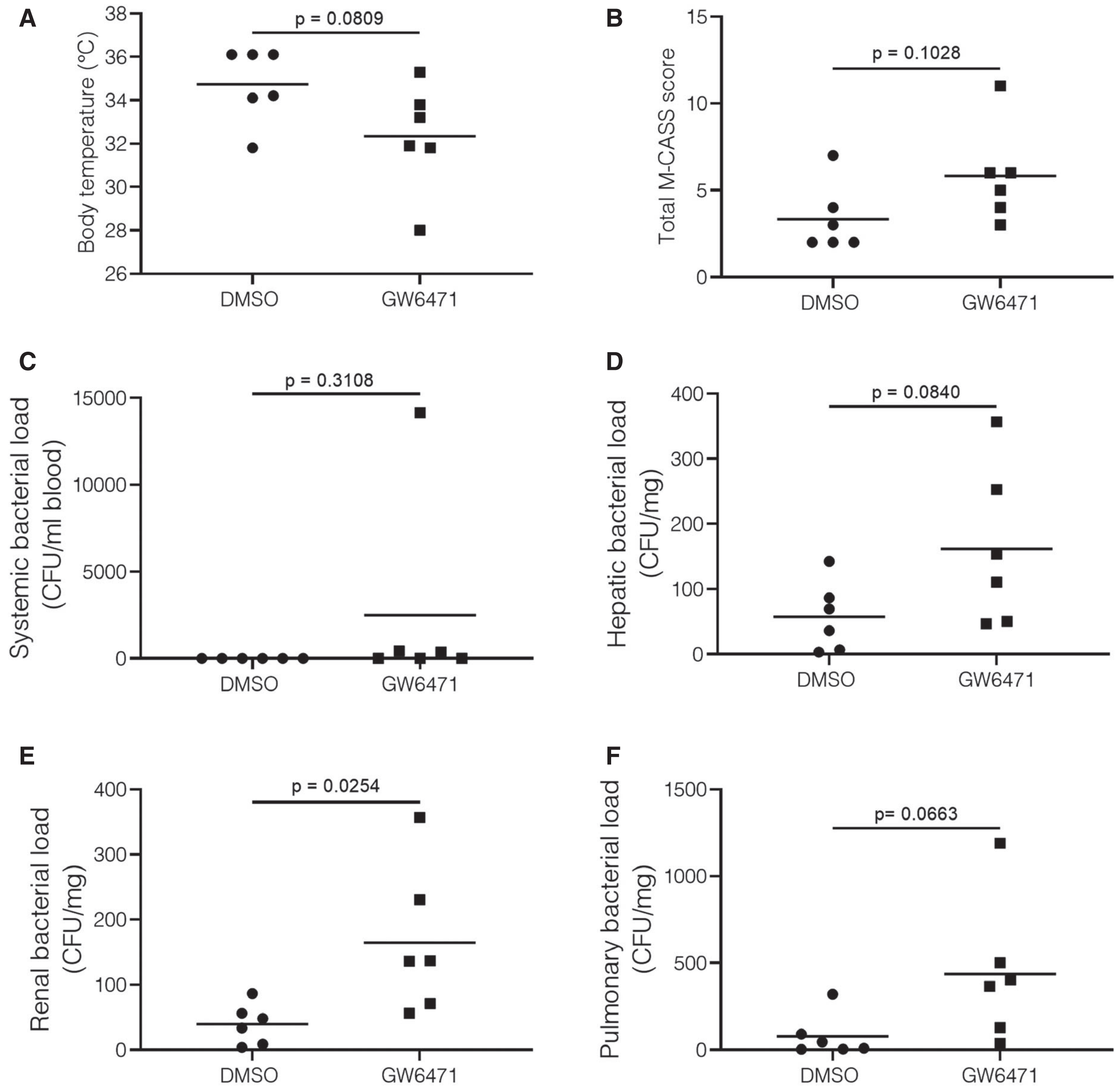

Figure EV4. GW6471 treatment worsens septic disease parameters and bacterial load in blood and organs.

Mice were injected with the PPARα antagonist GW6471 (10 μg/g) or vehicle (DMSO) 3 h pre-CLP and 3 h post-CLP, 24 h post-CLP blood, and organs were isolated (n = 5-6/group). One experiment.

A Body temperature of mice 24 h post-surgery. Central lines represent mean.
B Mouse Clinical Assessment Score for Sepsis (M-CASS) for septic mice 24 h post-surgery. Central lines represent mean.
C–F Bacterial load in (C) blood and (D) liver, (E) kidney, and (F) lung tissue homogenates of septic mice. Values are shown as CFU/mg tissue. Central lines represent mean.

Data information: *P*-values were calculated using two-way Student's *t*-tests.

© 2020 The Authors
