# Supplementary material for: Hepatic PPARα function and lipid metabolic pathways are dysregulated in polymicrobial sepsis
Source: EMBO Mol Med. 2020 Jan 9;12(2):e11319. doi: 10.15252/emmm.201911319 (PMC7005534; doi:10.15252/emmm.201911319)
Supplement: Supplementary file 5 — Dataset EV2 [file EMMM-12-e11319-s005.pdf]

Dataset EV2: List of genes downregulated by CLP. CLP1 = 6h after CLP, CLP2 = 8h after CLP, CLP 3 = 10h after CLP. Ensemble code, gene symbol, log fold change (LFC), and false discovery rate (FDR) are shown. A link to Pubmed for literature search is also provided.

		CLP1		CLP2		CLP3		
Ensg	Symbol	LFC	FDR	LFC	FDR	LFC	FDR	Link
ENSMUSG00000000056	Narf	-0.713	1.5317e-02	-1.184	1.1348e-03	-1.026	8.4286e-04	pubmed
ENSMUSG00000000058	Cav2	-1.317	2.1389e-04	-0.996	1.4266e-03	-0.850	6.0654e-02	pubmed
ENSMUSG00000000126	Wnt9a	-1.751	1.5636e-02	-2.762	8.0731e-03	-0.061	9.6240e-01	pubmed
ENSMUSG00000000142	Axin2	-2.372	8.9451e-14	-2.070	4.7987e-06	-1.550	3.5111e-03	pubmed
ENSMUSG00000000148	Brat1	-0.670	5.1405e-04	-1.444	1.6532e-03	-0.548	1.9573e-01	pubmed
ENSMUSG00000000149	Gna12	-1.363	9.9733e-25	-1.446	1.7172e-17	-0.604	2.5902e-03	pubmed
ENSMUSG00000000325	Arvcf	-2.514	4.2397e-20	-0.989	1.4420e-02	0.042	9.6700e-01	pubmed
ENSMUSG00000000686	Abhd15	-2.690	7.6877e-29	-2.005	1.0700e-07	-1.891	8.8964e-05	pubmed
ENSMUSG00000000693	Loxl3	-1.278	2.2040e-03	-1.010	1.9959e-01	-0.279	7.1815e-01	pubmed
ENSMUSG00000000881	Dlg3	-1.208	3.3037e-09	-1.199	4.7987e-06	-0.915	1.0084e-02	pubmed
ENSMUSG00000001138	Cnnm3	-1.084	1.3942e-08	-1.250	2.4817e-06	-0.493	5.1324e-02	pubmed
ENSMUSG00000001143	Lman2l	-0.248	3.5547e-01	-1.299	1.5118e-05	-0.795	1.0910e-02	pubmed
ENSMUSG00000001240	Ramp2	-1.212	2.8362e-05	-0.490	4.3319e-01	-0.477	2.1630e-01	pubmed
ENSMUSG00000001270	Ckb	-1.520	1.0365e-04	-2.289	2.1572e-04	-1.174	1.5361e-01	pubmed
ENSMUSG00000001288	Rarg	-1.754	9.8708e-11	-2.002	2.5125e-08	-1.455	2.5305e-02	pubmed
ENSMUSG00000001482	Def8	-1.301	8.0814e-12	-1.122	8.2722e-10	-0.657	4.5843e-02	pubmed
ENSMUSG00000001672	Marveld3	-1.596	9.2554e-04	-2.124	6.4556e-04	-0.757	5.0940e-01	pubmed
ENSMUSG00000001763	Tspan33	-1.122	1.8006e-05	-1.468	3.7381e-05	-1.034	3.6181e-03	pubmed
ENSMUSG00000001802	Lrp3	-0.656	1.0297e-02	-1.049	6.8527e-07	-0.668	1.9093e-02	pubmed
ENSMUSG00000001942	Siae	-0.688	5.1441e-04	-1.190	1.5989e-04	-1.163	1.3870e-06	pubmed
ENSMUSG00000002032	Tmem25	-4.520	1.2426e-56	-4.440	1.5824e-19	-4.086	5.4436e-10	pubmed
ENSMUSG00000002083	Bbc3	-0.510	2.4859e-01	-1.848	2.3041e-04	-0.808	2.8411e-01	pubmed
ENSMUSG00000002103	Acp2	-0.950	3.7456e-12	-1.312	3.6507e-09	-0.962	8.6747e-03	pubmed
ENSMUSG00000002109	Ddb2	-1.127	8.1112e-04	-1.565	9.7870e-03	-0.648	1.8222e-01	pubmed
ENSMUSG00000002227	Mov10	0.836	2.9338e-03	-1.059	4.3407e-04	-0.381	2.3697e-01	pubmed
ENSMUSG00000002265	Peg3	-2.010	6.8572e-10	-1.752	2.4493e-05	-1.111	9.0095e-02	pubmed
ENSMUSG00000002308	Cd320	-1.011	6.7474e-03	-1.786	5.5725e-03	-0.916	1.5216e-01	pubmed
ENSMUSG00000002346	Slc25a42	-1.488	3.2498e-11	-1.623	1.6236e-10	-0.763	1.9992e-02	pubmed
ENSMUSG00000002393	Nr2f6	-1.028	8.2437e-08	-1.715	9.7401e-10	-0.908	1.4732e-02	pubmed
ENSMUSG00000002396	Ocel1	-1.552	1.3078e-23	-1.329	4.2383e-06	-0.831	1.1637e-01	pubmed
ENSMUSG00000002409	Dyrk1b	-0.902	2.2172e-04	-1.516	1.1324e-04	-0.350	5.5628e-01	pubmed
ENSMUSG00000002504	Slc9a3r2	-1.300	6.9212e-11	-1.193	1.8673e-02	-0.904	3.8395e-02	pubmed
ENSMUSG00000002661	Alkbh7	-0.745	1.8179e-02	-1.235	9.9007e-04	-0.835	5.5704e-02	pubmed
ENSMUSG00000002668	Dennd1c	0.119	7.8593e-01	-1.287	3.0867e-03	-0.382	4.9992e-01	pubmed
ENSMUSG00000002763	Pex6	-0.604	1.0773e-07	-1.081	2.1589e-08	-0.958	1.6207e-03	pubmed
ENSMUSG00000002781	Tmem143	-0.882	3.6055e-06	-1.386	7.3264e-05	-0.850	5.1236e-02	pubmed
ENSMUSG00000002803	Btbd6	-0.991	2.5202e-06	-1.406	4.4594e-09	-0.715	2.8713e-02	pubmed
ENSMUSG00000002871	Tpra1	-1.004	2.6766e-11	-0.927	2.3214e-02	-0.608	9.5907e-02	pubmed
ENSMUSG00000002908	Kcnn1	-1.906	1.5188e-07	-1.733	1.5636e-03	-1.519	3.2679e-03	pubmed
ENSMUSG00000002992	Apoc2	-4.466	5.9347e-07	-2.389	5.7313e-02	-2.673	2.7682e-02	pubmed
ENSMUSG00000003123	Lipe	-2.101	7.7611e-23	-2.026	9.4848e-06	-1.166	4.5887e-02	pubmed
ENSMUSG00000003134	Tbc1d8	-0.549	2.9196e-02	-1.039	1.9234e-04	-0.953	2.4694e-02	pubmed
ENSMUSG00000003190	Bcl2l12	-1.330	6.0008e-03	-1.735	5.2833e-02	-1.537	1.2887e-02	pubmed
ENSMUSG00000003273	Car11	-4.362	3.5175e-03	-1.078	1	-1.012	1	pubmed
ENSMUSG00000003344	Btbd2	-0.797	6.9883e-05	-1.434	1.5828e-10	-0.646	6.4517e-03	pubmed
ENSMUSG00000003526	Prodh	-1.195	4.5053e-12	-0.590	8.3412e-02	-0.644	3.0621e-02	pubmed
ENSMUSG00000003555	Cyp17a1	-1.654	1.4093e-02	-3.523	3.1163e-09	-2.434	1	pubmed
ENSMUSG00000003585	Sec14l2	-1.524	1.1571e-18	-1.544	4.2791e-07	-1.262	1.6115e-03	pubmed
ENSMUSG00000003623	Crot	-0.510	1.7754e-01	-0.491	2.7096e-01	-1.404	8.4286e-04	pubmed
ENSMUSG00000003644	Rps6ka1	-1.229	1.2748e-09	-1.342	4.1552e-04	-0.225	5.8634e-01	pubmed
ENSMUSG00000003948	Mmd	-1.407	4.9692e-07	-1.306	3.7532e-09	-1.854	2.8034e-13	pubmed
ENSMUSG00000003949	Hlf	-1.911	4.2944e-08	-1.375	9.4770e-13	-0.896	8.6943e-03	pubmed
ENSMUSG00000004044	Cavin1	-1.110	3.8217e-05	-0.951	1.0300e-02	-0.696	2.5348e-01	pubmed
ENSMUSG00000004105	Angptl2	-2.012	2.0999e-13	-0.817	3.6067e-01	-1.250	1.2651e-01	pubmed
ENSMUSG00000004151	Etv1	-2.258	6.9827e-03	-0.758	4.0263e-01	-0.737	4.7997e-01	pubmed
ENSMUSG00000004187	Kifc2	-1.490	9.0884e-07	-0.601	4.4988e-01	-1.215	3.8772e-02	pubmed
ENSMUSG00000004561	Mettl17	-0.888	2.6188e-05	-1.639	8.8361e-04	-0.581	1.8806e-01	pubmed
ENSMUSG00000004562	Arhgef40	-0.711	1.5224e-04	-1.532	1.1489e-04	-0.545	1.3028e-01	pubmed
ENSMUSG00000004633	Chn2	-1.008	7.8485e-05	-0.997	4.5271e-05	-1.189	1.3870e-06	pubmed
ENSMUSG00000004748	Mtfp1	-1.516	3.7257e-06	-2.208	1.8488e-07	-1.561	1.5508e-05	pubmed
ENSMUSG00000004815	Dgkq	-0.629	2.8071e-06	-1.037	5.1540e-03	-0.255	5.6300e-01	pubmed
ENSMUSG00000005089	Slc1a2	-0.460	3.7840e-02	-0.557	1.8742e-02	-1.103	1.6298e-03	pubmed
ENSMUSG00000005107	Slc2a9	-1.017	1.5194e-05	-1.001	1.3682e-03	-0.805	2.3154e-03	pubmed
ENSMUSG00000005501	Usp40	-1.157	3.8935e-20	-1.143	1.5884e-03	-0.904	3.7029e-02	pubmed
ENSMUSG00000005514	Por	-0.562	4.0198e-02	-0.672	3.5199e-02	-1.024	9.4629e-03	pubmed
ENSMUSG00000005674	Tomm40l	-1.804	3.0271e-07	-0.936	4.5565e-02	-1.249	9.0199e-04	pubmed
ENSMUSG00000005677	Nr1i3	-3.156	1.4285e-13	-2.259	1.5596e-03	-2.340	3.2780e-06	pubmed
ENSMUSG00000005699	Pard6a	-1.243	5.7269e-03	-0.821	3.3470e-01	-0.445	6.0182e-01	pubmed
ENSMUSG00000005718	Tfap4	-2.338	2.4425e-06	-1.746	5.1758e-03	-0.399	7.3088e-01	pubmed
ENSMUSG00000005907	Pex1	-1.102	8.9022e-13	-0.758	4.3662e-05	-0.398	8.3329e-02	pubmed
ENSMUSG00000005968	Tuft1	-0.245	5.8262e-01	-1.061	2.5307e-03	-0.408	2.4470e-01	pubmed
ENSMUSG00000005982	Naa60	-1.184	6.6437e-15	-1.328	1.0185e-12	-1.096	4.5070e-05	pubmed
ENSMUSG00000006218	Fam131c	-1.185	4.6207e-02	-1.992	9.6121e-04	-1.886	1.1740e-02	pubmed
ENSMUSG00000006235	Epor	-2.914	5.9649e-03	-2.636	9.5204e-02	-0.651	1	pubmed
ENSMUSG00000006344	Ggt5	-1.353	1.3746e-05	-1.007	5.2826e-02	-0.818	1.6641e-01	pubmed
ENSMUSG00000006362	Cbfa2t3	-2.435	7.4211e-11	-1.179	2.2995e-02	-0.925	9.2981e-02	pubmed
ENSMUSG00000006386	Tek	-1.950	4.0994e-09	-1.031	1.5248e-02	-1.020	1.1276e-02	pubmed
ENSMUSG00000006567	Atp7b	-1.137	1.4040e-10	-1.079	4.1436e-04	-0.413	3.2737e-01	pubmed
ENSMUSG00000006641	Slc5a6	-1.912	6.8732e-18	-1.690	1.5804e-07	-0.416	4.3049e-01	pubmed
ENSMUSG00000006711	D130043K22Rik	-2.364	2.5184e-04	-2.185	2.4219e-03	-0.871	3.3645e-01	pubmed
ENSMUSG00000006715	Gmnn	-1.323	3.6301e-05	-1.161	4.7140e-02	0.157	7.4597e-01	pubmed
ENSMUSG00000006958	Chrd	-1.118	4.6237e-03	-0.961	7.9464e-03	-0.822	3.4141e-02	pubmed
ENSMUSG00000007097	Atp1a2	-1.627	6.5665e-03	-1.056	4.1772e-01	-0.507	6.9527e-01	pubmed
ENSMUSG00000007338	Mrpl49	-0.666	2.9965e-05	-1.148	9.8328e-06	0.044	9.4466e-01	pubmed
ENSMUSG00000007379	Dennd2c	-2.196	1.7127e-03	-0.128	9.3477e-01	-0.054	9.6178e-01	pubmed
ENSMUSG00000007827	Ankrd26	-1.384	7.8637e-07	-0.972	4.4287e-02	-0.490	2.7725e-01	pubmed
ENSMUSG00000007837	Prrg2	-0.828	3.3368e-05	-1.213	2.2685e-09	-0.672	1.4461e-02	pubmed
ENSMUSG00000008153	Clstn3	-3.992	9.1871e-10	-3.421	2.1067e-04	-1.709	1.0841e-01	pubmed
ENSMUSG00000008398	Elk3	-2.067	2.2108e-16	-1.885	1.0259e-08	-0.722	3.6076e-01	pubmed
ENSMUSG00000008822	Acyp1	-0.549	7.4574e-02	-0.867	4.5007e-02	-1.742	2.3121e-05	pubmed
ENSMUSG00000008845	Cd163	-2.255	4.9124e-64	-0.897	7.7090e-02	-0.873	9.3810e-02	pubmed

		CLP1		CLP2		CLP3		
Ensg	Symbol	LFC	FDR	LFC	FDR	LFC	FDR	Link
ENSMUSG00000009108	Gnat2	-1.287	4.0347e-02	-1.668	1.9918e-04	-1.320	4.0062e-03	pubmed
ENSMUSG00000009145	Dqx1	-2.082	6.2415e-16	-2.270	1.2889e-05	-1.116	5.5269e-02	pubmed
ENSMUSG00000009376	Met	-1.347	2.8135e-11	-1.358	1.2927e-21	-0.925	2.2700e-03	pubmed
ENSMUSG00000009378	Slc16a12	-1.489	1.9822e-04	-1.690	8.6764e-04	-1.973	2.3105e-06	pubmed
ENSMUSG00000009621	Vav2	-1.016	5.2221e-16	-1.491	2.1857e-19	-0.554	8.2458e-02	pubmed
ENSMUSG00000009633	G0s2	-4.717	1.5582e-21	-3.421	6.3978e-08	-0.983	4.8803e-01	pubmed
ENSMUSG00000009654	Oit3	-1.164	2.2873e-07	-0.933	8.5815e-05	-0.534	7.7177e-03	pubmed
ENSMUSG00000009772	Nuak2	-1.152	5.3581e-06	-2.062	1.8682e-12	-1.629	1.2561e-06	pubmed
ENSMUSG00000009995	Taz	-1.003	3.1744e-14	-1.195	2.1069e-07	-0.895	1.5928e-02	pubmed
ENSMUSG00000010047	Hyal2	-1.270	1.5260e-23	-0.871	1.5814e-02	-0.783	2.7597e-02	pubmed
ENSMUSG00000010051	Hyal1	-1.477	2.3140e-05	-1.032	5.1550e-06	-0.895	6.9608e-03	pubmed
ENSMUSG00000010057	Nprl2	-0.728	7.6107e-05	-1.161	4.4111e-04	-0.913	7.2763e-03	pubmed
ENSMUSG00000010307	Tmem86a	-1.267	4.0098e-08	-1.828	3.0130e-05	-1.028	7.8105e-03	pubmed
ENSMUSG00000010492	Uckl1os	-1.860	8.6913e-03	-1.358	4.0615e-01	-0.465	8.1224e-01	pubmed
ENSMUSG00000010601	Apol7a	-1.505	2.1377e-05	-1.919	6.2792e-06	-1.821	6.5308e-07	pubmed
ENSMUSG00000010797	Wnt2	-3.399	3.8181e-09	-1.818	4.4296e-02	-1.243	2.7853e-02	pubmed
ENSMUSG00000011263	Exoc3l2	-2.975	2.0938e-14	-0.946	3.2130e-01	-0.751	3.0526e-01	pubmed
ENSMUSG00000011382	Dhdh	-1.294	8.5936e-11	-1.696	1.1623e-07	-1.510	9.8413e-08	pubmed
ENSMUSG00000012123	Crybg2	-1.677	6.0771e-04	-0.446	6.0254e-01	0.763	4.9614e-01	pubmed
ENSMUSG00000013033	Adgrl1	-2.110	1.6940e-09	-1.642	8.0650e-03	-0.353	6.1028e-01	pubmed
ENSMUSG00000013236	Ptprs	-1.327	1.4149e-04	-1.051	1.6464e-01	-0.528	3.8321e-01	pubmed
ENSMUSG00000013495	Tmem175	-1.397	3.7752e-09	-1.061	2.7710e-02	-0.873	6.7305e-02	pubmed
ENSMUSG00000013539	Tango2	-1.628	1.5511e-24	-1.339	4.0425e-11	-0.996	7.5597e-10	pubmed
ENSMUSG00000014164	Klhl3	-1.929	3.3796e-13	-1.620	7.9218e-05	-1.195	1.2221e-02	pubmed
ENSMUSG00000014303	Glis2	-1.009	6.2567e-03	-0.314	7.0763e-01	0.330	6.7063e-01	pubmed
ENSMUSG00000014361	Mertk	-1.191	3.5319e-05	-0.877	3.6069e-03	-0.684	1.1837e-02	pubmed
ENSMUSG00000014633	Cmc2	-1.080	6.8826e-05	-1.001	5.2390e-03	-0.603	5.8427e-02	pubmed
ENSMUSG00000014837	4931428F04Rik	-1.891	4.6237e-03	-1.084	2.5120e-01	-0.201	8.3189e-01	pubmed
ENSMUSG00000014850	Msh3	-1.050	3.7610e-04	-1.560	1.7435e-08	-1.069	7.6367e-03	pubmed
ENSMUSG00000015599	Ttbk1	-2.171	5.4691e-05	-0.927	4.3056e-01	-0.543	6.3758e-01	pubmed
ENSMUSG00000015776	Med22	-1.370	1.0205e-12	-1.626	7.2540e-09	-1.085	2.9626e-03	pubmed
ENSMUSG00000015843	Rxrg	-1.605	1.7098e-04	-0.979	7.6257e-02	-0.042	9.5851e-01	pubmed
ENSMUSG00000015854	Cd5l	-0.514	1.3583e-02	-0.310	3.2863e-01	-1.132	7.1692e-04	pubmed
ENSMUSG00000015869	Prpsap1	-0.750	1.2341e-09	-1.199	5.4315e-10	-0.917	3.7117e-03	pubmed
ENSMUSG00000015944	Castor2	-1.237	6.4903e-05	-0.667	2.1301e-01	0.078	8.9978e-01	pubmed
ENSMUSG00000015966	Il17rb	-1.176	6.5688e-05	-1.836	7.5769e-06	-0.934	4.3379e-02	pubmed
ENSMUSG00000015970	Chdh	-1.125	2.0454e-13	-1.432	3.2136e-12	-1.237	3.8970e-03	pubmed
ENSMUSG00000016028	Celsr1	-3.549	2.1273e-23	-3.401	1.5671e-21	-1.099	4.6722e-01	pubmed
ENSMUSG00000016128	Stard13	-1.254	3.0927e-13	-0.703	1.0431e-02	-0.887	3.1900e-03	pubmed
ENSMUSG00000016510	Mtif3	-1.021	1.9584e-09	-1.000	1.7222e-03	-0.447	2.5934e-01	pubmed
ENSMUSG00000016757	Ttll12	-0.203	5.1754e-01	-1.081	4.6569e-03	-0.834	3.4605e-02	pubmed
ENSMUSG00000016758	Bik	-1.693	8.6539e-07	-0.861	1.0428e-01	-0.435	4.3390e-01	pubmed
ENSMUSG00000016933	Plcg1	-1.399	1.1293e-13	-1.381	3.3187e-15	-0.406	2.8134e-01	pubmed
ENSMUSG00000016940	Kctd2	-1.159	5.7709e-08	-1.175	4.2459e-06	-0.980	1.6353e-04	pubmed
ENSMUSG00000017146	Brca1	-2.622	1.4339e-08	-2.322	8.3385e-08	-1.277	4.3379e-02	pubmed
ENSMUSG00000017309	Cd300lg	-1.498	3.3923e-05	-0.796	1.0450e-01	-1.308	5.8297e-03	pubmed
ENSMUSG00000017376	Nlk	-0.518	4.7905e-03	-1.226	6.3155e-03	-1.029	2.5117e-03	pubmed
ENSMUSG00000017386	Traf4	-1.211	1.6882e-14	-1.088	1.9409e-04	-1.026	1.1846e-03	pubmed
ENSMUSG00000017466	Timp2	-1.111	8.3232e-06	-0.329	5.5541e-01	-0.133	8.1351e-01	pubmed
ENSMUSG00000017607	Tns4	-3.713	3.5216e-03	-0.757	6.5442e-01	-1.414	1	pubmed
ENSMUSG00000017639	Rab11fip4	-2.471	2.7093e-34	-2.519	4.7193e-10	-1.769	2.6474e-03	pubmed
ENSMUSG00000017868	Sgk2	-1.818	5.3253e-04	-3.051	1.7301e-08	-2.582	1.4038e-07	pubmed
ENSMUSG00000018166	Erbb3	-2.077	3.8878e-19	-2.603	2.3673e-14	-2.336	1.4178e-02	pubmed
ENSMUSG00000018169	Mfng	-1.223	9.6294e-03	-1.316	1.0090e-01	-0.026	9.8678e-01	pubmed
ENSMUSG00000018411	Mapt	-3.166	1.5795e-09	-2.466	4.8340e-03	-1.214	1.9595e-01	pubmed
ENSMUSG00000018474	Chd3	-1.186	4.3096e-34	-0.677	6.3037e-06	-0.462	2.2211e-01	pubmed
ENSMUSG00000018547	Pip4k2b	-0.812	9.1741e-06	-1.581	4.6952e-20	-0.667	6.1025e-03	pubmed
ENSMUSG00000018604	Tbx3	-2.392	8.6444e-40	-2.233	2.1617e-18	-1.782	1.2832e-07	pubmed
ENSMUSG00000018727	Cpsf4l	-2.787	4.0416e-12	-2.787	1.3897e-06	-1.900	3.0485e-03	pubmed
ENSMUSG00000018740	Slc25a35	-3.726	1.7724e-04	-3.214	8.1585e-02	-1.167	3.7385e-01	pubmed
ENSMUSG00000018750	Zbtb4	-1.080	2.0148e-06	-1.238	3.0540e-05	-0.742	1.1058e-01	pubmed
ENSMUSG00000018796	Acsl1	-1.222	4.4790e-03	-0.254	7.3188e-01	-0.875	1.3152e-02	pubmed
ENSMUSG00000018809	Smyd4	-0.981	4.4318e-02	-1.432	4.5200e-03	-0.603	3.4984e-01	pubmed
ENSMUSG00000018841	Rad51d	-0.613	4.1781e-02	-1.278	7.7230e-06	-0.585	7.8587e-02	pubmed
ENSMUSG00000018849	Wwc1	-0.191	5.0234e-01	-1.177	9.5918e-04	-1.135	1.8571e-03	pubmed
ENSMUSG00000019066	Rab3d	-1.075	1.9963e-05	-0.659	9.8172e-02	-0.509	3.1056e-01	pubmed
ENSMUSG00000019082	Slc25a22	-0.818	3.5172e-03	-1.217	3.4809e-05	-0.691	2.1991e-01	pubmed
ENSMUSG00000019139	Isyna1	-1.146	1.5173e-06	-1.455	1.2874e-04	-1.130	1.0977e-05	pubmed
ENSMUSG00000019256	Ahr	-0.199	5.2532e-01	-0.634	1.4842e-01	-1.601	5.7597e-04	pubmed
ENSMUSG00000019312	Grb7	-1.051	3.3765e-09	-1.903	1.5835e-13	-0.922	7.8478e-02	pubmed
ENSMUSG00000019467	Arhgef25	-1.919	2.0153e-04	-1.755	8.0764e-02	-0.590	6.0385e-01	pubmed
ENSMUSG00000019647	Sema6a	-1.173	5.3905e-03	-1.148	1.5511e-03	-0.778	2.1518e-01	pubmed
ENSMUSG00000019718	L3hypdh	-1.394	3.0831e-08	-1.984	1.1744e-11	-1.314	3.5986e-03	pubmed
ENSMUSG00000019726	Lyst	-1.629	1.6166e-13	-1.574	4.0133e-12	-0.398	5.5295e-01	pubmed
ENSMUSG00000019737	Syne4	-1.380	1.7812e-03	-0.873	1.2492e-01	-0.286	6.9492e-01	pubmed
ENSMUSG00000019763	Rmnd1	-0.966	7.7889e-07	-0.976	2.0040e-05	-1.151	1.0531e-04	pubmed
ENSMUSG00000019813	Cep57l1	-1.427	1.8082e-03	-1.601	1.5363e-03	-0.739	3.2003e-01	pubmed
ENSMUSG00000019878	Hsf2	-1.636	8.8434e-16	-1.684	3.3175e-09	-0.997	2.4812e-03	pubmed
ENSMUSG00000019906	Lin7a	-2.274	4.3853e-06	-2.107	1.1613e-09	-1.990	3.1270e-05	pubmed
ENSMUSG00000019948	Actr6	-1.163	7.9128e-06	-0.808	6.9091e-04	-0.900	1.4036e-03	pubmed
ENSMUSG00000020017	Hal	-1.327	5.6716e-10	-1.767	8.1837e-06	-1.371	1.2287e-05	pubmed
ENSMUSG00000020019	Ntn4	-1.395	2.2569e-06	-1.436	2.9686e-03	-0.482	1.1428e-01	pubmed
ENSMUSG00000020021	Fgd6	-1.121	7.1304e-10	-0.892	7.3727e-09	-0.970	8.6718e-03	pubmed
ENSMUSG00000020037	Rfx4	-3.044	4.4396e-03	-1.555	3.2838e-01	-1.680	2.0192e-01	pubmed
ENSMUSG00000020069	Hnrnph3	-1.105	1.7315e-08	-1.271	3.9068e-07	-0.837	2.2984e-03	pubmed
ENSMUSG00000020085	Aifm2	-1.117	2.3289e-20	-1.408	1.5770e-07	-0.666	1.0230e-01	pubmed
ENSMUSG00000020087	Tysnd1	-1.070	2.2743e-13	-0.994	7.9697e-04	-0.484	7.2124e-02	pubmed
ENSMUSG00000020099	Unc5b	-1.122	3.7796e-03	-1.136	5.5743e-02	-0.056	9.4911e-01	pubmed
ENSMUSG00000020100	Slc29a3	-0.563	4.0552e-03	-1.035	7.5950e-04	0.023	9.6760e-01	pubmed
ENSMUSG00000020131	Pcsk4	-2.277	1.8280e-11	-2.322	1.3532e-08	-0.782	2.5637e-01	pubmed
ENSMUSG00000020137	Thap2	-0.662	3.9351e-03	-0.648	1.9959e-01	-1.466	3.2150e-11	pubmed

Ensg	Symbol	CLP1 LFC	CLP1 FDR	CLP2 LFC	CLP2 FDR	CLP3 LFC	CLP3 FDR	Link
ENSMUSG00000020140	Lgr5	-2.829	1.0570e-09	-3.103	3.0540e-05	-2.625	2.1093e-04	pubmed
ENSMUSG00000020154	Ptprb	-1.437	1.0207e-13	-0.557	1.0795e-01	-0.289	5.1232e-01	pubmed
ENSMUSG00000020156	Pwwp3a	-1.027	3.0366e-05	-1.270	2.2466e-05	-0.542	1.0044e-01	pubmed
ENSMUSG00000020160	Meis1	-1.756	5.4409e-07	-1.623	1.4328e-02	-0.358	5.5427e-01	pubmed
ENSMUSG00000020175	Rab36	-3.399	1.6989e-10	-2.502	2.9790e-05	-1.250	6.4166e-02	pubmed
ENSMUSG00000020183	Cpm	-1.154	4.0783e-04	-1.103	2.0250e-03	-0.473	5.8871e-01	pubmed
ENSMUSG00000020205	Phlda1	-0.674	7.9035e-02	-1.004	7.1629e-03	-2.173	1.1675e-06	pubmed
ENSMUSG00000020258	Glyctk	-1.576	2.3067e-13	-1.741	6.7574e-04	-1.131	3.8611e-02	pubmed
ENSMUSG00000020261	Slc36a1	-1.379	1.3239e-17	-1.326	2.9856e-06	-0.424	2.4859e-01	pubmed
ENSMUSG00000020309	Chac2	-2.097	4.2615e-08	-2.775	2.9659e-22	-2.635	8.2385e-07	pubmed
ENSMUSG00000020335	Zfp354b	-2.632	3.8858e-05	-1.322	3.3252e-02	-1.205	1.8197e-01	pubmed
ENSMUSG00000020364	Zfp354a	-1.563	2.5394e-04	-1.719	1.9481e-03	-0.402	5.4463e-01	pubmed
ENSMUSG00000020380	Rad50	-0.662	9.9640e-03	-1.076	2.4455e-04	-0.154	7.3973e-01	pubmed
ENSMUSG00000020435	Osbp2	-4.024	6.8249e-03	-3.736	6.6399e-02	-2.983	1	pubmed
ENSMUSG00000020439	Smtn	-1.017	2.2217e-04	-0.705	1.9028e-01	0.091	9.1757e-01	pubmed
ENSMUSG00000020486	Sept4	-2.141	9.2275e-10	-1.618	3.6964e-03	-1.170	2.1661e-02	pubmed
ENSMUSG00000020513	Tubd1	-1.186	1.8961e-04	-0.729	3.9650e-01	-0.668	1.8219e-01	pubmed
ENSMUSG00000020534	Shmt1	-1.276	3.8604e-05	-1.232	2.0172e-05	-1.074	4.5833e-04	pubmed
ENSMUSG00000020546	Stxbp4	-1.794	5.6347e-05	-1.780	8.2835e-03	-0.970	7.2054e-02	pubmed
ENSMUSG00000020605	Hs1bp3	-0.245	3.0776e-01	-1.631	1.0448e-06	-0.986	2.2977e-02	pubmed
ENSMUSG00000020614	Fam20a	-1.872	8.1592e-29	-1.949	4.5883e-09	-0.833	2.6858e-01	pubmed
ENSMUSG00000020623	Map2k6	-3.821	1.4390e-05	-1.188	9.4056e-02	-1.617	1.2459e-01	pubmed
ENSMUSG00000020629	Adi1	-0.884	1.6579e-04	-1.109	9.6510e-04	-0.976	5.7679e-05	pubmed
ENSMUSG00000020674	Pxdn	-0.896	3.6521e-07	-1.507	1.3733e-04	-0.607	1.4076e-01	pubmed
ENSMUSG00000020707	Rnf135	-0.462	3.2188e-02	-1.490	1.1300e-06	-0.969	1.1230e-02	pubmed
ENSMUSG00000020709	Adap2	-1.310	6.2806e-13	-1.007	2.8871e-07	-0.800	4.0609e-03	pubmed
ENSMUSG00000020715	Ern1	-1.269	2.0807e-09	-1.543	2.1955e-09	-0.794	9.1705e-05	pubmed
ENSMUSG00000020718	Polg2	-1.930	1.4210e-10	-1.982	1.6848e-04	-2.287	4.8068e-05	pubmed
ENSMUSG00000020744	Slc25a19	-1.011	3.0085e-08	-1.157	2.1781e-02	-0.953	3.4701e-04	pubmed
ENSMUSG00000020810	Cygb	-1.145	2.9948e-05	-0.850	1.1455e-01	-0.039	9.5396e-01	pubmed
ENSMUSG00000020823	Sec14l1	-0.289	2.7290e-01	-1.075	1.6005e-03	-0.886	7.4212e-03	pubmed
ENSMUSG00000020827	Mink1	-0.896	2.8363e-14	-1.071	1.9016e-12	-0.640	3.1931e-02	pubmed
ENSMUSG00000020830	Vmo1	-1.631	2.3540e-03	-2.883	3.4046e-06	-1.335	7.3912e-02	pubmed
ENSMUSG00000020921	Tmem101	-0.116	8.0377e-01	-1.084	4.7403e-03	-0.205	6.1236e-01	pubmed
ENSMUSG00000021013	Ttc8	-1.156	6.8604e-03	-0.352	4.7888e-01	0.045	9.3208e-01	pubmed
ENSMUSG00000021097	Clmn	-1.314	6.7143e-25	-1.123	5.3374e-04	-0.802	2.2245e-02	pubmed
ENSMUSG00000021120	Pigh	-0.706	5.4081e-02	-1.609	1.7254e-03	-1.012	9.9465e-02	pubmed
ENSMUSG00000021135	Slc10a1	-1.018	1.1506e-04	-0.950	7.7092e-03	-1.006	2.8617e-02	pubmed
ENSMUSG00000021143	Pacs2	-0.395	1.2040e-02	-1.133	2.4506e-06	-0.491	1.3123e-01	pubmed
ENSMUSG00000021186	Fbln5	-1.322	2.5844e-04	-0.591	1.6859e-01	-1.030	9.7982e-02	pubmed
ENSMUSG00000021203	Otub2	-1.038	1.9831e-03	-0.781	2.5931e-01	-0.371	6.7255e-01	pubmed
ENSMUSG00000021240	Abcd4	-1.045	2.8596e-09	-0.942	7.6971e-04	-0.939	1.6223e-02	pubmed
ENSMUSG00000021245	Mlh3	-1.167	1.0483e-11	-1.504	3.8260e-03	-0.767	1.4335e-01	pubmed
ENSMUSG00000021286	Zfyve21	-1.019	1.1288e-05	-0.654	1.9662e-01	0.061	8.6476e-01	pubmed
ENSMUSG00000021294	Kif26a	-2.243	3.0013e-04	-1.902	3.9195e-02	-0.632	6.5411e-01	pubmed
ENSMUSG00000021327	Zkscan3	-1.299	1.2487e-21	-0.813	7.7520e-06	-0.926	3.3053e-03	pubmed
ENSMUSG00000021364	Elovl2	-0.710	1.1610e-06	-1.508	1.3566e-07	-1.771	3.8198e-09	pubmed
ENSMUSG00000021423	Ly86	-0.941	3.2009e-03	-0.949	6.5421e-02	-1.316	9.8516e-03	pubmed
ENSMUSG00000021466	Ptch1	-1.217	1.8795e-09	-1.813	1.6442e-08	-1.308	1.0497e-02	pubmed
ENSMUSG00000021509	Slc25a48	-1.276	1.9701e-04	-1.286	8.9886e-02	-1.620	1.7290e-02	pubmed
ENSMUSG00000021559	Dapk1	-1.229	4.1963e-04	-1.135	3.0944e-02	-1.031	7.5271e-04	pubmed
ENSMUSG00000021573	Tppp	-1.695	1.4422e-07	-1.912	1.2135e-06	-1.920	1.6541e-05	pubmed
ENSMUSG00000021611	Tert	-2.461	9.5599e-07	-2.272	9.0433e-04	-1.788	3.0003e-03	pubmed
ENSMUSG00000021636	Marveld2	-1.336	1.9389e-06	-1.710	6.5536e-11	-1.174	3.6181e-03	pubmed
ENSMUSG00000021638	Ocln	-1.809	1.8331e-16	-2.061	2.8420e-16	-0.725	1.0638e-01	pubmed
ENSMUSG00000021779	Thrb	-1.017	2.5180e-11	-0.392	5.6039e-02	-0.614	2.8811e-02	pubmed
ENSMUSG00000021824	Ap3m1	-0.950	1.8214e-22	-1.077	5.9980e-03	-0.761	3.6534e-02	pubmed
ENSMUSG00000021876	Rnase4	-0.524	1.3983e-06	-0.685	1.4586e-05	-1.116	9.1159e-05	pubmed
ENSMUSG00000021884	Hacl1	-1.232	2.3419e-04	-0.566	1.5741e-01	-1.120	1.3532e-02	pubmed
ENSMUSG00000021902	Phf7	-1.229	4.9106e-05	-1.542	5.6286e-07	-0.734	7.2604e-02	pubmed
ENSMUSG00000021943	Gdf10	-1.724	1.5674e-04	-0.551	4.4459e-01	-0.555	3.0502e-01	pubmed
ENSMUSG00000021959	Lats2	-0.142	5.5704e-01	-0.548	4.9792e-02	-1.009	3.2980e-03	pubmed
ENSMUSG00000022041	Chrna2	-2.726	2.4614e-05	-1.888	5.2694e-06	-2.222	2.6917e-05	pubmed
ENSMUSG00000022048	Dpysl2	-0.651	2.3832e-01	-1.767	6.7042e-03	-0.299	7.6213e-01	pubmed
ENSMUSG00000022075	Rhobtb2	-1.589	2.3066e-12	-1.646	3.0895e-05	-0.583	1.5963e-01	pubmed
ENSMUSG00000022091	Sorbs3	-1.751	1.7411e-13	-2.087	2.3880e-06	-1.292	3.0516e-04	pubmed
ENSMUSG00000022103	Gfra2	-2.862	2.1829e-03	-4.311	1.2584e-02	-0.764	1	pubmed
ENSMUSG00000022178	Ajuba	-1.544	2.4367e-06	-2.270	3.2448e-07	-0.843	2.3453e-01	pubmed
ENSMUSG00000022179	4931414P19Rik	-1.907	6.3835e-07	-2.183	2.1211e-03	-0.990	1.1077e-01	pubmed
ENSMUSG00000022364	Tbc1d31	-1.553	6.8962e-04	-1.079	6.4307e-02	0.139	8.3436e-01	pubmed
ENSMUSG00000022383	Ppara	-1.274	2.5658e-07	-2.102	1.8765e-10	-1.477	7.5051e-02	pubmed
ENSMUSG00000022388	Ttll8	-1.154	5.9079e-03	-2.040	2.6113e-03	-2.006	1.2107e-01	pubmed
ENSMUSG00000022389	Tef	-1.406	9.4723e-07	-1.438	8.3611e-08	-0.812	4.2233e-02	pubmed
ENSMUSG00000022428	Cby1	-1.746	1.5390e-07	-1.856	1.7832e-06	-0.986	3.2253e-02	pubmed
ENSMUSG00000022442	Ttll1	-2.103	2.7568e-04	-2.596	4.5925e-05	-0.922	1.1148e-01	pubmed
ENSMUSG00000022475	Hdac7	-1.092	1.3492e-08	-0.592	1.0632e-01	-0.173	7.7859e-01	pubmed
ENSMUSG00000022494	Shisa9	-6.084	7.4319e-06	-0.911	4.6689e-01	-0.147	9.2036e-01	pubmed
ENSMUSG00000022512	Cldn1	-2.587	8.5860e-16	-1.750	6.6361e-06	-1.703	4.1672e-03	pubmed
ENSMUSG00000022528	Hes1	-0.660	2.6920e-02	-1.267	2.0014e-04	-0.166	7.4747e-01	pubmed
ENSMUSG00000022537	Tmem44	-2.885	1.6782e-07	-1.757	1.2644e-02	-0.763	2.8272e-01	pubmed
ENSMUSG00000022540	Rogdi	-1.004	2.2592e-05	-0.358	4.1746e-01	-0.764	4.6810e-03	pubmed
ENSMUSG00000022560	Slc52a2	-1.036	1.4467e-07	-0.945	1.4803e-01	-0.363	6.2124e-01	pubmed
ENSMUSG00000022571	Pycrl	-1.501	4.5188e-14	-1.936	7.2918e-10	-1.398	5.2731e-04	pubmed
ENSMUSG00000022574	Naprt	-0.656	4.3484e-03	-1.213	4.5491e-12	-1.262	2.0322e-07	pubmed
ENSMUSG00000022676	Snai2	-1.348	2.5820e-03	-1.382	5.6838e-02	-1.374	1.7126e-02	pubmed
ENSMUSG00000022701	Ccdc191	-1.497	3.5161e-08	-1.219	9.1352e-02	-0.766	4.1591e-02	pubmed
ENSMUSG00000022742	Cpox	-2.426	2.3131e-22	-1.348	5.1163e-05	-1.128	6.8495e-04	pubmed
ENSMUSG00000022761	Lztr1	-0.702	9.4766e-18	-1.111	5.8945e-05	-0.683	7.4534e-02	pubmed
ENSMUSG00000022774	Ncbp2	-0.794	8.4271e-07	-1.106	1.5251e-02	-1.241	3.2109e-03	pubmed
ENSMUSG00000022788	Fgd4	-1.367	3.1924e-08	-1.601	2.4405e-11	-0.720	7.7904e-02	pubmed
ENSMUSG00000022791	Tnk2	-1.087	1.4556e-08	-0.237	6.1731e-01	-0.257	4.4844e-01	pubmed

		CLP1		CLP2		CLP3		
Ensg	Symbol	LFC	FDR	LFC	FDR	LFC	FDR	Link
ENSMUSG00000022802	Lmln	-1.756	2.1860e-05	-2.714	1.5276e-05	-0.337	6.0406e-01	pubmed
ENSMUSG00000022809	Nr1i2	-0.774	2.9363e-05	-1.016	3.9285e-05	-1.416	4.8925e-03	pubmed
ENSMUSG00000022817	Itgb5	-1.285	1.4573e-13	-1.096	1.6346e-08	-0.775	2.1570e-03	pubmed
ENSMUSG00000022836	Mylk	-1.098	6.9033e-07	-0.553	4.8675e-03	-0.640	1.4204e-01	pubmed
ENSMUSG00000022843	Clcn2	-1.128	1.4917e-17	-0.763	3.2795e-02	-0.721	2.3627e-02	pubmed
ENSMUSG00000022856	Tmem41a	-1.046	2.0746e-09	-1.389	1.3487e-03	-1.029	3.8163e-02	pubmed
ENSMUSG00000022877	Hrg	-0.711	5.6358e-08	-0.888	9.3711e-07	-1.018	2.3121e-05	pubmed
ENSMUSG00000022881	Rfc4	-1.569	2.0576e-04	0.115	8.5464e-01	-0.275	6.7231e-01	pubmed
ENSMUSG00000022883	Robo1	0.100	8.6819e-01	-1.230	3.6789e-03	-0.897	2.1022e-02	pubmed
ENSMUSG00000022948	Setd4	-1.287	1.9209e-03	-0.548	2.9181e-01	-0.144	8.3438e-01	pubmed
ENSMUSG00000022994	Adcy6	-1.747	1.3307e-29	-2.273	5.6593e-19	-0.741	1.4555e-01	pubmed
ENSMUSG00000023017	Asic1	-1.465	8.4978e-03	-1.475	1.2821e-01	-0.832	4.0811e-01	pubmed
ENSMUSG00000023022	Lima1	-1.095	2.6026e-13	-1.463	1.0099e-08	-1.098	7.7040e-03	pubmed
ENSMUSG00000023066	Rttn	-1.292	2.5786e-03	-1.015	7.9693e-02	-0.404	5.6369e-01	pubmed
ENSMUSG00000023087	Noct	-1.377	1.3819e-03	-0.016	9.7467e-01	-0.246	8.7287e-01	pubmed
ENSMUSG00000023094	Msrb2	-1.048	1.1811e-05	-0.886	7.4527e-03	-0.600	1.1257e-01	pubmed
ENSMUSG00000023176	Cpn2	-0.942	6.3585e-10	-1.277	2.9545e-06	-1.218	2.0281e-05	pubmed
ENSMUSG00000023243	Kcnk5	-2.052	1.2226e-04	-2.063	1.0415e-11	-0.463	4.7795e-01	pubmed
ENSMUSG00000023266	Frs3	-1.363	2.6743e-03	-2.137	7.9464e-03	0.030	9.7938e-01	pubmed
ENSMUSG00000023495	Pcbp4	-1.125	4.3777e-07	-1.441	6.9506e-03	-0.637	1.8364e-01	pubmed
ENSMUSG00000023800	Tiam2	-1.073	4.8198e-04	-1.146	3.0219e-03	-0.444	5.1549e-01	pubmed
ENSMUSG00000023885	Thbs2	-2.073	2.5662e-05	-0.665	3.3090e-01	-0.166	8.4179e-01	pubmed
ENSMUSG00000023909	Paqr4	-2.495	2.3759e-04	-1.966	9.3221e-03	-1.385	1.4773e-01	pubmed
ENSMUSG00000023912	Slc25a27	-1.948	2.0340e-05	-2.732	1.4659e-02	-0.901	3.1498e-01	pubmed
ENSMUSG00000023942	Slc29a1	-1.759	5.4516e-17	-1.832	9.1073e-20	-1.458	5.8710e-06	pubmed
ENSMUSG00000023990	Tfeb	-1.493	2.9305e-06	-1.919	7.6656e-04	-0.823	1.9191e-01	pubmed
ENSMUSG00000024013	Fgd2	-2.448	1.0902e-08	-2.625	4.8340e-03	-1.306	1.7362e-02	pubmed
ENSMUSG00000024049	Myom1	-1.212	8.2949e-03	-0.539	4.6996e-01	-0.282	7.9216e-01	pubmed
ENSMUSG00000024052	Lpin2	-1.826	2.0837e-12	-2.396	1.2795e-68	-1.559	1.0278e-03	pubmed
ENSMUSG00000024063	Lbh	-1.120	3.6264e-05	-0.068	9.1142e-01	0.090	8.7334e-01	pubmed
ENSMUSG00000024065	Ehd3	-1.824	3.0494e-21	-1.224	7.0955e-04	-1.082	1.3529e-03	pubmed
ENSMUSG00000024070	Prkd3	-1.795	7.7334e-26	-1.916	7.4165e-33	-1.164	1.6239e-11	pubmed
ENSMUSG00000024118	Tedc2	-2.305	8.5440e-35	-2.302	9.7304e-21	-1.730	2.6426e-05	pubmed
ENSMUSG00000024131	Slc3a1	-0.823	4.0552e-03	-1.344	1.0645e-04	-1.383	3.6332e-04	pubmed
ENSMUSG00000024155	Meiob	-1.503	2.2651e-03	-1.282	8.0575e-03	-1.512	1.8643e-04	pubmed
ENSMUSG00000024168	Tmem204	-1.430	2.8611e-08	-0.546	3.4149e-01	-0.469	3.0633e-01	pubmed
ENSMUSG00000024169	Ift140	-1.869	1.5694e-14	-1.862	1.7738e-05	-0.971	2.8757e-02	pubmed
ENSMUSG00000024206	Rfx2	-1.522	9.8157e-03	-1.282	1.3116e-01	-0.716	3.7862e-01	pubmed
ENSMUSG00000024232	Bambi	-0.981	1.2863e-02	-1.423	9.1706e-04	0.022	9.7922e-01	pubmed
ENSMUSG00000024254	Abcg8	-0.676	1.1157e-01	-0.522	3.8100e-01	-1.254	2.5128e-03	pubmed
ENSMUSG00000024299	Adamts10	-1.619	2.2606e-10	-0.549	2.0373e-01	-0.006	9.9292e-01	pubmed
ENSMUSG00000024395	Lims2	-1.814	2.7022e-13	-1.377	2.7054e-05	-1.079	7.9631e-04	pubmed
ENSMUSG00000024397	Aif1	-0.763	1.4506e-01	-0.627	4.4618e-01	-2.070	8.3912e-03	pubmed
ENSMUSG00000024411	Aqp4	-0.989	7.5368e-02	-1.845	1.2519e-07	-0.763	2.1464e-01	pubmed
ENSMUSG00000024413	Npc1	-1.586	5.7952e-22	-1.380	2.6810e-10	-0.765	8.8833e-03	pubmed
ENSMUSG00000024426	Atat1	-1.445	1.0992e-14	-1.434	2.2047e-04	-1.123	1.0534e-02	pubmed
ENSMUSG00000024440	Pcdh12	-4.219	4.3491e-15	-2.694	3.6789e-07	-1.138	9.6324e-02	pubmed
ENSMUSG00000024580	Grpel2	-1.312	2.7861e-13	-1.081	1.4843e-03	-0.653	9.2323e-02	pubmed
ENSMUSG00000024663	Rab3il1	-1.963	5.6505e-06	-2.031	3.8725e-04	-1.692	2.1140e-02	pubmed
ENSMUSG00000024664	Fads3	-1.058	8.7065e-03	-0.471	4.3121e-01	0.068	9.4869e-01	pubmed
ENSMUSG00000024665	Fads2	-0.953	1.5860e-05	-1.457	4.0101e-05	-1.440	3.0706e-08	pubmed
ENSMUSG00000024759	Atl3	-1.025	9.7395e-10	-1.044	8.2221e-11	-0.766	1.6398e-02	pubmed
ENSMUSG00000024769	Cdc42bpg	-1.140	6.2480e-12	-1.494	3.9820e-07	-1.064	2.4556e-02	pubmed
ENSMUSG00000024787	Snx15	-0.680	3.1031e-02	-1.153	3.2399e-05	-0.672	1.6846e-02	pubmed
ENSMUSG00000024818	Slc25a45	-1.665	1.4938e-24	-1.857	6.5188e-12	-1.246	6.2273e-03	pubmed
ENSMUSG00000024866	Acy3	-0.750	1.3483e-02	-1.259	1.6047e-07	-0.735	3.8938e-02	pubmed
ENSMUSG00000024891	Slc29a2	-2.779	4.6163e-02	-4.706	5.0179e-03	-2.209	1.0519e-01	pubmed
ENSMUSG00000024900	Cpt1a	-0.730	1.3734e-06	-1.022	2.9174e-11	-0.871	1.2021e-03	pubmed
ENSMUSG00000024906	Mus81	-1.070	3.4161e-05	-1.013	6.9998e-03	-0.646	2.1531e-01	pubmed
ENSMUSG00000024921	Smarca2	-1.253	1.1216e-07	-1.440	9.9565e-14	-1.158	2.7686e-03	pubmed
ENSMUSG00000024947	Men1	-0.683	1.3851e-04	-1.156	5.6866e-03	-0.692	9.8883e-03	pubmed
ENSMUSG00000024955	Esrra	-1.466	2.9537e-21	-1.587	5.1019e-06	-0.744	1.3600e-01	pubmed
ENSMUSG00000024958	Gpr137	-1.058	2.4058e-08	-1.237	5.4302e-07	-0.598	4.5887e-02	pubmed
ENSMUSG00000024959	Bad	-1.114	2.0518e-09	-1.014	1.3415e-02	-0.895	9.1280e-03	pubmed
ENSMUSG00000024960	Plcb3	-1.125	1.4755e-18	-1.283	4.0925e-12	-0.626	4.0005e-02	pubmed
ENSMUSG00000024986	Hhex	-4.664	8.8887e-97	-4.020	1.7887e-30	-3.423	4.7525e-10	pubmed
ENSMUSG00000025003	Cyp2c39	-0.745	4.4346e-01	-1.168	4.1975e-02	-1.933	2.2413e-04	pubmed
ENSMUSG00000025014	Dntt	-1.098	1.5315e-03	-1.323	1.6254e-02	-1.052	6.5158e-02	pubmed
ENSMUSG00000025036	Sfxn2	-0.668	3.4150e-09	-0.941	2.3214e-04	-1.012	1.5389e-05	pubmed
ENSMUSG00000025092	Hspa12a	-2.506	2.7100e-07	-2.025	3.9548e-03	-0.059	9.6920e-01	pubmed
ENSMUSG00000025145	Lrrc45	-1.388	6.2202e-08	-1.147	1.1574e-02	-0.910	1.0096e-02	pubmed
ENSMUSG00000025154	Arhgap19	-2.132	4.1522e-11	-1.295	1.1990e-02	-1.088	1.9600e-02	pubmed
ENSMUSG00000025175	Fn3k	-1.636	1.2275e-07	-1.342	3.5072e-03	-1.235	1.4333e-02	pubmed
ENSMUSG00000025194	Abcc2	-1.389	1.5854e-08	-1.004	2.6637e-05	-1.515	1.2809e-05	pubmed
ENSMUSG00000025195	Dnmbp	-0.839	1.5466e-10	-1.245	2.5662e-12	-0.776	3.2661e-03	pubmed
ENSMUSG00000025209	Twnk	-1.360	2.0297e-12	-1.378	1.3711e-04	-0.604	1.2744e-01	pubmed
ENSMUSG00000025213	Kazald1	-3.825	8.9082e-03	-1.039	5.8657e-01	-0.403	1	pubmed
ENSMUSG00000025227	Mfsd13a	-0.308	4.3406e-01	-1.343	7.5160e-03	-0.641	3.2572e-01	pubmed
ENSMUSG00000025241	Fyco1	-0.824	6.5541e-10	-1.403	5.6270e-22	-0.692	1.1258e-03	pubmed
ENSMUSG00000025262	Fam120c	-0.839	2.7162e-02	-1.758	5.9123e-04	-1.142	1.4971e-02	pubmed
ENSMUSG00000025271	Pfkfb1	-1.656	5.9258e-13	-1.542	7.0843e-12	-1.839	3.4942e-06	pubmed
ENSMUSG00000025278	Flnb	-0.315	3.4360e-01	-1.078	1.3610e-05	-0.492	2.0502e-01	pubmed
ENSMUSG00000025279	Dnase1l3	-0.716	8.9277e-03	-1.226	1.5557e-07	-0.986	7.5435e-04	pubmed
ENSMUSG00000025316	Banp	-2.336	3.2760e-15	-1.849	1.2183e-02	-1.798	3.2568e-03	pubmed
ENSMUSG00000025317	Car5a	-1.447	1.4386e-04	-1.834	5.1550e-06	-1.311	6.7455e-03	pubmed
ENSMUSG00000025347	Mettl7b	-0.969	3.9656e-03	-1.506	2.2532e-08	-1.234	1.5109e-03	pubmed
ENSMUSG00000025348	Itga7	-1.365	4.8796e-08	-0.842	8.0585e-04	-0.396	2.1794e-01	pubmed
ENSMUSG00000025355	Mmp19	-0.582	3.4689e-02	-1.146	4.3253e-13	-0.682	3.2589e-03	pubmed
ENSMUSG00000025369	Smarcc2	-0.526	1.5915e-08	-1.002	2.8083e-11	-0.414	8.3211e-02	pubmed
ENSMUSG00000025372	Baiap2	-1.052	2.0371e-03	-2.070	7.0055e-04	-0.701	3.0332e-01	pubmed
ENSMUSG00000025375	Aatk	-1.519	3.8341e-06	-1.808	1.4873e-02	-0.864	2.7007e-01	pubmed

Ensg	Symbol	CLP1 LFC	CLP1 FDR	CLP2 LFC	CLP2 FDR	CLP3 LFC	CLP3 FDR	Link
ENSMUSG00000025402	Nab2	-1.279	3.2890e-04	-2.051	3.4866e-06	-1.241	5.2254e-02	pubmed
ENSMUSG00000025405	Inhbc	-0.886	1.2764e-02	-1.766	4.6371e-11	-1.351	3.3619e-03	pubmed
ENSMUSG00000025422	Agap2	-2.055	6.9234e-11	-2.955	3.7986e-09	-1.064	2.0261e-01	pubmed
ENSMUSG00000025464	Paox	-0.677	3.1398e-05	-0.856	3.6650e-04	-1.167	9.1722e-04	pubmed
ENSMUSG00000025479	Cyp2e1	-0.800	8.5772e-03	-0.478	6.1537e-02	-1.021	7.4580e-04	pubmed
ENSMUSG00000025494	Sigirr	-1.122	1.7309e-14	-1.159	5.7831e-09	-1.299	1.3037e-04	pubmed
ENSMUSG00000025495	Ptdss2	-0.662	1.3601e-03	-1.181	8.2930e-04	-0.756	1.8763e-02	pubmed
ENSMUSG00000025504	Eps8l2	-0.588	1.2803e-03	-1.991	1.8580e-04	-1.958	1.8857e-07	pubmed
ENSMUSG00000025505	Tmem80	-0.872	4.6207e-05	-1.227	2.8388e-04	-1.178	6.9805e-02	pubmed
ENSMUSG00000025533	Asl	-1.005	1.2891e-13	-0.510	1.1312e-01	-0.424	2.3411e-01	pubmed
ENSMUSG00000025538	Sumf2	-1.148	5.1251e-06	-1.003	1.2339e-02	-0.981	3.9914e-03	pubmed
ENSMUSG00000025577	Cbx2	-3.071	3.4897e-13	-3.359	2.5398e-14	-1.698	3.4750e-04	pubmed
ENSMUSG00000025578	Cbx8	-2.219	3.3866e-10	-1.240	3.6766e-02	-1.370	1.0375e-02	pubmed
ENSMUSG00000025608	Podxl	-0.282	5.0412e-01	-1.218	2.2900e-03	-0.773	1.9266e-01	pubmed
ENSMUSG00000025732	Mcrip2	-1.937	2.4566e-12	-1.261	5.2436e-04	-1.134	2.8348e-05	pubmed
ENSMUSG00000025795	Rassf3	-1.352	1.8157e-23	-1.162	6.8867e-06	-0.742	9.7326e-02	pubmed
ENSMUSG00000025813	Homer2	-1.007	1.2200e-03	-1.246	1.5276e-05	-0.898	7.6895e-04	pubmed
ENSMUSG00000025815	Dhtkd1	-1.933	3.2010e-09	-1.247	1.1652e-05	-1.144	3.4114e-03	pubmed
ENSMUSG00000025854	Fam20c	-1.037	4.3963e-04	-2.705	3.1860e-15	-0.891	1.6106e-01	pubmed
ENSMUSG00000025856	Pdgfa	-0.905	4.9903e-03	-1.922	1.0250e-04	-0.913	2.3451e-01	pubmed
ENSMUSG00000025893	Kbtbd3	-1.095	2.5490e-03	-0.351	4.7740e-01	-0.462	3.5647e-01	pubmed
ENSMUSG00000025902	Sox17	-1.034	1.7077e-03	-0.086	9.2230e-01	0.746	2.6438e-01	pubmed
ENSMUSG00000025964	Adam23	-1.424	1.2248e-09	-0.768	3.9821e-02	-0.407	1.4300e-01	pubmed
ENSMUSG00000025993	Slc40a1	-2.568	1.2614e-24	-2.027	7.4543e-12	-1.462	6.7227e-04	pubmed
ENSMUSG00000026004	Kansl1l	-0.542	4.7074e-02	-1.048	3.7606e-04	-1.434	2.1953e-04	pubmed
ENSMUSG00000026024	Als2	-1.370	7.0193e-13	-1.280	1.1518e-09	-0.620	4.7823e-03	pubmed
ENSMUSG00000026027	Stradb	-0.717	3.2027e-05	-0.900	1.6368e-06	-1.093	2.4920e-05	pubmed
ENSMUSG00000026097	Ormdl1	-1.127	6.4508e-06	-0.973	6.7735e-05	-0.970	5.3752e-04	pubmed
ENSMUSG00000026098	Pms1	-1.422	2.3715e-06	-0.923	4.8140e-03	-0.668	5.6966e-02	pubmed
ENSMUSG00000026117	Zap70	-0.590	2.1207e-01	-1.548	3.1077e-04	-0.858	3.7596e-02	pubmed
ENSMUSG00000026179	Pnkd	-0.871	8.0173e-05	-1.359	1.7697e-03	-1.483	2.5731e-04	pubmed
ENSMUSG00000026185	Igfbp5	-1.964	3.0494e-08	-1.038	2.6221e-01	0.033	9.7656e-01	pubmed
ENSMUSG00000026202	Tuba4a	-0.754	1.3268e-03	-1.088	5.7082e-09	-0.407	1.2457e-01	pubmed
ENSMUSG00000026227	2810459M11Rik	-1.931	3.6356e-31	-2.219	2.4939e-18	-1.785	4.5171e-05	pubmed
ENSMUSG00000026259	Ngef	-1.905	1.6791e-10	-1.475	2.8987e-03	-1.520	4.4296e-03	pubmed
ENSMUSG00000026278	Bok	-0.364	3.1603e-01	-0.682	1.2607e-02	-1.280	4.6329e-03	pubmed
ENSMUSG00000026304	Rab17	-0.964	4.7589e-03	-1.154	2.0172e-05	-0.616	2.8393e-02	pubmed
ENSMUSG00000026315	Serpinb8	-1.417	5.6260e-03	-1.873	1.0204e-03	-1.047	1.6156e-01	pubmed
ENSMUSG00000026437	Cdk18	-1.101	9.5982e-29	-1.073	1.0123e-04	-0.316	1.7832e-01	pubmed
ENSMUSG00000026447	Pik3c2b	-1.380	5.3336e-09	-1.548	7.9285e-05	-0.595	3.3347e-01	pubmed
ENSMUSG00000026456	Cyb5r1	-0.387	2.2586e-01	-1.161	8.2528e-04	-0.507	4.0951e-01	pubmed
ENSMUSG00000026494	Kif26b	-1.185	5.4630e-03	-0.798	2.8477e-01	-0.846	1.6796e-01	pubmed
ENSMUSG00000026578	Ccdc181	-0.554	1.0763e-01	-1.303	8.5672e-03	-0.776	8.6902e-02	pubmed
ENSMUSG00000026585	Kifap3	-0.883	3.4742e-03	-1.322	2.2656e-05	-0.677	1.6010e-01	pubmed
ENSMUSG00000026589	Sec16b	-0.971	4.8266e-08	-1.047	1.2273e-04	-0.891	1.8478e-02	pubmed
ENSMUSG00000026594	Ralgps2	-1.321	6.5086e-08	-1.999	1.2202e-29	-1.286	4.8589e-05	pubmed
ENSMUSG00000026621	Marc1	-1.206	1.9639e-15	-1.443	1.0301e-06	-1.508	9.9267e-07	pubmed
ENSMUSG00000026627	Tmem206	-1.671	2.8557e-04	-1.956	8.2994e-03	-1.404	2.5305e-02	pubmed
ENSMUSG00000026638	Irf6	-1.014	1.9140e-04	-0.454	1.2418e-01	0.084	8.3686e-01	pubmed
ENSMUSG00000026639	Lamb3	-1.386	6.4130e-07	-0.526	3.5891e-01	-0.472	3.6795e-01	pubmed
ENSMUSG00000026656	Fcgr2b	-1.170	2.5990e-07	-1.004	1.2785e-07	-1.175	7.3737e-08	pubmed
ENSMUSG00000026659	Dusp12	-1.273	2.7876e-06	-1.038	4.8539e-03	-0.762	5.8902e-02	pubmed
ENSMUSG00000026669	Mcm10	-1.554	3.8879e-10	-2.650	3.4504e-15	-1.554	5.9449e-04	pubmed
ENSMUSG00000026692	Fmo4	-2.857	3.3183e-15	-2.016	4.1893e-06	-0.805	2.9317e-01	pubmed
ENSMUSG00000026698	Pigc	-1.012	6.1621e-09	-0.883	4.2193e-02	-0.535	2.5233e-01	pubmed
ENSMUSG00000026730	Pter	-0.734	2.5617e-02	-1.710	1.4328e-17	-1.422	7.8476e-07	pubmed
ENSMUSG00000026811	St6galnac6	-1.141	8.0388e-27	-1.347	4.9318e-06	-1.458	1.8102e-06	pubmed
ENSMUSG00000026840	Lamc3	-1.043	1.5848e-03	-0.108	9.0414e-01	-0.783	2.7598e-01	pubmed
ENSMUSG00000026853	Crat	-1.704	1.5516e-10	-1.861	7.3371e-16	-1.526	5.1370e-04	pubmed
ENSMUSG00000026858	Miga2	-2.649	9.6807e-84	-2.588	5.6098e-10	-1.236	2.4516e-02	pubmed
ENSMUSG00000026866	Kynu	-0.483	5.1315e-02	-0.736	1.2077e-02	-1.048	6.1910e-04	pubmed
ENSMUSG00000026870	Cutal	-2.125	4.3639e-26	-2.077	4.9920e-23	-1.307	4.7135e-03	pubmed
ENSMUSG00000026873	Phf19	-2.517	2.8183e-04	-2.811	1.5248e-02	-0.244	8.8030e-01	pubmed
ENSMUSG00000026883	Dab2ip	-1.266	2.1720e-16	-0.943	1.7185e-05	-0.262	4.8090e-01	pubmed
ENSMUSG00000026890	Lhx6	-2.570	1.3612e-08	-1.552	4.5783e-03	-0.397	4.1630e-01	pubmed
ENSMUSG00000026918	Brd3	-1.061	2.3589e-21	-1.064	3.2747e-04	-0.826	2.2845e-02	pubmed
ENSMUSG00000026925	Inpp5e	-1.012	7.1456e-05	-1.118	1.8600e-02	-0.364	4.5414e-01	pubmed
ENSMUSG00000026932	Nacc2	-1.137	9.4408e-09	-1.259	2.9971e-07	-0.713	1.4178e-02	pubmed
ENSMUSG00000027001	Dusp19	-1.372	3.1394e-06	-1.398	3.0839e-04	-0.585	3.7656e-01	pubmed
ENSMUSG00000027016	Zfp385b	-2.477	3.2376e-14	-2.414	9.7315e-11	-1.826	1.5012e-02	pubmed
ENSMUSG00000027086	Fastkd1	-1.216	1.0483e-11	-1.108	6.4208e-06	-0.818	1.8569e-02	pubmed
ENSMUSG00000027199	Gatm	-1.511	6.7871e-05	-0.783	7.7234e-02	-0.820	5.6621e-02	pubmed
ENSMUSG00000027242	Wdr76	-2.118	1.1010e-06	-1.733	1.5033e-02	-0.089	9.0804e-01	pubmed
ENSMUSG00000027243	Harbi1	-1.069	2.9585e-11	-1.233	6.4929e-04	-0.892	5.2028e-02	pubmed
ENSMUSG00000027253	Lrp4	-1.278	2.2055e-10	-1.258	1.9914e-04	-0.648	3.4387e-02	pubmed
ENSMUSG00000027259	Adal	-1.021	5.3683e-07	-1.116	6.3739e-07	-1.293	3.3903e-04	pubmed
ENSMUSG00000027274	Mkks	-1.452	7.9434e-08	-1.413	2.4326e-04	-1.450	1.8287e-03	pubmed
ENSMUSG00000027309	4930402H24Rik	-0.897	4.3067e-07	-1.396	3.2933e-06	-0.788	1.4526e-01	pubmed
ENSMUSG00000027312	Atrn	-1.039	1.0677e-10	-0.371	2.2780e-01	-0.250	5.9727e-01	pubmed
ENSMUSG00000027322	Siglec1	-1.234	1.6847e-14	-0.728	9.1352e-02	-1.197	4.8567e-06	pubmed
ENSMUSG00000027330	Cdc25b	-1.274	7.2462e-03	-1.160	2.0833e-01	-0.797	3.9078e-01	pubmed
ENSMUSG00000027353	Mcm8	-1.222	9.6800e-03	-0.604	3.3305e-01	-0.055	9.5213e-01	pubmed
ENSMUSG00000027358	Bmp2	-1.355	2.1926e-06	-1.097	2.3234e-03	-1.484	5.8515e-04	pubmed
ENSMUSG00000027359	Slc27a2	-0.872	1.7596e-01	-0.852	1.0226e-05	-1.025	1.6529e-06	pubmed
ENSMUSG00000027424	Mgme1	-0.907	7.8403e-04	-1.431	5.0956e-03	-1.562	1.3907e-05	pubmed
ENSMUSG00000027439	Gzf1	-1.046	1.7773e-10	-1.246	4.3969e-11	-0.954	1.5389e-05	pubmed
ENSMUSG00000027513	Pck1	-0.930	2.2285e-03	-2.519	7.3831e-09	-1.927	2.3600e-01	pubmed
ENSMUSG00000027579	Srms	-1.764	6.7806e-04	-1.525	2.6590e-02	-0.168	8.7522e-01	pubmed
ENSMUSG00000027589	Pcmtd2	-0.820	4.9435e-06	-1.206	4.5313e-04	-0.984	7.8744e-05	pubmed
ENSMUSG00000027634	Ndrg3	-1.297	1.3183e-16	-1.351	9.1911e-05	-0.578	9.9695e-02	pubmed
ENSMUSG00000027762	Sucnr1	-3.887	4.5881e-16	-2.359	5.3663e-06	-1.405	4.2219e-02	pubmed

		CLP1		CLP2		CLP3		
Ensg	Symbol	LFC	FDR	LFC	FDR	LFC	FDR	Link
ENSMUSG00000027765	P2ry1	-2.921	2.1590e-08	-2.052	1.1741e-05	-1.525	2.3166e-03	pubmed
ENSMUSG00000027796	Smad9	-3.909	2.6738e-19	-3.275	7.3350e-08	-2.037	3.2308e-03	pubmed
ENSMUSG00000027858	Tspan2	-0.441	3.6757e-01	-1.428	6.7006e-03	-0.574	5.0823e-01	pubmed
ENSMUSG00000027931	Npr1	-2.157	9.9759e-16	-2.127	1.8534e-08	-0.915	1.6241e-01	pubmed
ENSMUSG00000027932	Slc27a3	-2.650	2.3141e-04	-2.010	1.3036e-01	-0.634	5.4811e-01	pubmed
ENSMUSG00000027983	Cyp2u1	-3.423	5.2806e-22	-3.266	6.6264e-15	-1.826	6.3776e-04	pubmed
ENSMUSG00000028035	Dnajb4	-1.242	7.0379e-08	-0.923	4.1832e-04	-0.794	4.0271e-02	pubmed
ENSMUSG00000028082	Sh3d19	-0.581	1.2662e-08	-1.090	8.8289e-10	-0.742	7.3792e-02	pubmed
ENSMUSG00000028088	Fmo5	-1.273	5.1404e-12	-0.821	5.5918e-03	-0.837	4.1642e-02	pubmed
ENSMUSG00000028101	Pias3	-1.184	3.9440e-06	-1.028	1.2813e-01	-0.558	2.7213e-01	pubmed
ENSMUSG00000028121	Bcar3	-2.471	8.2551e-30	-1.885	8.6855e-05	-0.772	1.3297e-01	pubmed
ENSMUSG00000028128	F3	-1.758	4.3815e-09	-0.290	6.3488e-01	0.199	6.8792e-01	pubmed
ENSMUSG00000028145	Them4	-2.472	2.8024e-31	-1.874	1.9835e-08	-1.633	9.8620e-02	pubmed
ENSMUSG00000028150	Rorc	-1.313	1.2904e-03	-0.449	4.0301e-01	-0.213	7.6213e-01	pubmed
ENSMUSG00000028240	Cyp7a1	-5.420	1.9006e-06	-6.580	4.0929e-17	-4.642	1.4255e-10	pubmed
ENSMUSG00000028251	Tstd3	-0.823	2.8098e-03	-1.013	5.0391e-09	-1.041	7.6208e-04	pubmed
ENSMUSG00000028312	Smc2	-1.225	1.0995e-02	-1.358	7.6251e-04	-0.736	1.1549e-01	pubmed
ENSMUSG00000028339	Col15a1	-1.351	1.3070e-03	-1.229	6.0808e-03	-0.588	1.8626e-01	pubmed
ENSMUSG00000028344	Invs	-1.679	4.5601e-13	-1.311	3.3629e-02	-0.897	9.2913e-02	pubmed
ENSMUSG00000028383	Hsdl2	-0.994	1.2465e-05	-0.748	7.9887e-06	-1.086	1.7270e-04	pubmed
ENSMUSG00000028402	Mpdz	-1.087	5.4009e-04	-1.367	4.2760e-06	-1.017	3.0864e-03	pubmed
ENSMUSG00000028434	Epb41l4b	-0.733	2.5394e-04	-1.013	8.5825e-04	-1.124	1.1176e-05	pubmed
ENSMUSG00000028445	Enho	-0.407	3.2029e-01	-2.244	2.0644e-06	-1.775	2.0376e-03	pubmed
ENSMUSG00000028453	Fancg	-1.566	1.8551e-04	-2.313	1.6500e-02	-1.051	2.0831e-01	pubmed
ENSMUSG00000028467	Gba2	-1.286	2.1750e-08	-1.472	4.1825e-06	-0.507	1.4477e-01	pubmed
ENSMUSG00000028479	Gne	-1.299	9.8769e-10	-1.551	1.0531e-10	-1.435	3.8519e-11	pubmed
ENSMUSG00000028497	Hacd4	-2.613	4.9252e-05	-2.630	7.0155e-04	-1.137	1.2091e-01	pubmed
ENSMUSG00000028536	2610528J11Rik	-3.997	6.5804e-13	-3.495	4.0373e-09	-2.677	1.1972e-02	pubmed
ENSMUSG00000028550	Atg4c	-0.965	5.3005e-04	-1.345	3.6708e-05	-1.241	4.5313e-03	pubmed
ENSMUSG00000028551	Cdkn2c	-1.214	8.4580e-05	-0.973	1.0886e-02	-1.131	1.3272e-02	pubmed
ENSMUSG00000028600	Podn	-1.540	9.9464e-06	0.264	8.1859e-01	-0.480	5.9621e-01	pubmed
ENSMUSG00000028621	Cyb5rl	-0.631	1.7931e-02	-1.005	9.4021e-04	-0.388	3.0681e-01	pubmed
ENSMUSG00000028653	Trit1	-1.161	6.2975e-09	-1.297	2.7764e-08	-0.827	4.4656e-03	pubmed
ENSMUSG00000028654	Mycl	-2.772	1.0579e-10	-2.544	8.9305e-11	-0.777	2.1709e-01	pubmed
ENSMUSG00000028698	Pik3r3	-0.904	1.4419e-02	-1.816	1.2108e-03	-0.402	4.8813e-01	pubmed
ENSMUSG00000028712	Cyp4a31	-0.779	5.0757e-01	-0.189	7.6956e-01	-1.407	3.2800e-03	pubmed
ENSMUSG00000028713	Cyp4b1	-0.695	1.1174e-03	-1.600	2.3828e-05	-0.399	4.4669e-01	pubmed
ENSMUSG00000028795	Ccdc28b	-1.097	6.0873e-03	-1.541	3.9775e-03	-0.489	5.4710e-01	pubmed
ENSMUSG00000028803	Nipal3	-1.917	2.2713e-13	-1.958	3.7400e-10	-1.165	1.7379e-02	pubmed
ENSMUSG00000028838	Extl1	-4.594	3.6334e-16	-2.341	1.9817e-02	-0.432	8.3551e-01	pubmed
ENSMUSG00000028860	Sytl1	-1.129	3.1323e-03	-1.665	1.0705e-03	-0.480	4.4636e-01	pubmed
ENSMUSG00000028909	Ptpru	-1.759	3.2933e-03	-0.552	6.3926e-01	0.797	4.2932e-01	pubmed
ENSMUSG00000028914	Casp9	-1.118	1.0225e-11	-1.711	1.1441e-10	-0.983	3.7970e-04	pubmed
ENSMUSG00000028919	Arhgef19	-2.826	1.2571e-57	-2.849	1.0153e-16	-1.782	1.8698e-03	pubmed
ENSMUSG00000028944	Prkag2	-0.554	8.6913e-03	-1.132	6.9368e-05	-0.752	1.1092e-02	pubmed
ENSMUSG00000028957	Per3	-3.756	6.1267e-19	-3.730	1.6017e-13	-1.739	1.5398e-01	pubmed
ENSMUSG00000028976	Slc2a5	-3.678	1.1018e-10	-3.506	2.7688e-12	-2.202	1.0149e-02	pubmed
ENSMUSG00000029020	Mfn2	-0.772	3.9644e-17	-1.014	1.1229e-12	-0.796	1.2806e-04	pubmed
ENSMUSG00000029032	Arhgef16	-1.232	4.3014e-03	-0.917	1.3981e-01	-0.560	5.4653e-01	pubmed
ENSMUSG00000029047	Pex10	-1.452	9.0285e-09	-0.917	5.3947e-02	-0.514	3.1653e-01	pubmed
ENSMUSG00000029053	Prkcz	-2.117	5.9869e-23	-1.936	1.4226e-04	-1.189	4.1431e-03	pubmed
ENSMUSG00000029060	Mib2	-1.029	1.0201e-11	-1.230	2.1944e-04	-0.556	4.7144e-02	pubmed
ENSMUSG00000029070	Mxra8	-1.047	1.3067e-04	-0.813	1.4815e-01	-0.369	5.9562e-01	pubmed
ENSMUSG00000029095	Ablim2	-1.747	2.5468e-05	-1.098	1.8078e-01	-0.141	8.3730e-01	pubmed
ENSMUSG00000029096	Htra3	-3.182	2.4471e-10	-1.585	8.6469e-02	-0.944	2.9625e-01	pubmed
ENSMUSG00000029161	Cgref1	-1.435	2.2531e-05	-0.942	2.8562e-01	-0.230	7.8528e-01	pubmed
ENSMUSG00000029177	Cenpa	-1.295	8.1378e-03	-1.340	3.1218e-02	-0.801	9.3892e-02	pubmed
ENSMUSG00000029189	Sel1l3	-1.143	7.7347e-10	-0.738	2.6691e-03	-0.294	4.1411e-01	pubmed
ENSMUSG00000029195	Klb	-2.285	2.4147e-04	-2.629	2.2469e-05	-2.382	1.1416e-08	pubmed
ENSMUSG00000029254	Stap1	-2.271	4.0868e-04	-2.644	1.6763e-05	-1.581	3.3654e-02	pubmed
ENSMUSG00000029270	Dipk1a	-0.097	8.5515e-01	-0.332	4.9940e-01	-1.096	4.8653e-03	pubmed
ENSMUSG00000029291	Rufy3	-1.107	1.2174e-26	-1.362	5.2403e-15	-0.831	1.0986e-02	pubmed
ENSMUSG00000029299	Abcg3	-3.159	5.6622e-09	-2.743	6.8734e-06	-1.338	1.8980e-01	pubmed
ENSMUSG00000029314	Gpat3	-1.305	1.1740e-03	-0.145	8.5285e-01	-0.133	9.1691e-01	pubmed
ENSMUSG00000029370	Rassf6	-2.060	2.4212e-19	-1.348	1.9211e-03	-0.450	2.5075e-01	pubmed
ENSMUSG00000029408	Abcb9	-1.597	1.1130e-03	-1.590	3.1963e-02	-0.368	5.9311e-01	pubmed
ENSMUSG00000029428	Stx2	-1.954	6.1792e-22	-2.744	1.1804e-12	-1.428	5.9576e-04	pubmed
ENSMUSG00000029438	Bcl7a	-1.641	1.4483e-21	-1.563	2.0852e-05	-0.817	1.7343e-02	pubmed
ENSMUSG00000029516	Cit	-1.137	8.7276e-03	-0.805	4.7158e-01	-0.080	9.6266e-01	pubmed
ENSMUSG00000029521	Chek2	-1.548	3.0025e-02	-2.236	7.5160e-03	-1.305	1.5796e-01	pubmed
ENSMUSG00000029524	Sirt4	-1.005	4.9445e-05	-1.195	4.5237e-03	-0.923	7.0012e-02	pubmed
ENSMUSG00000029556	Hnf1a	-1.078	1.8072e-08	-0.819	4.2870e-05	-0.254	5.2757e-01	pubmed
ENSMUSG00000029557	Mrm2	-0.522	5.0628e-02	-1.202	3.6012e-04	-0.339	4.4677e-01	pubmed
ENSMUSG00000029563	Foxp2	-2.981	3.2479e-12	-2.006	1.0523e-04	-0.860	2.3268e-01	pubmed
ENSMUSG00000029575	Mmab	-0.781	2.4113e-07	-1.304	3.4586e-09	-0.894	1.7733e-02	pubmed
ENSMUSG00000029600	Rita1	-1.969	3.7503e-05	-1.857	1.9737e-03	-0.938	1.9243e-01	pubmed
ENSMUSG00000029650	Slc46a3	-1.303	4.9484e-06	-1.690	5.3216e-12	-1.479	5.5828e-08	pubmed
ENSMUSG00000029657	Hsph1	-0.795	1.2010e-02	-1.763	3.2153e-04	-0.977	3.3368e-02	pubmed
ENSMUSG00000029675	Eln	-1.228	6.9674e-03	-1.510	1.4163e-01	-0.109	9.2405e-01	pubmed
ENSMUSG00000029710	Ephb4	-1.066	8.4441e-11	-0.796	5.3571e-04	-0.297	3.5757e-01	pubmed
ENSMUSG00000029722	Agfg2	-1.617	6.3720e-14	-2.439	2.1667e-18	-1.197	1.1962e-03	pubmed
ENSMUSG00000029727	Cyp3a13	-1.765	8.4948e-16	-1.648	6.0370e-22	-1.593	1.7825e-08	pubmed
ENSMUSG00000029859	Epha1	-1.449	6.0502e-10	-1.264	3.8413e-12	-0.578	1.1802e-01	pubmed
ENSMUSG00000030008	Pradc1	-0.998	1.0909e-03	-1.393	1.2087e-03	-0.609	2.1390e-01	pubmed
ENSMUSG00000030030	1700003E16Rik	-3.010	6.8689e-05	-2.312	2.5580e-02	-2.450	4.8106e-02	pubmed
ENSMUSG00000030032	Wdr54	-1.952	8.8879e-03	-1.703	2.7666e-01	-0.717	5.4571e-01	pubmed
ENSMUSG00000030087	Klf15	-0.917	9.4116e-03	-1.207	3.1274e-04	-1.314	8.7292e-02	pubmed
ENSMUSG00000030089	Slc41a3	-1.481	1.1634e-07	-2.084	2.2929e-03	-1.764	3.3288e-04	pubmed
ENSMUSG00000030123	Plxnd1	-1.332	8.2481e-06	-1.625	3.8667e-09	-0.467	3.6833e-01	pubmed
ENSMUSG00000030157	Clec2d	-0.748	2.4189e-03	-1.902	7.2785e-10	-2.261	7.3048e-11	pubmed
ENSMUSG00000030168	Adipor2	-1.072	7.8329e-09	-0.682	7.7846e-03	-0.485	4.1406e-02	pubmed

		CLP1		CLP2		CLP3		
Ensg	Symbol	LFC	FDR	LFC	FDR	LFC	FDR	Link
ENSMUSG00000030200	Bcl2l14	-1.376	3.0998e-02	-3.007	4.5723e-04	-1.990	5.0501e-02	pubmed
ENSMUSG00000030207	Fam234b	-1.100	7.4513e-20	-1.130	2.6083e-10	-1.113	5.8072e-06	pubmed
ENSMUSG00000030217	Art4	-2.687	3.4616e-06	-1.850	8.4834e-03	-0.805	4.3355e-01	pubmed
ENSMUSG00000030236	Slco1b2	-0.848	1.3739e-03	-0.410	1.8656e-01	-1.354	1.0302e-04	pubmed
ENSMUSG00000030244	Gys2	-0.907	5.2501e-03	-1.323	9.3496e-07	-0.717	3.4606e-02	pubmed
ENSMUSG00000030257	Srgap3	-1.586	2.2297e-02	-1.928	6.2405e-03	-1.249	1.0638e-01	pubmed
ENSMUSG00000030284	Creld1	-0.889	1.4572e-05	-1.314	4.7292e-03	-1.010	1.5439e-02	pubmed
ENSMUSG00000030322	Mbd4	-2.627	1.4481e-07	-1.215	7.7444e-02	-0.699	2.3697e-01	pubmed
ENSMUSG00000030340	Scnn1a	-1.010	1.5941e-10	-1.070	2.1678e-04	-0.999	5.9821e-03	pubmed
ENSMUSG00000030409	Dmpk	-1.506	6.1293e-09	-0.529	3.7817e-01	-0.381	2.3384e-01	pubmed
ENSMUSG00000030486	Zfp108	-2.587	2.8709e-05	-1.072	2.1476e-01	-0.921	2.5344e-01	pubmed
ENSMUSG00000030494	Rhpn2	-1.304	8.6518e-05	-1.136	1.7472e-02	-0.357	3.4121e-01	pubmed
ENSMUSG00000030551	Nr2f2	-1.906	1.3775e-21	-1.528	7.4658e-09	-0.545	2.1201e-01	pubmed
ENSMUSG00000030555	Ttc23	-1.857	6.0177e-07	-0.907	6.5473e-02	-0.177	6.6602e-01	pubmed
ENSMUSG00000030583	Sipa1l3	-0.636	3.6223e-03	-1.121	1.2550e-03	0.173	7.0809e-01	pubmed
ENSMUSG00000030614	Tmem126b	-0.801	5.0191e-06	-1.031	2.2629e-03	-1.052	2.2176e-03	pubmed
ENSMUSG00000030670	Cyp2r1	-0.266	5.8017e-01	-0.670	3.3991e-02	-1.283	1.2737e-05	pubmed
ENSMUSG00000030671	Pde3b	-0.899	1.9674e-10	-1.053	1.3939e-09	-0.676	1.4333e-02	pubmed
ENSMUSG00000030729	Pgm2l1	-1.138	2.1112e-04	-1.670	9.5902e-08	-1.008	2.7658e-02	pubmed
ENSMUSG00000030737	Slco2b1	-0.991	1.7819e-07	-1.019	2.9317e-03	-0.518	2.0761e-01	pubmed
ENSMUSG00000030761	Myo7a	-1.182	2.1289e-11	-1.116	5.6013e-04	-0.280	5.1546e-01	pubmed
ENSMUSG00000030763	Lcmt1	-1.115	7.5847e-06	-0.651	2.8326e-02	-0.577	8.3086e-02	pubmed
ENSMUSG00000030772	Dkk3	-1.071	7.6971e-03	-0.348	6.0632e-01	-0.760	1.9253e-01	pubmed
ENSMUSG00000030800	Prss8	-1.946	4.2566e-07	-1.848	9.0839e-05	-0.215	7.9231e-01	pubmed
ENSMUSG00000030826	Bcat2	-1.880	7.4604e-12	-2.875	3.3570e-11	-1.344	4.9031e-04	pubmed
ENSMUSG00000030827	Fgf21	-1.866	3.4559e-03	0.021	9.8641e-01	1.108	4.4448e-01	pubmed
ENSMUSG00000030834	Abcc6	-1.037	1.6448e-13	-1.075	3.8589e-03	-1.038	7.4078e-03	pubmed
ENSMUSG00000030865	Chp2	-1.635	6.4724e-04	-0.814	1.0699e-01	-0.259	8.1479e-01	pubmed
ENSMUSG00000030909	Anks4b	-2.133	1.2559e-20	-1.442	2.2567e-03	-0.849	1.0515e-01	pubmed
ENSMUSG00000030934	Oat	-0.464	8.4032e-02	-0.428	3.8799e-02	-1.045	4.8180e-07	pubmed
ENSMUSG00000030935	Acsm3	-1.066	2.4604e-11	-0.641	8.6428e-03	-1.045	1.0489e-04	pubmed
ENSMUSG00000030946	Lhpp	-0.633	2.1752e-02	-0.922	6.5662e-04	-1.313	6.1372e-08	pubmed
ENSMUSG00000030956	Fam53b	-1.060	4.5734e-09	-1.625	6.8591e-07	-0.746	1.0657e-02	pubmed
ENSMUSG00000030979	Uros	-0.848	4.6158e-09	-0.903	1.6500e-02	-1.063	3.9514e-03	pubmed
ENSMUSG00000031016	Wee1	-1.779	1.8293e-06	-1.049	1.5714e-01	0.091	9.2130e-01	pubmed
ENSMUSG00000031072	LTO1	-1.093	2.6776e-08	-0.831	4.1285e-02	-0.536	1.6044e-01	pubmed
ENSMUSG00000031129	Slc9a9	-0.396	4.0560e-01	-0.438	6.4301e-01	-1.494	3.9136e-04	pubmed
ENSMUSG00000031147	Magix	-1.186	9.7298e-05	-2.590	4.1217e-10	-1.755	1.8863e-03	pubmed
ENSMUSG00000031216	Stard8	-1.999	6.6610e-13	-1.417	4.8867e-06	-0.768	2.9789e-02	pubmed
ENSMUSG00000031217	Efnb1	-1.032	8.3270e-08	-0.826	2.4331e-03	-0.333	4.2286e-01	pubmed
ENSMUSG00000031227	Magee1	-0.640	8.5366e-02	-1.539	4.5540e-03	-0.088	9.0616e-01	pubmed
ENSMUSG00000031286	Glt28d2	-1.389	3.5294e-04	-1.121	8.3372e-04	-0.747	8.3467e-02	pubmed
ENSMUSG00000031292	Cdkl5	-1.125	1.6659e-04	-0.517	3.6663e-01	-0.222	6.8035e-01	pubmed
ENSMUSG00000031295	Phka2	-1.051	3.8201e-05	-0.807	2.3566e-02	-0.596	2.4772e-02	pubmed
ENSMUSG00000031378	Abcd1	-1.123	5.8893e-13	-1.482	1.4304e-09	-0.953	1.6740e-03	pubmed
ENSMUSG00000031399	Fam3a	-1.054	1.5683e-10	-1.401	7.5660e-06	-1.074	3.1387e-02	pubmed
ENSMUSG00000031431	Tsc22d3	-1.559	3.5584e-10	-1.229	1.8568e-05	-1.332	1.7952e-02	pubmed
ENSMUSG00000031445	Proz	-0.719	4.0337e-06	-0.727	1.4180e-02	-1.009	1.4474e-03	pubmed
ENSMUSG00000031486	Adgra2	-1.526	5.8340e-11	-1.502	7.0701e-05	-0.407	2.8959e-01	pubmed
ENSMUSG00000031489	Adrb3	-2.687	1.0530e-09	-2.372	5.9063e-03	-1.466	1.3660e-02	pubmed
ENSMUSG00000031508	Ankrd10	-1.091	5.0198e-09	-0.869	3.9627e-02	-0.531	1.0040e-01	pubmed
ENSMUSG00000031523	Dlc1	-0.855	1.7664e-11	-1.032	2.8785e-08	-0.412	1.6804e-01	pubmed
ENSMUSG00000031539	Ap3m2	-1.332	6.4270e-04	-1.070	8.9779e-02	-0.445	5.3080e-01	pubmed
ENSMUSG00000031549	Ido2	-1.404	1.8817e-06	-1.735	1.8892e-23	-1.534	1.6529e-06	pubmed
ENSMUSG00000031561	Tenm3	-1.334	1.5630e-05	-1.324	5.8780e-05	-1.234	3.0238e-03	pubmed
ENSMUSG00000031618	Nr3c2	-1.705	2.9643e-05	-2.097	1.3004e-05	-1.174	3.8197e-02	pubmed
ENSMUSG00000031626	Sorbs2	0.978	5.0989e-03	-0.658	1.2520e-01	-1.167	2.6435e-03	pubmed
ENSMUSG00000031661	Nkd1	-1.102	1.7800e-04	-0.871	1.4351e-01	-0.380	6.0824e-01	pubmed
ENSMUSG00000031665	Sall1	-1.304	1.9573e-04	-1.276	1.4426e-03	-1.321	2.2920e-02	pubmed
ENSMUSG00000031767	Nudt7	-1.130	3.0831e-08	-0.129	8.4105e-01	-0.771	9.6819e-02	pubmed
ENSMUSG00000031775	Pllp	-1.970	2.0339e-03	-1.270	1.2799e-01	-0.923	3.3763e-01	pubmed
ENSMUSG00000031790	Mmp15	-1.591	2.7298e-07	-2.333	7.2874e-06	-1.604	1.8823e-03	pubmed
ENSMUSG00000031808	Slc27a1	-1.058	3.1243e-07	-0.456	3.9270e-01	0.018	9.7884e-01	pubmed
ENSMUSG00000031822	Gse1	-1.786	5.9970e-14	-1.659	2.0526e-06	-0.565	4.5300e-01	pubmed
ENSMUSG00000031833	Mast3	-1.512	1.0381e-12	-1.649	2.0836e-22	-0.169	7.5931e-01	pubmed
ENSMUSG00000031840	Rab3a	-2.361	5.0463e-07	-1.809	3.3060e-03	-0.579	3.5333e-01	pubmed
ENSMUSG00000031842	Pde4c	-1.309	1.1568e-07	-1.168	3.2243e-02	-0.831	5.0702e-02	pubmed
ENSMUSG00000031851	Ntpcr	-1.476	2.8495e-07	-0.984	3.6095e-03	-0.778	4.1164e-02	pubmed
ENSMUSG00000031853	Map3k21	-1.317	9.5440e-04	-1.399	7.0444e-04	-0.343	3.7469e-01	pubmed
ENSMUSG00000031864	Ints10	-0.408	2.1685e-01	-1.037	3.7843e-03	-0.587	1.4314e-01	pubmed
ENSMUSG00000031903	Pla2g15	-1.357	3.8834e-16	-1.576	1.3559e-10	-0.868	3.4073e-03	pubmed
ENSMUSG00000031906	Smpd3	-1.711	1.3593e-04	-1.589	7.8012e-05	0.387	8.0183e-01	pubmed
ENSMUSG00000031949	Adat1	-1.030	1.1554e-06	-1.020	1.6378e-01	-0.182	7.3939e-01	pubmed
ENSMUSG00000031955	Bcar1	-0.763	3.5673e-04	-1.015	1.4379e-04	0.032	9.3735e-01	pubmed
ENSMUSG00000032009	Sesn3	-0.649	9.2761e-03	-1.147	4.4594e-09	-0.660	2.8836e-02	pubmed
ENSMUSG00000032010	Usp2	-1.702	7.4286e-07	-0.880	1.5819e-01	-0.041	9.5717e-01	pubmed
ENSMUSG00000032014	Oaf	-2.112	2.0433e-19	-2.719	3.9977e-20	-1.744	4.9226e-02	pubmed
ENSMUSG00000032028	Nxpe2	-0.468	4.1356e-01	-1.087	9.8873e-02	-1.943	6.3776e-04	pubmed
ENSMUSG00000032064	Dixdc1	-2.242	1.6612e-14	-2.370	1.7348e-08	-1.516	5.1368e-03	pubmed
ENSMUSG00000032066	Bco2	-0.845	1.8726e-02	-0.671	1.8615e-01	-1.178	3.0490e-04	pubmed
ENSMUSG00000032080	Apoa4	-1.154	4.2832e-03	-1.189	1.8219e-01	-1.089	6.5542e-02	pubmed
ENSMUSG00000032086	Bace1	-1.387	1.5027e-20	-1.252	6.6550e-06	-0.976	1.1581e-02	pubmed
ENSMUSG00000032092	Mpzl2	-1.795	8.6612e-06	-1.448	2.9326e-06	-1.153	4.6647e-02	pubmed
ENSMUSG00000032120	C2cd2l	-1.058	7.2644e-11	-0.372	3.7597e-01	0.392	3.3887e-02	pubmed
ENSMUSG00000032122	Slc37a2	-1.438	3.4569e-04	-2.093	1.0482e-02	-0.801	2.7409e-01	pubmed
ENSMUSG00000032177	Pde4a	-1.162	4.3420e-03	-2.292	3.5392e-05	-1.032	1.1362e-01	pubmed
ENSMUSG00000032179	Bmp5	-1.125	7.0065e-04	-0.250	6.8556e-01	-0.052	9.4658e-01	pubmed
ENSMUSG00000032180	Tmed1	-0.754	1.7873e-03	-1.139	1.2892e-03	-0.764	6.7305e-02	pubmed
ENSMUSG00000032194	Kank2	-2.047	1.5270e-19	-2.161	4.3463e-07	-0.847	1.0780e-02	pubmed
ENSMUSG00000032198	Dock6	-1.200	6.5818e-13	-0.587	1.5535e-01	-0.119	7.3817e-01	pubmed
ENSMUSG00000032204	Aqp9	-1.959	6.2134e-09	-2.175	6.9390e-65	-2.201	4.3315e-11	pubmed

Ensg	Symbol	CLP1 LFC	CLP1 FDR	CLP2 LFC	CLP2 FDR	CLP3 LFC	CLP3 FDR	Link
ENSMUSG00000032220	Myo1e	-0.509	8.8645e-02	-1.056	2.8654e-04	-1.262	2.6015e-04	pubmed
ENSMUSG00000032246	Calml4	-3.953	1.3774e-03	0.472	7.0834e-01	-0.556	6.0686e-01	pubmed
ENSMUSG00000032298	Neil1	-1.024	1.5397e-04	-1.632	1.2712e-03	-0.772	1.3705e-01	pubmed
ENSMUSG00000032312	Csk	-0.744	1.1043e-05	-1.367	2.4585e-15	-0.751	2.8623e-03	pubmed
ENSMUSG00000032363	Adamts7	-0.873	3.2659e-02	-1.830	9.2740e-03	-0.649	4.1893e-01	pubmed
ENSMUSG00000032377	Plscr4	-1.943	2.5594e-06	-1.303	2.4570e-02	-0.793	1.6610e-01	pubmed
ENSMUSG00000032380	Dapk2	-1.286	4.5813e-05	-1.802	9.9526e-05	-0.549	4.2715e-01	pubmed
ENSMUSG00000032402	Smad3	-1.125	2.3523e-05	-1.376	4.0495e-05	-0.392	5.0333e-01	pubmed
ENSMUSG00000032403	2300009A05Rik	-1.317	7.6795e-04	-0.787	1.1427e-01	-0.393	4.6240e-01	pubmed
ENSMUSG00000032470	Mras	-1.608	1.9367e-04	-1.966	1.0394e-03	-1.267	9.8219e-02	pubmed
ENSMUSG00000032492	Pth1r	-3.001	1.0467e-09	-1.978	3.1962e-02	-1.547	1.3002e-02	pubmed
ENSMUSG00000032500	Dclk3	-2.215	2.1756e-04	-0.732	5.0294e-01	0.123	9.2805e-01	pubmed
ENSMUSG00000032513	Gorasp1	-1.179	4.9087e-11	-1.294	1.8364e-04	-0.286	4.8903e-01	pubmed
ENSMUSG00000032528	Vipr1	-1.732	2.4250e-27	-1.059	1.8512e-04	-0.778	5.2068e-02	pubmed
ENSMUSG00000032531	Amotl2	-0.599	2.6698e-04	-0.418	3.7003e-01	-1.100	1.3683e-04	pubmed
ENSMUSG00000032549	Rab6b	-1.521	4.5081e-03	-2.270	5.8404e-03	-0.745	4.9192e-01	pubmed
ENSMUSG00000032598	Nckipsd	-0.891	9.1484e-04	-1.677	2.0193e-06	-0.525	1.6129e-01	pubmed
ENSMUSG00000032606	Nicn1	-1.156	3.4527e-08	-1.269	6.4978e-05	-1.194	3.4078e-03	pubmed
ENSMUSG00000032607	Amt	-0.691	1.3697e-03	-0.983	3.4389e-04	-1.136	2.0471e-03	pubmed
ENSMUSG00000032641	Gpr19	-1.074	7.6971e-03	-0.360	4.7535e-01	0.093	8.7169e-01	pubmed
ENSMUSG00000032702	Kank1	-1.441	2.3188e-32	-1.224	8.5068e-09	-0.240	4.4156e-01	pubmed
ENSMUSG00000032718	Mansc1	-1.334	6.2698e-03	0.091	9.4071e-01	0.173	8.6727e-01	pubmed
ENSMUSG00000032724	Abtb2	-1.083	2.4587e-05	-1.825	2.1036e-05	-0.365	4.1503e-01	pubmed
ENSMUSG00000032733	Snx33	-1.299	7.3500e-12	-1.535	8.3503e-07	-0.851	1.8979e-01	pubmed
ENSMUSG00000032737	Inppl1	-0.676	6.2875e-06	-1.384	3.9198e-07	-0.430	3.4704e-01	pubmed
ENSMUSG00000032741	Tpcn1	-2.112	6.5869e-46	-1.945	1.5649e-23	-1.620	9.0771e-07	pubmed
ENSMUSG00000032754	Slc8b1	-0.974	2.8042e-04	-1.524	6.7659e-13	-1.163	1.5949e-06	pubmed
ENSMUSG00000032782	Cntrob	-1.594	1.2085e-10	-1.425	6.7797e-04	-0.536	4.6692e-01	pubmed
ENSMUSG00000032827	Ppp1r9a	-1.109	1.4269e-06	-1.074	5.8799e-05	-1.155	1.1408e-02	pubmed
ENSMUSG00000032883	Acsl3	-0.720	8.9828e-03	-1.073	9.7796e-08	-0.487	4.0783e-02	pubmed
ENSMUSG00000032898	Fbxo21	-2.130	5.9869e-23	-2.196	1.0275e-45	-0.763	9.8883e-03	pubmed
ENSMUSG00000032946	Rasgrp2	-1.753	3.9548e-13	-0.833	5.5725e-02	-0.553	2.6252e-01	pubmed
ENSMUSG00000033060	Lmo7	-2.370	6.7444e-35	-1.584	1.8842e-04	-0.913	1.9474e-01	pubmed
ENSMUSG00000033083	Tbc1d4	-1.076	3.1937e-03	-0.781	2.4252e-02	-0.407	3.8274e-01	pubmed
ENSMUSG00000033102	Cdc14b	-1.107	3.9573e-06	-1.076	2.5640e-07	-0.600	8.6679e-02	pubmed
ENSMUSG00000033111	3830406C13Rik	-0.783	1.5927e-05	-1.068	7.9121e-05	-1.130	1.8539e-04	pubmed
ENSMUSG00000033149	Phldb2	-1.055	2.3188e-06	-1.316	8.1523e-12	-1.426	4.2111e-03	pubmed
ENSMUSG00000033170	Card10	-1.400	2.9186e-14	-1.459	4.5524e-07	-0.747	1.3595e-01	pubmed
ENSMUSG00000033174	Mgll	-2.017	1.5922e-12	-1.340	1.0338e-04	-1.146	7.5134e-04	pubmed
ENSMUSG00000033209	Ttc28	-1.963	1.5188e-07	-0.909	1.5609e-01	-0.255	7.6118e-01	pubmed
ENSMUSG00000033233	Trim45	-2.097	2.5145e-03	-2.588	1.4992e-02	-0.564	6.1275e-01	pubmed
ENSMUSG00000033249	Hsf4	-1.847	5.2363e-08	-0.567	2.1455e-01	0.232	6.8181e-01	pubmed
ENSMUSG00000033256	Shf	-1.176	2.1162e-02	-1.315	3.4480e-03	-0.690	2.6213e-01	pubmed
ENSMUSG00000033313	Fbxl8	-1.530	2.7644e-04	-1.767	5.6597e-03	-0.718	4.7853e-01	pubmed
ENSMUSG00000033318	Gstt2	-1.094	2.3523e-05	-0.621	1.2704e-03	-0.471	1.9834e-01	pubmed
ENSMUSG00000033460	Armcx1	-1.413	1.0172e-05	-1.628	1.0683e-02	-1.089	1.3268e-01	pubmed
ENSMUSG00000033486	Catsper2	-1.048	1.0932e-03	-0.571	3.4000e-01	-0.756	1.3458e-01	pubmed
ENSMUSG00000033589	Reep4	-0.950	9.6797e-04	-1.204	1.0869e-03	-0.200	6.7316e-01	pubmed
ENSMUSG00000033594	Spata2l	-2.680	1.4367e-09	-2.590	1.6249e-20	-1.317	7.5703e-03	pubmed
ENSMUSG00000033610	Pank1	-1.244	1.3845e-04	-0.842	2.9213e-03	-0.878	1.0167e-02	pubmed
ENSMUSG00000033705	Stard9	-1.178	7.7585e-07	-0.772	8.6597e-02	-0.411	4.2584e-01	pubmed
ENSMUSG00000033720	Sfxn5	-1.310	1.5843e-11	-1.071	1.9914e-04	-0.828	1.8537e-02	pubmed
ENSMUSG00000033763	Mtss1l	-2.452	1.7400e-08	-2.371	1.2869e-04	0.195	8.8647e-01	pubmed
ENSMUSG00000033781	Asb13	-0.538	7.8282e-03	-1.185	8.8289e-10	-0.911	8.6485e-05	pubmed
ENSMUSG00000033871	Ppargc1b	-2.639	3.1043e-06	-1.327	1.1695e-01	-0.173	8.2593e-01	pubmed
ENSMUSG00000033900	Map9	-2.054	9.3937e-03	-1.626	6.7062e-02	-0.114	9.2964e-01	pubmed
ENSMUSG00000033985	Tesk2	-0.560	1.7750e-01	-0.709	6.0140e-02	-1.128	7.0735e-06	pubmed
ENSMUSG00000034006	Pqlc1	-1.211	7.1573e-08	-0.689	1.1025e-02	0.192	5.2673e-01	pubmed
ENSMUSG00000034035	Ccdc17	-1.030	6.1241e-03	-0.968	1.1916e-01	-0.982	1.1159e-01	pubmed
ENSMUSG00000034071	Zfp551	-2.122	1.8528e-03	-0.997	1.8624e-01	-0.491	5.2079e-01	pubmed
ENSMUSG00000034177	Rnf43	-2.692	9.9443e-31	-2.082	1.1220e-12	-0.840	7.7232e-02	pubmed
ENSMUSG00000034220	Gpc1	-1.099	3.2892e-03	-2.106	3.9233e-05	-0.598	2.7756e-01	pubmed
ENSMUSG00000034254	Agpat1	-1.045	4.2576e-06	-1.018	3.5896e-02	-0.924	1.3702e-01	pubmed
ENSMUSG00000034258	Flvcr2	-1.751	4.9682e-04	-3.081	1.3621e-03	-1.362	7.2821e-02	pubmed
ENSMUSG00000034265	Zdhhc14	-1.027	3.9711e-02	-1.347	3.4537e-03	-0.686	1.5383e-01	pubmed
ENSMUSG00000034353	Ramp1	-1.394	1.3763e-05	-0.382	4.9639e-01	-0.714	1.3806e-01	pubmed
ENSMUSG00000034382	AI661453	-0.888	2.8329e-03	-1.734	1.6198e-15	-0.212	7.6078e-01	pubmed
ENSMUSG00000034429	Zfp707	-1.304	4.1987e-09	-1.111	1.5628e-05	-0.972	1.3013e-04	pubmed
ENSMUSG00000034435	Tmem30b	-1.259	1.4319e-23	-1.290	3.2806e-10	-0.912	1.7472e-02	pubmed
ENSMUSG00000034445	Cyb561a3	-1.470	2.3123e-11	-0.782	3.2683e-02	0.045	9.4297e-01	pubmed
ENSMUSG00000034522	Zfp395	-2.213	1.7349e-10	-1.816	1.0569e-01	-1.034	9.0298e-02	pubmed
ENSMUSG00000034538	Zfp418	-1.155	8.4552e-03	0.360	6.4081e-01	-0.135	8.6203e-01	pubmed
ENSMUSG00000034584	Exph5	-1.560	1.2901e-07	-0.834	7.2675e-02	-0.835	1.2994e-01	pubmed
ENSMUSG00000034601	2700049A03Rik	-1.507	4.1327e-07	-1.208	1.3532e-02	-0.773	1.3119e-02	pubmed
ENSMUSG00000034641	Cd300ld	-0.130	7.8049e-01	-0.476	2.4852e-01	-1.121	2.4653e-03	pubmed
ENSMUSG00000034673	Pbx2	-0.411	2.3934e-02	-1.155	2.3279e-04	-0.817	1.7379e-02	pubmed
ENSMUSG00000034714	Ttyh2	-0.885	8.6255e-11	-1.421	2.1948e-09	-0.719	1.6129e-01	pubmed
ENSMUSG00000034800	Zfp661	-1.299	2.7388e-07	-0.952	9.7595e-02	-0.808	6.5400e-02	pubmed
ENSMUSG00000034848	Ttc21b	-0.816	9.8378e-03	-0.998	3.3265e-02	-1.237	4.5900e-03	pubmed
ENSMUSG00000034854	Mfsd12	-1.009	1.2179e-04	-1.209	2.2454e-02	-0.107	8.4162e-01	pubmed
ENSMUSG00000034858	Fam214a	-2.791	2.8951e-20	-2.601	7.5366e-21	-1.710	4.0927e-05	pubmed
ENSMUSG00000034867	Ankrd27	-0.600	4.5745e-03	-1.042	9.3711e-07	-0.460	1.7165e-01	pubmed
ENSMUSG00000034880	Mrpl34	-0.749	3.8361e-04	-1.195	2.4611e-05	-1.073	1.9068e-05	pubmed
ENSMUSG00000034881	Tbxa2r	-2.962	1.2236e-03	-1.844	2.1551e-01	-1.225	1	pubmed
ENSMUSG00000034911	Ushbp1	-2.297	5.4113e-12	-2.047	2.1611e-06	-1.099	4.8985e-02	pubmed
ENSMUSG00000034926	Dhcr24	-1.510	3.8071e-26	-2.032	5.0010e-12	-1.449	1.4995e-03	pubmed
ENSMUSG00000034930	Rtkn	-1.151	5.5577e-04	-2.224	4.6859e-04	-0.500	4.7139e-01	pubmed
ENSMUSG00000034936	Arl4d	-0.305	4.8003e-01	-0.872	1.6770e-02	-1.285	6.4101e-03	pubmed
ENSMUSG00000035064	Eef2k	-1.687	4.0994e-09	-1.480	1.0464e-04	-0.187	6.0105e-01	pubmed
ENSMUSG00000035104	Eva1a	-2.314	3.3617e-25	-1.888	8.4912e-29	-1.449	2.3546e-04	pubmed
ENSMUSG00000035112	Wnk4	-1.582	3.4732e-03	-1.403	2.5229e-01	-0.558	5.5884e-01	pubmed

		CLP1		CLP2		CLP3		
Ensg	Symbol	LFC	FDR	LFC	FDR	LFC	FDR	Link
ENSMUSG00000035172	Plekhh3	-1.491	3.1337e-08	-1.444	2.1202e-04	-0.497	5.8223e-01	pubmed
ENSMUSG00000035198	Tubg1	-0.849	5.4171e-06	-1.106	5.6719e-07	-0.818	4.8376e-04	pubmed
ENSMUSG00000035206	Sppl2b	-1.386	2.3284e-13	-1.828	1.5966e-06	-0.767	2.7658e-02	pubmed
ENSMUSG00000035283	Adrb1	-3.267	9.2463e-03	-0.105	9.4686e-01	-1.283	1	pubmed
ENSMUSG00000035342	Lzts2	-0.872	1.7309e-04	-1.185	1.8968e-03	-0.382	4.2343e-01	pubmed
ENSMUSG00000035367	Rmi1	-1.281	5.2539e-08	-0.913	2.5025e-02	-0.998	3.9569e-03	pubmed
ENSMUSG00000035397	Klf16	-0.435	4.8876e-01	-2.068	3.3989e-06	-0.464	6.6490e-01	pubmed
ENSMUSG00000035413	Tmem98	-0.975	1.2074e-04	-0.932	2.7732e-02	-1.017	6.0073e-03	pubmed
ENSMUSG00000035451	Foxa1	-1.292	1.0483e-11	-0.981	1.8831e-03	-0.671	4.3041e-02	pubmed
ENSMUSG00000035504	Reep6	-1.131	4.4456e-06	-1.203	5.4333e-07	-0.933	1.0520e-03	pubmed
ENSMUSG00000035547	Capn5	-1.980	1.4485e-08	-1.179	1.2246e-02	-0.638	3.8300e-01	pubmed
ENSMUSG00000035595	1600002K03Rik	-1.014	7.9897e-03	-0.847	7.5594e-02	-0.457	4.0332e-01	pubmed
ENSMUSG00000035629	Rubcn	-1.342	2.7211e-14	-1.428	3.6789e-03	-0.540	1.1362e-01	pubmed
ENSMUSG00000035697	Arhgap45	-0.663	1.3379e-03	-1.196	1.8042e-03	-0.571	2.5042e-01	pubmed
ENSMUSG00000035759	Bbs10	-1.575	3.7245e-06	-1.555	4.3979e-02	-1.419	6.5400e-02	pubmed
ENSMUSG00000035790	Cep19	-1.525	5.1248e-05	-1.820	9.5263e-04	-1.005	8.2245e-02	pubmed
ENSMUSG00000035834	Polr3g	-0.519	1.1787e-01	-1.733	5.0066e-08	-1.087	6.0073e-04	pubmed
ENSMUSG00000035842	Ddx11	-1.200	1.2755e-03	-2.135	1.2635e-06	-0.768	1.2464e-01	pubmed
ENSMUSG00000035868	Zfp983	-1.242	4.8660e-05	-1.426	2.1879e-02	-1.202	2.6061e-02	pubmed
ENSMUSG00000035875	AI182371	-0.712	1.1315e-04	-0.889	7.9631e-03	-1.122	4.8502e-04	pubmed
ENSMUSG00000035877	Zhx3	-0.805	3.6541e-07	-1.149	2.5173e-10	-0.685	1.5499e-03	pubmed
ENSMUSG00000035878	Hykk	-2.837	2.0835e-44	-3.001	1.5700e-04	-2.057	1	pubmed
ENSMUSG00000035944	Ttc38	-1.311	4.8311e-23	-1.681	7.6427e-07	-1.300	9.4109e-04	pubmed
ENSMUSG00000036022	Fam122b	-1.085	8.4351e-03	-1.040	6.9037e-02	-1.406	9.4882e-03	pubmed
ENSMUSG00000036027	1810046K07Rik	-4.551	2.9436e-03	-1.797	2.0200e-01	-0.371	8.1740e-01	pubmed
ENSMUSG00000036040	Adamtsl2	-1.135	3.6055e-06	-0.392	5.6897e-01	0.015	9.8513e-01	pubmed
ENSMUSG00000036078	Sigmar1	-1.473	2.7729e-23	-1.884	5.6278e-26	-1.386	1.0463e-03	pubmed
ENSMUSG00000036098	Myrf	-0.941	3.1424e-05	-1.280	3.3202e-08	-0.234	6.4683e-01	pubmed
ENSMUSG00000036136	Fam110c	-1.471	1.3853e-03	-0.347	6.1684e-01	-0.003	9.9820e-01	pubmed
ENSMUSG00000036158	Prickle1	-1.147	1.0146e-03	1.094	9.2678e-02	1.494	6.5693e-02	pubmed
ENSMUSG00000036181	Hist1h1c	-2.223	2.7244e-31	-2.485	1.3072e-16	-2.184	3.9961e-06	pubmed
ENSMUSG00000036295	Lrrn3	-3.002	5.8523e-03	-0.629	5.9920e-01	-0.289	1	pubmed
ENSMUSG00000036368	Rmdn2	-1.011	6.0512e-06	-0.218	4.3435e-01	-0.458	1.4125e-02	pubmed
ENSMUSG00000036528	Ppfibp2	-1.618	1.4010e-23	-1.933	1.5560e-11	-0.820	2.3626e-01	pubmed
ENSMUSG00000036555	Iqce	-1.656	6.3162e-10	-2.422	1.5558e-09	-0.721	1.7061e-01	pubmed
ENSMUSG00000036565	Ttyh3	-0.367	1.4595e-01	-1.025	9.7744e-03	-0.386	2.7651e-01	pubmed
ENSMUSG00000036585	Fgf1	-1.898	3.8494e-14	-1.137	6.1758e-04	-1.282	1.8200e-04	pubmed
ENSMUSG00000036617	Etl4	-1.430	5.2323e-09	-1.003	2.1575e-03	-0.504	3.5380e-01	pubmed
ENSMUSG00000036641	Ccdc148	-0.782	4.1082e-01	-0.908	2.9392e-01	-2.297	3.9569e-03	pubmed
ENSMUSG00000036687	Tmem184a	-1.676	5.7935e-14	-2.030	2.5156e-13	-1.358	8.6747e-03	pubmed
ENSMUSG00000036764	Dnajc12	-1.829	3.3302e-18	-1.892	6.3542e-09	-1.338	3.3926e-03	pubmed
ENSMUSG00000036775	Decr2	-0.827	1.1231e-04	-0.601	3.0773e-02	-1.093	1.1067e-03	pubmed
ENSMUSG00000036813	Entpd8	-1.769	5.4668e-15	-1.511	1.5203e-06	-1.524	2.7521e-06	pubmed
ENSMUSG00000036856	Wnt4	-1.497	1.1405e-03	0.237	8.3859e-01	-0.131	9.2117e-01	pubmed
ENSMUSG00000036863	Syde2	-2.098	7.7713e-08	-2.097	1.3353e-07	-1.728	1.0414e-03	pubmed
ENSMUSG00000036864	Proser3	-1.953	5.2248e-05	-1.996	3.1289e-02	-1.100	1.7526e-01	pubmed
ENSMUSG00000036875	Dna2	-1.301	1.5573e-11	-1.362	3.8508e-08	-0.758	1.1377e-01	pubmed
ENSMUSG00000036882	Arhgap33	-3.008	3.4025e-03	-2.253	1.5541e-01	-0.768	5.9282e-01	pubmed
ENSMUSG00000036934	4921524J17Rik	-0.993	1.0912e-03	-0.559	4.3843e-02	-1.013	1.2948e-03	pubmed
ENSMUSG00000036948	Map11	-0.445	1.1498e-02	-1.059	5.8555e-03	-0.574	2.8952e-01	pubmed
ENSMUSG00000036957	Lrfn3	-1.287	5.2882e-05	-1.393	3.6285e-03	-0.152	8.6423e-01	pubmed
ENSMUSG00000036959	Bcorl1	-1.194	1.4766e-06	-1.619	2.2307e-05	-0.924	8.1826e-02	pubmed
ENSMUSG00000036968	Cnpy4	-1.083	1.2283e-03	-1.296	1.1733e-03	-0.943	6.2078e-02	pubmed
ENSMUSG00000036975	Tmem177	-1.121	7.1406e-06	-1.117	6.8935e-03	-0.610	2.7849e-01	pubmed
ENSMUSG00000037020	Wdr62	-1.100	4.2333e-03	-0.850	2.2726e-01	-0.240	7.6213e-01	pubmed
ENSMUSG00000037025	Foxa2	-1.404	4.8950e-04	-1.141	1.6009e-03	-1.443	8.5520e-05	pubmed
ENSMUSG00000037031	Tspan15	-1.599	1.2104e-06	-0.516	3.8601e-01	-0.065	9.3904e-01	pubmed
ENSMUSG00000037206	Islr	-1.324	1.8912e-03	-0.336	7.2475e-01	-0.075	9.4569e-01	pubmed
ENSMUSG00000037243	Zfp692	-1.147	3.2701e-05	-1.224	9.6040e-03	-1.088	3.9076e-03	pubmed
ENSMUSG00000037251	Pomk	-1.064	4.5011e-04	-1.009	1.4423e-03	-0.869	1.0065e-02	pubmed
ENSMUSG00000037266	Rsrp1	-0.233	1.5179e-01	-0.800	5.5921e-03	-1.084	2.2622e-03	pubmed
ENSMUSG00000037306	Man1c1	-1.214	2.7629e-05	-0.851	9.5641e-02	-0.318	5.9321e-01	pubmed
ENSMUSG00000037347	Chst7	-1.731	2.9304e-02	-3.852	9.3987e-03	-0.178	1	pubmed
ENSMUSG00000037348	Paqr7	-4.376	4.0381e-47	-3.859	3.5680e-18	-1.939	4.1000e-02	pubmed
ENSMUSG00000037363	Letm2	-1.675	1.8506e-05	-0.964	1.7505e-01	-0.756	2.1933e-01	pubmed
ENSMUSG00000037370	Enpp1	-0.610	7.8929e-03	-1.013	3.8242e-04	-1.040	1.6508e-04	pubmed
ENSMUSG00000037379	Spon2	0.214	8.6480e-01	-1.171	8.4827e-02	-2.630	1.0010e-03	pubmed
ENSMUSG00000037415	Ranbp10	-1.218	4.3030e-49	-1.402	1.5293e-07	-0.736	1.8450e-04	pubmed
ENSMUSG00000037440	Vnn1	-0.603	1.5957e-01	-0.483	3.2669e-01	-1.284	2.3995e-03	pubmed
ENSMUSG00000037455	Slc18b1	-2.003	1.2494e-07	-1.711	5.0669e-03	-0.317	5.7266e-01	pubmed
ENSMUSG00000037523	Mavs	-1.471	3.7293e-36	-1.830	1.5355e-06	-1.290	5.9588e-03	pubmed
ENSMUSG00000037542	Aldh8a1	-1.800	2.0524e-24	-1.621	7.7230e-18	-1.568	1.0362e-03	pubmed
ENSMUSG00000037573	Tob1	-1.013	2.3810e-06	-1.119	1.1025e-03	-0.695	2.3411e-01	pubmed
ENSMUSG00000037583	Nr0b2	-0.254	7.8947e-01	-5.151	2.1196e-07	-4.215	5.4701e-08	pubmed
ENSMUSG00000037669	Ldah	-0.740	2.9761e-08	-1.187	4.2624e-04	-1.094	1.4323e-03	pubmed
ENSMUSG00000037685	Atp8a1	-0.675	1.4222e-02	-0.409	2.7450e-01	-1.011	6.7546e-03	pubmed
ENSMUSG00000037686	Aspg	-1.086	2.1505e-04	-1.037	4.4805e-04	-1.119	1.1946e-03	pubmed
ENSMUSG00000037703	Lzts3	-1.788	1.6727e-19	-2.017	1.4368e-04	-1.347	2.8630e-03	pubmed
ENSMUSG00000037709	Fam13a	-3.614	7.8900e-23	-4.292	1.4900e-20	-1.886	9.9749e-03	pubmed
ENSMUSG00000037752	Xkr8	-1.048	8.5151e-05	-1.705	8.5351e-04	-1.004	4.8487e-02	pubmed
ENSMUSG00000037754	Ppp1r16b	-1.323	9.2943e-04	-1.194	5.4995e-02	-0.119	9.2157e-01	pubmed
ENSMUSG00000037797	Adh4	-0.992	5.4062e-02	-0.891	8.0984e-02	-1.702	7.5233e-05	pubmed
ENSMUSG00000037826	Ppm1k	-1.387	6.0556e-05	-1.826	1.3590e-06	-1.708	3.8849e-04	pubmed
ENSMUSG00000037890	Wdr19	-0.891	3.7435e-02	-2.049	1.8107e-05	-0.492	3.8083e-01	pubmed
ENSMUSG00000037904	Ankrd9	-1.001	9.6987e-02	-2.832	2.1057e-03	-1.270	1.1011e-01	pubmed
ENSMUSG00000037922	Bank1	-2.028	8.4351e-03	-0.768	4.3258e-01	-1.347	1.0557e-01	pubmed
ENSMUSG00000037992	Rara	-1.319	1.7947e-09	-1.738	9.8742e-09	-0.712	1.2937e-01	pubmed
ENSMUSG00000038010	Ccdc138	-0.109	9.0341e-01	-1.356	9.5233e-03	-1.239	9.5862e-03	pubmed
ENSMUSG00000038065	Mturn	-1.531	2.6509e-05	-2.672	2.8278e-05	-1.380	8.3329e-02	pubmed
ENSMUSG00000038068	Rnf144b	-1.662	8.3270e-08	-1.105	1.1197e-02	-1.113	8.9531e-05	pubmed
ENSMUSG00000038070	Cntln	-0.883	7.7214e-02	-1.356	8.5100e-03	-0.638	2.7154e-01	pubmed

Ensg	Symbol	CLP1 LFC	CLP1 FDR	CLP2 LFC	CLP2 FDR	CLP3 LFC	CLP3 FDR	Link
ENSMUSG00000038079	Tmem237	-0.994	3.5955e-02	-1.122	8.6909e-03	-0.413	4.9067e-01	pubmed
ENSMUSG00000038080	Kdm1b	-0.817	8.7688e-05	-1.194	4.9431e-10	-0.919	4.4649e-04	pubmed
ENSMUSG00000038122	Tbc1d32	-0.603	5.6731e-02	-1.014	1.1048e-02	-1.368	1.3137e-03	pubmed
ENSMUSG00000038126	Mphosph9	-1.076	9.8947e-03	-1.445	7.4453e-03	-0.816	1.0804e-01	pubmed
ENSMUSG00000038143	Stox2	-2.472	4.4765e-07	-0.794	2.8360e-01	-0.113	9.1674e-01	pubmed
ENSMUSG00000038145	Snrk	-1.233	3.0463e-14	-0.860	7.9642e-04	-0.575	3.9239e-02	pubmed
ENSMUSG00000038173	Enpp6	-1.260	3.5448e-03	-0.526	4.5399e-01	-0.893	1.5287e-01	pubmed
ENSMUSG00000038175	Mylip	-1.137	1.8227e-05	0.076	8.9405e-01	-0.166	7.7122e-01	pubmed
ENSMUSG00000038193	Hand2	-1.365	1.3276e-04	0.009	9.8877e-01	-0.079	9.0562e-01	pubmed
ENSMUSG00000038195	Rilp	-1.885	2.4074e-13	-2.760	5.0391e-09	-2.034	1.6740e-03	pubmed
ENSMUSG00000038241	Cep250	-1.362	4.6103e-12	-1.057	1.5511e-03	-0.365	3.7233e-01	pubmed
ENSMUSG00000038267	Slc22a23	-1.186	7.6435e-07	-1.121	3.5895e-14	-1.146	3.9136e-04	pubmed
ENSMUSG00000038295	Atg9b	-1.720	7.3203e-04	-1.328	1.6811e-02	-0.955	1.2754e-01	pubmed
ENSMUSG00000038298	Pdzk1	-0.926	2.8770e-03	-1.563	1.2926e-05	-1.299	2.1113e-05	pubmed
ENSMUSG00000038323	1700066M21Rik	-1.017	2.3011e-04	-1.292	1.6932e-09	-0.986	4.4076e-03	pubmed
ENSMUSG00000038366	Lasp1	-0.447	5.9446e-02	-1.399	5.0461e-05	-1.057	1.3274e-02	pubmed
ENSMUSG00000038370	Pcp4l1	-2.825	1.3128e-08	-2.454	4.5406e-04	-1.761	5.8126e-03	pubmed
ENSMUSG00000038375	Trp53inp2	-0.976	5.5885e-05	-1.023	2.9264e-08	-1.224	2.3496e-04	pubmed
ENSMUSG00000038403	Hjv	-2.357	2.6365e-03	-2.329	6.5677e-20	-1.468	1.1846e-01	pubmed
ENSMUSG00000038415	Foxq1	-2.989	7.5889e-15	-2.198	3.8792e-10	-2.268	1.9050e-07	pubmed
ENSMUSG00000038422	Hdhd3	-2.136	3.2781e-08	-2.129	1.0518e-09	-1.521	1.3657e-02	pubmed
ENSMUSG00000038451	Spsb2	-1.067	5.6125e-05	-0.475	1.0604e-01	0.227	6.4243e-01	pubmed
ENSMUSG00000038599	Capn8	-2.020	2.8207e-06	-3.010	1.9165e-04	-1.465	4.0061e-02	pubmed
ENSMUSG00000038618	Rassf7	-2.606	1.0503e-25	-2.190	7.4579e-15	-1.370	1.1357e-03	pubmed
ENSMUSG00000038623	Tm6sf1	-1.411	3.0186e-04	-0.545	5.1128e-01	-0.614	3.1212e-01	pubmed
ENSMUSG00000038637	Lrrc56	-1.726	3.2786e-06	-1.860	1.3621e-03	-1.965	2.7661e-04	pubmed
ENSMUSG00000038644	Pold1	-1.330	8.6210e-05	-1.636	3.1386e-02	-0.175	7.9707e-01	pubmed
ENSMUSG00000038692	Hoxb4	-2.318	1.9988e-06	-0.537	6.7100e-01	-0.346	7.0546e-01	pubmed
ENSMUSG00000038695	Josd2	-1.198	3.4411e-11	-1.235	1.5563e-04	-0.846	3.7949e-02	pubmed
ENSMUSG00000038742	Angptl6	-1.179	3.0612e-03	-0.533	3.5532e-01	-0.502	3.8281e-01	pubmed
ENSMUSG00000038751	Ptk6	-3.795	1.0343e-05	-1.870	3.5831e-02	-1.250	3.9605e-01	pubmed
ENSMUSG00000038777	Sema6c	-6.499	9.2959e-07	-1.107	3.4666e-01	-1.164	3.0044e-01	pubmed
ENSMUSG00000038797	Zscan2	-1.270	5.9935e-03	-1.143	6.0066e-02	-0.450	6.2805e-01	pubmed
ENSMUSG00000038831	Ralgps1	-1.461	2.6894e-05	-1.961	6.8346e-04	-1.189	7.8587e-02	pubmed
ENSMUSG00000038949	Cnst	-1.154	7.5885e-07	-0.576	3.5798e-02	0.075	8.4192e-01	pubmed
ENSMUSG00000038967	Pdk2	-1.548	1.8790e-21	-2.397	2.4347e-29	-1.631	1.8690e-04	pubmed
ENSMUSG00000038982	Bloc1s5	-0.889	1.0714e-05	-1.282	3.9836e-06	-0.696	2.4405e-02	pubmed
ENSMUSG00000038990	Cables2	-0.763	4.1981e-05	-1.401	8.8061e-06	-0.894	8.9855e-03	pubmed
ENSMUSG00000039062	Anpep	-0.538	3.1406e-04	-1.088	1.6124e-15	-1.023	1.1706e-03	pubmed
ENSMUSG00000039081	Zfp503	-1.927	2.1407e-05	-1.504	4.2383e-02	-1.693	7.1455e-02	pubmed
ENSMUSG00000039084	Chad	-2.854	2.2958e-11	-2.238	1.4349e-01	-1.725	3.0956e-02	pubmed
ENSMUSG00000039089	L3mbtl3	-1.235	6.2604e-10	-1.339	9.6819e-05	-0.450	5.5625e-01	pubmed
ENSMUSG00000039096	Rsad1	-1.091	1.5386e-05	-0.270	6.3926e-01	0.495	2.2427e-01	pubmed
ENSMUSG00000039157	Fam102a	-1.308	3.6085e-07	-1.839	1.5816e-07	-1.126	3.1619e-03	pubmed
ENSMUSG00000039167	Adgrl4	-1.186	3.4259e-06	-0.786	2.6982e-04	-0.483	1.7041e-01	pubmed
ENSMUSG00000039206	Daglb	-1.011	3.7449e-05	-1.023	6.5677e-06	-0.697	2.1577e-03	pubmed
ENSMUSG00000039238	Zfp750	-1.091	8.6527e-05	-1.688	7.6938e-04	-1.275	2.1764e-01	pubmed
ENSMUSG00000039242	B3galnt2	-0.827	4.0726e-05	-1.437	8.2940e-03	-1.408	9.8883e-03	pubmed
ENSMUSG00000039253	Fn3krp	-1.301	1.3954e-09	-1.057	6.2736e-02	-0.572	1.2670e-01	pubmed
ENSMUSG00000039307	Hexdc	-1.053	6.1876e-05	-0.804	1.0871e-01	-0.052	9.2399e-01	pubmed
ENSMUSG00000039349	C130074G19Rik	-1.201	5.9837e-17	-1.252	3.2204e-09	-1.012	5.2746e-04	pubmed
ENSMUSG00000039457	Ppl	-0.614	2.9494e-02	-1.950	7.3149e-07	-0.522	4.3623e-01	pubmed
ENSMUSG00000039461	Tcta	-1.126	6.4012e-16	-0.872	7.0132e-02	-0.571	2.5492e-01	pubmed
ENSMUSG00000039485	Tspyl4	-1.119	7.3913e-03	-1.100	1.4247e-01	-0.042	9.6711e-01	pubmed
ENSMUSG00000039533	Mmd2	-1.169	1.0826e-01	-3.192	6.7713e-03	-0.433	5.6530e-01	pubmed
ENSMUSG00000039620	Trmt9b	-1.338	4.4727e-03	-0.320	6.2123e-01	-0.046	9.5830e-01	pubmed
ENSMUSG00000039632	Ccdc151	-2.982	1.7413e-06	-2.507	5.9133e-03	-1.911	3.6689e-02	pubmed
ENSMUSG00000039660	Spout1	-0.467	2.1847e-02	-1.279	1.6665e-07	-0.619	2.7597e-02	pubmed
ENSMUSG00000039670	Oxld1	-1.245	1.6098e-03	-1.265	1.0074e-02	-1.096	2.2215e-02	pubmed
ENSMUSG00000039671	Zmynd8	-0.830	1.5549e-12	-1.291	1.0414e-07	-0.699	2.9471e-02	pubmed
ENSMUSG00000039686	Zer1	-1.059	3.2514e-12	-1.265	3.3856e-07	-0.645	2.2380e-02	pubmed
ENSMUSG00000039741	Bahcc1	-1.422	4.4335e-11	-1.615	4.9732e-08	-0.447	4.4009e-01	pubmed
ENSMUSG00000039759	Thap3	-1.167	3.6379e-05	-1.277	8.6369e-04	-0.694	1.0778e-01	pubmed
ENSMUSG00000039763	Dnajc28	-1.897	1.2324e-16	-1.974	1.3941e-13	-1.400	5.7251e-04	pubmed
ENSMUSG00000039765	Cc2d2a	-1.426	4.7822e-05	-0.246	6.9735e-01	0.029	9.6266e-01	pubmed
ENSMUSG00000039781	Cep131	-1.563	2.5883e-06	-1.671	3.8531e-02	-1.041	1.3834e-01	pubmed
ENSMUSG00000039831	Arhgap29	-0.892	8.9461e-11	-1.092	1.3939e-09	-0.771	1.1416e-04	pubmed
ENSMUSG00000039853	Trim14	-0.075	8.2339e-01	-1.196	5.5929e-03	-0.547	2.5025e-01	pubmed
ENSMUSG00000039959	Hip1	-1.230	6.9500e-11	-0.470	3.5927e-01	-0.099	8.5979e-01	pubmed
ENSMUSG00000039976	Tbc1d16	-0.760	1.8918e-04	-1.097	3.0055e-05	-0.107	8.0272e-01	pubmed
ENSMUSG00000039989	Cbx4	-1.445	5.1186e-09	-1.681	3.5644e-04	-0.262	7.4110e-01	pubmed
ENSMUSG00000040035	Disp2	-1.845	6.2676e-04	-1.664	5.3978e-03	-0.171	8.6343e-01	pubmed
ENSMUSG00000040102	Klhl42	-1.147	5.5166e-09	-1.468	1.1236e-04	-0.993	2.3071e-02	pubmed
ENSMUSG00000040127	Sdr9c7	-3.519	6.0594e-22	-3.935	4.7034e-22	-3.311	2.0510e-04	pubmed
ENSMUSG00000040146	Rgl3	-1.773	5.3721e-25	-1.879	1.1991e-05	-0.986	7.8331e-02	pubmed
ENSMUSG00000040170	Fmo2	-1.229	2.5539e-06	-1.323	5.6101e-03	-1.169	9.0350e-03	pubmed
ENSMUSG00000040195	Nemp1	-0.642	4.3582e-03	-1.186	6.8652e-04	-0.490	9.8016e-02	pubmed
ENSMUSG00000040280	Ndufa4l2	-1.811	7.8162e-03	-1.118	3.0936e-01	-1.375	1	pubmed
ENSMUSG00000040282	BC052040	-1.459	1.9295e-03	-1.203	2.4279e-02	-0.763	1.7177e-01	pubmed
ENSMUSG00000040283	Btnl9	-3.518	9.2113e-09	-4.373	2.8083e-11	-2.594	2.9637e-04	pubmed
ENSMUSG00000040350	Trim7	-1.546	2.1359e-07	-1.628	6.3792e-05	-0.309	6.1615e-01	pubmed
ENSMUSG00000040434	Large2	-2.352	2.6050e-04	-1.406	2.6566e-01	-0.625	6.0571e-01	pubmed
ENSMUSG00000040441	Slc26a10	-1.850	1.3736e-03	-1.015	1.9035e-01	-1.683	8.7679e-02	pubmed
ENSMUSG00000040447	Spns2	-1.916	4.0939e-17	-1.171	3.2222e-04	-0.379	5.1089e-01	pubmed
ENSMUSG00000040536	Necab1	-0.708	1.8845e-04	-0.799	3.1080e-04	-1.056	2.3039e-03	pubmed
ENSMUSG00000040616	Tmem51	-0.658	6.8240e-02	-1.198	1.8539e-03	-1.052	3.2537e-02	pubmed
ENSMUSG00000040640	Erc2	-0.486	2.1878e-01	-1.349	1.3269e-03	-0.797	7.6926e-02	pubmed
ENSMUSG00000040658	Dnph1	-1.733	4.0389e-05	-1.327	4.3294e-04	-1.029	1.8497e-02	pubmed
ENSMUSG00000040687	Madd	-1.231	1.3289e-09	-1.621	3.9219e-13	-0.576	5.1210e-02	pubmed
ENSMUSG00000040712	Camta2	-1.049	1.7529e-16	-0.972	5.4547e-04	-0.433	3.5066e-01	pubmed
ENSMUSG00000040717	Il17rd	-1.424	8.2436e-03	-1.491	1.2773e-01	-0.021	9.8574e-01	pubmed

		CLP1		CLP2		CLP3		
Ensg	Symbol	LFC	FDR	LFC	FDR	LFC	FDR	Link
ENSMUSG00000040724	Kcna2	-3.742	1.7211e-09	-2.759	2.3252e-04	-2.728	5.0674e-05	pubmed
ENSMUSG00000040740	Slc25a34	-2.907	2.8531e-09	-3.114	8.8939e-04	-1.960	9.9397e-02	pubmed
ENSMUSG00000040795	Iqcc	-2.237	1.4025e-14	-2.395	3.8276e-05	-1.103	1.6541e-01	pubmed
ENSMUSG00000040841	Six5	-1.146	2.3401e-03	-1.335	2.0615e-02	-0.023	9.8397e-01	pubmed
ENSMUSG00000040860	Crocc	-1.151	7.5465e-03	-1.274	1.2742e-01	-0.457	6.1639e-01	pubmed
ENSMUSG00000040891	Foxa3	-0.219	1.4670e-01	-0.559	1.3803e-01	-1.034	2.5903e-04	pubmed
ENSMUSG00000040918	Slc19a2	-0.896	2.1469e-19	-1.390	2.1272e-20	-0.977	5.3270e-04	pubmed
ENSMUSG00000040957	Cables1	-1.247	4.6204e-03	-0.527	4.9772e-01	-0.446	6.0525e-01	pubmed
ENSMUSG00000041012	Cmtm8	-0.565	1.4003e-03	-1.023	5.9846e-04	-0.714	1.5702e-02	pubmed
ENSMUSG00000041075	Fzd7	-0.940	1.9936e-04	-1.529	6.6741e-06	-0.702	7.7490e-02	pubmed
ENSMUSG00000041117	Ccdc8	-1.313	3.1586e-02	-2.729	5.8129e-03	-0.993	3.1016e-01	pubmed
ENSMUSG00000041132	N4bp2l1	-2.018	9.0510e-11	-2.207	1.2125e-18	-1.648	5.7251e-04	pubmed
ENSMUSG00000041147	Brca2	-1.927	1.2918e-05	-0.775	2.6137e-01	-0.567	3.9743e-01	pubmed
ENSMUSG00000041215	Yeats2	-1.256	1.9490e-13	-1.090	2.8762e-04	-0.497	2.7652e-01	pubmed
ENSMUSG00000041272	Tox	-2.407	2.3511e-07	-1.614	1.9890e-04	-0.975	1.7053e-01	pubmed
ENSMUSG00000041298	Katnal1	-1.154	2.8319e-07	-0.987	6.8992e-02	-0.438	3.4731e-01	pubmed
ENSMUSG00000041324	Inhba	-3.895	1.8892e-12	-1.721	4.9868e-04	-0.658	3.6703e-01	pubmed
ENSMUSG00000041351	Rap1gap	-1.397	9.8546e-05	-1.683	1.9215e-03	-0.191	8.6474e-01	pubmed
ENSMUSG00000041354	Rgl2	-1.158	2.7158e-11	-0.868	1.0031e-02	-0.642	1.0119e-01	pubmed
ENSMUSG00000041361	Myzap	-1.937	4.0117e-04	-0.771	2.1825e-01	-0.752	2.5921e-01	pubmed
ENSMUSG00000041372	B4galnt3	-3.052	1.2476e-07	-3.566	4.4255e-07	-1.103	3.3891e-01	pubmed
ENSMUSG00000041429	Nthl1	-1.919	1.9952e-06	-2.352	4.6249e-04	-0.699	2.2682e-01	pubmed
ENSMUSG00000041445	Mmrn2	-0.991	1.2081e-05	-1.359	2.6308e-05	-0.873	2.9633e-03	pubmed
ENSMUSG00000041471	Shld2	-1.356	1.3332e-13	-0.874	5.3474e-02	-1.546	9.6423e-02	pubmed
ENSMUSG00000041491	Cep78	-1.377	3.8022e-04	-0.843	1.6344e-01	-0.123	8.2600e-01	pubmed
ENSMUSG00000041530	Ago1	-0.367	7.2776e-02	-1.104	2.5568e-09	-0.497	7.7866e-02	pubmed
ENSMUSG00000041540	Sox5	-1.589	3.3011e-10	-1.163	1.0418e-03	-0.810	6.0416e-02	pubmed
ENSMUSG00000041609	Bicdl1	-2.569	1.2335e-05	-1.667	1.0705e-02	-0.555	5.7760e-01	pubmed
ENSMUSG00000041684	Bivm	-1.117	4.7530e-06	-1.333	1.2239e-07	-0.754	5.6774e-02	pubmed
ENSMUSG00000041688	Amot	-1.730	1.7284e-11	-1.820	1.1215e-07	-0.444	3.6143e-01	pubmed
ENSMUSG00000041729	Coro2b	-2.279	1.1989e-06	-1.837	9.4490e-02	0.350	7.4552e-01	pubmed
ENSMUSG00000041757	Plekha6	-1.351	1.9520e-05	-1.430	1.5293e-07	-0.835	1.2178e-01	pubmed
ENSMUSG00000041782	Lad1	-2.655	4.4308e-08	-2.517	4.2165e-12	-1.164	1.6985e-01	pubmed
ENSMUSG00000041797	Abca9	-3.465	2.8834e-10	-2.543	2.9864e-08	-1.630	3.1830e-03	pubmed
ENSMUSG00000041939	Mvk	-0.655	4.8971e-02	-1.121	3.0867e-04	-0.923	1.9650e-02	pubmed
ENSMUSG00000041966	Dcaf17	-1.085	3.8991e-10	-1.329	1.7639e-07	-0.745	4.6858e-02	pubmed
ENSMUSG00000041975	Mettl8	-1.190	2.2276e-06	-1.507	2.4452e-06	-1.461	1.8407e-03	pubmed
ENSMUSG00000042010	Acacb	-1.150	1.5491e-04	-0.734	2.4733e-02	-0.739	5.4408e-02	pubmed
ENSMUSG00000042066	Tmcc2	-1.732	1.1273e-06	-1.223	1.3621e-03	-0.516	5.0335e-01	pubmed
ENSMUSG00000042099	Kank3	-1.081	8.7688e-05	-0.611	3.7698e-01	-0.814	1.2394e-01	pubmed
ENSMUSG00000042115	Klhdc8a	-1.048	1.8183e-02	-2.145	1.5063e-03	-1.493	1.1740e-02	pubmed
ENSMUSG00000042118	Bhmt2	-1.266	4.3156e-05	-1.130	3.2834e-06	-1.280	5.9006e-08	pubmed
ENSMUSG00000042202	Slc35e2	-1.074	1.8539e-09	-1.422	1.8949e-19	-0.772	1.3320e-05	pubmed
ENSMUSG00000042320	Prox2	-1.595	1.1177e-03	-1.553	1.0922e-01	-0.885	1.6847e-01	pubmed
ENSMUSG00000042328	Hps4	-0.586	2.4505e-02	-1.160	2.4848e-04	-0.933	3.8779e-02	pubmed
ENSMUSG00000042404	Dennd4b	-0.408	7.2994e-02	-0.535	2.5885e-01	-1.152	9.8759e-04	pubmed
ENSMUSG00000042564	Fam227a	-2.849	1.2433e-03	-2.097	2.1241e-01	-0.594	5.8368e-01	pubmed
ENSMUSG00000042579	4632404H12Rik	-1.322	3.4327e-06	-1.691	7.5844e-03	-0.723	4.1915e-01	pubmed
ENSMUSG00000042589	Cux2	-1.951	5.8204e-03	-1.930	1	-1.984	2.3812e-02	pubmed
ENSMUSG00000042605	Atxn2	-1.143	7.2514e-30	-1.194	8.8254e-07	-0.320	4.9812e-01	pubmed
ENSMUSG00000042628	Zfyve1	-0.625	5.7575e-07	-1.099	7.8036e-04	-0.524	7.4085e-03	pubmed
ENSMUSG00000042647	Acad12	-0.749	3.1302e-03	-1.379	2.4817e-06	-1.162	8.1326e-04	pubmed
ENSMUSG00000042675	Ypel3	-0.832	9.1504e-05	-1.127	2.5567e-05	-0.950	3.9859e-04	pubmed
ENSMUSG00000042743	Sgtb	-1.366	1.3318e-03	-1.137	3.2508e-02	-0.474	3.7089e-01	pubmed
ENSMUSG00000042797	Aqp11	-2.047	1.5922e-12	-2.763	1.5430e-14	-2.061	7.4790e-13	pubmed
ENSMUSG00000042807	Hecw2	-0.338	5.9455e-01	-0.323	4.2735e-01	-1.125	2.9647e-03	pubmed
ENSMUSG00000042834	Nrep	-1.555	8.2400e-03	-1.453	1.8362e-01	-1.424	3.7247e-02	pubmed
ENSMUSG00000043013	Onecut1	-1.703	4.5753e-04	-1.017	7.1515e-02	-1.247	3.4794e-02	pubmed
ENSMUSG00000043065	Spice1	-2.279	2.6634e-09	-1.878	8.5458e-03	-1.608	1.1365e-02	pubmed
ENSMUSG00000043090	Zfp866	-0.951	1.2776e-04	-1.236	3.7843e-03	-0.914	5.0127e-02	pubmed
ENSMUSG00000043099	Hic1	-1.635	2.9346e-03	-2.376	1.7788e-04	-0.277	8.3083e-01	pubmed
ENSMUSG00000043154	Ppp2r3a	-1.103	2.3684e-07	-0.879	1.4531e-04	-0.191	5.4410e-01	pubmed
ENSMUSG00000043251	Exoc3l	-1.354	1.0703e-05	-0.498	5.1568e-01	0.266	7.3639e-01	pubmed
ENSMUSG00000043257	Pigv	-1.053	1.8249e-04	-1.230	3.2256e-02	-0.903	4.8696e-02	pubmed
ENSMUSG00000043487	Acot6	-1.249	8.2210e-03	-0.176	8.3663e-01	0.044	9.5724e-01	pubmed
ENSMUSG00000043510	Hscb	-0.742	9.8944e-02	-1.634	3.5264e-03	-1.159	1.0702e-01	pubmed
ENSMUSG00000043587	Pxylp1	-1.711	4.1107e-05	-1.339	1.8155e-02	0.105	8.6011e-01	pubmed
ENSMUSG00000043602	Zfp3	-1.061	9.6169e-03	-0.300	7.2794e-01	-0.455	5.7499e-01	pubmed
ENSMUSG00000043631	Ecm2	-1.902	3.4219e-03	-0.718	5.6422e-01	-1.119	2.8867e-01	pubmed
ENSMUSG00000043648	Pld6	-3.150	2.5129e-02	-4.302	9.0447e-03	-2.791	1	pubmed
ENSMUSG00000043789	Vwce	-1.752	8.6648e-05	-1.450	9.1430e-09	-0.532	4.0269e-01	pubmed
ENSMUSG00000043993	2900052L18Rik	-3.106	8.9648e-08	-1.673	1.7462e-01	-0.893	3.6691e-01	pubmed
ENSMUSG00000044026	Slc35g1	-1.157	5.7805e-04	-0.565	1.8980e-01	-0.373	4.4814e-01	pubmed
ENSMUSG00000044033	Ccdc141	-2.437	2.8641e-12	-1.662	1.6177e-06	-0.584	4.1718e-01	pubmed
ENSMUSG00000044066	Cep68	-1.099	6.2224e-06	-1.751	3.6186e-09	-1.133	3.5243e-02	pubmed
ENSMUSG00000044134	Pheta1	-0.985	2.2267e-05	-1.201	1.3572e-03	-0.422	1.8537e-01	pubmed
ENSMUSG00000044206	Vsig4	-1.258	3.9058e-07	-0.541	2.2566e-01	-0.995	1.4234e-03	pubmed
ENSMUSG00000044229	Nxpe4	-2.018	2.6238e-03	-1.822	2.8907e-02	-1.677	9.7192e-02	pubmed
ENSMUSG00000044231	Nhlrc1	-2.270	2.0134e-04	-2.152	3.0922e-02	-0.183	8.9133e-01	pubmed
ENSMUSG00000044252	Osbpl1a	-0.578	9.8720e-04	-0.735	1.4438e-03	-1.124	6.0781e-05	pubmed
ENSMUSG00000044279	Crb3	-0.964	3.7735e-05	-1.554	2.3672e-05	-0.769	1.2781e-01	pubmed
ENSMUSG00000044328	Trp53i13	-1.550	2.4316e-04	-0.924	2.1891e-01	-0.466	4.3445e-01	pubmed
ENSMUSG00000044345	Marveld1	-2.105	1.0709e-31	-2.091	3.1211e-12	-0.990	9.4255e-02	pubmed
ENSMUSG00000044359	P2ry4	-2.971	1.5408e-06	-4.614	2.0262e-06	-2.154	1.5109e-03	pubmed
ENSMUSG00000044468	Tent5c	-0.377	3.4306e-01	-1.786	2.1221e-12	-0.695	2.9689e-02	pubmed
ENSMUSG00000044469	Tnfaip8l1	-1.628	4.7526e-15	-2.269	2.1050e-23	-2.246	6.1593e-07	pubmed
ENSMUSG00000044548	Dact1	-0.759	4.2015e-02	-1.444	2.7880e-03	0.013	9.9353e-01	pubmed
ENSMUSG00000044641	Pard6b	-1.218	1.5908e-06	-0.973	1.9641e-02	-0.098	8.6696e-01	pubmed
ENSMUSG00000044700	Tmem201	-1.578	7.9650e-14	-1.825	6.0712e-09	-0.755	1.3364e-01	pubmed
ENSMUSG00000044788	Fads6	-2.060	2.9663e-28	-2.568	4.9726e-30	-1.601	6.1844e-03	pubmed
ENSMUSG00000044820	AY074887	-4.251	9.1959e-03	-0.083	1	0.034	1	pubmed

		CLP1		CLP2		CLP3		
Ensg	Symbol	LFC	FDR	LFC	FDR	LFC	FDR	Link
ENSMUSG00000044876	Zfp444	-1.756	3.5068e-16	-2.370	1.1117e-11	-1.351	1.7303e-03	pubmed
ENSMUSG00000044881	Coa4	-0.892	1.1511e-02	-1.410	5.0112e-02	-1.507	2.5225e-03	pubmed
ENSMUSG00000044916	1700029I15Rik	-1.463	4.1744e-03	-0.122	9.3119e-01	0.147	8.4365e-01	pubmed
ENSMUSG00000044937	Ttc41	-1.159	7.3381e-04	-0.670	5.2677e-01	-0.423	5.4447e-01	pubmed
ENSMUSG00000044952	Kctd21	-1.065	1.8313e-08	-1.534	2.3358e-02	-1.183	2.7712e-02	pubmed
ENSMUSG00000045064	Zc2hc1c	-1.812	1.2486e-06	-0.950	3.8088e-02	-0.748	1.3743e-01	pubmed
ENSMUSG00000045087	S1pr5	-3.508	4.8838e-08	-3.038	3.2448e-07	-2.165	1.2680e-03	pubmed
ENSMUSG00000045094	Arhgef37	-1.432	9.5574e-05	-2.616	2.3597e-05	-2.042	4.3342e-03	pubmed
ENSMUSG00000045103	Dmd	-1.029	1.8664e-04	-0.807	2.4844e-02	-0.213	5.4734e-01	pubmed
ENSMUSG00000045211	Nudt18	-0.784	2.0024e-03	-1.149	1.3530e-06	-0.692	1.9981e-01	pubmed
ENSMUSG00000045287	Rtn4rl1	-3.248	6.8288e-13	-4.087	1.5688e-12	-1.847	1.9098e-02	pubmed
ENSMUSG00000045312	Lhfpl2	-3.274	1.3280e-23	-2.550	4.2165e-12	-1.791	3.9735e-02	pubmed
ENSMUSG00000045316	Fahd1	-1.909	6.8752e-09	-2.448	1.8463e-23	-1.650	3.9347e-05	pubmed
ENSMUSG00000045319	Proser2	-2.224	3.6243e-21	-2.244	4.7470e-16	-1.730	6.3545e-03	pubmed
ENSMUSG00000045374	Wdr81	-0.451	9.3785e-02	-1.092	1.1159e-05	-0.107	6.6486e-01	pubmed
ENSMUSG00000045392	Olfr1033	-1.232	7.6661e-05	-2.257	2.6244e-08	-1.876	2.2356e-06	pubmed
ENSMUSG00000045414	Dipk2a	-1.844	2.7938e-26	-1.598	5.0166e-18	-1.294	1.6910e-04	pubmed
ENSMUSG00000045441	Gprin3	-2.506	5.2088e-05	-2.086	2.6637e-05	-1.124	6.9779e-03	pubmed
ENSMUSG00000045691	Thtpa	-1.029	5.5635e-06	-0.645	2.6888e-02	-0.546	2.8397e-01	pubmed
ENSMUSG00000045775	Slc16a5	-4.626	4.3128e-44	-4.759	8.3343e-14	-3.125	1.0325e-02	pubmed
ENSMUSG00000045822	Zswim3	-1.316	4.7228e-03	-0.984	2.1629e-01	-0.778	3.2205e-01	pubmed
ENSMUSG00000045875	Adra1a	-2.735	3.6225e-14	-0.892	2.0157e-01	-0.657	3.7284e-01	pubmed
ENSMUSG00000045930	Clec14a	-2.731	1.7959e-14	-1.948	5.2804e-05	-0.961	1.7690e-01	pubmed
ENSMUSG00000045954	Cavin2	-2.678	7.8876e-14	-1.856	1.2521e-10	-1.092	1.1805e-01	pubmed
ENSMUSG00000045991	Onecut2	-1.276	7.0657e-06	-1.051	1.3572e-03	-1.297	7.6762e-04	pubmed
ENSMUSG00000046027	Stard5	-2.365	1.0513e-26	-1.823	2.8871e-07	-1.321	5.0738e-03	pubmed
ENSMUSG00000046070	Igfals	-0.906	7.4437e-02	-1.380	3.4801e-02	-1.124	5.9588e-03	pubmed
ENSMUSG00000046080	Clec9a	-1.233	6.0577e-04	-1.843	3.7843e-03	-0.964	5.0039e-02	pubmed
ENSMUSG00000046312	Myorg	-1.885	6.3906e-11	-2.204	2.8424e-30	-0.905	3.1393e-05	pubmed
ENSMUSG00000046314	Stxbp6	-1.153	8.8163e-03	-1.361	2.2959e-04	-1.204	3.1270e-05	pubmed
ENSMUSG00000046318	Ccbe1	-1.210	2.4326e-03	-0.297	6.9671e-01	-0.235	6.9026e-01	pubmed
ENSMUSG00000046324	Ermp1	-0.837	1.2050e-06	-1.273	5.4400e-11	-0.824	8.8840e-03	pubmed
ENSMUSG00000046329	Slc25a23	-0.519	9.5138e-03	-1.001	3.5603e-03	-0.903	3.1387e-02	pubmed
ENSMUSG00000046380	Jrk	-1.391	9.1246e-03	-0.665	5.8389e-01	-0.502	6.3137e-01	pubmed
ENSMUSG00000046417	Lrrc75a	-1.454	3.6404e-04	-1.588	1.3622e-03	-2.133	1.1239e-04	pubmed
ENSMUSG00000046447	Camk2n1	-1.307	6.4286e-20	-1.971	7.7001e-23	-1.584	2.2536e-05	pubmed
ENSMUSG00000046470	Sox18	-2.276	2.7053e-09	-2.874	2.9940e-05	-1.606	1.1960e-02	pubmed
ENSMUSG00000046532	Ar	-2.069	9.5123e-04	-2.071	2.7640e-03	-1.136	2.1013e-01	pubmed
ENSMUSG00000046574	Prr12	-0.942	4.6838e-06	-1.588	4.8764e-05	0.061	9.1990e-01	pubmed
ENSMUSG00000046667	Rbm12b1	-1.730	1.0154e-08	-1.133	1.7889e-01	-1.436	1.4033e-02	pubmed
ENSMUSG00000046668	Cxxc5	-1.345	4.8055e-12	-2.013	3.7733e-12	-1.119	4.0062e-03	pubmed
ENSMUSG00000046811	Gltpd2	-0.922	1.0202e-11	-1.227	4.4859e-11	-1.063	2.4333e-03	pubmed
ENSMUSG00000046861	Hectd3	-1.208	2.3818e-15	-1.347	2.7820e-06	-0.473	1.4773e-01	pubmed
ENSMUSG00000046959	Slc26a1	-1.703	3.4150e-09	-2.070	1.3037e-13	-1.425	5.4658e-07	pubmed
ENSMUSG00000046997	Spsb4	-4.722	2.5623e-03	-2.114	1.0128e-01	-1.444	1	pubmed
ENSMUSG00000047003	Zfp41	-1.268	7.1794e-03	-2.114	7.0386e-04	-1.045	6.6510e-02	pubmed
ENSMUSG00000047037	Nipa1	-1.968	3.4754e-03	-1.344	4.1975e-02	-1.523	5.8516e-02	pubmed
ENSMUSG00000047090	Tmem198b	-1.503	1.4820e-05	-0.753	3.1749e-01	-0.162	8.4699e-01	pubmed
ENSMUSG00000047246	Hist1h2be	-3.380	1.5082e-04	-4.185	3.8260e-03	-5.405	1	pubmed
ENSMUSG00000047250	Ptgs1	-2.010	1.0690e-12	-1.266	2.7816e-04	-0.984	5.7403e-02	pubmed
ENSMUSG00000047379	B4gat1	-1.103	2.2000e-07	-1.142	3.7720e-05	-0.816	2.6099e-02	pubmed
ENSMUSG00000047420	Fam180a	-2.472	1.8051e-03	-1.281	2.9007e-01	-0.975	3.9416e-01	pubmed
ENSMUSG00000047492	Inhbe	-2.444	2.1331e-08	-1.109	3.6199e-01	-0.901	1	pubmed
ENSMUSG00000047496	Rnf152	-2.147	6.0456e-12	-1.841	1.8243e-10	-0.788	1.6023e-01	pubmed
ENSMUSG00000047603	Zfp235	-1.040	2.7605e-06	-0.130	7.9404e-01	-0.397	2.8871e-01	pubmed
ENSMUSG00000047635	2810006K23Rik	-1.345	3.0252e-06	-2.095	4.3100e-09	-1.738	6.2300e-04	pubmed
ENSMUSG00000047638	Nr1h4	-1.376	1.1453e-03	-0.949	2.6449e-03	-1.258	1.3108e-04	pubmed
ENSMUSG00000047656	Trpt1	-1.483	7.7716e-04	-0.866	2.1819e-01	-1.211	3.7218e-02	pubmed
ENSMUSG00000047728	Ly6g2	-1.085	2.9028e-09	-1.280	8.7602e-09	-1.335	4.8434e-06	pubmed
ENSMUSG00000047793	Sned1	-1.273	3.9058e-07	-0.800	8.0705e-02	-0.474	2.8952e-01	pubmed
ENSMUSG00000047822	Angptl8	-2.067	3.7017e-07	-1.890	3.2604e-04	-1.621	4.5436e-03	pubmed
ENSMUSG00000047866	Lonp2	-1.025	1.0811e-04	-0.381	6.0353e-02	-0.680	6.4681e-03	pubmed
ENSMUSG00000047875	Gpr157	-1.484	1.4245e-05	-1.513	5.6367e-04	0.100	8.9556e-01	pubmed
ENSMUSG00000047963	Stbd1	-2.124	3.7227e-19	-1.943	1.2760e-06	-0.729	5.1021e-01	pubmed
ENSMUSG00000048047	Zbtb33	-0.955	2.4224e-08	-1.166	1.2397e-03	-0.984	4.5324e-02	pubmed
ENSMUSG00000048200	Cracr2b	-1.168	1.7486e-03	-1.090	2.6120e-01	0.265	7.8167e-01	pubmed
ENSMUSG00000048406	B330016D10Rik	-2.121	4.0284e-03	-0.575	5.9510e-01	-0.750	5.5547e-01	pubmed
ENSMUSG00000048481	Mypop	-0.991	2.0217e-01	-2.956	1.7353e-03	-1.278	2.0697e-01	pubmed
ENSMUSG00000048482	Bdnf	-5.247	9.4950e-05	-1.919	1.3769e-02	-1.290	2.0011e-01	pubmed
ENSMUSG00000048497	Mmgt2	-1.606	2.6159e-04	-1.229	9.5970e-02	-1.721	2.4191e-03	pubmed
ENSMUSG00000048503	Tmem136	-1.630	5.7125e-03	-1.044	3.9612e-01	-1.226	2.4107e-01	pubmed
ENSMUSG00000048550	Thnsl1	-1.645	1.7158e-07	-2.168	1.1105e-04	-1.023	1.2672e-01	pubmed
ENSMUSG00000048728	Zfp454	-5.474	1.5650e-04	-4.610	4.4269e-03	-1.366	1	pubmed
ENSMUSG00000048826	Dact2	-3.496	1.0909e-21	-3.188	1.0100e-19	-1.434	5.3896e-02	pubmed
ENSMUSG00000048897	Zfp710	-0.190	4.2293e-01	-1.194	8.2503e-04	-0.229	6.9157e-01	pubmed
ENSMUSG00000048938	Nr1h5	-4.314	4.3469e-05	-2.765	4.6645e-02	-2.388	2.5217e-02	pubmed
ENSMUSG00000048965	Mrgpre	-2.313	3.4551e-03	-2.981	1.8201e-03	-0.945	3.8845e-01	pubmed
ENSMUSG00000049038	Mterf2	-1.224	3.9656e-03	-1.168	1.4090e-02	0.208	7.7470e-01	pubmed
ENSMUSG00000049044	Rapgef4	-0.194	5.6977e-01	-0.265	6.2794e-01	-1.590	3.8698e-09	pubmed
ENSMUSG00000049115	Agtr1a	-1.318	6.1811e-19	-0.983	3.1082e-04	-0.738	4.6769e-03	pubmed
ENSMUSG00000049184	Purg	-1.893	9.1029e-04	-1.151	1.3068e-01	-1.459	5.2485e-02	pubmed
ENSMUSG00000049295	Zfp219	-1.180	2.4399e-05	-1.114	1.7265e-03	-0.039	9.4210e-01	pubmed
ENSMUSG00000049321	Zfp2	-1.246	1.0025e-04	-0.850	7.8019e-02	-0.513	2.6264e-01	pubmed
ENSMUSG00000049411	Tmem241	-1.037	2.2160e-03	-0.323	5.9045e-01	-0.204	5.2873e-01	pubmed
ENSMUSG00000049493	Pls1	-1.452	7.8206e-03	-0.232	8.4313e-01	-0.268	8.2984e-01	pubmed
ENSMUSG00000049532	Sall2	-1.670	5.7925e-05	-1.596	1.4226e-04	-0.682	2.8824e-01	pubmed
ENSMUSG00000049577	Zfpm1	-1.368	7.3356e-20	-1.733	1.5563e-06	-0.586	3.2602e-01	pubmed
ENSMUSG00000049672	Zbtb14	-0.983	8.7630e-05	-1.257	4.2566e-04	-1.235	3.2594e-02	pubmed
ENSMUSG00000049686	Orai1	-0.528	5.2481e-02	-1.076	3.1360e-03	-0.519	1.4526e-01	pubmed
ENSMUSG00000049721	Gal3st1	-2.006	3.2819e-04	-0.754	5.2991e-01	-1.382	2.5557e-01	pubmed
ENSMUSG00000049811	Fam161a	-1.663	1.4389e-03	-1.026	2.1066e-01	0.405	6.4368e-01	pubmed

		CLP1		CLP2		CLP3		
Ensg	Symbol	LFC	FDR	LFC	FDR	LFC	FDR	Link
ENSMUSG00000050052	Tdrp	-1.110	6.8856e-08	-0.864	1.6972e-04	-0.515	1.3028e-01	pubmed
ENSMUSG00000050069	Grem2	-1.064	5.2268e-03	-1.309	4.7588e-04	-1.054	3.3207e-01	pubmed
ENSMUSG00000050122	Vwa3b	-1.606	2.9923e-03	-1.653	1.8326e-02	-1.563	3.5954e-02	pubmed
ENSMUSG00000050188	Lsm10	-0.978	9.3479e-03	-1.913	1.4338e-05	-0.815	1.5907e-01	pubmed
ENSMUSG00000050270	Tmem220	-1.470	8.3021e-11	-1.333	6.0106e-10	-1.316	1.5921e-05	pubmed
ENSMUSG00000050373	Snx21	-0.556	7.2362e-02	-1.560	1.0921e-04	-0.904	6.9678e-03	pubmed
ENSMUSG00000050390	C77080	-1.072	1.8410e-07	-1.357	1.7542e-17	-0.316	3.6900e-01	pubmed
ENSMUSG00000050445	Cyp8b1	-1.794	2.5985e-08	-1.366	7.0972e-02	-1.885	2.8811e-02	pubmed
ENSMUSG00000050493	Fam167b	-2.259	3.2067e-04	-1.020	1.6213e-01	-1.912	3.0864e-03	pubmed
ENSMUSG00000050556	Kcnb1	-2.866	3.1815e-13	-0.735	4.1539e-01	-0.377	7.1665e-01	pubmed
ENSMUSG00000050592	Fam78a	-2.088	2.3594e-03	-0.447	6.6006e-01	-0.916	3.1045e-01	pubmed
ENSMUSG00000050627	Gpd1l	-2.062	3.5389e-21	-2.040	3.7484e-24	-1.166	1.8698e-03	pubmed
ENSMUSG00000050714	Zbtb26	-1.697	1.2355e-07	-1.664	3.2840e-03	-1.297	1.4774e-01	pubmed
ENSMUSG00000050730	Arhgap42	-1.132	9.7824e-10	-0.678	5.9123e-04	-0.356	1.2146e-01	pubmed
ENSMUSG00000050777	Tmem37	-1.022	1.3503e-04	-1.299	1.1876e-04	-0.919	1.8121e-03	pubmed
ENSMUSG00000050947	Amigo1	-0.895	5.8074e-04	-1.390	3.0055e-05	-0.978	2.2376e-02	pubmed
ENSMUSG00000051007	Gatd1	-1.855	3.6055e-17	-1.871	2.0428e-04	-1.251	3.1240e-02	pubmed
ENSMUSG00000051043	Gprc5c	-1.288	3.1920e-06	-1.469	1.6517e-11	-0.413	1.5082e-01	pubmed
ENSMUSG00000051098	Mblac2	-2.112	3.3341e-06	-3.170	5.6183e-11	-1.330	1.5191e-02	pubmed
ENSMUSG00000051184	Zfp524	-1.746	9.2128e-04	-1.204	8.8036e-02	-1.384	3.2740e-02	pubmed
ENSMUSG00000051427	Ccdc157	-1.406	1.1051e-14	-1.853	3.9513e-14	-1.104	2.6468e-02	pubmed
ENSMUSG00000051452	Gm11437	-2.862	1.7430e-06	-2.804	3.6780e-08	-1.643	4.2040e-02	pubmed
ENSMUSG00000051497	Kcnj16	-5.570	3.2219e-06	-2.534	3.4514e-03	-2.723	5.1990e-04	pubmed
ENSMUSG00000051615	Rap2a	-0.663	1.7611e-03	-1.321	9.6867e-07	-0.875	2.6308e-02	pubmed
ENSMUSG00000051627	Hist1h1e	-3.690	4.2967e-03	-4.455	1.1827e-02	-3.445	1	pubmed
ENSMUSG00000051652	Lrrc3	-2.110	1.2621e-13	-2.977	6.2689e-27	-1.815	2.0424e-04	pubmed
ENSMUSG00000051671	Coa6	-1.309	3.6127e-13	-0.871	6.4704e-04	-1.022	4.9002e-03	pubmed
ENSMUSG00000051705	Senp8	-2.065	5.3786e-05	-2.807	2.6361e-03	-1.568	6.1972e-02	pubmed
ENSMUSG00000051817	Sox12	-1.441	2.1767e-02	-2.165	2.4851e-04	-1.451	2.2830e-02	pubmed
ENSMUSG00000051977	Prdm9	-0.961	1.6675e-04	-1.344	8.5365e-04	-0.568	2.2991e-01	pubmed
ENSMUSG00000051984	Sec31b	-1.438	4.1987e-07	-1.197	1.7226e-02	-0.180	8.1446e-01	pubmed
ENSMUSG00000052102	Gnpda1	-1.665	2.8972e-11	-1.866	5.5156e-05	-0.778	6.2993e-02	pubmed
ENSMUSG00000052117	D630039A03Rik	-1.652	1.4096e-12	-1.276	4.1346e-04	-1.350	1.4359e-03	pubmed
ENSMUSG00000052137	Rbm12b2	-1.846	3.3658e-07	-0.155	8.6407e-01	-0.795	2.2231e-01	pubmed
ENSMUSG00000052160	Pld4	-1.054	4.6754e-04	-0.983	5.9636e-02	-0.871	6.0854e-02	pubmed
ENSMUSG00000052229	Gpr17	-2.544	1.1153e-06	-1.035	2.5645e-01	-1.253	1.2525e-01	pubmed
ENSMUSG00000052302	Tbc1d30	-3.346	1.7285e-17	-2.955	1.9142e-17	-1.610	1.4706e-03	pubmed
ENSMUSG00000052392	Acot4	-2.039	8.7054e-07	-1.057	2.9634e-02	-0.136	6.4242e-01	pubmed
ENSMUSG00000052435	Cebpe	-1.284	3.9590e-02	-2.482	1.4368e-04	-0.608	5.8246e-01	pubmed
ENSMUSG00000052563	D930048N14Rik	-3.035	1.4596e-05	-3.695	1.1876e-03	-2.139	9.0935e-03	pubmed
ENSMUSG00000052565	Hist1h1d	-4.638	1.8565e-05	-5.173	1.1756e-03	-5.468	1	pubmed
ENSMUSG00000052566	Hook2	-1.097	2.6845e-07	-1.567	2.2624e-13	-0.956	2.2301e-02	pubmed
ENSMUSG00000052595	A1cf	-1.078	9.8291e-06	-0.728	9.3386e-02	-1.318	5.2703e-05	pubmed
ENSMUSG00000052609	Plekhg3	-1.090	1.1796e-09	-1.461	8.3539e-11	-1.214	2.2318e-03	pubmed
ENSMUSG00000052676	Zmat1	-2.067	4.6743e-10	-1.517	1.0464e-03	-1.845	1.6439e-03	pubmed
ENSMUSG00000052698	Tln2	-1.490	7.9543e-08	-1.846	1.5975e-08	-1.183	3.1540e-04	pubmed
ENSMUSG00000052751	Repin1	-1.397	6.3015e-14	-1.389	2.9291e-06	-0.903	6.2112e-04	pubmed
ENSMUSG00000052794	1700030K09Rik	-1.015	5.2381e-04	-0.982	6.6001e-02	-0.518	2.7665e-01	pubmed
ENSMUSG00000052889	Prkcb	-1.240	6.9138e-03	-0.427	5.0294e-01	-0.754	2.4476e-01	pubmed
ENSMUSG00000052957	Gas1	-6.156	7.6006e-08	-3.552	1.8120e-09	-1.860	1.7122e-02	pubmed
ENSMUSG00000053080	2700081O15Rik	-1.112	1.7503e-04	-0.471	3.6660e-01	-0.585	3.2281e-01	pubmed
ENSMUSG00000053286	Trmt1l	-1.093	3.2801e-14	-1.047	3.8088e-02	-1.203	1.1092e-02	pubmed
ENSMUSG00000053411	Cbx7	-0.937	1.8874e-03	-1.315	1.2378e-04	-0.446	2.0841e-01	pubmed
ENSMUSG00000053414	Hunk	-2.443	1.3233e-08	-3.246	8.4521e-06	-0.326	6.5411e-01	pubmed
ENSMUSG00000053613	Notumos	-3.932	4.3944e-04	-3.616	2.4947e-02	-3.981	1	pubmed
ENSMUSG00000053846	Lipg	-1.349	1.7952e-03	-1.375	1.3836e-03	-0.889	6.4203e-02	pubmed
ENSMUSG00000053964	Lgals4	-1.768	2.3461e-08	-0.067	9.3220e-01	-0.112	9.1245e-01	pubmed
ENSMUSG00000053985	Zfp14	-1.391	3.1177e-03	-0.906	3.4695e-01	-0.028	9.8086e-01	pubmed
ENSMUSG00000054115	Skp2	-1.980	5.7456e-06	-1.693	9.7987e-03	-1.329	2.1218e-02	pubmed
ENSMUSG00000054150	Syne3	-1.608	7.8778e-09	-1.834	1.2283e-06	-0.940	8.1558e-02	pubmed
ENSMUSG00000054252	Fgfr3	-1.301	5.3877e-13	-0.723	1.8877e-02	-0.491	1.3272e-01	pubmed
ENSMUSG00000054426	A930005H10Rik	-1.593	4.0975e-05	-2.202	3.4681e-06	-1.152	1.1270e-01	pubmed
ENSMUSG00000054434	Tmem120b	-1.590	3.5507e-05	-1.659	1.7167e-04	-0.352	6.3595e-01	pubmed
ENSMUSG00000054435	Gimap4	-0.551	4.6926e-02	-1.166	4.7673e-04	-0.958	5.6910e-02	pubmed
ENSMUSG00000054477	Kcnn2	-1.365	1.1596e-08	-1.704	2.6328e-15	-1.444	2.6137e-04	pubmed
ENSMUSG00000054517	Trim65	-1.574	1.8103e-08	-1.273	9.7987e-03	-0.866	1.0774e-01	pubmed
ENSMUSG00000054545	Ugt1a6a	-2.753	5.3685e-12	-2.112	2.8823e-05	-1.850	1.5082e-02	pubmed
ENSMUSG00000054640	Slc8a1	-1.189	2.3474e-03	-0.625	3.2224e-01	-0.338	5.1968e-01	pubmed
ENSMUSG00000054676	1600014C10Rik	-1.186	1.4418e-20	-1.420	2.5226e-33	-1.411	4.1274e-05	pubmed
ENSMUSG00000054708	Ankrd24	-1.117	1.9860e-04	-1.593	1.1450e-02	-0.229	7.1490e-01	pubmed
ENSMUSG00000054723	Vmac	-1.205	2.4727e-06	-1.211	1.5211e-03	-0.195	6.4619e-01	pubmed
ENSMUSG00000054889	Dsp	-1.144	2.9776e-07	-1.352	2.3902e-12	-0.453	2.9245e-01	pubmed
ENSMUSG00000054893	Zfp667	-1.438	9.8202e-04	-0.901	1.6926e-01	-0.230	7.5044e-01	pubmed
ENSMUSG00000054932	Afp	-2.215	1.9844e-04	-1.680	3.3291e-03	-0.119	9.1733e-01	pubmed
ENSMUSG00000054939	Zfp174	-2.449	2.3999e-08	-2.097	2.3938e-02	-1.464	5.2754e-02	pubmed
ENSMUSG00000054967	Zfp647	-2.271	8.4402e-03	-0.524	7.2810e-01	-0.596	6.8849e-01	pubmed
ENSMUSG00000055027	Smyd1	-1.118	1.6883e-02	-1.082	3.2992e-03	-0.846	3.0432e-02	pubmed
ENSMUSG00000055240	Zfp101	-1.103	4.2764e-06	-0.420	5.3068e-01	-0.562	3.6326e-01	pubmed
ENSMUSG00000055296	Tmem245	-1.069	5.3325e-09	-0.616	5.3859e-05	-0.397	1.5618e-01	pubmed
ENSMUSG00000055301	Adh7	-0.920	8.0434e-02	-1.322	2.1288e-06	-1.248	4.5415e-02	pubmed
ENSMUSG00000055435	Maf	-1.142	1.6456e-05	-0.705	4.4667e-02	-0.679	4.8996e-04	pubmed
ENSMUSG00000055541	Lair1	-1.809	3.8732e-06	-0.605	3.5247e-01	-1.090	7.7937e-02	pubmed
ENSMUSG00000055612	Cdca7	-1.576	4.8203e-04	-0.843	1.9028e-01	-0.678	2.1898e-01	pubmed
ENSMUSG00000055733	Nap1l3	-2.389	1.1246e-03	-1.522	5.6039e-02	-0.630	6.7863e-01	pubmed
ENSMUSG00000055782	Abcd2	-0.723	1.1157e-01	-1.901	6.4426e-03	-1.705	3.4024e-03	pubmed
ENSMUSG00000055799	Tcf7l1	-1.720	2.4893e-08	-1.213	2.5279e-04	-0.213	6.8451e-01	pubmed
ENSMUSG00000055818	A230083G16Rik	-2.512	3.2419e-04	-1.365	2.7510e-01	-0.287	8.1837e-01	pubmed
ENSMUSG00000055862	Izumo4	-1.126	2.0464e-04	-0.870	1.6250e-01	-1.359	2.4217e-02	pubmed
ENSMUSG00000055866	Per2	-2.042	1.0087e-07	-1.028	3.3454e-03	-0.181	9.0740e-01	pubmed
ENSMUSG00000055923	Aasdh	-1.645	7.9694e-15	-1.515	8.8764e-05	-1.075	1.3434e-02	pubmed
ENSMUSG00000056091	St3gal5	-1.500	1.2055e-08	-0.851	5.2842e-02	-0.962	7.8587e-02	pubmed

		CLP1		CLP2		CLP3		
Ensg	Symbol	LFC	FDR	LFC	FDR	LFC	FDR	Link
ENSMUSG00000056148	Rdh9	-0.807	1.5777e-01	-0.982	2.3877e-02	-2.155	8.6235e-05	pubmed
ENSMUSG00000056185	Snx32	-1.067	3.4181e-03	-1.172	9.7502e-02	-1.151	3.3135e-02	pubmed
ENSMUSG00000056216	Cebpg	-0.216	2.4777e-01	-0.544	8.5501e-02	-1.126	3.7845e-04	pubmed
ENSMUSG00000056643	Chst13	-1.351	7.4248e-03	-1.921	1.2948e-03	-1.294	4.8260e-02	pubmed
ENSMUSG00000056665	Them6	-1.223	6.5692e-03	-1.147	2.3550e-01	-1.223	3.4916e-02	pubmed
ENSMUSG00000056698	Elmod3	-1.655	3.5320e-10	-1.353	1.4271e-08	-0.853	4.2977e-03	pubmed
ENSMUSG00000056724	Nbeal2	-1.091	1.0741e-08	-1.396	1.6662e-03	-0.340	5.1213e-01	pubmed
ENSMUSG00000056938	Acbd4	-1.170	1.2063e-19	-1.413	3.5245e-17	-1.119	1.0524e-04	pubmed
ENSMUSG00000056966	Gjc3	-2.055	2.3581e-05	-2.103	5.9990e-05	-1.705	9.4255e-02	pubmed
ENSMUSG00000057037	Cfhr1	-0.572	6.3023e-04	-0.441	1.6559e-01	-1.006	2.6772e-04	pubmed
ENSMUSG00000057054	Inca1	-1.120	3.1360e-07	-0.818	4.5195e-02	-0.812	9.7116e-03	pubmed
ENSMUSG00000057068	Fam47e	-3.362	2.1867e-11	-3.369	3.8654e-13	-1.106	3.2991e-01	pubmed
ENSMUSG00000057342	Sphk2	-0.691	5.1991e-06	-1.216	1.5346e-05	-0.680	5.2028e-02	pubmed
ENSMUSG00000057729	Prtn3	-1.704	4.8107e-02	-4.441	3.5372e-07	-2.957	3.8849e-04	pubmed
ENSMUSG00000057894	Zfp329	-1.090	2.9227e-08	-0.976	1.1028e-01	-1.325	3.1563e-03	pubmed
ENSMUSG00000058331	Zfp85	-1.123	8.2543e-03	0.077	9.2230e-01	-0.046	9.5243e-01	pubmed
ENSMUSG00000058385	Hist1h2bg	-4.816	1.8776e-03	-1.252	1	-1.046	1	pubmed
ENSMUSG00000058396	Gpr182	-1.124	2.1618e-05	-0.901	1.0955e-02	-0.349	2.9520e-01	pubmed
ENSMUSG00000058613	Cyp2d41-ps	-2.629	3.1733e-06	-1.870	4.7733e-03	-2.224	2.2113e-03	pubmed
ENSMUSG00000058620	Adra2b	-6.060	1.5834e-05	-4.183	4.2566e-04	-1.785	1.5012e-01	pubmed
ENSMUSG00000058706	0610030E20Rik	-0.971	8.4828e-09	-1.184	3.2840e-03	-0.589	3.8008e-02	pubmed
ENSMUSG00000058886	Deaf1	-0.995	6.0289e-06	-1.103	4.9068e-05	-0.897	8.8074e-03	pubmed
ENSMUSG00000058921	Slc10a5	-1.297	1.1036e-10	-1.578	2.8121e-09	-1.850	4.1479e-09	pubmed
ENSMUSG00000059456	Ptk2b	-0.838	9.4007e-07	-1.507	5.1536e-12	-1.080	1.5037e-02	pubmed
ENSMUSG00000059689	Zfp637	-0.739	9.2989e-04	-1.031	4.0645e-05	-0.456	1.0776e-01	pubmed
ENSMUSG00000059772	Slx1b	-1.872	1.7728e-08	-2.910	1.1468e-06	-1.498	8.9992e-03	pubmed
ENSMUSG00000059810	Rgs3	-1.388	2.7351e-07	-1.106	3.9234e-03	0.077	9.2201e-01	pubmed
ENSMUSG00000059824	Dbp	-3.168	2.5702e-14	-3.924	3.2839e-26	-1.933	1.0388e-01	pubmed
ENSMUSG00000059939	9430015G10Rik	-1.098	1.9445e-04	-1.227	1.1072e-02	-0.598	2.4536e-01	pubmed
ENSMUSG00000060181	Slc35e3	-1.403	3.8435e-09	-1.531	1.3874e-04	-1.547	2.3505e-03	pubmed
ENSMUSG00000060261	Gtf2i	-1.182	4.5542e-33	-1.362	8.9784e-19	-1.255	3.2538e-06	pubmed
ENSMUSG00000060317	Acnat2	-0.812	3.6623e-01	-1.570	3.6275e-03	-2.095	1.9331e-08	pubmed
ENSMUSG00000060371	Caln1	-0.831	2.4451e-01	-0.472	5.5631e-01	-1.600	2.3083e-03	pubmed
ENSMUSG00000060548	Tnfrsf19	-1.713	1.1569e-03	-0.720	4.3127e-01	-0.265	7.2353e-01	pubmed
ENSMUSG00000060639	Hist1h4i	-2.648	3.8120e-03	-2.711	1.6407e-02	-1.602	1.2830e-01	pubmed
ENSMUSG00000060657	Marf1	-0.736	2.6151e-07	-1.216	1.8984e-07	-0.749	1.2827e-03	pubmed
ENSMUSG00000060862	Zbtb40	-1.328	9.0596e-07	-1.147	1.8879e-01	-0.861	2.4872e-01	pubmed
ENSMUSG00000061013	Mkx	-4.960	4.5148e-04	-1.269	3.3685e-01	-1.578	2.1504e-01	pubmed
ENSMUSG00000061288	Taok3	-1.036	2.0515e-11	-1.205	1.2861e-10	-0.267	4.9549e-01	pubmed
ENSMUSG00000061353	Cxcl12	-1.890	3.8677e-15	-2.008	3.8718e-37	-1.314	4.7273e-09	pubmed
ENSMUSG00000061371	Zfp873	-1.853	3.0301e-05	-1.947	5.3476e-03	-0.934	1.1787e-01	pubmed
ENSMUSG00000061474	Mrps36	-0.595	1.3808e-01	-1.010	4.6094e-03	-0.754	5.7528e-02	pubmed
ENSMUSG00000061533	Cep128	-2.388	5.9737e-04	-0.578	4.7458e-01	-0.626	3.8083e-01	pubmed
ENSMUSG00000061536	Sec22c	-1.507	4.1385e-14	-1.592	8.3611e-08	-0.684	5.8052e-02	pubmed
ENSMUSG00000061601	Pclo	-4.678	1.1291e-03	-3.123	7.9314e-03	-1.281	3.0977e-01	pubmed
ENSMUSG00000061718	Ppp1r1b	-1.767	3.8823e-06	0.434	5.2895e-01	-0.373	7.2236e-01	pubmed
ENSMUSG00000061815	Rufy4	-2.432	9.6800e-03	-1.339	1	-0.606	7.4663e-01	pubmed
ENSMUSG00000061882	Ccdc62	-1.308	1.1943e-04	-0.517	4.5333e-01	-1.018	1.3038e-01	pubmed
ENSMUSG00000061887	Ssbp3	-1.142	2.2439e-11	-1.162	3.9388e-11	-0.455	1.1397e-01	pubmed
ENSMUSG00000062248	Cks2	-1.630	4.0120e-06	-1.235	2.6874e-04	-1.326	3.0617e-03	pubmed
ENSMUSG00000062300	Nectin2	-0.834	3.6116e-06	-1.006	5.6013e-04	-0.731	4.9520e-02	pubmed
ENSMUSG00000062545	Tlr12	-4.739	6.1363e-20	-4.436	2.9983e-13	-1.762	3.4609e-02	pubmed
ENSMUSG00000062563	Cys1	-2.158	3.1770e-04	-3.460	6.2615e-04	-0.242	8.7169e-01	pubmed
ENSMUSG00000062585	Cnr2	-2.116	4.7874e-11	-2.136	4.8773e-06	-1.067	1.4627e-01	pubmed
ENSMUSG00000062646	Ganc	-1.201	5.0283e-13	-1.028	7.3852e-07	-1.026	2.7941e-04	pubmed
ENSMUSG00000062785	Kcnc3	-0.758	1.1282e-02	-1.316	1.2137e-03	0.102	8.4630e-01	pubmed
ENSMUSG00000062949	Atp11c	-1.010	4.6636e-09	-0.772	8.0655e-03	-1.156	8.7735e-07	pubmed
ENSMUSG00000062960	Kdr	-3.146	3.0277e-33	-2.759	3.3068e-16	-1.986	1.9132e-04	pubmed
ENSMUSG00000063146	Clip2	-0.304	4.2539e-01	-1.466	4.5540e-03	-0.306	7.4804e-01	pubmed
ENSMUSG00000063179	Pstk	-1.379	2.2285e-05	-1.642	8.7640e-06	-1.263	4.2977e-03	pubmed
ENSMUSG00000063382	Bcl9l	-1.827	3.2010e-08	-2.196	1.2222e-13	-0.743	4.9669e-01	pubmed
ENSMUSG00000063415	Cyp26b1	-4.557	1.7234e-15	-4.553	3.6355e-08	0.018	9.9616e-01	pubmed
ENSMUSG00000063428	Ddo	-2.013	1.4857e-14	-1.799	3.1251e-09	-1.268	3.6898e-03	pubmed
ENSMUSG00000063455	D630045J12Rik	-1.368	8.4024e-04	-1.410	2.3134e-03	-0.978	1.4301e-02	pubmed
ENSMUSG00000063488	Zkscan7	-1.014	9.5663e-09	-1.226	9.7987e-03	-0.755	1.9735e-01	pubmed
ENSMUSG00000063659	Zbtb18	-0.804	3.5415e-07	-1.198	6.0260e-06	-0.644	1.5639e-01	pubmed
ENSMUSG00000063683	Glyat	-1.289	1.2134e-03	-0.819	1.0869e-04	-1.132	1.0856e-05	pubmed
ENSMUSG00000063704	Mapk15	-0.409	5.4217e-01	-1.914	9.9374e-03	-0.809	3.0005e-01	pubmed
ENSMUSG00000063849	Ppcdc	-1.143	3.0952e-06	-0.909	2.7898e-03	-0.794	1.1423e-02	pubmed
ENSMUSG00000063929	Cyp4a32	-1.037	1.0012e-05	-0.089	8.1684e-01	-0.639	8.9413e-02	pubmed
ENSMUSG00000064225	Paqr9	-2.017	4.8543e-04	-1.599	5.2248e-10	-1.531	1.1325e-06	pubmed
ENSMUSG00000064288	Hist1h4k	-4.978	9.3979e-03	-1.658	1	-3.533	1	pubmed
ENSMUSG00000066026	Dhrs3	-0.350	1.4128e-01	-1.252	1.5842e-03	-1.261	1.8121e-03	pubmed
ENSMUSG00000066235	Pomgnt2	-1.534	1.5871e-05	-1.655	4.1626e-03	-0.728	3.0284e-01	pubmed
ENSMUSG00000066319	Rtp3	-1.120	4.1169e-13	-1.056	1.0755e-03	-0.638	1.4190e-01	pubmed
ENSMUSG00000066477	Gm16551	-1.836	3.5207e-01	-1.987	2.3312e-04	-1.091	3.6762e-01	pubmed
ENSMUSG00000066613	Zfp932	-1.210	1.5085e-04	-1.245	3.6401e-03	-0.970	4.4297e-02	pubmed
ENSMUSG00000066647	Gm5113	-1.305	7.5334e-05	-0.448	3.1444e-01	-0.032	9.5724e-01	pubmed
ENSMUSG00000067071	Hes6	-1.358	1.6087e-08	-1.493	5.4514e-05	-0.867	2.7505e-03	pubmed
ENSMUSG00000067219	Nipal1	-3.458	3.9055e-11	-2.998	4.5119e-26	-2.571	1.3910e-02	pubmed
ENSMUSG00000067279	Ppp1r3c	-2.038	4.2656e-10	-1.859	4.1995e-06	-1.823	2.4451e-06	pubmed
ENSMUSG00000067455	Hist1h4j	-4.682	3.4967e-03	-2.922	1	-3.536	1	pubmed
ENSMUSG00000067629	Syngap1	-1.197	6.9168e-03	-1.148	1.1793e-01	-0.526	5.6321e-01	pubmed
ENSMUSG00000067813	Xkr9	-1.176	4.5859e-02	-1.194	1.8336e-04	-1.512	1.0137e-02	pubmed
ENSMUSG00000067825	Pex26	-1.695	5.6271e-08	-2.084	2.5730e-20	-1.775	8.7735e-07	pubmed
ENSMUSG00000067889	Sptbn2	-1.497	4.3342e-11	-1.984	3.4160e-18	-1.246	1.9439e-05	pubmed
ENSMUSG00000067928	Zfp760	-1.512	7.3530e-10	-2.438	8.3274e-07	-1.283	1.1745e-02	pubmed
ENSMUSG00000068394	Cep152	-1.165	1.7147e-05	-0.892	2.3413e-02	-0.059	8.9740e-01	pubmed
ENSMUSG00000068551	Zfp467	-1.425	3.3610e-08	-1.561	5.0018e-04	-0.935	1.3854e-01	pubmed
ENSMUSG00000068854	Hist2h2be	-3.119	4.6907e-13	-3.106	7.6546e-06	-1.414	1.8434e-01	pubmed
ENSMUSG00000068876	Cgn	-1.024	1.5490e-04	-1.553	1.6689e-10	-0.874	1.0728e-01	pubmed

		CLP1		CLP2		CLP3		
Ensg	Symbol	LFC	FDR	LFC	FDR	LFC	FDR	Link
ENSMUSG00000068923	Syt11	-1.371	9.4950e-05	-1.448	1.4815e-02	-0.906	1.3735e-01	pubmed
ENSMUSG00000069184	Zfp72	-1.133	7.0168e-03	-0.418	4.5747e-01	-0.135	7.8872e-01	pubmed
ENSMUSG00000069208	Zfp825	-1.153	6.3293e-08	-0.561	4.6891e-01	-0.710	1.4100e-01	pubmed
ENSMUSG00000069601	Ank3	-1.537	4.0696e-13	-1.375	2.9581e-02	-1.302	3.8246e-02	pubmed
ENSMUSG00000069733	Ube2u	-0.889	1.9247e-01	-1.242	3.7106e-03	-1.342	9.4629e-03	pubmed
ENSMUSG00000070044	Fam149a	-1.040	3.1495e-07	-1.387	1.2235e-10	-0.995	1.0688e-02	pubmed
ENSMUSG00000070332	Trim80	-5.802	1.3940e-06	-3.169	2.1134e-03	-2.302	1.0230e-01	pubmed
ENSMUSG00000070368	Prok1	-2.399	4.3315e-03	-1.687	3.9274e-02	-2.477	6.6516e-04	pubmed
ENSMUSG00000070371	Prss36	-1.149	2.2547e-03	-1.759	4.9663e-04	-1.253	4.0541e-03	pubmed
ENSMUSG00000070473	Cldn3	-1.945	1.6958e-33	-2.136	2.4235e-08	-1.058	8.0303e-02	pubmed
ENSMUSG00000070565	Rasal2	-1.458	1.4915e-06	-1.309	4.9529e-05	-0.674	7.9971e-02	pubmed
ENSMUSG00000070576	Mn1	-2.634	2.3804e-13	-1.812	3.1146e-04	-0.812	3.3911e-01	pubmed
ENSMUSG00000070808	Bicra	-0.163	5.1563e-01	-1.039	1.7390e-03	-0.115	8.0881e-01	pubmed
ENSMUSG00000070814	Zswim9	-1.157	1.7515e-03	-0.674	3.3027e-01	-0.369	4.3368e-01	pubmed
ENSMUSG00000070873	Lilra5	-1.135	6.2112e-04	-1.083	4.0528e-02	-1.560	3.2254e-03	pubmed
ENSMUSG00000070985	Acnat1	-2.501	1.0007e-37	-2.408	1.9170e-17	-1.961	9.5309e-04	pubmed
ENSMUSG00000071176	Arhgef10	-1.195	5.6313e-04	-0.678	1.5876e-01	-0.065	9.3837e-01	pubmed
ENSMUSG00000071335	Mfsd4b3-ps	-0.986	1.3847e-02	-1.017	3.5005e-03	-1.057	3.5364e-03	pubmed
ENSMUSG00000071547	Nt5dc2	-1.332	3.0993e-05	-1.058	1.3003e-02	-1.142	4.7342e-03	pubmed
ENSMUSG00000072115	Ang	-0.656	2.8864e-15	-0.841	8.2419e-06	-1.323	1.4736e-08	pubmed
ENSMUSG00000072294	Klf12	-0.476	2.5134e-01	-1.205	1.1990e-01	-2.051	4.7392e-06	pubmed
ENSMUSG00000072494	Ppp1r3e	-1.436	1.1101e-03	-1.815	1.1599e-03	-0.992	3.0046e-01	pubmed
ENSMUSG00000072568	Fam84b	-1.838	5.2300e-08	-0.197	6.6904e-01	0.190	7.9495e-01	pubmed
ENSMUSG00000072571	Tmem253	-2.108	3.4265e-05	-1.720	1.3234e-01	-0.559	5.9690e-01	pubmed
ENSMUSG00000072572	Slc39a2	-1.552	2.8161e-03	-3.080	5.7362e-02	-2.014	5.1926e-02	pubmed
ENSMUSG00000072596	Ear2	-1.922	1.0403e-05	-0.418	5.3574e-01	-0.473	6.2735e-01	pubmed
ENSMUSG00000072640	Lyrm9	-1.198	4.7676e-06	-1.107	3.2812e-03	-0.625	1.7927e-01	pubmed
ENSMUSG00000072673	Gm10392	-3.349	7.7426e-03	-2.481	9.1311e-02	-1.822	9.9032e-02	pubmed
ENSMUSG00000072704	Smim10l1	-0.676	2.2795e-08	-0.872	8.5520e-10	-1.003	6.8495e-04	pubmed
ENSMUSG00000072762	4930522L14Rik	-1.458	7.3076e-03	-1.011	2.2505e-01	-0.683	3.2065e-01	pubmed
ENSMUSG00000072763	5430403G16Rik	-1.485	2.9045e-03	-1.827	1.9692e-02	-0.656	4.5587e-01	pubmed
ENSMUSG00000072884	Gm10433	-2.308	1.8635e-03	-1.030	4.2769e-01	-0.557	4.9512e-01	pubmed
ENSMUSG00000072893	4933439C10Rik	-1.336	5.6793e-04	-1.020	2.6215e-01	-1.090	9.5816e-02	pubmed
ENSMUSG00000073002	Vamp5	-1.095	9.6466e-05	-1.812	1.5296e-03	-1.170	2.7992e-02	pubmed
ENSMUSG00000073236	2500004C02Rik	-1.661	3.1809e-03	-1.882	3.8299e-02	-2.263	1.0511e-02	pubmed
ENSMUSG00000073424	Cyp4f15	-0.984	2.2958e-11	-1.168	7.9333e-03	-1.399	4.3160e-05	pubmed
ENSMUSG00000073436	Eme2	-1.154	1.0201e-06	-1.476	1.3668e-03	-0.824	1.9574e-01	pubmed
ENSMUSG00000073437	D330041H03Rik	-2.739	7.5896e-05	-1.850	2.4787e-01	-1.715	1.2701e-01	pubmed
ENSMUSG00000073555	Gm4951	-0.321	3.2004e-01	-0.638	4.8888e-06	-1.138	2.3419e-03	pubmed
ENSMUSG00000073600	Prob1	-1.576	1.4958e-04	-1.957	3.1794e-04	-1.009	2.0161e-01	pubmed
ENSMUSG00000073678	Pgap1	-1.588	5.1186e-09	-2.765	1.1326e-29	-1.868	1.3012e-07	pubmed
ENSMUSG00000073680	Tmem88b	-2.006	1.1018e-04	-0.993	3.8123e-01	-0.801	4.3276e-01	pubmed
ENSMUSG00000073755	5730409E04Rik	-0.674	3.4735e-02	-1.104	3.6789e-03	0.088	8.8164e-01	pubmed
ENSMUSG00000073771	Btbd19	-1.222	2.1124e-05	0.133	8.8194e-01	1.016	1.9730e-01	pubmed
ENSMUSG00000073910	Mob3b	-1.056	1.0539e-11	-1.279	6.8618e-05	-0.856	3.3368e-02	pubmed
ENSMUSG00000074052	BC048644	-5.844	1.3658e-06	-6.261	4.8112e-05	-5.263	1	pubmed
ENSMUSG00000074064	Mlycd	-1.151	5.7255e-17	-1.222	2.9887e-04	-0.995	1.0212e-04	pubmed
ENSMUSG00000074071	Fam169b	-1.432	3.1770e-04	-0.857	1.1055e-02	-0.553	3.8557e-01	pubmed
ENSMUSG00000074166	AW146154	-1.124	5.8553e-06	-1.049	7.2373e-03	-0.889	1.5366e-01	pubmed
ENSMUSG00000074211	Sdhaf1	-1.203	5.5562e-03	-1.465	3.4374e-04	-0.567	1.6264e-01	pubmed
ENSMUSG00000074277	Phldb3	-0.741	2.9579e-02	-1.310	3.8588e-03	-0.298	5.7534e-01	pubmed
ENSMUSG00000074284	Gm10658	-2.710	4.1711e-07	-2.208	7.1420e-06	-1.142	1.3358e-01	pubmed
ENSMUSG00000074361	C5ar2	-2.490	1.2091e-08	-2.484	1.1061e-03	-2.276	9.4629e-03	pubmed
ENSMUSG00000074491	Clec4g	-1.398	8.9283e-07	-1.089	1.3914e-04	-1.731	2.5458e-06	pubmed
ENSMUSG00000074622	Mafb	-0.541	1.9386e-01	-1.283	4.5599e-04	-1.020	7.2330e-02	pubmed
ENSMUSG00000074646	6430550D23Rik	-2.760	6.1152e-04	-1.660	1.9891e-01	-1.157	2.6425e-01	pubmed
ENSMUSG00000074785	Plxnc1	-1.706	8.5331e-09	-0.683	1.4825e-01	-0.861	3.2661e-03	pubmed
ENSMUSG00000074794	Arrdc3	-0.882	2.8620e-01	-2.586	8.8275e-10	-0.913	6.4861e-02	pubmed
ENSMUSG00000074824	Rslcan18	-2.135	1.0714e-02	-1.207	3.5937e-01	-3.706	4.2601e-03	pubmed
ENSMUSG00000074916	Chst14	-1.293	3.6854e-03	-0.974	1.1639e-01	0.352	6.4436e-01	pubmed
ENSMUSG00000075225	Ccdc162	-1.235	4.0762e-05	-0.765	4.4056e-02	-1.016	2.5605e-03	pubmed
ENSMUSG00000075273	Ttc30b	-1.097	1.1488e-04	-0.581	1.3962e-01	-0.521	2.0570e-01	pubmed
ENSMUSG00000075304	Sp5	-3.796	2.7919e-03	-2.993	3.9197e-02	-0.311	1	pubmed
ENSMUSG00000075324	Fign	-1.976	6.0018e-09	-1.321	3.6435e-03	-0.500	5.7782e-01	pubmed
ENSMUSG00000075389	2810410L24Rik	-1.765	9.9033e-06	-1.532	1.1566e-02	-1.161	1.2707e-01	pubmed
ENSMUSG00000075419	Dolk	-0.939	3.2406e-06	-1.222	1.1873e-05	-1.378	1.8431e-02	pubmed
ENSMUSG00000075511	1700001L05Rik	-1.331	1.1913e-04	-1.251	1.7713e-03	-0.090	8.1942e-01	pubmed
ENSMUSG00000075517	Cyp2d37-ps	-2.915	1.7284e-11	-2.006	2.6918e-07	-1.343	1.2141e-02	pubmed
ENSMUSG00000075543	Urad	-1.216	1.3424e-07	-0.861	2.9056e-04	-0.978	8.5803e-05	pubmed
ENSMUSG00000075592	Nynrin	-3.331	1.0385e-04	-1.706	1.0385e-01	-0.671	6.0503e-01	pubmed
ENSMUSG00000075703	Selenoi	-1.067	1.9769e-12	-1.137	5.6854e-13	-0.735	5.9958e-03	pubmed
ENSMUSG00000078234	Klhdc7a	-2.149	1.0135e-06	-1.944	8.9057e-05	-0.697	5.4232e-01	pubmed
ENSMUSG00000078350	Smim1	-1.281	4.4640e-03	-0.856	3.3199e-01	-0.404	6.5326e-01	pubmed
ENSMUSG00000078552	Dcdc2b	-1.686	9.8626e-03	-4.712	9.9092e-03	-0.817	6.0577e-01	pubmed
ENSMUSG00000078584	AU022252	-0.766	2.8167e-04	-1.124	1.6557e-03	-0.649	2.0272e-02	pubmed
ENSMUSG00000078607	1810010H24Rik	-1.406	3.8525e-03	-0.669	2.8531e-01	-0.557	5.1232e-01	pubmed
ENSMUSG00000078650	G6pc	-2.705	9.3073e-03	-0.269	8.0671e-01	0.231	7.4366e-01	pubmed
ENSMUSG00000078716	Tmem8b	-1.520	6.8136e-05	-1.183	4.5565e-02	-0.291	6.6665e-01	pubmed
ENSMUSG00000078851	Hist3h2a	-2.945	1.9385e-08	-1.739	6.5449e-02	-1.336	2.0570e-01	pubmed
ENSMUSG00000078861	Zfp931	-1.520	5.9324e-04	-1.592	1.4671e-05	-1.302	3.3897e-02	pubmed
ENSMUSG00000078963	Hsbp1l1	-1.397	6.4077e-03	-1.195	1.7990e-02	-0.986	8.5219e-02	pubmed
ENSMUSG00000079003	Samd1	-1.588	1.0572e-06	-3.352	5.3215e-09	-1.266	4.0634e-02	pubmed
ENSMUSG00000079045	Prox1os	-1.365	3.4373e-04	-1.509	2.5386e-04	-1.086	6.1522e-02	pubmed
ENSMUSG00000079144	A130010J15Rik	-1.233	3.7540e-05	-0.619	1.2024e-01	0.012	9.8916e-01	pubmed
ENSMUSG00000079164	Tlr5	-2.430	3.2364e-17	-1.403	1.8063e-02	-0.294	6.1028e-01	pubmed
ENSMUSG00000079227	Ccr5	-1.922	3.8991e-10	-1.361	1.5506e-03	-1.480	1.0366e-02	pubmed
ENSMUSG00000079334	Naa80	-1.159	3.5501e-10	-0.607	4.6249e-04	-0.513	5.8034e-02	pubmed
ENSMUSG00000079434	Neu2	-2.512	4.6386e-04	-2.838	1.0536e-02	-0.701	5.2673e-01	pubmed
ENSMUSG00000079470	Utp14b	-1.073	7.7841e-06	-0.725	5.6556e-02	0.127	8.2971e-01	pubmed
ENSMUSG00000081058	Hist2h3c2	-7.166	1.7318e-07	-4.783	5.1738e-04	-2.479	7.4402e-03	pubmed
ENSMUSG00000081988	Gm15746	-2.508	3.4186e-04	-1.382	1.2029e-01	-2.819	6.8166e-03	pubmed

		CLP1		CLP2		CLP3		
Ensg	Symbol	LFC	FDR	LFC	FDR	LFC	FDR	Link
ENSMUSG00000083396	Gm15542	-0.744	9.2395e-02	-0.833	1.5453e-02	-1.027	7.7177e-03	pubmed
ENSMUSG00000083813	Gm15502	-1.765	8.7159e-03	-2.507	8.6786e-03	-1.892	6.6188e-02	pubmed
ENSMUSG00000084128	Esrp2	-1.142	2.3050e-10	-1.411	5.6940e-16	-0.979	7.9304e-02	pubmed
ENSMUSG00000084822	Myadml2os	-4.994	1.2434e-03	-4.068	1	-1.378	1	pubmed
ENSMUSG00000084883	Ccdc85c	-1.302	3.4327e-06	-2.052	4.6624e-07	-0.714	2.0992e-01	pubmed
ENSMUSG00000084923	Gm15611	-0.633	5.3481e-01	-1.324	1.6740e-01	-3.474	4.9982e-04	pubmed
ENSMUSG00000084939	Gm830	-4.112	6.6840e-03	-2.068	1	0.201	1	pubmed
ENSMUSG00000084983	Gm11789	-1.871	2.2052e-13	-1.541	5.7437e-16	-1.279	3.5531e-04	pubmed
ENSMUSG00000085001	Rapgef4os2	-0.873	4.2044e-01	-0.247	9.0293e-01	-3.050	7.2006e-03	pubmed
ENSMUSG00000085028	Slc2a4rg-ps	-2.154	2.7445e-14	-1.520	1.5207e-02	-0.409	5.4240e-01	pubmed
ENSMUSG00000085037	4933421O10Rik	-1.223	7.3752e-07	-0.822	9.2042e-02	-0.596	2.2881e-01	pubmed
ENSMUSG00000085095	Gm15635	-1.742	5.4348e-04	-1.315	1.6593e-01	-0.217	8.4873e-01	pubmed
ENSMUSG00000085151	1110018N20Rik	-4.238	6.0292e-03	-1.422	4.2614e-01	-1.560	1	pubmed
ENSMUSG00000085175	Gm11423	-1.938	2.4988e-04	-1.573	7.4740e-02	-1.075	2.9922e-01	pubmed
ENSMUSG00000085208	Brip1os	-0.687	3.8469e-05	-1.205	1.0508e-03	-1.163	1.7911e-02	pubmed
ENSMUSG00000085287	4833418N02Rik	-1.591	1.2836e-06	-0.731	1.3609e-01	-0.020	9.8086e-01	pubmed
ENSMUSG00000085404	Gm12909	-1.712	9.5537e-04	-1.776	5.6325e-03	-2.152	8.2038e-04	pubmed
ENSMUSG00000085438	1700020I14Rik	-0.960	5.6115e-16	-1.169	2.7763e-03	-0.991	1.0167e-02	pubmed
ENSMUSG00000085445	Gm16348	-1.577	2.4846e-06	-0.666	7.4914e-02	-1.389	3.2538e-06	pubmed
ENSMUSG00000085468	Gm15343	-4.025	4.6942e-04	-4.100	3.2243e-04	-1.767	2.0593e-01	pubmed
ENSMUSG00000085558	4930412C18Rik	-1.954	1.5327e-04	-1.760	4.9085e-04	-0.842	1.3982e-01	pubmed
ENSMUSG00000085566	A730017L22Rik	-1.731	5.7915e-06	-1.890	6.1554e-10	-1.150	7.8234e-02	pubmed
ENSMUSG00000085826	Gm15638	-1.789	1.0140e-03	-2.474	1.2760e-06	-2.920	1.0413e-09	pubmed
ENSMUSG00000085834	Gm15622	-4.404	2.0007e-03	-5.006	1.1604e-02	-4.848	2.4988e-03	pubmed
ENSMUSG00000085882	2610507I01Rik	-2.460	9.4850e-09	-2.322	4.5271e-05	-1.207	1.6893e-01	pubmed
ENSMUSG00000085929	Gm13421	-2.312	1.3879e-05	-2.181	7.5615e-02	-1.063	2.0143e-01	pubmed
ENSMUSG00000085990	Gm16731	-2.381	1.7490e-04	-2.562	1.0795e-01	-1.205	2.5877e-01	pubmed
ENSMUSG00000085995	Gm2788	-0.866	4.4852e-03	-1.107	1.5473e-02	-2.071	9.9280e-10	pubmed
ENSMUSG00000086010	Gm15318	-3.717	7.7569e-06	-3.161	9.1783e-07	-2.866	2.9078e-05	pubmed
ENSMUSG00000086040	Wipf3	-1.371	3.9765e-03	0.097	9.1183e-01	-0.135	8.5654e-01	pubmed
ENSMUSG00000086071	Gm12354	-3.190	7.4172e-04	-4.109	8.0679e-05	-3.715	3.6161e-03	pubmed
ENSMUSG00000086141	9030622O22Rik	-2.150	1.2200e-07	-0.803	1.0244e-01	-1.323	9.8440e-04	pubmed
ENSMUSG00000086213	A330040F15Rik	-5.688	1.4229e-04	-2.436	3.7047e-02	-1.667	1.9354e-01	pubmed
ENSMUSG00000086231	Rapgef4os3	-1.052	2.3935e-03	-1.227	2.8690e-02	-1.883	6.8495e-04	pubmed
ENSMUSG00000086296	D030055H07Rik	-1.839	1.0400e-04	-0.757	5.2346e-01	-0.949	1.6689e-01	pubmed
ENSMUSG00000086390	1810019D21Rik	-2.693	1.7303e-11	-2.753	5.9713e-06	-1.296	3.4691e-01	pubmed
ENSMUSG00000086432	B430119L08Rik	-2.378	1.3827e-25	-2.171	1.3944e-02	-1.951	3.6827e-01	pubmed
ENSMUSG00000086507	Adap2os	-1.423	6.3632e-04	-1.457	6.5328e-02	-1.169	9.7968e-02	pubmed
ENSMUSG00000086593	Gm16548	-1.373	1.3755e-03	-1.641	4.9421e-04	-1.921	1.0023e-03	pubmed
ENSMUSG00000086628	Gm16157	-1.211	9.8727e-05	-0.793	6.2293e-02	-0.893	1.6749e-02	pubmed
ENSMUSG00000086859	Snhg20	-1.053	7.0593e-04	-0.101	9.0518e-01	-0.359	5.3972e-01	pubmed
ENSMUSG00000086868	Gm15883	-1.247	3.2368e-04	0.009	9.8721e-01	0.129	8.3017e-01	pubmed
ENSMUSG00000086877	A230072C01Rik	-0.355	3.9753e-01	-0.709	2.7997e-01	-1.552	1.0463e-03	pubmed
ENSMUSG00000086924	Gm11766	-4.726	1.9910e-03	-2.648	1	-2.472	1	pubmed
ENSMUSG00000086968	4933431E20Rik	-1.546	3.0337e-04	-1.682	2.6934e-03	-0.406	4.7079e-01	pubmed
ENSMUSG00000087030	Gm16143	-3.935	3.1276e-03	-2.239	1	-2.204	1	pubmed
ENSMUSG00000087138	Gm15545	-2.097	1.2591e-04	-2.045	3.0186e-02	-0.769	3.1629e-01	pubmed
ENSMUSG00000087141	Plcxd2	-1.017	4.6107e-05	-1.378	1.0855e-02	-1.313	7.5721e-04	pubmed
ENSMUSG00000087165	2010001A14Rik	-1.412	2.4597e-03	-0.814	2.3961e-01	-0.580	3.7227e-01	pubmed
ENSMUSG00000087178	A230056P14Rik	-3.804	2.2486e-03	-4.005	3.9754e-03	-1.731	6.8066e-02	pubmed
ENSMUSG00000087213	2810408I11Rik	-2.293	3.3821e-04	-2.033	6.4444e-04	-0.740	2.6469e-01	pubmed
ENSMUSG00000087241	Gm11844	-1.931	3.6917e-04	-2.594	4.6247e-05	-1.828	1.3125e-02	pubmed
ENSMUSG00000087403	Kantr	-1.945	3.8951e-05	-1.748	6.5918e-03	-1.060	5.0702e-02	pubmed
ENSMUSG00000087404	Gm11752	-3.082	2.8714e-03	-2.127	9.1352e-02	-1.314	2.8414e-01	pubmed
ENSMUSG00000087424	5730405O15Rik	-2.741	4.0060e-03	-3.903	3.4852e-02	-0.458	1	pubmed
ENSMUSG00000087439	Gm15788	-3.010	4.6298e-04	-2.997	9.2880e-02	-2.094	1.6096e-01	pubmed
ENSMUSG00000087516	Tbx3os1	-2.414	4.1357e-06	-2.042	1.1480e-01	-3.311	2.9508e-03	pubmed
ENSMUSG00000087672	Gm15122	-1.802	9.5582e-03	-1.535	3.5243e-01	-0.555	6.3211e-01	pubmed
ENSMUSG00000089635	Gm16559	-2.689	5.5457e-04	-5.598	6.5491e-04	-2.297	3.3092e-02	pubmed
ENSMUSG00000089712	Gm15889	-1.400	2.7229e-03	-2.766	5.0423e-03	-0.844	5.4582e-01	pubmed
ENSMUSG00000089715	Cbx6	-1.727	1.9098e-10	-0.940	2.6391e-02	-0.446	3.4237e-01	pubmed
ENSMUSG00000089774	Slc5a3	-0.549	2.7145e-01	-2.404	1.0259e-07	-1.369	5.8719e-02	pubmed
ENSMUSG00000089810	Gm16536	-0.670	2.8969e-03	-1.148	2.7890e-03	-0.868	5.0383e-02	pubmed
ENSMUSG00000090019	Gimap1	-1.342	2.0672e-03	-1.073	1.6567e-01	-1.179	1.3478e-01	pubmed
ENSMUSG00000090038	Gm16573	-1.788	1.8013e-13	-1.732	1.9676e-04	-1.812	1.1906e-01	pubmed
ENSMUSG00000090086	AI480526	-2.031	3.6640e-19	-1.663	8.3974e-04	-1.062	7.1645e-02	pubmed
ENSMUSG00000090210	Itga10	-1.110	6.9337e-03	-0.987	2.9057e-01	-1.005	1.0718e-01	pubmed
ENSMUSG00000090306	Adh6-ps1	-2.906	4.7989e-16	-2.820	2.8302e-21	-2.457	5.8510e-04	pubmed
ENSMUSG00000090555	Gm8893	-0.809	1.2050e-06	-0.640	1.8288e-02	-1.215	7.7099e-06	pubmed
ENSMUSG00000090622	A930033H14Rik	-2.348	3.7769e-05	-2.468	1.4126e-02	-2.044	1.2279e-02	pubmed
ENSMUSG00000090659	Zfp493	-2.078	1.9701e-04	-1.532	3.3412e-02	-0.745	5.4868e-01	pubmed
ENSMUSG00000090877	Hspa1b	-1.172	2.5658e-02	-2.646	4.2092e-05	-1.430	3.7596e-02	pubmed
ENSMUSG00000090942	F830016B08Rik	-0.110	8.8951e-01	-0.609	6.0630e-02	-1.506	1.1919e-04	pubmed
ENSMUSG00000091405	Hist2h4	-4.931	1.0932e-03	-2.644	1	-2.721	1	pubmed
ENSMUSG00000091474	2610021A01Rik	-1.362	4.2309e-08	-1.082	8.8393e-03	-0.766	2.1713e-01	pubmed
ENSMUSG00000091721	Gimd1	-2.078	8.1387e-04	-1.891	1.0772e-07	-1.690	3.3179e-04	pubmed
ENSMUSG00000092274	Neat1	-1.139	2.1229e-04	-0.695	4.3412e-02	-0.463	3.0161e-01	pubmed
ENSMUSG00000092471	Cyp21a2-ps	-0.533	9.0328e-02	-1.447	7.1612e-03	-1.095	2.1195e-02	pubmed
ENSMUSG00000092545	Gm20319	-1.585	5.0657e-07	-1.066	8.2604e-04	-1.414	1.8240e-06	pubmed
ENSMUSG00000093405	Gm20684	-0.643	4.4822e-01	-1.892	5.3948e-03	-0.954	2.3624e-01	pubmed
ENSMUSG00000093507	Gm20627	-4.219	8.8005e-03	-3.411	1	-0.084	1	pubmed
ENSMUSG00000093606	B130034C11Rik	-2.942	7.8759e-03	-3.085	5.9037e-02	-1.744	1	pubmed
ENSMUSG00000095026	Gm3336	-2.951	1.5551e-03	-3.455	4.6337e-02	0.368	7.8876e-01	pubmed
ENSMUSG00000095041	AC149090.1	-1.195	3.1245e-12	-1.427	2.1431e-06	-0.948	1.7952e-02	pubmed
ENSMUSG00000095098	Ccdc85b	-0.908	7.7925e-04	-1.775	3.1959e-05	-0.623	4.3351e-01	pubmed
ENSMUSG00000096606	Tpbgl	-3.258	3.0610e-14	-2.368	2.9368e-09	-1.493	3.9457e-02	pubmed
ENSMUSG00000096917	2500002B13Rik	-2.198	1.5573e-03	-1.632	1.1560e-01	-2.090	2.6243e-02	pubmed
ENSMUSG00000096971	4930556M19Rik	-2.012	6.6145e-10	-2.246	2.3427e-06	-1.100	2.0622e-01	pubmed
ENSMUSG00000097042	Gm17491	-1.017	7.6011e-06	-0.587	2.3210e-01	-0.337	4.6708e-01	pubmed
ENSMUSG00000097061	9330151L19Rik	-1.087	4.9445e-05	-0.974	1.6090e-01	-0.884	6.7331e-02	pubmed
ENSMUSG00000097101	1810034E14Rik	-3.048	6.8136e-08	-3.444	1.5834e-07	-1.470	4.2626e-02	pubmed

		CLP1		CLP2		CLP3		
Ensg	Symbol	LFC	FDR	LFC	FDR	LFC	FDR	Link
ENSMUSG00000097119	B230354K17Rik	-1.584	3.8737e-09	-1.916	1.7354e-03	-1.392	4.0025e-02	pubmed
ENSMUSG00000097121	D130020L05Rik	-0.962	5.4351e-03	-1.223	5.7600e-03	-1.457	4.4068e-03	pubmed
ENSMUSG00000097123	Gm6297	-2.465	2.3055e-03	-5.469	8.7484e-04	-0.602	7.5524e-01	pubmed
ENSMUSG00000097131	D230017M19Rik	-2.784	1.5505e-16	-2.179	4.8164e-05	-1.076	1.9710e-02	pubmed
ENSMUSG00000097145	9230114K14Rik	-1.941	3.0867e-03	-1.863	7.6376e-03	-0.978	1.9245e-01	pubmed
ENSMUSG00000097164	Cep83os	-1.511	1.9677e-05	-1.472	2.7372e-03	-0.538	3.4604e-01	pubmed
ENSMUSG00000097204	Gm17690	-1.734	5.6955e-03	-2.563	4.1804e-02	-1.273	2.6544e-01	pubmed
ENSMUSG00000097284	4930480K23Rik	-2.735	1.7387e-03	-2.128	8.7851e-03	-1.555	6.6275e-02	pubmed
ENSMUSG00000097286	Gm26684	-1.339	1.0325e-01	-3.459	3.2517e-04	-0.034	9.8397e-01	pubmed
ENSMUSG00000097290	1300002E11Rik	-0.722	3.9635e-04	-1.038	4.7661e-03	-0.601	9.5057e-02	pubmed
ENSMUSG00000097294	Gm26888	-2.287	4.4006e-03	-2.818	1	-1.579	1	pubmed
ENSMUSG00000097310	A930038B10Rik	-5.041	7.9757e-05	-6.721	7.0363e-05	-3.998	1.3353e-02	pubmed
ENSMUSG00000097321	1700028E10Rik	-1.676	6.2181e-08	-1.626	7.6689e-06	-1.683	4.4954e-03	pubmed
ENSMUSG00000097353	A430046D13Rik	-2.257	3.5298e-04	-2.457	4.0983e-03	-2.090	2.5802e-02	pubmed
ENSMUSG00000097354	2310001H17Rik	-0.840	9.7941e-04	-0.780	1.2805e-01	-2.064	4.9033e-07	pubmed
ENSMUSG00000097383	1500026H17Rik	-3.104	9.2925e-04	-1.960	2.3433e-02	-1.664	1.2183e-01	pubmed
ENSMUSG00000097426	Gm8941	-3.051	2.5922e-20	-3.536	2.6810e-10	-4.802	8.2302e-13	pubmed
ENSMUSG00000097503	3110045C21Rik	-0.823	4.1399e-01	-2.670	4.4805e-04	-2.006	2.4217e-02	pubmed
ENSMUSG00000097504	4930516B21Rik	-1.551	2.1915e-03	-1.237	2.1781e-01	-1.324	3.0228e-01	pubmed
ENSMUSG00000097673	Gm26608	-3.321	5.9179e-05	-6.791	2.4111e-06	-3.354	3.2044e-03	pubmed
ENSMUSG00000097691	9030616G12Rik	-4.641	3.7561e-04	-4.988	3.4946e-06	-2.991	2.9633e-03	pubmed
ENSMUSG00000097706	E030037K01Rik	-4.664	3.0658e-03	-1.754	3.0332e-01	-2.280	1	pubmed
ENSMUSG00000097730	Gm26588	-4.940	2.4433e-14	-4.456	1.6520e-13	-1.433	2.5152e-02	pubmed
ENSMUSG00000097762	4732463B04Rik	-3.100	1.6877e-04	-1.929	1.5784e-01	0.527	4.7356e-01	pubmed
ENSMUSG00000097785	B230217O12Rik	-1.915	3.8239e-03	-2.567	5.9299e-02	-1.520	1.4032e-01	pubmed
ENSMUSG00000097811	2810425M01Rik	-2.868	6.1107e-05	-2.685	3.3863e-03	-1.530	3.9691e-02	pubmed
ENSMUSG00000097867	Lppos	-1.333	5.1402e-04	-1.907	2.8879e-04	-0.954	8.3009e-02	pubmed
ENSMUSG00000097908	4933404O12Rik	-3.199	2.7970e-12	-2.770	6.9634e-04	-1.346	1.4710e-02	pubmed
ENSMUSG00000097930	C330002G04Rik	-1.350	7.6971e-03	-1.551	1.3084e-02	-2.048	1.7427e-03	pubmed
ENSMUSG00000097974	Gm10605	-4.162	5.6316e-05	-2.436	1.2044e-02	-1.009	4.8462e-01	pubmed
ENSMUSG00000098014	Gm26967	-2.538	4.9965e-02	-4.945	9.3333e-03	-0.698	5.8712e-01	pubmed
ENSMUSG00000098112	Bin2	-1.315	5.6354e-04	-0.523	3.2321e-01	-0.747	7.1843e-02	pubmed
ENSMUSG00000098146	Gm26935	-2.654	2.5808e-03	-0.179	1	-0.078	9.5442e-01	pubmed
ENSMUSG00000098176	Ccdc166	-2.911	3.4950e-06	-2.164	4.2784e-03	-0.533	5.5705e-01	pubmed
ENSMUSG00000098678	Mroh6	-4.251	1.4205e-06	-2.160	3.1035e-02	-2.741	1.2901e-02	pubmed
ENSMUSG00000098747	Gm27216	-0.560	1.7979e-01	-0.692	6.6031e-03	-1.171	5.5879e-05	pubmed
ENSMUSG00000098867	Gm27463	-2.667	8.1467e-07	-1.337	3.3459e-02	-1.436	4.6087e-02	pubmed
ENSMUSG00000099034	2810039B14Rik	-1.176	5.1140e-03	-0.443	6.0898e-01	-0.064	9.3520e-01	pubmed
ENSMUSG00000099146	0610031O16Rik	-1.586	3.4038e-09	-1.366	8.9646e-04	-2.049	2.8758e-07	pubmed
ENSMUSG00000099881	2810013P06Rik	-1.518	1.3461e-14	-1.615	5.8893e-05	-1.397	6.1491e-03	pubmed
ENSMUSG00000099966	2810402E24Rik	-1.074	3.4536e-04	-0.508	2.0950e-01	-0.373	5.3481e-01	pubmed
ENSMUSG00000100005	B130024G19Rik	-4.208	4.8258e-04	-3.570	2.1941e-02	-0.151	9.2845e-01	pubmed
ENSMUSG00000100094	1810008I18Rik	-0.408	6.0881e-02	-0.696	5.0168e-02	-1.123	8.5969e-03	pubmed
ENSMUSG00000100277	1810053B23Rik	-3.791	8.1451e-06	-4.839	6.0346e-07	-2.675	2.0272e-02	pubmed
ENSMUSG00000100586	Vmn1r90	-5.395	1.2179e-04	-2.954	1	-3.945	1	pubmed
ENSMUSG00000100594	2810414N06Rik	-1.971	2.4635e-04	-1.511	2.2332e-02	-1.565	1.7952e-02	pubmed
ENSMUSG00000100975	Gm28875	-1.833	1.6331e-03	-0.559	5.6194e-01	-1.262	1.4555e-01	pubmed
ENSMUSG00000101210	Gm28720	-3.464	8.9811e-04	-4.784	1.1900e-02	-3.129	1	pubmed
ENSMUSG00000101225	1700008J07Rik	-3.719	3.3106e-03	-2.534	1.5554e-02	-1.129	3.3203e-01	pubmed
ENSMUSG00000101397	Mug-ps1	-1.164	3.1920e-06	0.165	8.4745e-01	-0.383	2.7899e-01	pubmed
ENSMUSG00000101514	Gm5524	-4.164	7.8040e-07	-5.299	1.0187e-13	-2.821	4.7792e-03	pubmed
ENSMUSG00000101599	Gm20342	-1.687	7.7749e-08	-0.999	1.2365e-01	-0.738	1.8314e-01	pubmed
ENSMUSG00000102091	Olfr1034	-2.058	3.9195e-03	-2.314	2.3962e-02	-2.036	5.7394e-02	pubmed
ENSMUSG00000102095	C730036E19Rik	-3.142	5.0408e-11	-3.628	5.0289e-09	-3.473	1.3615e-07	pubmed
ENSMUSG00000102101	Zbtb11os1	-1.453	8.9111e-03	-0.753	2.4046e-01	-0.368	7.2282e-01	pubmed
ENSMUSG00000102353	Gm38345	-4.903	2.3914e-11	-3.195	3.0260e-09	-2.319	7.1721e-03	pubmed
ENSMUSG00000102881	Gm3807	-4.284	3.3095e-03	-3.428	1	-3.942	1	pubmed
ENSMUSG00000103222	Gm37729	-3.042	1.2543e-08	-2.837	2.9812e-04	-2.889	2.3505e-03	pubmed
ENSMUSG00000103421	Golt1a	-0.882	3.9222e-02	-1.405	4.0398e-03	-0.957	2.7420e-01	pubmed
ENSMUSG00000103680	Gm37072	-2.297	1.7064e-03	-2.160	9.0931e-02	-2.475	2.0090e-03	pubmed
ENSMUSG00000103839	Gm37607	-2.245	3.5294e-03	-2.191	1.3979e-01	-2.722	4.4142e-02	pubmed
ENSMUSG00000103898	Gm30238	-1.417	2.1824e-03	-0.562	6.0992e-01	-1.484	4.0783e-02	pubmed
ENSMUSG00000103928	Gm37893	-1.208	4.2652e-05	-2.012	7.7611e-03	-1.122	9.6646e-02	pubmed
ENSMUSG00000103983	Gm20045	-2.157	8.3540e-08	-2.125	4.6307e-02	-1.947	7.3324e-02	pubmed
ENSMUSG00000104011	Gm32391	-4.063	7.4035e-03	-1.631	3.1841e-01	-1.987	1	pubmed
ENSMUSG00000104156	Gm38102	-1.429	1.3391e-04	-1.255	2.0710e-01	-0.525	5.7754e-01	pubmed
ENSMUSG00000104392	Gm37962	-2.559	7.0683e-04	-1.021	4.9874e-01	-0.337	8.0944e-01	pubmed
ENSMUSG00000104528	Gm43314	-2.113	1.1976e-03	-2.672	3.8414e-02	-2.725	2.2448e-02	pubmed
ENSMUSG00000104664	Gm35570	-1.579	2.6907e-03	-1.539	6.4246e-02	-1.470	2.9748e-01	pubmed
ENSMUSG00000105095	8430422M14Rik	-1.539	8.8495e-04	0.240	7.8655e-01	-0.747	3.2722e-01	pubmed
ENSMUSG00000105837	Gm35986	-0.676	3.9006e-01	-1.219	1.6058e-03	-0.777	2.4255e-01	pubmed
ENSMUSG00000105881	4932422M17Rik	-1.800	1.2632e-06	-2.879	1.3624e-03	-0.482	6.1774e-01	pubmed
ENSMUSG00000106087	Gm43609	-1.235	3.5336e-04	-0.831	3.5176e-01	-1.093	2.9714e-02	pubmed
ENSMUSG00000106303	Gm7652	-2.356	3.0813e-04	-1.058	2.5262e-01	-1.319	3.7118e-01	pubmed
ENSMUSG00000106397	Gm21049	-2.469	3.8980e-03	-2.581	2.0611e-03	-1.489	2.7465e-01	pubmed
ENSMUSG00000106464	C130083M11Rik	-2.359	8.7191e-06	-2.131	1.2145e-02	-1.069	2.7149e-01	pubmed
ENSMUSG00000106526	Gm42604	-1.491	8.0139e-04	-0.901	2.3029e-01	-0.339	7.0753e-01	pubmed
ENSMUSG00000106825	2510016D11Rik	-4.049	1.1979e-03	-2.827	5.2542e-02	-1.549	1.9521e-01	pubmed
ENSMUSG00000106838	1810017P11Rik	-4.614	4.5339e-04	-2.575	1.0128e-01	-3.488	2.1874e-03	pubmed
ENSMUSG00000106889	Gm7463	-6.011	3.4265e-05	-5.015	1.8209e-03	-1.796	1	pubmed
ENSMUSG00000106896	G630022F23Rik	-4.534	3.2704e-03	-1.246	1	-2.940	1	pubmed
ENSMUSG00000106951	5930430L01Rik	-3.146	3.2599e-05	-3.613	2.2473e-04	-0.941	4.5803e-01	pubmed
ENSMUSG00000107313	Gm43332	-4.816	5.2392e-03	-3.606	1	-3.532	1	pubmed
ENSMUSG00000107505	Stambp-ps1	-4.451	4.5648e-03	-2.945	1	-3.150	1	pubmed
ENSMUSG00000107516	Gm30784	-1.429	6.6303e-02	-3.525	3.8246e-06	-2.207	1.2820e-02	pubmed
ENSMUSG00000107605	Gm44117	-4.874	1.0140e-03	-3.934	5.3606e-02	-2.362	1	pubmed
ENSMUSG00000107655	Gm44220	-1.574	3.5904e-03	-0.675	4.8917e-01	-1.034	2.8828e-01	pubmed
ENSMUSG00000107962	Gm43980	-0.522	1.3959e-01	-0.626	5.3938e-01	-1.196	7.5638e-03	pubmed
ENSMUSG00000108207	1810059H22Rik	-3.156	4.5087e-04	-2.725	7.9525e-03	-1.555	1.5712e-01	pubmed
ENSMUSG00000108314	Prkcz2	-1.698	1.4560e-03	-1.852	2.9597e-03	-0.574	4.6427e-01	pubmed
ENSMUSG00000108436	Gm44851	-1.858	2.0218e-04	-1.411	1.5762e-01	-1.276	1.4462e-01	pubmed

		CLP1		CLP2		CLP3		
Ensg	Symbol	LFC	FDR	LFC	FDR	LFC	FDR	Link
ENSMUSG00000108621	Gm37494	-1.357	1.6498e-18	-1.388	1.5567e-05	-0.834	5.9017e-02	pubmed
ENSMUSG00000108659	Gm34121	-2.584	4.4502e-03	-2.771	3.6401e-02	-2.985	1.4997e-02	pubmed
ENSMUSG00000108822	Gm44787	-2.199	1.7379e-02	-1.985	4.7491e-03	-2.427	2.5337e-06	pubmed
ENSMUSG00000108825	Gm45838	-2.590	2.6885e-12	-2.033	3.0648e-04	-1.252	6.7655e-02	pubmed
ENSMUSG00000108961	Gm32540	-2.514	5.0973e-02	-3.578	1.0016e-03	-1.237	3.6037e-01	pubmed
ENSMUSG00000109015	Gm31024	-2.860	3.8823e-03	-0.340	1	-0.510	7.0791e-01	pubmed
ENSMUSG00000109115	Gm44669	-3.677	6.5345e-03	-5.212	7.6276e-04	-1.693	1.4628e-01	pubmed
ENSMUSG00000109482	Gm4756	-1.483	1.1990e-03	-1.873	8.1976e-04	-2.274	1.5449e-04	pubmed
ENSMUSG00000109587	Gm31105	-4.031	5.9466e-03	-3.236	1	-1.647	1	pubmed
ENSMUSG00000109628	BC024386	-1.626	4.5978e-18	-1.092	1.0543e-05	-1.222	7.0875e-05	pubmed
ENSMUSG00000109644	0610005C13Rik	-1.860	3.4332e-17	-2.257	5.2234e-16	-1.412	2.5956e-03	pubmed
ENSMUSG00000109674	Gm45470	-2.435	2.3462e-11	-2.747	1.0962e-03	-2.063	2.4870e-03	pubmed
ENSMUSG00000109702	Gm45674	-3.112	3.6353e-03	-4.300	1.5444e-02	-1.740	1.9778e-01	pubmed
ENSMUSG00000109771	Gm35315	-1.849	1.8974e-03	-2.259	1.1072e-03	-1.243	1.1579e-01	pubmed
ENSMUSG00000109783	Gm45338	-1.237	2.2012e-02	-1.078	9.4999e-03	-1.088	1.1434e-02	pubmed
ENSMUSG00000109836	Gm45819	-1.717	1.0480e-03	-1.986	4.2919e-06	-2.157	1.9822e-04	pubmed
ENSMUSG00000109904	Igip	-4.733	1.5465e-03	-3.109	1	-4.113	1	pubmed
ENSMUSG00000110185	Flt3l	-1.776	2.0341e-10	-2.185	9.9940e-07	-1.625	1.6740e-03	pubmed
ENSMUSG00000110206	Gm45716	-1.691	3.7735e-05	-1.604	2.7982e-02	-0.832	2.9460e-01	pubmed
ENSMUSG00000110344	Gm33543	-1.435	7.1658e-04	-0.760	1.4489e-01	-1.641	6.9260e-05	pubmed
ENSMUSG00000110353	Mup22	-0.918	4.9101e-04	-1.593	1.5511e-03	-1.456	7.9373e-04	pubmed
ENSMUSG00000110439	Gm10033	-3.290	8.5445e-03	0.442	7.7233e-01	0.079	1	pubmed
ENSMUSG00000110444	Gm45724	-0.912	6.0152e-06	-1.067	5.8780e-05	-0.752	2.6657e-02	pubmed
ENSMUSG00000110488	Gm45767	-6.343	2.6674e-01	-5.476	2.1169e-02	-5.898	5.3108e-03	pubmed
ENSMUSG00000110702	Gm31992	-2.944	1.6542e-03	-0.322	1	0.735	5.7892e-01	pubmed
ENSMUSG00000110781	Gm32281	-2.433	2.2927e-03	-1.917	1.6122e-02	-1.544	1.1459e-01	pubmed
ENSMUSG00000110827	Gm36251	-1.542	1.0860e-04	-0.932	1.9130e-01	-1.188	1.2128e-02	pubmed
ENSMUSG00000110996	Gm32511	-1.751	5.6198e-03	-0.713	2.9823e-01	-1.124	1.6451e-01	pubmed
ENSMUSG00000111429	Gm47465	-4.293	6.0556e-05	-2.766	3.2313e-02	-3.013	1	pubmed
ENSMUSG00000111771	Pbld1	-1.817	1.5382e-02	-1.393	1.2086e-02	-1.401	3.7879e-03	pubmed
ENSMUSG00000112129	Gm30262	-2.108	6.8697e-18	-2.500	6.2202e-08	-2.806	1.0702e-12	pubmed
ENSMUSG00000112249	Gm48226	-0.601	7.0634e-02	-1.100	2.3854e-06	-1.162	1.0463e-03	pubmed
ENSMUSG00000112302	C730027H18Rik	-1.462	6.3861e-03	-0.654	3.6663e-01	-0.468	5.7833e-01	pubmed
ENSMUSG00000112366	Gm48072	-1.087	7.5165e-05	-1.244	6.1131e-03	-0.824	8.4027e-02	pubmed
ENSMUSG00000112374	Gm35696	-5.564	8.5825e-04	-3.424	1	-4.403	1	pubmed
ENSMUSG00000112527	A730063M14Rik	-2.831	1.3296e-09	-2.314	2.6711e-04	-1.422	9.0981e-02	pubmed
ENSMUSG00000112639	Gm36172	-2.358	7.3512e-09	-2.031	5.3252e-03	-1.320	2.8142e-01	pubmed
ENSMUSG00000112758	Gm40770	-0.936	3.9649e-02	-1.575	7.6523e-04	-0.939	6.6990e-02	pubmed
ENSMUSG00000112796	Gm46430	-1.148	5.3412e-02	-1.068	9.7301e-02	-2.049	6.7546e-03	pubmed
ENSMUSG00000113204	B930059L03Rik	-1.083	6.4822e-07	-1.087	4.9643e-03	-0.341	4.2024e-01	pubmed
ENSMUSG00000113361	4930404H11Rik	-2.821	4.4769e-03	-1.077	5.0941e-01	-1.388	3.0436e-01	pubmed
ENSMUSG00000113630	2210039B01Rik	-2.542	9.1454e-03	-0.756	5.9109e-01	-1.188	1	pubmed
ENSMUSG00000113800	Gm6566	-2.693	1.9492e-03	-1.751	1.7479e-01	-1.455	1.9740e-01	pubmed
ENSMUSG00000113918	Gm49331	-2.279	2.5654e-03	-0.756	5.2863e-01	-1.018	1.4930e-01	pubmed
ENSMUSG00000114025	Gm32063	-2.068	5.3511e-05	-1.044	1.3216e-01	-1.261	1.7460e-01	pubmed
ENSMUSG00000114247	Gm31544	-3.751	1.7595e-18	-3.311	5.1016e-07	-2.427	1.5744e-01	pubmed
ENSMUSG00000114375	Gm9502	-0.379	5.1932e-01	-1.633	2.8425e-03	-0.742	2.8461e-01	pubmed
ENSMUSG00000114977	Gm49284	-2.808	5.7261e-02	-4.613	9.9543e-03	0.999	1	pubmed
ENSMUSG00000114995	Gm34934	-1.373	1.5649e-04	-1.235	7.8182e-02	-0.007	9.9392e-01	pubmed
ENSMUSG00000115100	Gm49201	-1.999	3.2788e-04	-1.881	1.5601e-02	-1.177	1.7268e-01	pubmed
ENSMUSG00000115124	Gm49417	-1.608	5.3043e-05	-1.078	3.7424e-02	-1.548	4.2431e-03	pubmed
ENSMUSG00000115186	Gm17753	-3.177	4.6969e-07	-2.611	8.5218e-03	-2.405	8.6372e-03	pubmed
ENSMUSG00000115867	Gm31583	-3.235	6.7190e-13	-3.561	1.8415e-13	-1.682	1.4729e-02	pubmed
ENSMUSG00000115919	Gm49431	-1.505	1.5250e-02	-0.573	5.6279e-01	-1.899	3.1563e-03	pubmed
ENSMUSG00000116130	C030006K11Rik	-1.708	5.3469e-03	-1.908	1.3479e-03	-2.124	1.4310e-03	pubmed
ENSMUSG00000116138	Gm49495	-0.868	6.5046e-06	-1.076	1.6809e-07	-0.542	1.4215e-02	pubmed
ENSMUSG00000116141	Gm49544	-5.731	1.9455e-04	-4.705	9.2849e-03	-4.266	1	pubmed
ENSMUSG00000116262	Cyp2d38-ps	-3.522	1.1421e-08	-2.915	5.8423e-05	-1.682	1.2937e-01	pubmed
ENSMUSG00000116354	5730414N17Rik	-3.003	4.6025e-08	-1.772	5.9566e-04	-1.287	2.2348e-02	pubmed
ENSMUSG00000116506	Gm49668	-1.457	1.3985e-04	-1.274	1.7377e-02	-0.841	3.6107e-02	pubmed
ENSMUSG00000116718	Gm21926	-1.052	5.3145e-04	-1.030	1.9615e-02	-1.345	1.1646e-03	pubmed
ENSMUSG00000116995	Gm22146	-1.475	6.0230e-05	-1.535	4.7472e-05	-0.860	9.7575e-02	pubmed
ENSMUSG00000117084	Gm31084	-1.369	9.4472e-02	-1.485	4.8516e-03	-1.946	2.2245e-02	pubmed
ENSMUSG00000117226	Ntn3	-1.132	3.5416e-01	-3.697	2.2521e-03	-1.583	1	pubmed
ENSMUSG00000117406	Gm19696	-1.189	3.5306e-03	-1.638	1.8813e-03	-1.829	3.4078e-03	pubmed
ENSMUSG00000117485		-4.302	4.4661e-17	-4.333	2.4206e-08	-3.705	2.4451e-06	pubmed
