# Supplementary figures and images for: Hepatic PPARα function and lipid metabolic pathways are dysregulated in polymicrobial sepsis
Source: EMBO Mol Med. 2020 Jan 9;12(2):e11319. doi: 10.15252/emmm.201911319 (PMC7005534; doi:10.15252/emmm.201911319)

Source data Figure 2

B

Upper panel

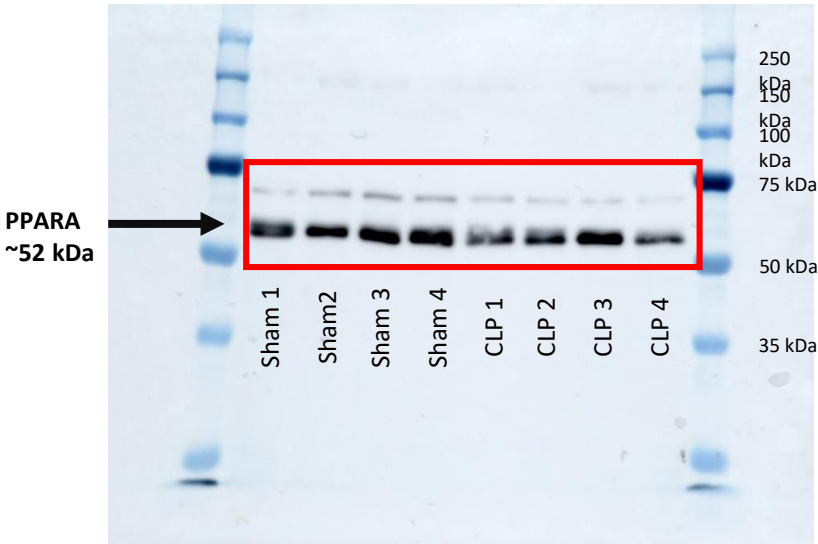

Lower panel

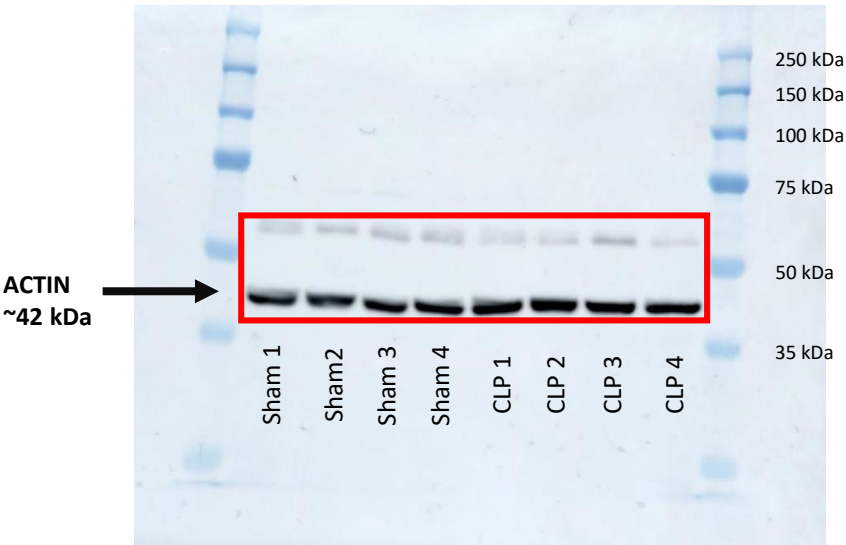

Supplement: Supplementary file 8 — Source Data for Figure 2 [file EMMM-12-e11319-s006.pdf]
