# Supplementary material for: Hepatic PPARα function and lipid metabolic pathways are dysregulated in polymicrobial sepsis
Source: EMBO Mol Med. 2020 Jan 9;12(2):e11319. doi: 10.15252/emmm.201911319 (PMC7005534; doi:10.15252/emmm.201911319)
Supplement: Supplementary file 7 — Review Process File [file EMMM-12-e11319-s008.pdf]

EMBO
Molecular Medicine

Hepatic PPARα function and lipid metabolic pathways are dysregulated in polymicrobial sepsis

Lise Van Wyngene, Tineke Vanderhaeghen, Steven Timmermans, Jolien Vandewalle, Kelly Van Looveren, Jolien Souffriau, Charlotte Wallaeys, Melanie Eggermont, Sam Ernst, Evelien Van Hamme, Amanda Gonçalves, Guy Eelen, Anneleen Remmerie, Charlotte L. Scott, Caroline Rombouts, Lynn Vanhaecke, Liesbet De Bus, Johan Decruyenaere, Peter Carmeliet, Claude Libert

Review timeline:

Submission date:	19 August 2019
Editorial Decision:	13 September 2019
Revision received:	4 November 2019
Editorial Decision:	19 November 2019
Revision received:	28 November 2019
Accepted:	29 November 2019

Editor: Lise Roth

Transaction Report:

(Note: With the exception of the correction of typographical or spelling errors that could be a source of ambiguity, letters and reports are not edited. The original formatting of letters and referee reports may not be reflected in this compilation.)

1st Editorial Decision 13 September 2019

Thank you for the submission of your manuscript to EMBO Molecular Medicine, and please accept my apologies for the delay in getting back to you, which is due to the fact that I sought external advice from a good expert in the field. Indeed, as you will see below, we have received contradictory feedbacks from the two reviewers who initially agreed to evaluate your manuscript. While referee #1 is overall positive, referee #2 has several major concerns that should be experimentally addressed, but also mentions the incremental advance and lack of novelty of the findings. Given these reports and as previously mentioned, I contacted a third expert, who stated:

"despite the partial known things about PPARa, the paper should get the chance for revision (and potentially be published). I actually think that the mechanism is important and these data are really beneficial to the field."

We would therefore like to invite revisions of the manuscript. Addressing the reviewers' concerns in full will be necessary for further considering the manuscript in our journal, and acceptance of the manuscript will entail a second round of review. EMBO Molecular Medicine encourages a single round of revision only and therefore, acceptance or rejection of the manuscript will depend on the completeness of your responses included in the next, final version of the manuscript. For this reason, and to save you from any frustrations in the end, I would strongly advise against returning an incomplete revision.

I look forward to receiving your revised manuscript.

***** Reviewer's comments *****

Referee #1 (Remarks for Author):

Sepsis constitutes a major and long-standing public heath problem which has not seen qualitative improvement in its management in decades, translating the insufficient understanding of its

© European Molecular Biology Organization

pathological bases. Most studies have focused on resistance and inflammatory mechanisms. Van Wyngene et al. explore the role of liver metabolism in the pathophysiology of sepsis, and demonstrate a central role for PPARa in this condition due to its inhibition during the course of this condition. This work is of interest both from a biological and biomedical perspective, as many therapeutic strategies focusing on the modulation of resistance and inflammatory mechanisms have so far produced disappointing results. The modulation of metabolism is a novel angle, which holds considerable promise.

This manuscript could potentially be made stronger if the authors characterise the effects of drug-modulation of PPARa (agonists and antagonists) on resistance and disease tolerance components of defense, during CLP by measuring bacterial loads (peripheral blood and target organs like lung, liver and kidney), as well as cytokine levels (e.g. TNF, IL-1b and IL-6) in addition to serologic markers of organ lesion. These data might help the authors speculate about the role of fatty acid metabolism during infection / sepsis in relation to its requirement for the infection and / or tissue damage control.

On a minor note, on page 11, it is stated that GW6471 is a PPARa agonist when it is in fact an antagonist, which should be corrected to avoid confusion.

Referee #2 (Comments on Novelty/Model System for Author):

During sepsis, hepatocyte-specific Ppara-deficiency is deleterious as it impairs the adaptive metabolic shift from glucose to FA utilization. Metabolic control by PPARα in hepatocytes therefore appears to play a key role in the host defense against infection. PPAR-β/δ agonists protect against multiple organ injury and dysfunction, and inflammation caused by endotoxic shock and improves survival in polymicrobial sepsis by a mechanism that may involve activation of Akt and inhibition of GSK-3β and NF-κB.

Referee #2 (Remarks for Author):

The authors present an investigation on hepatic PPARa function and lipid metabolic pathway are dysregulated in polymicrobial sepsis. The basic title reports well-established metabolic changes in experimental sepsis.

This work adds to a substantial body of literature suggesting a role for PPAR in organ protection during sepsis. Indeed, recent literature shows that during sepsis, Ppara-deficiency in hepatocytes is deleterious as it impairs the adaptive metabolic shift from glucose to FA utilization. Metabolic control by PPARα in hepatocytes plays a key role in the host defense against infection.

The authors have adopted a translational approach from lab to patient, which should be commended.

Main comments:
1. Fibrates have an extensive and pluripotent impact on many cell processes in sepsis. e.g. blockade of CXCR2 expression antagonizes the beneficial effects of fibrates in sepsis [EMBO Mol Med 2014]. The lack of hepatocyte-specific PPAR deficiency, which is clearly technically feasible, limits the robustness of the conclusions drawn [see: J Hepatol. 2019 May;70(5):963-973. doi: 10.1016/j.jhep.2018.12.037. Hepatic PPARα is critical in the metabolic adaptation to sepsis].
2. PPAR agonists confer organ protection; even delayed treatment with GW0742 consistently improves long-term survival in septic CD-1 mice by partially modulating the post-CLP systemic cytokine response and coagulation systems [Innate Immun. 2018 May;24(4):262-273. doi: 10.1177/1753425918771748]. These data suggest that liver dysfunction alone is not critical. The rationale for the duration of treatment for PPARa agonist pemafibrate is unclear.
3. A key issue in the murine data is controlling for core temperature. While starvation alone elicits less rapid changes in FFA excess, mouse undergoing CLP exhibited profound hypothermia is many cases [see:Fig 2D.] Examining the hepatic PPAR response to hypothermia alone is necessary. The hypothermic temperature evident in Fig 2D suggest imminent death.
4. What temperature were mice normally housed at? Was this thermoneutral [see:-Journal of Thermal Biology 37(8):654-685].

5. There are no clinical severity or activity data shown for septic mice. Since CLP/sepsis reduces physical activity, and muscle PPARδ expression positively correlates with activity, reduced locomotion as a driver for impaired PPAR expression should also be considered.
6. Glucose measurements in sham/septic mice would be instructive.
7. Immunoblot/PCR data on naive mice would be helpful, for comparison to sham/septic.
8. Seahorse data [Fig.2] shows one timepoint; no other data is provided in Suppl data. Please justify data presentation in this manner.

1st Revision - authors' response 4 November 2019

Referee #1

1. "Sepsis constitutes a major and long-standing public health problem which has not seen qualitative improvement in its management in decades, translating the insufficient understanding of its pathological bases. Most studies have focused on resistance and inflammatory mechanisms. Van Wyngene et al. explore the role of liver metabolism in the pathophysiology of sepsis, and demonstrate a central role for PPARα in this condition due to its inhibition during the course of this condition. This work is of interest both from a biological and biomedical perspective, as many therapeutic strategies focusing on the modulation of resistance and inflammatory mechanisms have so far produced disappointing results. The modulation of metabolism is a novel angle which holds considerable promise."

We thank the referee for these kind words.

2. "This manuscript could potentially be made stronger if the authors characterize the effects of drug-modulation of PPARα (agonists and antagonists) on resistance and disease tolerance components of defense, during CLP by measuring bacterial loads (peripheral blood and target organs like lung, liver and kidney), as well as cytokine levels (e.g. TNF, IL-1b and IL-6) in addition to serologic markers of organ lesion. These data might help the authors speculate about the role of fatty acid metabolism during infection / sepsis in relation to its requirement for the infection and / or tissue damage control."

*We agree with this remark, therefore, bacterial loads were analyzed via plating of liver, lung and kidney homogenates on LB agar plates and blood on TSA agar plates to determine the amount of colony forming units (CFUs) in sham and septic mice, with or without agonist (pemafibrate) or antagonist (GW6471) treatment. We observed a decrease in the systemic, hepatic, renal and pulmonary bacterial load in mice treated with pemafibrate 24h post-CLP (**Rebuttal figure 1A-D**). In contrast, treatment with the PPARa antagonist GW6471 caused an increase in bacterial load in these organs (an increase was observed in systemic circulation, but was not significant) (**Rebuttal figure 1E-H**). These data are in line with the study of Tancevski et al. that has previously demonstrated similar observations in the E. Coli infection model with fenofibrate treatment (Tancevski et al, 2014). There, the decreased bacterial load was attributed to enhance neutrophil recruitment and more efficient early clearance of bacteria through fibrate-induced stabilization of CXCR2 expression. Our results indicate that the protective mechanism of pemafibrate is partly caused by the decrease in bacterial load in circulation and organs. Indeed, potentially, the preservation of metabolic integrity could aid in the control of infection, which emphasizes the crucial link between metabolism and the immune system.*

*Because of the relevance of these data, **figure 6** in the manuscript was adapted to include the results of the systemic bacterial load during pemafibrate treatment (**Figure 6E**). In addition, this section was added to the results: "*Moreover, fibrates have been demonstrated to reduce systemic and organ bacterial loads through increased recruitment of neutrophils to the site of infection. In accordance, mice that were treated with pemafibrate showed lower systemic, hepatic, renal, and pulmonary bacterial levels 24h after sepsis-initiation (**Fig 6E, Fig EV3F-G**)."

*Data of bacterial loads in liver, kidney and lung can be found in EV figure 3 (**Figure EV3 F-H**). Results of the bacterial load during sepsis with GW6471 have been added to the supplementals as Figure EV4C-F. The text in the results was adapted to this: "*Mice that were treated with GW6471 displayed a significant increase in mortality during CLP from ~44% to ~88%, and worsened disease and metabolic parameters, showing that inhibition of PPARα function is detrimental for survival during sepsis (**Fig 6H, Fig EV4**)."

In addition, this section of text was added to the discussion to include the potential link between preservation of metabolic health to immunity as a protective mechanism of

*pemafibrate:"*Comparable to fenofibrate, pemafibrate reduced bacterial loads in circulation and target organs, indicating that through modulation of PPARa expression and metabolic pathways, pemafibrate may assist in the control of tissue infection and damage. Indeed, several studies have shown that metabolism is clearly linked to immunity and that the metabolic status of inflammatory cells changes their response to infection."

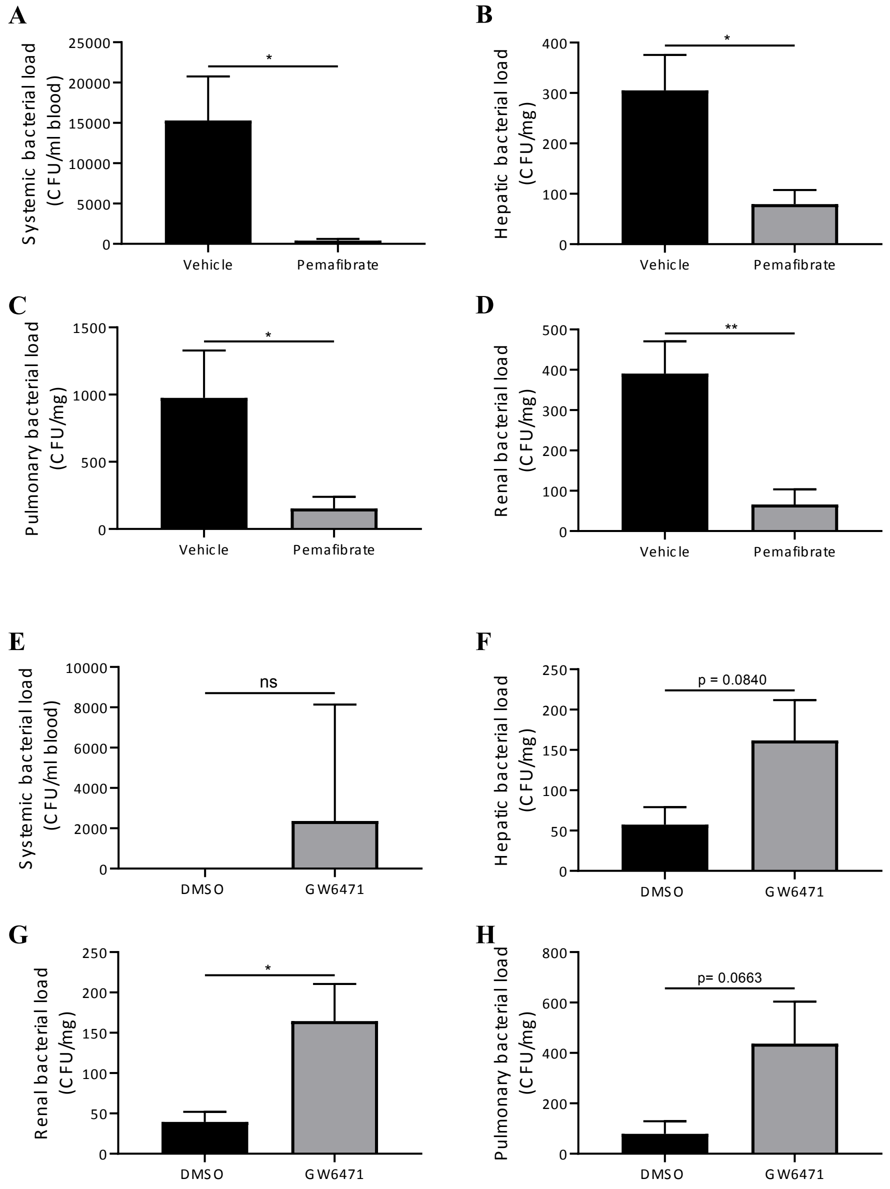

Rebuttal figure 1. PPARa agonism and antagonism change systemic and organ bacterial loads after sepsis. A-D*:* Mice were pre-treated with pemafibrate (1mg/kg) or vehicle (0,9% NaCl) for 1 week before being subjected to CLP, 24h post-CLP blood and organs were isolated (n = 5-6/group). Bacterial load was determined in **(A)** blood and **(B)** liver, **(C)** kidney and **(D)** lung tissue homogenates. Values are shown as mean CFU/mg tissue ± SEM, .p-values were calculated using two-way Student's t-tests. One experiment. **E-H**: Mice were injected with the PPARα antagonist GW6471 (10µg/g) or vehicle (DMSO) 3h pre- and 3h post-CLP, 24h post CLP blood and organs were isolated (n = 5-6/group). Bacterial load was determined in **(A)** blood and **(B)** liver, **(C)** kidney and **(D)** lung tissue homogenates. Values are shown as mean CFU/mg tissue ± SEM. p-values were calculated using two-way Student's t-tests. One experiment. **P < 0.01; *P < 0.05.

To answer the second part of the reviewers remark, namely the effect of PPARa agonism or antagonism on cytokine levels during sepsis, we determined IL-6, IFN-g, TNF and IL-1b plasma

© European Molecular Biology Organization

levels via Bioplex (on plasma of sham and CLP mice treated with pemafibrate or GW6471, 24h post sepsis initiation). We observed comparable plasma IL-6 levels when blood was analyzed with Bioplex as we have seen with the IL-6 ELISA previously (see ***Figure 6F****). Levels of IFNg, TNF and IL-1b did not differ between sham or CLP mice, nor did treatment with pemafibrate or GW6471 influence these cytokine levels (****Rebuttal figure 2****). Studies have shown that IL-6 is the one of the most predominant cytokines during CLP, and that IL-6 serves as a marker for disease severity in sepsis (Remick et al, 2005). In contract, another study demonstrated that TNF and IL-1b blood levels peak very early after the induction of sepsis and decrease afterwards, which could explain why we did not observe an increase in TNF and IL-1b levels 24h after sepsis (Villa et al, 1995). Moreover, a recent study by our research group has proved that TNF plays only a minor role in sepsis, as TNF receptor knock-out mice were not protected during CLP (unpublished data). In addition, Orman et al. showed that IFNg levels did not increase in the blood 16h after CLP (Orman et al, 2011). Since TNF, IL-1B and IFNg do not seem to be essential in the disease progression of sepsis, and IL-6 results from the Bioplex experiment were similar to that of the IL-6 ELISA, we have decided to not include these results in the manuscript.*

© European Molecular Biology Organization

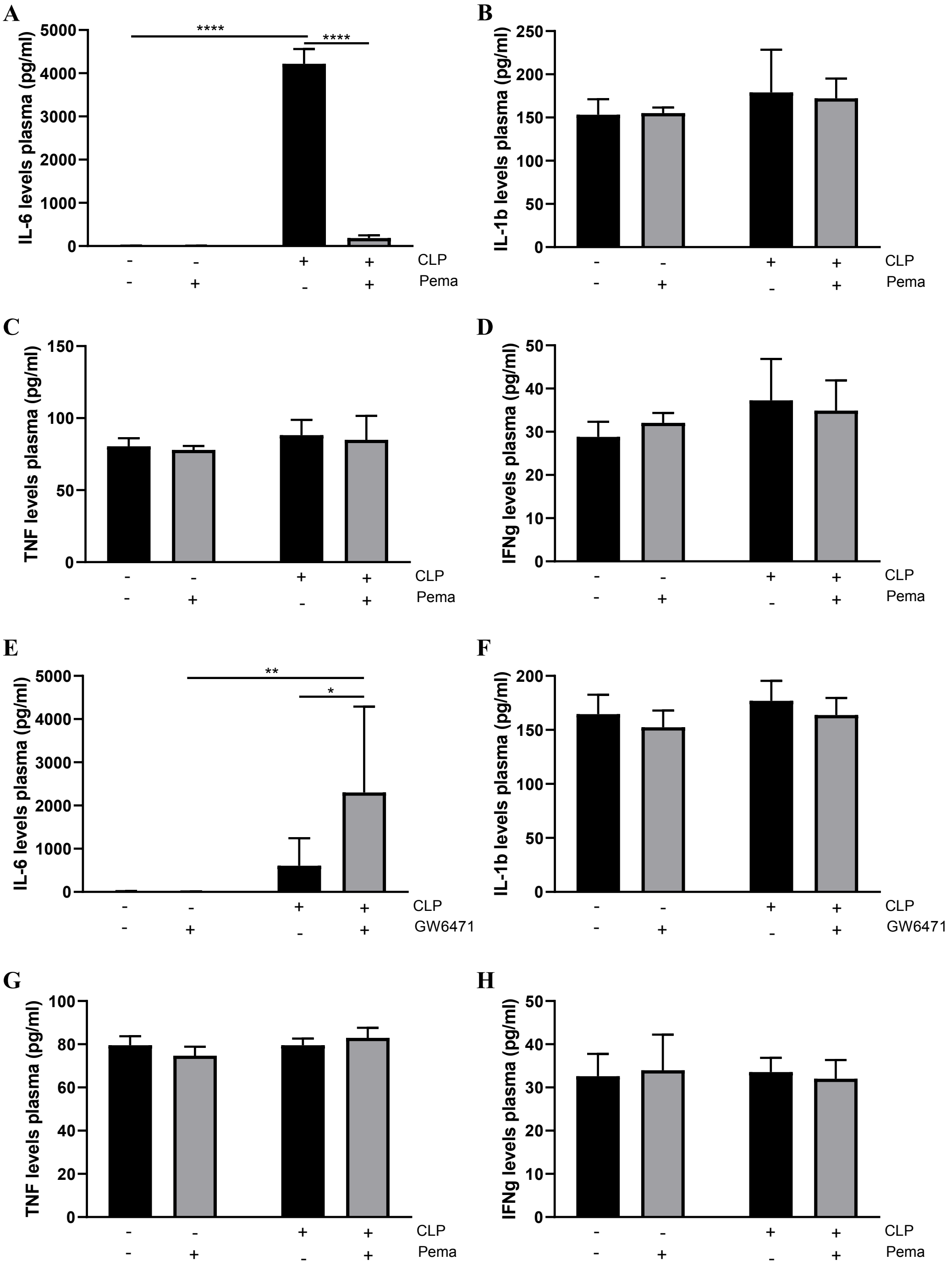

Rebuttal figure 2. PPARa agonism and antagonism only alters IL-6 plasma levels during sepsis. A-*D*: Mice were pre-treated with pemafibrate (1mg/kg) or vehicle (0,9% NaCl) for 1 week before being subjected to CLP, 24h post-CLP blood was collected (n = 5-6/group). Plasma levels of **(A)** IL-6, **(B)** IL-1β, **(C)** TNF and **(D)** IFNγ were determined via Bioplex. Values are shown as mean ± SEM. p-values were calculated using P-values were calculated with 2-way ANOVA tests. One experiment. **E-H**: Mice were injected with the PPARα antagonist GW6471 (10µg/g) or vehicle (DMSO) 3h pre- and 3h post-CLP, 24h post CLP blood was collected (n = 5-6/group). Plasma levels of **(A)** IL-6, **(B)** IL-1β, **(C)** TNF and **(D)** IFNγ were determined via Bioplex. Values are shown as mean ± SEM. p-values were calculated using P-values were calculated with 2-way ANOVA tests. One experiment. ****P < 0.0001; ***P < 0.001; **P < 0.01; *P < 0.05

3. “On a minor note, on page 11, it is stated that GW6471 is a PPARα agonist when it is in fact an antagonist, which should be corrected to avoid confusion.”

© European Molecular Biology Organization

Indeed, to avoid confusion, we have corrected the word agonist to antagonist in the revised manuscript.

Referee #2

1. "During sepsis, hepatocyte-specific Ppara-deficiency is deleterious as it impairs the adaptive metabolic shift from glucose to FA utilization. Metabolic control by PPARα in hepatocytes therefore appears to play a key role in the host defense against infection. PPAR-β/δ agonists protect against multiple organ injury and dysfunction, and inflammation caused by endotoxic shock and improves survival in polymicrobial sepsis by a mechanism that may involve activation of Akt and inhibition of GSK-3β and NF-κB."

Indeed, we agree with the referee on the statement that fibrates have a wide variety of physiological effects. We will address this notion into more detail in point 3 & 4.

2. "The authors present an investigation on hepatic PPARa function and lipid metabolic pathway are dysregulated in polymicrobial sepsis. The basic title reports well-established metabolic changes in experimental sepsis.
   This work adds to a substantial body of literature suggesting a role for PPAR in organ protection during sepsis. Indeed, recent literature shows that during sepsis, Ppara-deficiency in hepatocytes is deleterious as it impairs the adaptive metabolic shift from glucose to FA utilization. Metabolic control by PPARα in hepatocytes plays a key role in the host defense against infection.
   The authors have adopted a translational approach from lab to patient, which should be commended."

We thank the referee for this kind remark. Indeed, several studies have investigated the role of PPARα during sepsis, with most research focusing on the effect of PPARs and their agonist on the inflammatory component of sepsis. We are aware of the recent publication by Paumelle et al., a study that shows as one of the first that metabolic derangements can have major effects during sepsis and that PPARα in the liver plays a pivotal role in these adaptations. At the time of the release of this story, we were finishing up our study for publication. Our stories have a similar storyline and several common aspects, however, we believe we have added a substantial amount of new findings concerning the role of hepatic PPARα during sepsis, sepsis patient data, and new insight into the therapeutic potential of PPARα agonists in sepsis.

3. "Fibrates have an extensive and pluripotent impact on many cell processes in sepsis. e.g. blockade of CXCR2 expression antagonizes the beneficial effects of fibrates in sepsis [EMBO Mol Med 2014]. The lack of hepatocyte-specific PPAR deficiency, which is clearly technically feasible, limits the robustness of the conclusions drawn [see: J Hepatol. 2019 May;70(5):963-973. doi: 10.1016/j.jhep.2018.12.037. Hepatic PPARα is critical in the metabolic adaptation to sepsis]."
4. "PPAR agonists confer organ protection; even delayed treatment with GW0742 consistently improves long-term survival in septic CD-1 mice by partially modulating the post-CLP systemic cytokine response and coagulation systems [Innate Immun. 2018 May;24(4):262-273. doi: 10.1177/1753425918771748]. These data suggest that liver dysfunction alone is not critical. The rationale for the duration of treatment for PPARa agonist pemafibrate is unclear."

We have attempted to establish our own PPARα fl/fl-Alb-Cre mouse breeding in house in the past, however, we have experienced breeding difficulties and were therefore unsuccessful. Even though, we strongly agree with the reviewer and have chosen an alternative approach to strengthen the knowledge on the effect of pemafibrate on the liver for survival during sepsis. We have acquired PPARα fl/fl mice from a collaborator and used an adenoviral vector expressing the recombinase Cre to target the liver via intravenous (iv) injections. We have several years of experience with this technique and achieve a 50-90% knock-down of the target-gene in the liver, without obvious off-site effects of the viral vector.

Control (GFP tagged) and Cre-expressing adenoviruses (type 5) were purchased from VectorBuilder (https://en.vectorbuilder.com/). It is known that adenoviruses that are injected intravenously (iv) are predominantly sequestered by the liver (Shayakhmetov et al, 2004). During experiments, mice were injected with $2x10^{10}$ viral particles intravenously.

© European Molecular Biology Organization

*PPARα fl/fl mice were assigned to a pemafibrate-treated group or a control-treated group for 1 week and received an iv injection with either the adenoviral vector expressing Cre or a control empty viral vector 72h before sepsis (CLP) induction. Survival of all groups was followed over-time (**Rebuttal figure 3C**). In parallel, mice injected with the GFP control virus were sacrificed 72h post virus-injection to investigate the transduction efficiency of the hepatocytes. Liver was isolated and paraffin sections were analyzed via confocal microscopy. The data indicate that 70-80% of the liver was successfully transduced with the GFP-expressing virus 72h after iv injection (**Rebuttal figure 3A**). In addition to lethality, PPARa expression was analyzed in livers of PPARa fl/fl 72h post virus injection to confirm knock-down of PPARa, indeed, PPARa expression levels in the liver were reduced by about 3 fold (**Rebuttal figure 3B**).*

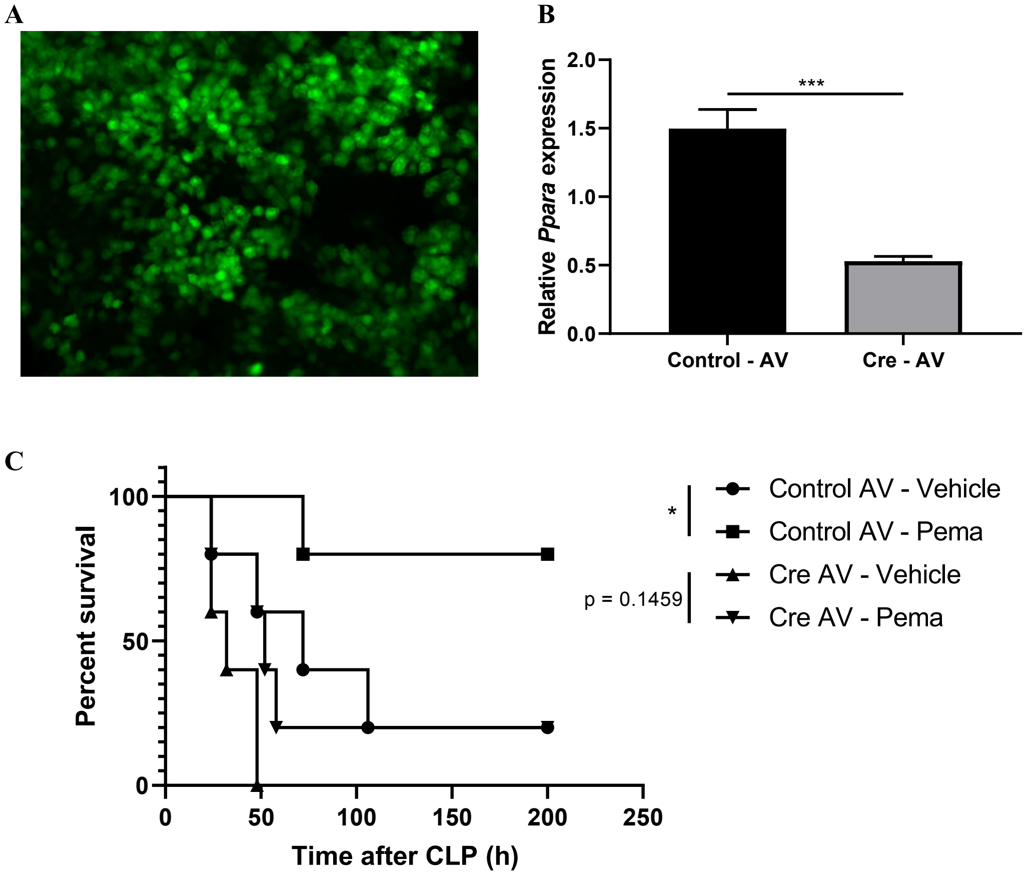

Rebuttal figure 3. Absence of hepatic PPARa reduces the protective effect of Pemafibrate during sepsis. *PPARa floxed mice were pre-treated with pemafibrate (1mg/kg) or vehicle (0,9% NaCl) for 1 week before being subjected to CLP. Additionally, mice were injected intravenously with $2x10^{10}$ viral particles of the control (GFP) adrenal vector (Control AV) or the Cre-expressing adrenal vector (Cre AV) 72h before CLP. (A) Liver from Control AV injected PPARa fl/fl mice was isolated, after which paraffin-embedded sections were analyzed for transduction efficiency through visualization of the GFP signal via fluorescent microscopy (n = 3). (B) Livers were isolated from Control and Cre AV injected PPARa fl/fl mice and Ppara gene expression was determined via qPCR. Gene expression values are shown as mean ± SEM, P-values were calculated via the two-way Student's t-tests. One experiment (n = 3/group). (C) During a survival experiment, mortality was monitored during 9 days, after which no further deaths occurred. Survival curves were analyzed via a Chi-square tests. One experiment (n = 5/group).* ***P < 0.001; **P < 0.01; *P < 0.05

*We agree with the referee on the notion that fibrates have many mechanism of action and liver is not the sole organ affected during sepsis. The results of the experiment with PPARα fl/fl mice and the adenoviral vectors partly confirm this hypothesis, as pemafibrate treatment shows a trend towards protection in mice with PPARα fl/fl knock-down in the liver (1/5 survivors in the pema group versus 0/5 survivors in the vehicle group) (**Rebuttal figure 3C**). However, when PPARα is present in the liver, pemafibrate is able to confer its full protection (4/5 survivors in the pemafibrate group versus 1/5 survivors in the vehicle group), suggesting that for this agonist, the liver is one of the main organs of action and that improvement of liver dysfunction can have beneficial effects during sepsis.*

© European Molecular Biology Organization

These results are preliminary and further investigation into the liver-specific action of pemafibrate is warranted with hepatocyte-specific knock-out studies as a necessity. We realize the preliminary status of these results (low n-values, partly removal of PPARa in the liver,…) , therefore, we have not yet added this data to the manuscript. ***We would like to ask the opinion of the editor on this topic: is it acceptable to already publish these preliminary results?***

*We acknowledge the importance studies that address the role of the liver in the protective mechanism of pemafibrate during sepsis, accordingly, we have added this section to the discussion: "*However, as fibrates have many mechanisms of action and the liver is not the only organ affected during sepsis, it is essential to study the role of the liver in the protection of pemafibrate during sepsis, for example via the use of PPARα hepatocyte-specific knock-out animal models.*"*
Concerning the duration of pemafibrate treatment. Pemafibrate has, up till now, only been tested in a chronic set-up during metabolic disorders such as nonalcoholic steatohepatitis (NASH) (Honda et al, 2017), in which a treatment of 4 weeks was used with success. Since sepsis is a very acute disorder, we wanted to reduce the treatment time and started our initial experiments with the 1 week set-up (on alternating days). At a later stage, when we were investigating a more therapeutic window, shorter treatment times were assessed.

5. "A key issue in the murine data is controlling for core temperature. While starvation alone elicits less rapid changes in FFA excess, mouse undergoing CLP exhibited profound hypothermia is many cases [see:Fig 2D.] Examining the hepatic PPAR response to hypothermia alone is necessary. The hypothermic temperature evident in Fig 2D suggest imminent death."

© European Molecular Biology Organization

6. *We thank the referee for this valid suggestion. Therefore, to investigate the effect of hypothermia on hepatic PPARα expression, we chose to use AMP injections to induce a pharmacological state of hypothermia (torpor). This method has been validated (Dugbartey et al, 2015) and has been successfully implemented in the lab before. Mice were injected Intraperitoneal with 0.15 mg/g of 5'AMP or saline as a control, and blood and organs were isolated 2h after injection (a timepoint at which body temperatures have decreased significantly and reflect the body temperature of CLP mice at the time of isolation, a graph visualizing the body temperatures can be found in* ***Rebuttal figure 4A****). FFA levels and hepatic Ppara gene expression did not differ in AMP injected mice compared to PBS controls. These data indicate that decreased body temperature in se does not alter Ppara expression levels and that an additional trigger (most probably acute inflammation) is required (****Rebuttal figure 5B****). These results were added to the* ***Appendix in Figure S2*** *and this text: "*PPAR expression has been linked to body temperature, therefore, to exclude an effect of body temperature on liver PPARa expression levels, mice were injected with AMP to mimic hypothermia. Differences in PPARa expression levels at lower body temperatures were not observed (**Appendix Figure S2**).*" was added to the results to account for the potential role of core temperature changes in the regulation of PPARa expression levels.*

Rebuttal figure 4. Hypothermia does not influence hepatic PPARa expression levels. *Hypothermia in mice was induced via the injection of 0,15mg/g of 5'AMP, and saline as a control,*

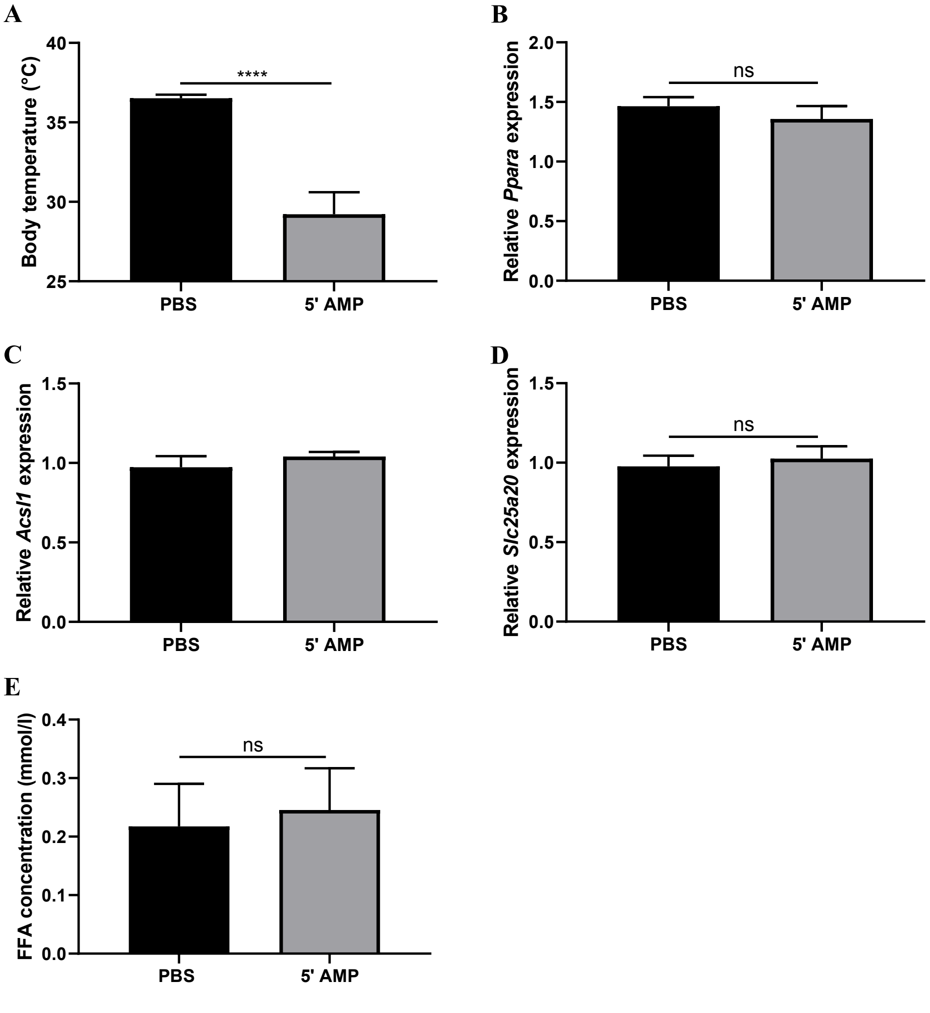

intraperitoneally. 2h after injection, blood and liver was collected (n = 6/group, one experiment). (A) Body temperature of mice 2h after injection. Values are shown as mean ± SEM, P-values were calculated with 2-way Student's t-tests. *(B-D)* mRNA from liver was prepared and gene expression levels of *Ppara, Acsl1 and Slc25a20* were analyzed via qPCR. Gene expression values are shown as mean ± SEM, P-values were calculated via 2-way Student's T-tests. (E) Plasma was isolated and free fatty acid (FFA) levels was determined as described in the method section. Values are shown as

mean ± SEM, P-values were calculated with 2-way Student's t-tests. ****P < 0.0001; ***P < 0.001; **P < 0.01; *P < 0.05

7. "What temperature were mice normally housed at? Was this thermoneutral [see:-Journal of Thermal Biology 37(8):654-685]."

Mice were housed at our animal facility at a constant temperature of 21°C. We acknowledge that housing at the mouse-thermoneutral temperature of 30°C would be ideal and would better reflect the metabolic homeostatic status, however, this was not implemented due to technical infeasibility.

8. "There are no clinical severity or activity data shown for septic mice. Since CLP/sepsis reduces physical activity, and muscle PPARδ expression positively correlates with activity, reduced locomotion as a driver for impaired PPAR expression should also be considered."

*We thank the referee for this interesting remark and have adopted the Modified Mouse Clinical Assessment Score for Sepsis (M-CASS) as a measure for disease severity (Mai et al, 2018). This scoring system accounts for several visual disease markers, including activity, posture and behavior. Results of the M-CASS screening in sham (scores of 0, data not shown) or septic mice with or without pemafibrate/GW6471 showed that pemafibrate reduced the total M-CASS score, while GW6471 treatment increased the total M-CASS score (although not significantly) (**Rebuttal figure 5**). Data of M-CASS results were added to **Figure EV2B** (Pemafibrate) and **Figure EV3B** (GW6471).*

Rebuttal figure 5. Modified Mouse Clinical Assessment Score for Sepsis (M-CASS) is influenced by PPARa (A) agonist (pemafibrate) or (B) antagonist (GW6471) treatment. (A) Mice were pre-treated with pemafibrate (1mg/kg) or vehicle (0,9% NaCl) for 1 week before being subjected to CLP, 24h post-CLP M-CASS scores were determined. (n = 5-6/group). Values are shown as mean ± SEM, .p-values were calculated using P-values were calculated with a 2-way Student's t-test. One experiment. **(B)** Mice were injected with the PPARα antagonist GW6471 (10µg/g) or vehicle (DMSO) 3h pre- and 3h post-CLP, 24h post-CLP M-CASS scores were determined. (n = 5-6/group). Values are shown as mean ± SEM, .p-values were calculated using P-values were calculated with a

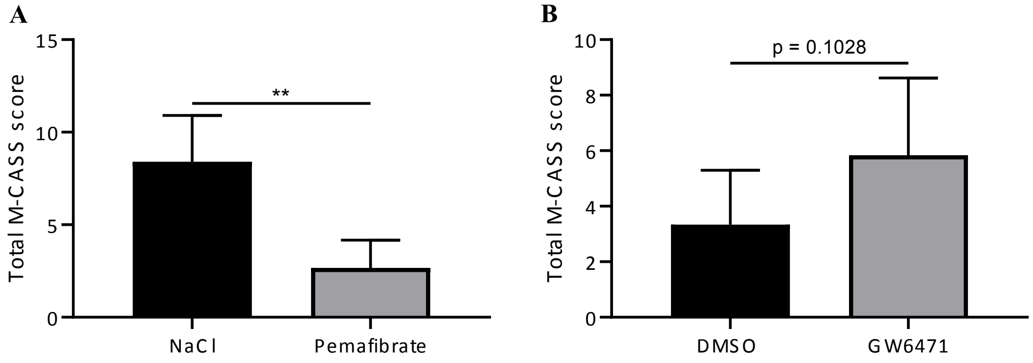

2-way Student's t-test. One experiment. **P < 0.01; *P < 0.05

*In addition, to account for the possibility that reduced locomotion may add to the reduced Ppara gene expression after sepsis, we have added this section of text to the discussion: "*PPAR expression has been demonstrated to be influenced by physical activity and cardiorespiratory fitness, therefore, reduced locomotion should also be considered as a potential cause of decreased PPARa expression in the liver. Indeed, septic mice showed a reduction in fitness and activity, demonstrated by the increase in M-CASS score, while treatment with pemafibrate improved the overall health of septic mice and thus improved locomotion and activity(Luquet et al, 2003; Queiroga et al, 2015)*"*

9. "Glucose measurements in sham/septic mice would be instructive."

*Indeed, changes in glucose metabolism during sepsis are of major interest. We have a substantial amount of experience with glucose measurements in sham/septic mice and we are currently working on a manuscript in which we address the effects of sepsis on glucose metabolism. Therefore, we have not included these results in this current paper, however, to address the concern of the reviewer, we have added the **Rebuttal figure 6** below. Glucose levels were determined in the blood via a handheld glucometer (FreeStyle).*

Glucose levels were measured in sham and septic mice, either supplemented with GW6471 or pemafibrate according to previously described methods, 24h after sepsis-initiation. 24h after sepsis, blood glucose levels were significantly down (hypoglycemia). Pemafibrate treated mice had higher

© European Molecular Biology Organization

*glucose levels compared to vehicle controls, reflecting the overall increased fitness and health after pemafibrate treatment **(Rebuttal Figure 6A)**, however, antagonist treatments had no effect on the glucose levels after sepsis (**rebuttal figure 6B**).*

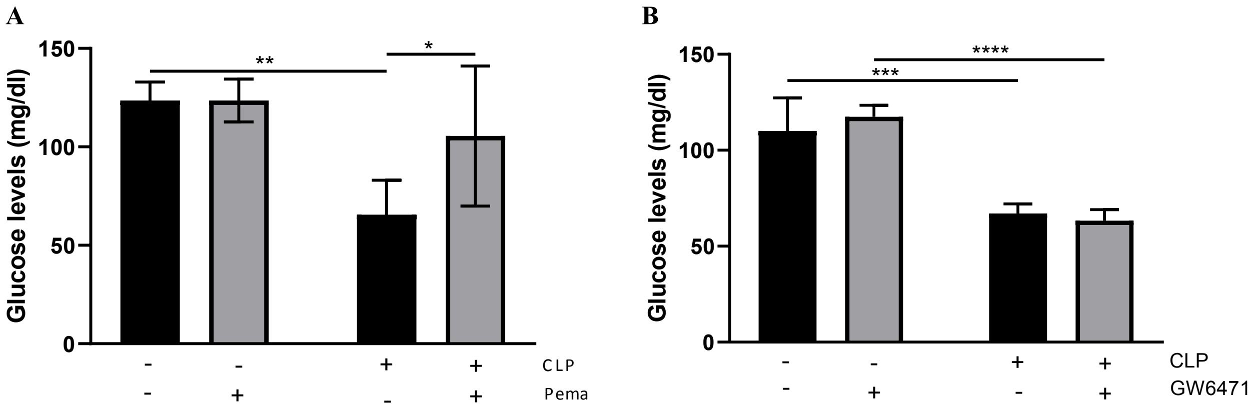

Rebuttal figure 6. Glucose levels 24h post sepsis in (A) pemafibrate and (B) GW6471 treated mice. ***(A)*** Mice were pre-treated with pemafibrate (1mg/kg) or vehicle (0,9% NaCl) for 1 week before being subjected to CLP, 24h post-CLP blood glucose levels were collected via the tail vain with a handheld glucometer. (n = 5-6/group). Values are shown as mean ± SEM. p-values were calculated using a 2-way Student's t-test. One experiment. **(B)** Mice were injected with the PPARα antagonist GW6471 (10µg/g) or vehicle (DMSO) 3h pre- and 3h post-CLP, 24h post-CLP blood glucose levels were collected via the tail vain with a handheld glucometer. (n = 5-6/group). Values are shown as mean ± SEM. p-values were calculated using a 2-way Student's t-test. One experiment. ****P < 0.0001; ***P < 0.001; **P < 0.01; *P < 0.05.

10. "Immunoblot/PCR data on naive mice would be helpful, for comparison to sham/septic."

*To address the concern of the referee, a western blot and qPCR analysis were conducted that included naive mice to compare this condition to sham and septic mice. Results are shown in **rebuttal figure 7.** Sham operations had no significant effect on hepatic PPARa expression and protein levels 24h post-surgery, compared to naive control mice. A slight trend towards increased PPARa levels could be observed in sham mice compared to naive mice, which is not unexpected as sham mice tend to eat less than naive mice. This decrease in food intake may stimulate PPARa and therefore increase ppara levels slightly.*

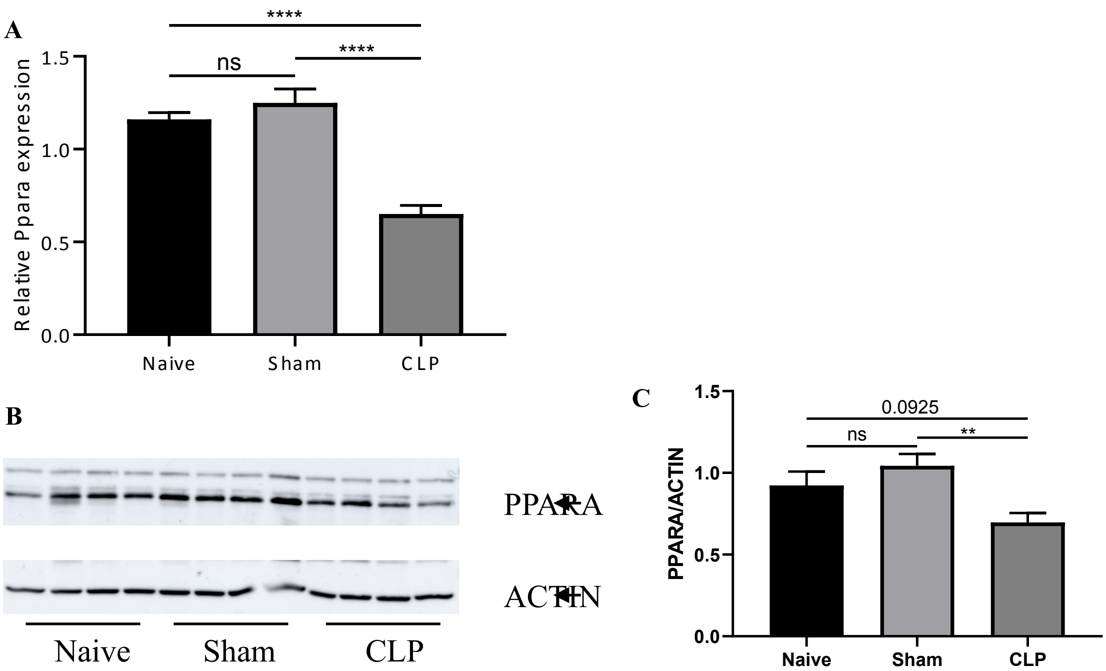

Rebuttal figure 7. Hepatic PPARa expression and protein levels of naive, sham and CLP mice. Mice were subjected to sham/CLP surgery or no surgery, after 24h liver was isolated (n = 4/group, pooled data of 2 independent experiments). **(A)** mRNA was isolated and hepatic Ppara expression was determined via qPCR. Gene expression values are shown as mean ± SEM, P-values were calculated via 2-way Student's T-tests. **(B-C)** PPARα protein levels were analyzed in livers by Western Blot using actin as a loading control. Values are presented as mean ± SEM and P-value was calculated via two-way Student's t-test (n = 4/group). ****P < 0.0001; ***P < 0.001; **P < 0.01; *P < 0.05.

11. "Seahorse data [Fig.2] shows one timepoint; no other data is provided in Suppl data. Please justify data presentation in this manner."

*Figure 2F shows the OCR of every timepoint (measurements every 6 minutes), which demonstrates that the values stay stable during the entire experiment. We opted to visualize the last timepoint in Figure 2G to more clearly show the differences between the different treatment groups. To be complete, a comparable representation of every timepoint has been added to the **appendix** In **figure S3.***

For the editor: while this manuscript was under revision, we had already started a Seahorse experiment to address the effect of pemafibrate treatment on activity of the β-oxidation pathway. We have observed that pemafibrate is able to recover some of the activity of this pathways as oxygen consumption rates (OCR) were higher in the pemafibrate treated group compared to vehicle treated control CLP mice (when palmitic acid was added). If the editor agrees, we would like to add this result to Figure 5 (part I) of the manuscript because we believe this data adds to the understanding of the mechanism of pemafibrate's protective action during sepsis. Similar to the Seahorse data in Figure 2 of the manuscript, we have added the results of every measurement timepoint to the Appendix as Figure S5.

References

Dugbartey GJ, Bouma HR, Strijkstra AM, Boerema AS, Henning RH (2015) Induction of a Torpor-Like State by 5'-AMP Does Not Depend on H2S Production. PLoS One 10**:** e0136113

Honda Y, Kessoku T, Ogawa Y, Tomeno W, Imajo K, Fujita K, Yoneda M, Takizawa T, Saito S, Nagashima Y et al (2017) Pemafibrate, a novel selective peroxisome proliferator-activated receptor alpha modulator, improves the pathogenesis in a rodent model of nonalcoholic steatohepatitis. Sci Rep 7**:** 42477

Luquet S, Lopez-Soriano J, Holst D, Fredenrich A, Melki J, Rassoulzadegan M, Grimaldi PA (2003) Peroxisome proliferator-activated receptor delta controls muscle development and oxidative capability. FASEB J 17**:** 2299-2301

Mai SHC, Sharma N, Kwong AC, Dwivedi DJ, Khan M, Grin PM, Fox-Robichaud AE, Liaw PC (2018) Body temperature and mouse scoring systems as surrogate markers of death in cecal ligation and puncture sepsis. Intensive Care Med Exp 6**:** 20

Orman MA, Nguyen TT, Ierapetritou MG, Berthiaume F, Androulakis IP (2011) Comparison of the cytokine and chemokine dynamics of the early inflammatory response in models of burn injury and infection. Cytokine 55**:** 362-371

Queiroga MR, Barbieri RA, Ferreira SA, Luchessi AD, Hirata RD, Hirata MH, Kokubun E (2015) Influence of cardiorespiratory fitness on PPARG mRNA expression using monozygotic twin case control. J Diabetes Res 2015**:** 538732

Remick DG, Bolgos G, Copeland S, Siddiqui J (2005) Role of interleukin-6 in mortality from and physiologic response to sepsis. Infect Immun 73**:** 2751-2757

Shayakhmetov DM, Li ZY, Ni S, Lieber A (2004) Analysis of adenovirus sequestration in the liver, transduction of hepatic cells, and innate toxicity after injection of fiber-modified vectors. J Virol 78**:** 5368-5381

Tancevski I, Nairz M, Duwensee K, Auer K, Schroll A, Heim C, Feistritzer C, Hoefer J, Gerner RR, Moschen AR et al (2014) Fibrates ameliorate the course of bacterial sepsis by promoting neutrophil recruitment via CXCR2. EMBO Mol Med 6**:** 810-820

Villa P, Sartor G, Angelini M, Sironi M, Conni M, Gnocchi P, Isetta AM, Grau G, Buurman W, van Tits LJ et al (1995) Pattern of cytokines and pharmacomodulation in sepsis induced by cecal ligation and puncture compared with that induced by endotoxin. Clin Diagn Lab Immunol 2**:** 549-553

2nd Editorial Decision 19 November 2019

Thank you for the submission of your revised manuscript to EMBO Molecular Medicine. We have received the referees' reports, and as you will see they are now supportive of publication of your study pending minor revisions. I am therefore pleased to inform you that we will be able to accept your manuscript once the following points will be addressed:

1) Referee's comments:
At this stage, we will not ask you to add data from separate models. However, please carefully address the other concerns from referee #2 in the manuscript and in a point-by-point response. If you have data at hand, we would be happy for you to include it, however we will not ask you to provide any additional experiments at this stage.

I look forward to reading a new revised version of your manuscript as soon as possible.

***** Reviewer's comments *****

Referee #1 (Remarks for Author):

The authors have addressed all my suggestions and concerns. As a result, the manuscript is now considerably stronger and can be published as it stands.

Referee #2 (Remarks for Author):

The authors have extensively addressed most critiques. Editorial discretion dictates whether additional data from separate models should be added to the manuscript. My chief outstanding concerns are:

1. Data should be presented as individual subjects, particularly given low n numbers.
2. Hypothermia in mice was induced via the injection of 0,15mg/g of 5'AMP, and saline as a control, intraperitoneally. 2h after injection, blood and liver was collected(n = 6/group, one experiment). Although body temperature of mice declined 2h after injection, it is extremely unlikely that gene transcription altered so rapidly [not least because of reduced metabolism!]. Therefore, mRNA/gene expression levels of Ppara, Acsl1 and Slc25a20 analyzed via qPCR seems far too early to draw the conclusion that there was no transcriptomic impact of hypothermia.
3. Thermoneutral temperature was clearly not considered in the design, and is an important confounder. At the very least, this limitation needs to be considered in Discussion.

2nd Revision - authors' response 28 November 2019

Authors made the requested changes.

© European Molecular Biology Organization

EMBO PRESS

YOU MUST COMPLETE ALL CELLS WITH A PINK BACKGROUND

PLEASE NOTE THAT THIS CHECKLIST WILL BE PUBLISHED ALONGSIDE YOUR PAPER

Corresponding Author Name: Claude Libert
Journal Submitted to: EMBO Molecular Medicine
Manuscript Number: EMM-2019-11319

Reporting Checklist For Life Sciences Articles (Rev. June 2017)

This checklist is used to ensure good reporting standards and to improve the reproducibility of published results. These guidelines are consistent with the Principles and Guidelines for Reporting Preclinical Research issued by the NIH in 2014. Please follow the journal's authorship guidelines in preparing your manuscript.

A- Figures
1. Data
The data shown in figures should satisfy the following conditions:

- the data were obtained and processed according to the field's best practice and are presented to reflect the results of the experiments in an accurate and unbiased manner.
- figure panels include only data points, measurements or observations that can be compared to each other in a scientifically meaningful way.
- graphs include clearly labeled error bars for independent experiments and sample sizes. Unless justified, error bars should not be shown for technical replicates.
- if n< 5, the individual data points from each experiment should be plotted and any statistical test employed should be justified
- Source Data should be included to report the data underlying graphs. Please follow the guidelines set out in the author ship guidelines on Data Presentation.

2. Captions

Each figure caption should contain the following information, for each panel where they are relevant:

- a specification of the experimental system investigated (eg cell line, species name).
- the assay(s) and method(s) used to carry out the reported observations and measurements
- an explicit mention of the biological and chemical entity(ies) that are being measured.
- an explicit mention of the biological and chemical entity(ies) that are altered/varied/perturbed in a controlled manner.

- the exact sample size (n) for each experimental group/condition, given as a number, not a range;
- a description of the sample collection allowing the reader to understand whether the samples represent technical or biological replicates (including how many animals, litters, cultures, etc.).
- a statement of how many times the experiment shown was independently replicated in the laboratory.
- definitions of statistical methods and measures:
  - common tests, such as t-test (please specify whether paired vs. unpaired), simple χ2 tests, Wilcoxon and Mann-Whitney tests, can be unambiguously identified by name only, but more complex techniques should be described in the methods section;
  - are tests one-sided or two-sided?
  - are there adjustments for multiple comparisons?
  - exact statistical test results, e.g., P values = x but not P values < x;
  - definition of 'center values' as median or average;
  - definition of error bars as s.d. or s.e.m.

Any descriptions too long for the figure legend should be included in the methods section and/or with the source data.

In the pink boxes below, please ensure that the answers to the following questions are reported in the manuscript itself. Every question should be answered. If the question is not relevant to your research, please write NA (non applicable). We encourage you to include a specific subsection in the methods section for statistics, reagents, animal models and human subjects.

USEFUL LINKS FOR COMPLETING THIS FORM

http://www.antibodypedia.com
http://1degreebio.org
http://www.equator-network.org/reporting-guidelines/improving-bioscience-research-repo
http://grants.nih.gov/grants/olaw/olaw.htm
http://www.mrc.ac.uk/Ourresearch/Ethicsresearchguidance/Useofanimals/index.htm
http://ClinicalTrials.gov
http://www.consort-statement.org
http://www.consort-statement.org/checklists/view/32-consort/66-title
http://www.equator-network.org/reporting-guidelines/reporting-recommendations-for-tum
http://datadryad.org
http://figshare.com
http://www.ncbi.nlm.nih.gov/gap
http://www.ebi.ac.uk/ega
http://biomodels.net/
http://biomodels.net/miriam/
http://jjj.biochem.sun.ac.za
http://oba.od.nih.gov/biosecurity/biosecurity_documents.html
http://www.selectagents.gov/

B- Statistics and general methods

Please fill out these boxes (Do not worry if you cannot see all your text once you press return)

1.a. How was the sample size chosen to ensure adequate power to detect a pre-specified effect size?	Group sizes were determined based on previous experience.
1.b. For animal studies, include a statement about sample size estimate even if no statistical methods were used.	Group sizes were determined based on previous experience.
2. Describe inclusion/exclusion criteria if samples or animals were excluded from the analysis. Were the criteria pre-established?	NA
3. Were any steps taken to minimize the effects of subjective bias when allocating animals/samples to treatment (e.g. randomization procedure)? If yes, please describe.	No randomization was used to determine experimental groups.
For animal studies, include a statement about randomization even if no randomization was used.	No randomization was used to determine experimental groups.
4.a. Were any steps taken to minimize the effects of subjective bias during group allocation or/and when assessing results (e.g. blinding of the investigator)? If yes please describe.	No blinding of the investigator was performed.
4.b. For animal studies, include a statement about blinding even if no blinding was done	No blinding of the investigator was performed.
5. For every figure, are statistical tests justified as appropriate?	Yes
Do the data meet the assumptions of the tests (e.g., normal distribution)? Describe any methods used to assess it.	Samples were assumed to be normaly distrubuted.

Is there an estimate of variation within each group of data?	Samples were assumed to have similar variance between groups.
Is the variance similar between the groups that are being statistically compared?	Samples were assumed to have similar variance between groups.

C- Reagents

6. To show that antibodies were profiled for use in the system under study (assay and species), provide a citation, catalog number and/or clone number, supplementary information or reference to an antibody validation profile. e.g., Antibodypedia (see link list at top right), 1DegreeBio (see link list at top right).	PPARa antibody: catalog sc-398394, Santa Cruz Biotechnology; b-Actin loading control antibody: catalog MA5-15739, Life Technologies Europe
7. Identify the source of cell lines and report if they were recently authenticated (e.g., by STR profiling) and tested for mycoplasma contamination.	NA

* for all hyperlinks, please see the table at the top right of the document

D- Animal Models

8. Report species, strain, gender, age of animals and genetic modification status where applicable. Please detail housing and husbandry conditions and the source of animals.	Male mice (C57BL/6J) were ordered from Janvier (Le Genest-St.Isle, France) and were housed in light controlled (14 hours light; 10 hours dark), air-conditioned, specific pathogen free conditions with food and water ad libitum.
9. For experiments involving live vertebrates, include a statement of compliance with ethical regulations and identify the committee(s) approving the experiments.	All experiments were approved by the institutional ethics committee for animal welfare of the Faculty of Sciences, Ghent University, Belgium. The methods were carried out in accordance with the relevant guidelines and regulations.
10. We recommend consulting the ARRIVE guidelines (see link list at top right) (PLoS Biol. 8(6), e1000412, 2010) to ensure that other relevant aspects of animal studies are adequately reported. See author guidelines, under 'Reporting Guidelines'. See also: NIH (see link list at top right) and MRC (see link list at top right) recommendations. Please confirm compliance.	We confirm compliance.

E- Human Subjects

11. Identify the committee(s) approving the study protocol.	The clinical study protocol was approved by the ethics committee of the University Hospital of Ghent.
12. Include a statement confirming that informed consent was obtained from all subjects and that the experiments conformed to the principles set out in the WMA Declaration of Helsinki and the Department of Health and Human Services Belmont Report.	After admission to the ICU, thirteen patients were enrolled within 24 h after meeting the criteria for severe sepsis or septic shock defined at the consensus conference of 2001 (1) and after a signed informed consent was obtained from the patient itself or a legal representative. The experiments conformed to the principles set out in the WMA Declaration of Helsinki and the Department of Health and Human Services Belmont Report.
13. For publication of patient photos, include a statement confirming that consent to publish was obtained.	NA
14. Report any restrictions on the availability (and/or on the use) of human data or samples.	NA
15. Report the clinical trial registration number (at ClinicalTrials.gov or equivalent), where applicable.	NA
16. For phase II and III randomized controlled trials, please refer to the CONSORT flow diagram (see link list at top right) and submit the CONSORT checklist (see link list at top right) with your submission. See author guidelines, under 'Reporting Guidelines'. Please confirm you have submitted this list.	NA
17. For tumor marker prognostic studies, we recommend that you follow the REMARK reporting guidelines (see link list at top right). See author guidelines, under 'Reporting Guidelines'. Please confirm you have followed these guidelines.	NA

F- Data Accessibility

18: Provide a "Data Availability" section at the end of the Materials & Methods, listing the accession codes for data generated in this study and deposited in a public database (e.g. RNA-Seq data: Gene Expression Omnibus GSE39462, Proteomics data: PRIDE PXD000208 etc.) Please refer to our author guidelines for 'Data Deposition'.<br><br>Data deposition in a public repository is mandatory for:<br>a. Protein, DNA and RNA sequences<br>b. Macromolecular structures<br>c. Crystallographic data for small molecules<br>d. Functional genomics data<br>e. Proteomics and molecular interactions	RNA-seq data: Gene expression.<br>Deposited at the National Center for Biotechnology Information Gene Expression Omnibus public database (http://www.ncbi.nlm.nih.gov/geo/) under accession number GSE139484.
19. Deposition is strongly recommended for any datasets that are central and integral to the study; please consider the journal's data policy. If no structured public repository exists for a given data type, we encourage the provision of datasets in the manuscript as a Supplementary Document (see author guidelines under 'Expanded View' or in unstructured repositories such as Dryad (see link list at top right) or Figshare (see link list at top right).	Done.
20. Access to human clinical and genomic datasets should be provided with as few restrictions as possible while respecting ethical obligations to the patients and relevant medical and legal issues. If practically possible and compatible with the individual consent agreement used in the study, such data should be deposited in one of the major public access-controlled repositories such as dbGAP (see link list at top right) or EGA (see link list at top right).	NA
21. Computational models that are central and integral to a study should be shared without restrictions and provided in a machine-readable form. The relevant accession numbers or links should be provided. When possible, standardized format (SBML, CellML) should be used instead of scripts (e.g. MATLAB). Authors are strongly encouraged to follow the MIRIAM guidelines (see link list at top right) and deposit their model in a public database such as Biomodels (see link list at top right) or JWS Online (see link list at top right). If computer source code is provided with the paper, it should be deposited in a public repository or included in supplementary information.	NA

G- Dual use research of concern

22. Could your study fall under dual use research restrictions? Please check biosecurity documents (see link list at top right) and list of select agents and toxins (APHIS/CDC) (see link list at top right). According to our biosecurity guidelines, provide a statement only if it could.	NA
